# A synthetic methodology toward π-extended porphyrin-rylenediimide conjugates†

**DOI:** 10.1039/d4ra08045a

**Published:** 2025-01-15

**Authors:** Christoph Oleszak, Christian L. Ritterhoff, Erik J. Schulze, Andreas Hirsch, Bernd Meyer, Norbert Jux

**Affiliations:** a Department of Chemistry and Pharmacy & Interdisciplinary Center for Molecular Materials (ICMM), Chair of Organic Chemistry II, Friedrich-Alexander-Universität Erlangen-Nürnberg Nikolaus-Fiebiger-Str. 10 91058 Erlangen Germany norbert.jux@fau.de; b Interdisciplinary Center for Molecular Materials (ICMM), Computer Chemistry Center (CCC), Friedrich-Alexander-Universität Erlangen-Nürnberg Nägelsbachstr. 25 91052 Erlangen Germany bernd.meyer@fau.de

## Abstract

In this work, we present a straightforward synthetic route for the preparation of functionalized β-*meso*-fused porphyrins, which are subsequently connected to rylendiimides. The resulting donor–acceptor-type conjugates exhibit intriguing optical properties, such as panchromatism and profoundly bathochromically shifted absorption curves. A better understanding of the molecules' electronic structure was gained through density-functional theory calculations, which unveiled small HOMO–LUMO gaps.

## Abstract 2

A straightforward synthetic route for the preparation of functionalized β-*meso*-fused porphyrins, which are then connected to rylendiimides is demonstrated. The donor–acceptor-type conjugates show features of panchromaticity and small energy gaps.

## Graphical abstract

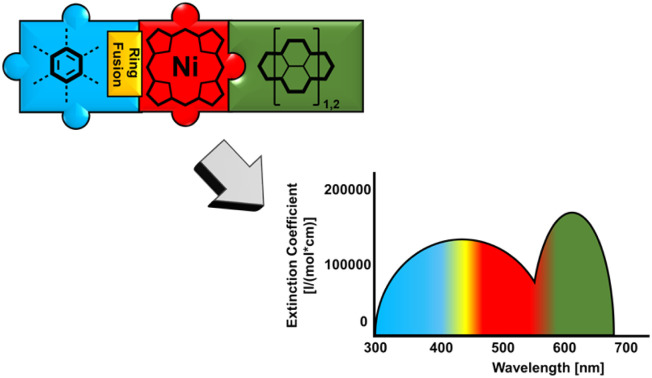

## Introduction

The last years have clearly demonstrated that one of the most significant challenges of modern society will be balancing the steadily rising demand for energy and reducing carbon emissions from fossil energy sources. Here, solar energy presents itself as one of the most promising alternatives in the form of photovoltaic (PV) technology, converting natural light into electricity. Even though the best-performing PV modules to this day rely on inorganic silicon solar cells, the research efforts toward organic molecule-based alternatives are more than significant.^1^ The underlying reason for this tremendous scientific interest in organic photovoltaic (OPV) technologies originates from their exceptional combination of features, namely, light weight, flexibility, easy processing, low cost, and, most importantly, low environmental impact.^2–4^ A typical organic solar cell (OSC) consists of an active layer containing an organic donor (D) and acceptor (A) moiety located between the anode and cathode.^5^ Most commonly, the active layer is fabricated as a so-called bulk heterojunction (BHJ) blend of two separate materials due to the very effective maximization of the donor–acceptor interfacial area, which can be achieved this way.^6,7^ Although these BHJ cells can reach PCE values of close to 20%, a major drawback lies in their thermodynamic instability of nanomorphology and progressive macrophase separation of donor and acceptor material, leading to relatively short lifetimes of the respective cells.^8,9^ An alternate strategy that circumvents this inherent problem is based on combining both the donor and acceptor sites of the active layer in one single molecule. The resulting architectures are known as single-material organic solar cells (SMOSCs). Due to the undeniable potential of SMOSCs, the design of such materials has gained tremendous attention in recent years.^10–12^ As a result, an extensive library of building blocks exists in literature, with porphyrins being one of their standout members. The 18π-electron macrocycle is one of nature's most important structural elements and plays a central role in the photosynthetic system, which strongly inspired researchers towards their application in OSCs.^13–18^ This is, of course, due to the unique properties of porphyrins, including strong absorption in the visible part of the solar spectrum, excellent tunability of optical and electronic features, remarkable stability, and efficient electron transfer.^19–24^ However, simple porphyrin chromophores showed little promise for a long time due to disappointing power conversion efficiencies. One of the main reasons for this is the severe lack of absorption between the Soret- and Q-band, strongly hampering the molecules' light harvesting abilities.^25,26^

A recent surge in interest was sparked by the introduction of modified porphyrin-based π-conjugated systems, which overcome the drawbacks of ordinary porphyrins and show vast improvements as OSC material.^27–29^ One of the most popular ways to tailor the properties of the porphyrins by further conjugating its π system is the direct coupling to other aromatic fragments *via* ethylene linkers.^30^ A much less prominent π-extension approach in the context of OPVs is connecting aromatics to the porphyrin periphery *via* ring closure.^31–42^ Nevertheless, related reports from literature in the context of OSCs include porphyrins fused to, for example, naphthalene,^43^ perylene,^44^ and anthracene.^45^ The exclusively six-ring fused porphyrins in these studies exhibit outstanding light-harvesting abilities, which can be attributed to their strongly red-shifted absorption and broadened Soret- and Q-bands. However, these species commonly suffer from low efficiencies, caused by aggregation and mismatched HOMO–LUMO levels.^46,47^ Recently in 2019, Imahori *et al.* tackled these shortfalls in the shape of donor–acceptor-type thiophene-fused porphyrins that bear bulky substituents in their periphery, achieving a fused-porphyrin record value of 10.7% in efficiency.^48^

**Fig. 1:**
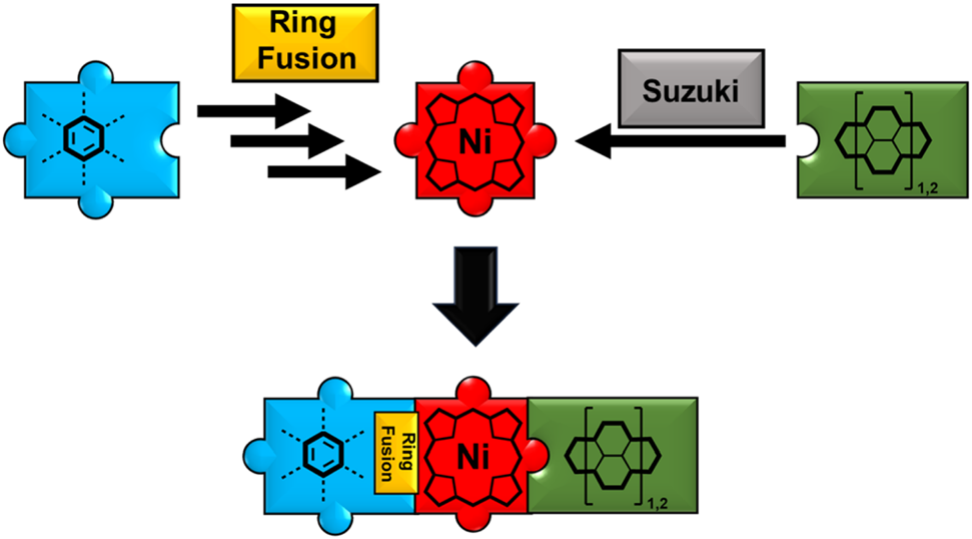
Visualization of the toolbox-like synthetic concept toward fused porphyrin-rylenediimide conjugates.

**Fig. 2:**
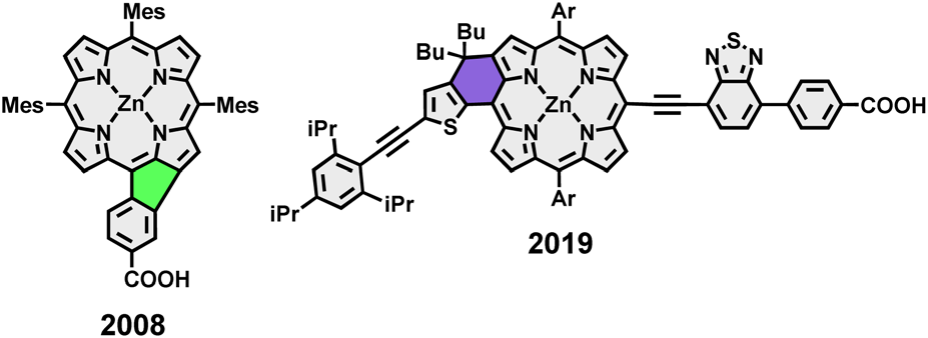
Examples from literature on the application of five-and six-ring fused porphyrins in solar cells.

Keeping this in mind, lately, many of our group's synthetic efforts have been focused on designing β-*meso* five-ring-fused porphyrin systems of varying sizes and shapes. These conjugates retained their excellent solution processability in common solvents while showing intriguing optoelectronic properties like panchromatic absorption reaching into the near-infrared area and drastically shrunk band gap energies. Furthermore, the synthetic strategy remained straightforward with few steps and good yields.^49,50^ Although the application of five-ring fused species in the field of OPVs is scarcely reported in literature, it appears to be a viable, yet not well explored strategy as demonstrated by Imahori *et al.* in their work on a rather simple phenyl-fused carboxylic acid porphyrin in 2008 (Fig. 2).^51^ The synthetic challenge in generating more elaborate donor–acceptor-type molecules now springs from the need for further post-functionalization of the fused systems. We tackled this by developing a synthetic route for fused porphyrins bearing a boronic ester in one of its *meso*-positions, making subsequent Suzuki cross-coupling reactions possible. To demonstrate the feasibility of this approach, a library of dyad and triad model compounds, resembling D–A and D–A–D architectures, respectively, was synthesized. Naphthalenediimide (NDI) and perylenediimide (PDI)^52–56^ building blocks were hereby employed as the covalently linked acceptor moieties (Fig. 1).^57–61^

## Results and discussion

**Scheme 1:**
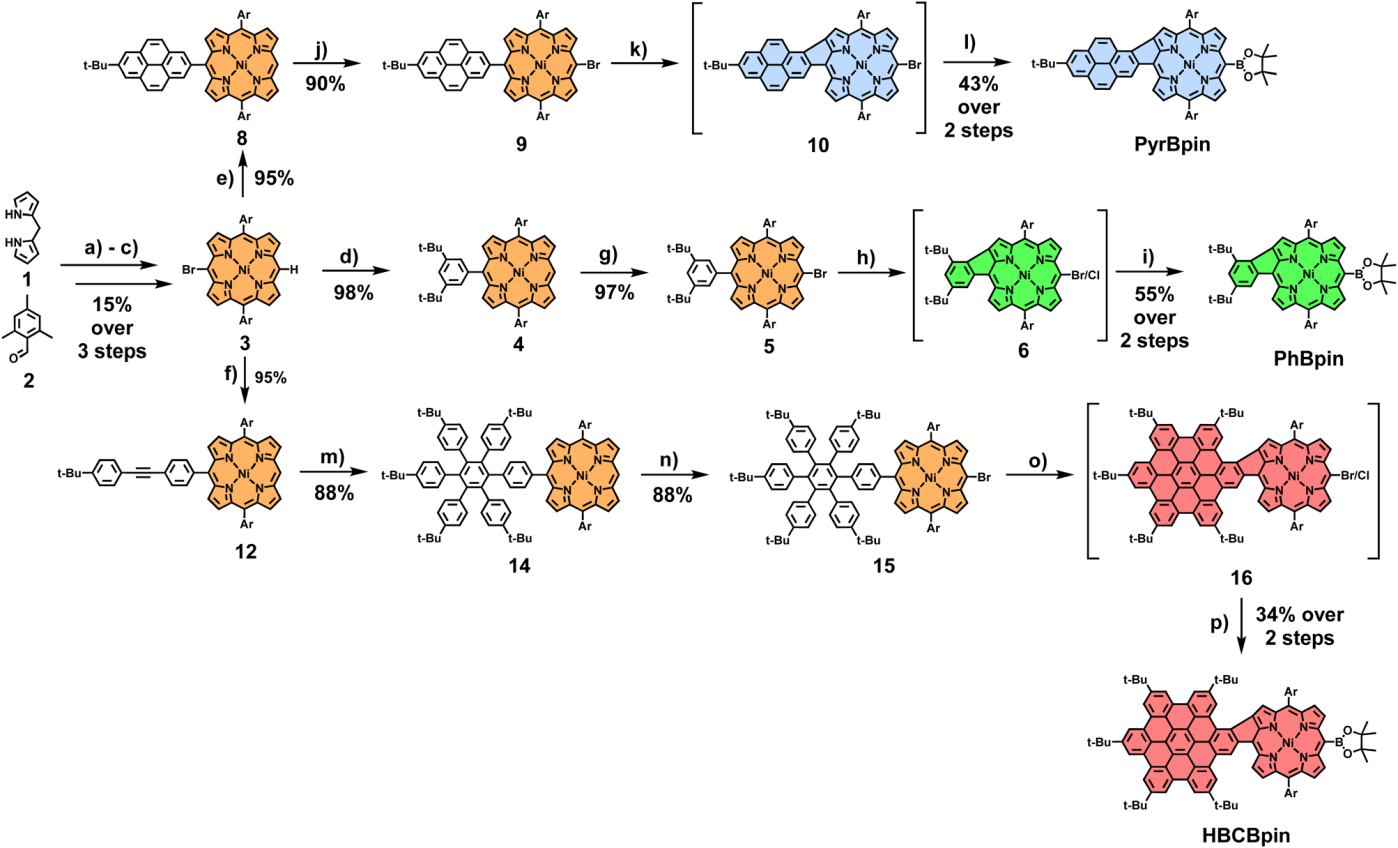
Synthesis of building blocks PhBpin, PyrBpin and HBCBpin. Reagents and conditions: (a) (1) BF_3_^.^OEt_2_, CHCl_3_, 3 h, rt, (2) DDQ, 30 min, rt; (b) NBS, CHCl_3_, pyridine, 25 min, 0 °C; (c) Ni(acac)_2_, toluene, 6 h, 140 °C; (d) 3,5-di-*tert*-butylphenylboronic acid, Pd(PPh_3_)_4_, Cs_2_CO_3_, toluene/DMF 2 : 1, 18 h, 80 °C; (e) 2-bromo-7-(*tert*-butyl)pyrene 7, Pd(PPh_3_)_4_, Cs_2_CO_3_, toluene/DMF 2 : 1, 18 h, 80 °C; (e) 2-(4-((4-(*tert*-butyl)phenyl)ethynyl)phenyl)-4,4,5,5-tetramethyl-1,3,2-dioxaborolane 11, Pd(PPh_3_)_4_, Cs_2_CO_3_, toluene/DMF 2 : 1, 18 h, 80 °C; (g) NBS, CHCl_3_, pyridine; (h) FeCl_3_ (8 equiv.), CH_3_NO_2_, CH_2_Cl_2_, 24 h, 0 °C → rt; (i) HBpin, PdCl_2_(PPh_3_)_2_, NEt_3_, 1,2-DCE, 24 h, 90 °C; (j) NBS, CHCl_3_, pyridine; (k) FeCl_3_ (8 equiv.), CH_3_NO_2_, CH_2_Cl_2_, 1 h, 0 °C → rt; (l) HBpin, PdCl_2_(PPh_3_)_2_, NEt_3_, 1,2-DCE, 24 h, 90 °C; (m) tetracyclone 13, 260 °C, μW, 24 h; (n) NBS, CHCl_3_, pyridine; (o) FeCl_3_ (30 equiv.), CH_3_NO_2_, CH_2_Cl_2_, 48 h, 0 °C → rt; (p) HBpin, PdCl_2_(PPh_3_)_2_, NEt_3_, 1,2-DCE, 24 h, 90 °C. Ar = mesityl; BPin = boronic acid pinacol ester.

To illustrate our concept's versatility, three distinct borylated fused porphyrin building blocks of varying π-system size were initially synthesized. Phenyl-fused PhBpin, pyrene-fused PyrBpin, and hexa-*peri*-hexabenzocoronene (HBC)-fused HBCBpin molecules were obtained in a straightforward manner. The general synthetic procedure starts from brominated nickel-dimesityl-porphyrin 3, which was synthesized in three steps (for experimental details, see Scheme S1†). The two *meso*-bound mesityl groups in this key starting molecule prevent any kind of unwanted side reactions during the subsequent steps. In particular, undesirable β-*meso*-fusions are rendered impossible by the position of the methyl groups in the mesityl substituents. The introduction of the nickel ion into the porphyrin is necessary to enable the crucial penultimate reaction step of the laid-out cascade, which fuses porphyrin-core and *meso*-coupled aromatic. This respective aromatic fragment (phenyl/pyrene/HBC) was then introduced *via* a Suzuki cross-coupling reaction. In the case of PhBpin and PyrBpin, this works directly by employing borylated phenyl and pyrene precursors (for comprehensive experimental details, see ESI Sections 2.1 and 2.2†). For **HBCBpin**, on the other hand, a two-step procedure is necessary, firstly introducing a tolane 11 to the porphyrins *meso*-position, which is subsequently converted to a hexaarylbenzene (HAB) by a Diels–Alder reaction. The *meso*-position opposing the aromatic fragment was brominated, and the resulting conjugates were subjected to oxidative cyclodehydrogenation conditions. The conversion to the respective β-*meso* five-ring-fused equivalent was successful for all three molecules by dissolving them in a mixture of CH_2_Cl_2_ and CH_3_NO_2_ and the addition of differing amounts of iron(iii)chloride at 0 °C, followed by stirring under slow warming to room temperature for varying amounts of time. For phenyl-substituted 5, 8 equivalents of FeCl_3_ with a reaction time of 24 h were necessary for full conversion of the starting material. The resulting mixture of *meso*-brominated and -chlorinated fused porphyrin 6 was then directly converted into PhBpin, which was obtained in a total yield of 55% after the two reaction steps. Pyrene-bearing 9, in comparison, appeared to be much more reactive and prone to overchlorination side reactions. Therefore, the reaction time was reduced to 1 h, using 8 equivalents of FeCl_3_ in a more diluted solution. Due to the much shorter reaction times, no *meso*-chlorinated side product was observed, and the follow-up transformation of 9 into PyrBpin proceeded smoothly with a 43% yield over two steps. Lastly, for HAB-coupled 15, six additional bonds had to be formed during the reaction, resulting in elongated reaction times of 48 h, and 60 equivalents of FeCl_3_ were needed for sufficient conversion. Again, borylation of the resulting halogen mixture 16 yielded the final building block HBCBpin in 34% yield (Scheme 1).

**Scheme 2:**
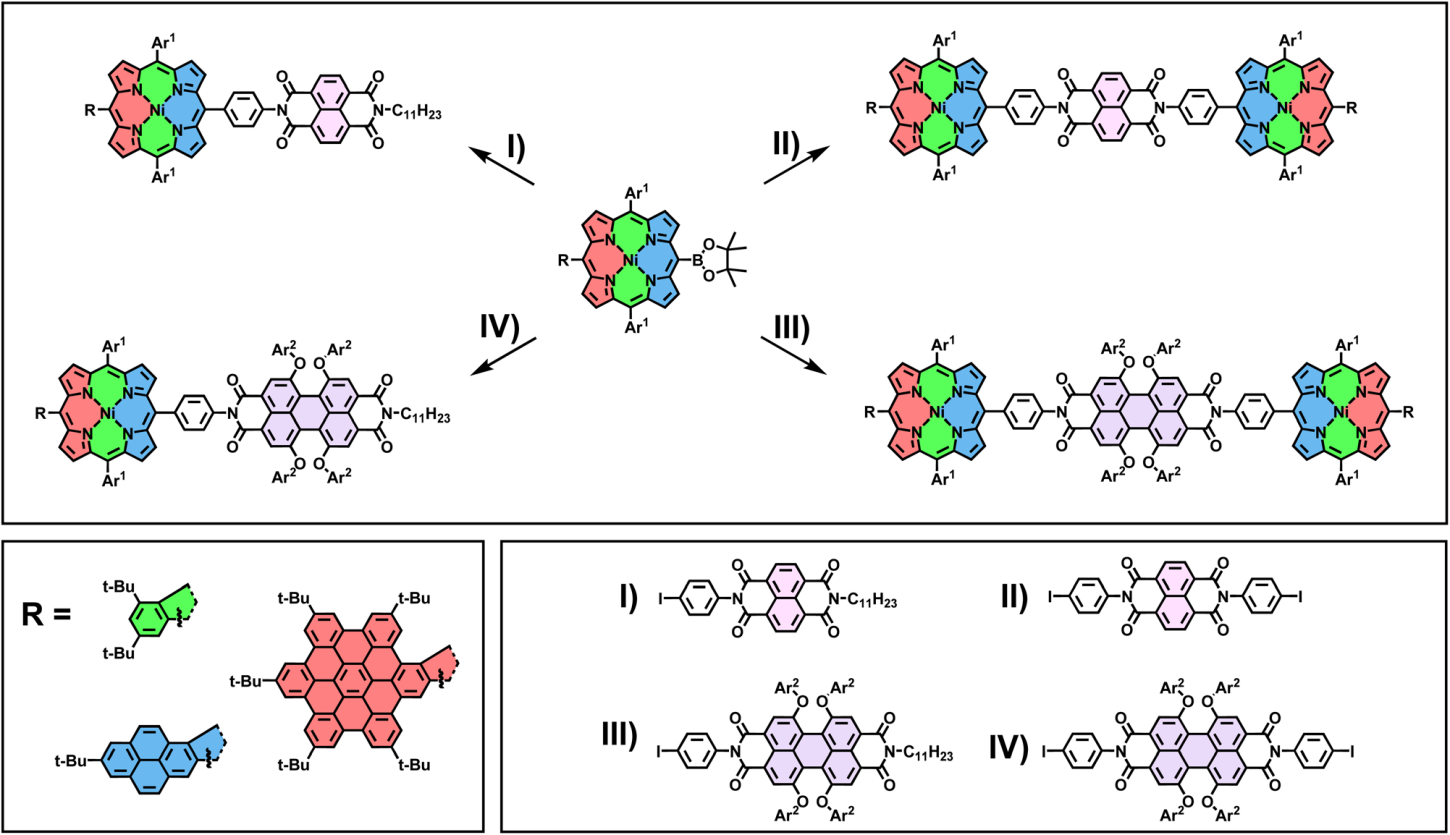
Reagents and conditions: Pd(PPh_3_)_4_, Cs_2_CO_3_, toluene/DMF 2 : 1, 18 h, 80 °C. Ar^1^ = mesityl; Ar^2^ = 4-*tert*-butylphenyl.

With the borylated porphyrins in hand, a subsequent Suzuki coupling with four different tailor-made halogen-functionalized rylenediimides I–IV was envisioned (for experimental details on the synthesis of I–IV, see ESI Section 2.4†). To this end, a general procedure for one- and two-fold coupling between the respective fragments was developed. The porphyrin and rylenediimide were dissolved in a mixture of toluene/DMF (ratio 2 : 1), and Cs_2_CO_3_ and Pd(PPh_3_)_4_ were added. After stirring the mixture for 18 h at 80 °C and removing the inorganic salts by filtration through silica, the final products were obtained *via* size exclusion chromatography on a Biobeads SX1 polymer (Scheme 2). Early during the screening for optimized reaction conditions, it became apparent that for perylenediimides (PDI) III and IV, additional solubilizing groups (*tert*-butyl-phenoxyethers) in the bay positions are necessary to make the Suzuki cross-coupling work under the above-mentioned conditions. For the naphthalenediimides (NDI) I and II, this modification is not required. All twelve possible conjugates were obtained following the laid-out synthetic procedure, with yields reaching from 20% to 68% for the D–A dyads (I and III) and 16% to 28% for the D–A–D triads (II and IV).

**Fig. 3:**
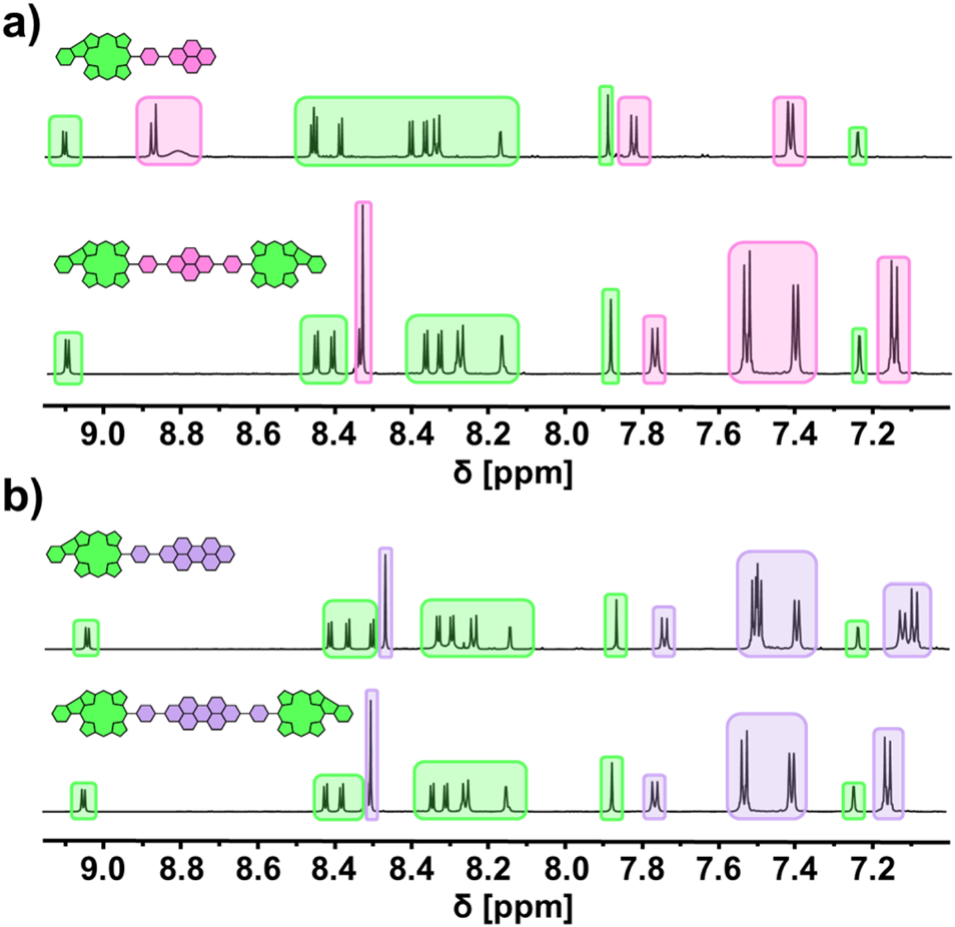
^1^H NMR spectra (aromatic region) of phenyl-fused conjugates (a) Ph-NDI (top) and Ph-NDI-Ph (bottom); (b) Ph-PDI (top) and Ph-PDI-Ph (bottom). The signals are highlighted according to the fragment they originate from: fused porphyrin (green), NDI (pink), and PDI (purple). Solvent: CD_2_Cl_2_.

All species were unambiguously identified and characterized by means of NMR and UV/vis absorption spectroscopy as well as mass spectrometric techniques. Exemplary, the aromatic areas of the ^1^H NMR spectra of the conjugates derived from PhBpin are shown in Fig. 3 to demonstrate the general spectral characteristics of the dye library (for ^1^H, ^13^C, and 2D spectra of all conjugates, see ESI†). Comparing the NDI-based species with the PDI-based ones, it becomes apparent that the magnetic environment of the fused porphyrin is only marginally influenced by the coupled rylenediimide. The respective signal splitting and chemical shift remain very similar (green) across all spectra. On the contrary, the NDI (pink) and PDI (purple) signals get altered between the dyads and triads due to the symmetry changes. However, the chemical shifts stay within the same area, implying a consistent magnetic influence received from the fused porphyrin.

**Fig. 4:**
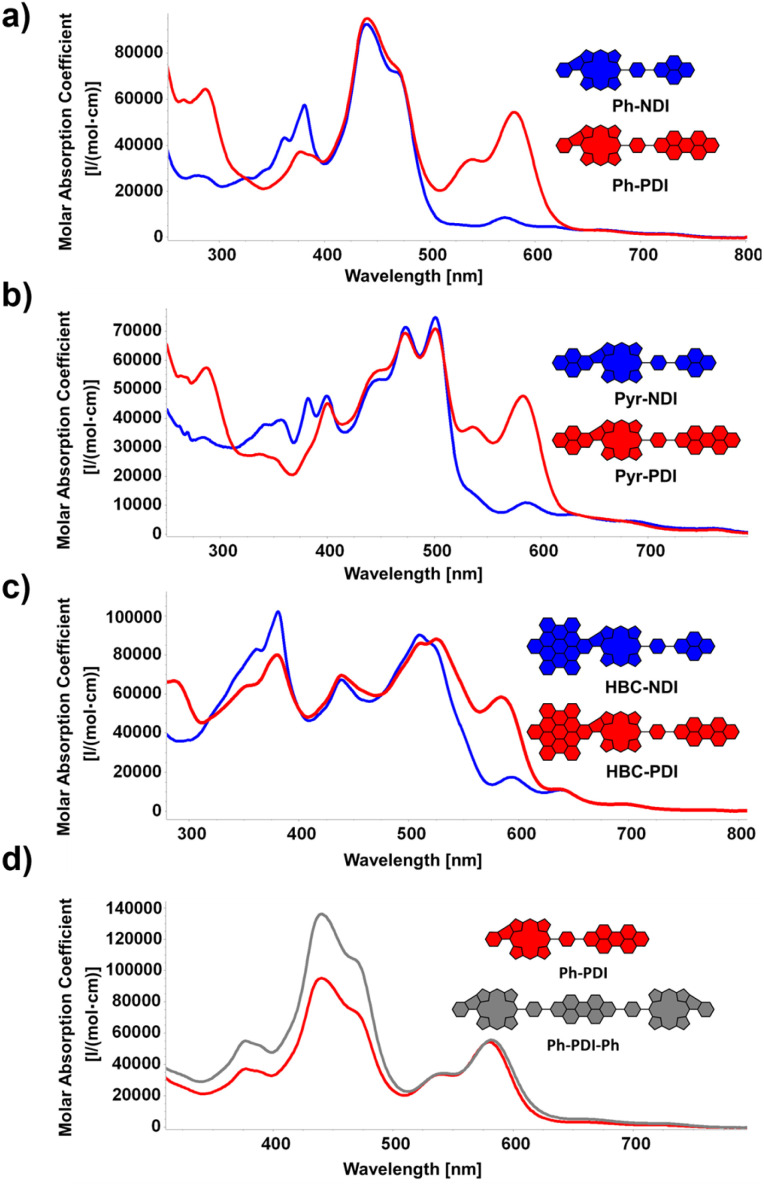
(a)–(c) UV/vis spectra of the D–A dyads. Solvent: CH_2_Cl_2_. (d) Comparison of UV/vis spectra of dyad Ph-PDI and respective triad Ph-PDI-Ph. Solvent: CH_2_Cl_2_.

By probing the photophysical features of the D–A dyads *via* UV/vis experiments, insights into the electronic structure of the conjugates were gathered. To this end, the influence of the two different rylendiimides (NDI: blue; PDI: red) on the absorption properties of the three differently sized π-extended fragments was investigated (Fig. 4a–c). For the smallest molecule Ph-NDI, shown in Fig. 4a, the prominent characteristic absorption band of the fused porphyrins dominates the spectra, covering primarily the area between 250 and 500 nm. Additional absorption peaks in the area between 300 and 400 nm can be observed, stemming from the NDI absorption (for reference, see the spectra of the individual building blocks in the ESI†). Comparing the integral of the absorption area from 250–800 nm to the precursor PhBpin, a relatively minor increase of 6% is found, which is explained by the strong overlap of the porphyrin absorption and the one of the NDI moiety. For Ph-PDI, on the other hand, the situation changes drastically, with the covered absorption area increasing by 50%. This is caused by the strong absorption of the PDI between 500 and 650 nm, with a strong local absorption maximum at 580 nm. Again, the rest of the absorption curve stays extremely reminiscent of the one of PhBpin, demonstrating and confirming the successful electronic ground-state separation of the fused porphyrin and the rylenediimide *via* the phenyl spacer. In Fig. 4b, similar tendencies can be observed. The pyrene-fused congeners Pyr-NDI and Pyr-PDI show an absorption area increase of 8% and 30%, respectively, compared to PyrBpin. The relative increase for the perylenediimide conjugate is significantly less pronounced than for the previously discussed Ph-PDI. This can be attributed to the enhanced near infrared (NIR) absorption of the fused pyrene porphyrin, which is caused by the size increase of the π-system. As a result, the spectral overlap of the two individual aromatic fragments is increased. These observed trends also stay true for the HBC-fused conjugates Fig. 4c.

A further increase in absorption can be expected for the D–A–D triads. Exemplary for the library of molecules synthesized, Ph-PDI-Ph (grey) was chosen and compared to the respective dyad Ph-PDI (red). The spectral shape of the two molecules remains basically identical, while the absorption originating from the extended porphyrin core is vastly increased for Ph-PDI-Ph. Quantifying this observation, a total rise in absorption area of around 30% can be claimed. Fluorescence was not detected at any wavelength for the conjugates. This behavior is in general expected for nickel-porphyrins, attributed to the heavy atom effect.^24^ However, our own experiments from the past and reports from the literature indicate, that in the case of five-ring fused porphyrins, free-base, and zinc derivatives also undergo a non-fluorescent, alternate, deactivation pathway, originating from the macrocycle's altered electronic properties.^32,49,50^ Namely, our spectroscopic studies on HBC- and phenyl-fused congeners revealed that the additional fused ring promotes biradicaloid and anti-aromatic character in the π-system, which leads to the observed quick, non-radiative deactivation. Although in principle hampering the application in OPVs, said process does by no means strictly prevent that, as was shown in the works of Imahori on similar fused species.^51^ Nevertheless, this circumstance presents a major challenge for the herein presented kind of molecules, which has to be potentially accounted for by further chemically tailoring the donor–acceptor moiety. Moreover, further probing is needed to evaluate the precise influence of the central metal on the conjugates' photophysical behavior and connected to this the possible necessity of changing the metal centers in the herein-presented porphyrins, as nickel-porphyrin-based OSC materials exist but are less common than zinc-centered ones.^46,47^ Still, the non-fluorescence of the PDI in the respective conjugates hints at ongoing interactions with the porphyrin in the excited state. This has to be further investigated in the future to be able to give proper insights into the ongoing processes.

DFT calculations were performed to elucidate the electronic structure of the conjugates. A clear spatial separation of the frontier orbitals is observed for all architectures. The HOMO is exclusively located on the porphyrin moiety. At the same time, the LUMO is always situated on the respective rylenediimide, indicating successful electronic decoupling of the two aromatic systems *via* the phenyl spacer group. Moreover, the contour plots of both HOMO and LUMO for all fused compounds resemble very closely those of their building blocks (see Fig. S116–S127 in the ESI†). The same holds true for the respective orbital energies (see Tables S1–S3 in the ESI†): the energy of the HOMO of the fused porphyrins is almost exactly preserved, while the LUMO level of the rylenediimides is only slightly lowered in the dyads and triads.

**Fig. 5:**
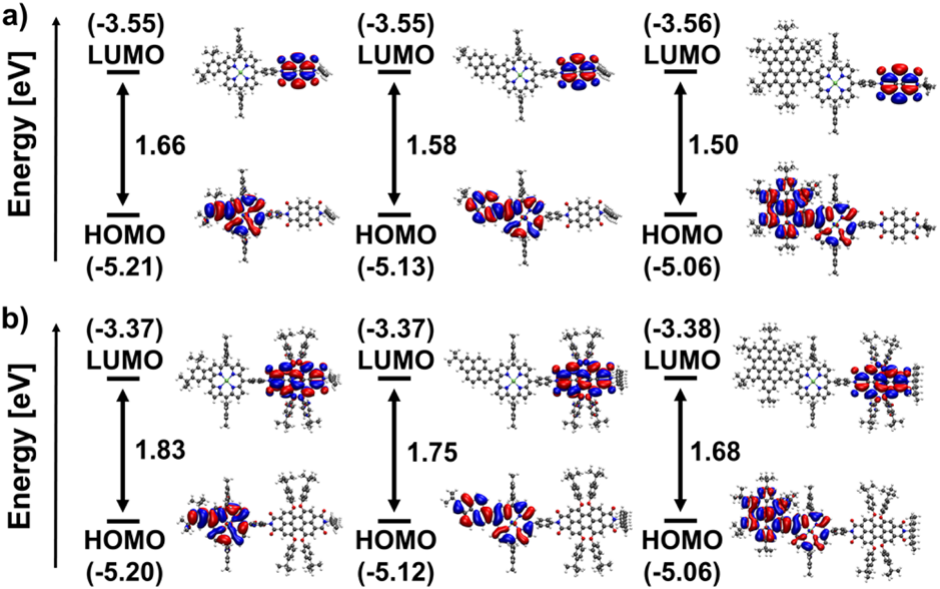
Eigenvalues and orbital contours of the HOMO and LUMO of (a) the NDI-based dyads Ph-NDI, Pyr-NDI, and HBC-NDI (left to right); (b) the PDI-based dyads Ph-PDI, Pyr-PDI, and HBC-PDI (left to right) in the gas phase calculated at DFT (B3LYP/def2-TZVPP) level of theory including an implicit solvation model.

**Fig. 6:**
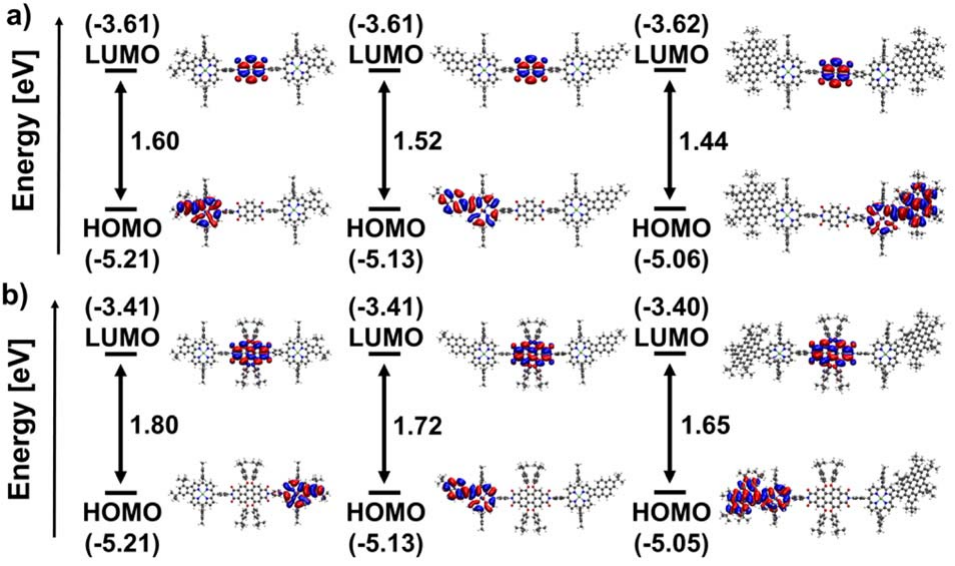
Eigenvalues and orbital contours of the HOMO and LUMO of (a) the NDI-based triads Ph-NDI-Ph, Pyr-NDI-Pyr, and HBC-NDI-HBC (left to right); (b) the PDI-based triads Ph-PDI-Ph, Pyr-PDI-Pyr, and HBC-PDI-HBC (left to right) in the gas phase calculated at DFT (B3LYP/def2-TZVPP) level of theory including an implicit solvation model.

Within the series of naphthalenediimide-based species, shown in Fig. 5a, a progressive reduction in gap energy is observed, which correlates directly with the size of the aromatic fragment fused to the porphyrin. The gap decreases from 1.66 eV for Ph-NDI to 1.58 eV for Pyr-NDI and 1.50 eV for HBC-NDI in steps of 0.08 eV. This can be attributed primarily to the upward shift in the HOMO energy caused by the increasing size of the porphyrins π-system, as can be seen for the HOMO levels of the fused porphyrin constituents. On the other hand, the LUMO level stays basically the same as in the naphthalenediimide due to its electronic decoupling from the porphyrin building block. The same trend is observed for perylenediimide as the acceptor functionality (Fig. 5b). Again, the gap energy decreases from Ph-PDI (1.83 eV) to Pyr-PDI (1.75 eV) to HBC-PDI (1.68 eV) in almost equal steps, while the overall gap energies are around 0.17 eV larger than for the respective naphthalenediimide analogues. The results for frontier orbitals and HOMO–LUMO gaps for the NDI and PDI triads shown in Fig. 6 mirror those for the dyads from Fig. 5 in basically all the mentioned aspects. However, a slight decrease in gap energy across all conjugates of 0.06 eV for the NDIs and 0.03 eV for the PDIs due to slightly lower lying LUMO levels can be noted.

## Conclusions

In summary, we demonstrated a modular synthetic pathway for the synthesis of strongly absorbing donor–acceptor dyes, whose structure is based on π-extended porphyrins and rylenediimides. A library of dyads and triads was prepared by combining three differently sized β-*meso*-fused porphyrins with naphthalenediimides and perylenediimides. Hereby, the general procedure relied on the synthesis of novel porphyrin boronic ester building blocks, which were connected afterwards to the acceptor moiety *via* Suzuki cross-coupling reactions. The obtained conjugates were investigated regarding their optical properties, revealing a pronounced panchromatism that is achieved by the overlap of the absorption of the two aromatic fragments. DFT calculations reveal a complete electronic decoupling of the constituent building blocks *via* the phenyl spacer group. Both the contour plots and energies of the frontier orbitals of all dyads and triads are close to an exact superposition of their respective building block counterpart. Subsequently, the frontier orbitals are spatially completely separated from each other, with the HOMO and the LUMO being localized exclusively on the fused porphyrin and the rylenediimide, respectively. The straightforward synthesis route and great solubility of the molecules make them interesting candidates for model compounds used in OSCs as well as NIR dyes. The modular nature of our synthetic approach opens up the possibility of extending the method's scope by using other well-defined molecular building blocks in the same style. However, the overcoming of the short excited-state lifetimes, as well as the influences of the central metal ion and the details of the porphyrin-RDI interaction have to be further investigated. Experiments in this context, as well as further probing of the charge-transfer features and the evaluation of potential device applicability of the presented molecules, are the subject of ongoing research in our group.

## Data availability

The data supporting this article have been included as part of the ESI.†

## Conflicts of interest

There are no conflicts to declare.

## Supplementary Material

RA-015-D4RA08045A-s001
