# Supplementary material for: A synthetic methodology toward π-extended porphyrin-rylenediimide conjugates
Source: RSC Adv. 2025 Jan 15;15(2):1212–9. doi: 10.1039/d4ra08045a (PMC11733737; doi:10.1039/d4ra08045a)
Supplement: RA-015-D4RA08045A-s001 [file RA-015-D4RA08045A-s001.pdf]

Supplementary Information (SI) for RSC Advances.
This journal is © The Royal Society of Chemistry 2025

Table of Content

1 General Information S2

2 Synthetic Procedures S3

2.1 Synthesis of Phenyl-Fused Porphyrin-Precursor S3

2.2 Synthesis of Pyrene-Fused Porphyrin-Precursor S8

2.3 Synthesis of HBC-Fused Porphyrin-Precursor S11

2.4 Synthesis of Naphthalene- and Perylenediimides S15

2.5 Synthesis of Donor-Acceptor Dyads S20

2.6 Synthesis of Donor-Acceptor-Donor Triads S27

3 Spectral Appendix S33

4 DFT Calculations S144

5 References S158

1 General Information

All chemicals were purchased from Sigma-Aldrich and used without any further purification. Solvents were distilled prior to usage. Dichloromethane was neutralized with K_2CO_3 before distillation. Thin layer chromatography (TLC) was performed on Merck silica gel 60 F524, detected by UV-light (254 nm, 366 nm). Column chromatography and flash column chromatography were performed on Macherey-Nagel silica gel 60 M (deactivated, 230−400 mesh, 0.04−0.063 mm). NMR spectroscopy was performed on Bruker Avance Neo Cryo-Probe DCH (^{1}H: 600 MHz, ^{13}C: 150 MHz), Bruker Avance Neo 500 (^{1}H: 500 MHz, ^{13}C: 126 MHz) and Bruker Avance 400 (^{1}H: 400 MHz, ^{13}C{^{1}H}: 101 MHz). Deuterated solvents were purchased from Sigma-Aldrich and used as received. Chemical shifts are referenced to residual protic impurities in the solvents (^{1}H: $CHCl_3$: 7.24 ppm) and (^{1}H: CH_2Cl_2: 5.32 ppm) or the deuterated solvent itself (^{13}C{^{1}H}: $CDCl_3$: 77.0 ppm) and (^{13}C{^{1}H}: CD_2Cl_2: 53.8 ppm). The resonance multiplicities are indicated as “s” (singlet), “d” (doublet), “t” (triplet), “q” (quartet) and “m” (multiplet). Signals referred to as “bs” (broad singlet) are not clearly resolved or significantly broadened. IR spectra were recorded on a Bruker FT-IR Tensor 27 spectrometer with a Pike MIRacle ATR unit. LDI/MALDI-ToF mass spectrometry was performed on a Bruker Ultraflex Extreme machine. In case of MALDI, the following matrices were used: 2,5-dihydroxybenzoic acid (DHB) or *trans*-2-[3-(4-*tert*-butylphenyl)-2-methyl-2-propenyl-idene]malononitrile (DCTB). High-resolution mass spectrometry (MS) was performed on an ESI/APPI-ToF mass spectrometer Bruker maXis 4G UHR MS/MS spectrometer, a Bruker micrOTOF II focus TOF MS spectrometer, or on a MALDI-ToF Bruker Ultraflex Extreme spectrometer. Microwave reactions were carried out in a monomode microwave reactor Biotage Initiator+ with an external IR surface temperature sensor. The microwave-assisted reactions were carried out exclusively in the fixed hold-time mode using an external IR temperature sensor. UV/vis spectroscopy was carried out on a Varian Cary 5000 UV−vis−NIR spectrometer.

2 Synthetic Procedures

2.1 Synthesis of Phenyl-Fused Porphyrin-Precursor

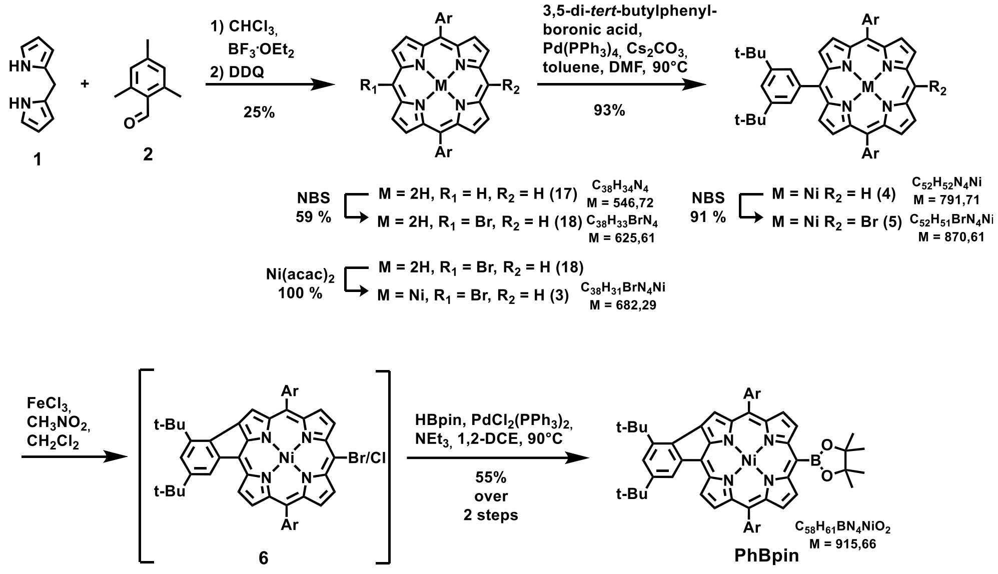

Scheme S1. Synthesis of phenyl-fused porphyrin building block **PhBpin**. Ar = mesityl.

5,15-Dimesitylporphyrin 17

Adapting a procedure from Chen *et al.,*[1] Ethanol (4.5 mL) stabilized $CHCl_3$ (600 mL) was degassed for 15 min (bubbling N_2 through the solution). Dipyrromethane **1** (890 mg, 6.00 mmol, 1 equiv) and mesitaldehyde **2** (885 µL, 6.00 mmol, 1 equiv) were added to the solution, and the reaction was degassed for another 10 min. $BF_3{\cdot}OEt_2$ (500 µL) was added, and the solution was stirred for 3 h at rt under the exclusion of light. DDQ (2.04 g, 9.00 mmol, 3 equiv) was added, and the mixture was stirred for a further 30 min. The acid was quenched via the addition of NEt_3 (8 mL), and the solvent was removed under reduced pressure. The crude was purified by filtration through silica (SiO_2, hexanes/CH_2Cl_2, 1:1, Ø 13 x 8 cm). The product **17** was obtained as a purple crystalline solid in 25% yield (407 mg, 744 µmol).

^{1}H NMR (400 MHz, CD_2Cl_2, rt): δ [ppm]: 10.25 (s, 2H), 9.37 (d, *J* = 4.6 Hz, 4H), 8.86 (d, *J* = 4.6 Hz, 4H), 7.35 (s, 4H), 2.66 (s, 6H), 1.84 (s, 12H), -3.13 (s, 2H).

^{13}C{^{1}H} NMR (101 MHz, $CDCl_3$, rt): δ [ppm]: 139.34, 138.00, 137.47, 131.85, 129.92, 127.80, 117.31, 104.45, 21.36, 21.19.

HRMS (MALDI, DCTB) for $C_{38}H_{34}N_4$ (M^+), calcd.: 546.2778, found: 546.2793.

Nickel-5,15-Dimesityl-10-Bromoporphyrin 3

Adapting a procedure from Mishra *et al.*[2], 5,15-Dimesitylporphyrin **17** (410 mg, 750 µmol, 1 equiv) was dissolved in $CHCl_3$ (200 mL) and pyridine (320 µL). The mixture was cooled to 0 °C, and NBS (133 mg, 750 µmol, 1 equiv) was added. After stirring for 25 min, the reaction was quenched with acetone (10 mL). The solution was washed with H_2O (100 mL) and subsequently dried over Na_2SO_4. The crude was separated by column chromatography (SiO_2, hexanes/CH_2Cl_2, 3:1, Ø 10 x 30 cm, 2nd band). The product was obtained as a purple crystalline solid in 59% yield (278 mg, 444 µmol). 5,15-Dimesityl-10-Bromoporphyrin **18** (226 mg, 361 µmol, 1 equiv) and Ni(acac)$_2$ (464 mg, 1.81 mmol, 5 equiv) were dissolved in toluene (40 mL). The mixture was heated to reflux for 6 h (heat-on temperature: 140 °C). The solvent was removed under reduced pressure, and the crude was purified by silica plug filtration (SiO_2, CH_2Cl_2, Ø 3.5 cm x 12 cm). After removal of the solvent, the residue was dissolved in CH_2Cl_2 (10 mL), and the product precipitated with MeOH (50 mL). The precipitate was filtered off and dried *in vacuo*. The product **3** was obtained as a red-brown solid in 100% yield (246 mg, 361 µmol).

^{1}H NMR (400 MHz, CD_2Cl_2, rt): δ [ppm]: 9.80 (s, 1H), 9.55 (d, *J* = 4.9 Hz, 2H), 9.10 (d, *J* = 4.8 Hz, 2H), 8.67 (m, 4H), 7.26 (s, 4H), 2.59 (s, 6H), 1.78 (s, 12H).

^{13}C{^{1}H} NMR (101 MHz, CD_2Cl_2, rt): δ [ppm]: 143.45, 143.26, 142.85, 142.19, 138.86, 138.08, 136.72, 133.30, 132.94, 131.99, 131.85, 127.79, 117.69, 104.86, 21.12, 21.00.

UV/Vis (CH_2Cl_2): *λ* [nm] (*ε* [$M^{-1}cm^{-1}$]): 409 (150000), 523 (12000).

HRMS (MALDI, DCTB) for $C_{38}H_{31}N_4NiBr$ (M^+), calcd.: 680.1080, found: 680.1070.

TLC: R_f [%]: 0.60 (hexanes/CH_2Cl_2 4:1).

Nickel-5,15-Dimesityl-10-(3,5-di-*tert*-butylphenyl)-Porphyrin 4

Nickel-5,15-Dimesityl-10-Bromoporphyrin **3** (260 mg, 382 µmol, 1 equiv), 3,5-di-*tert*-butylphenyl boronic acid (179 mg, 764 µmol, 2 equiv), Cs_2CO_3 (375 mg, 1.15 mmol, 3 equiv) and $Pd(PPh_3)_4$ (88 mg, 76 µmol, 0.2 equiv) were dissolved in toluene (20 mL) and DMF (10 mL) and were degassed. The reaction mixture was heated to 90 °C for 18 h. After cooling to rt, the solvent was removed under reduced pressure, and the crude was subjected to silica plug filtration (hexanes/CH_2Cl_2 - 1:1, Ø 3 x 12 cm). The product **4** was obtained in 93% yield (242 mg, 355 µmol).

^{1}H NMR (400 MHz, $CDCl_3$, rt): δ [ppm]: 9.78 (s, 1H), 9.09 (d, *J* = 4.7 Hz, 2H), 8.77 (d, *J* = 4.9, 2H), 8.71 (d, *J* = 4.7 Hz, 2H), 8.62 (d, *J* = 4.9, 2H), 7.91 (d, *J* = 1.8, 2H), 7.72 (m, 1H), 7.20 (s, 4H), 2.57 (s, 6H), 1.80 (s, 12H), 1.46 (s, 18H).

^{13}C{^{1}H} NMR (101 MHz, $CDCl_3$, rt): δ [ppm]: 148.80, 142.87, 142.81, 142.67, 142.49, 140.23, 139.08, 137.66, 137.42, 132.75, 132.34, 131.40, 130.79, 129.00, 127.74, 121.00, 120.42, 116.92, 104.10, 35.03, 31.70, 21.43, 21.40.

UV/Vis (CH_2Cl_2): *λ* [nm] (*ε* [$M^{-1}cm^{-1}$]): 408 (225000), 521 (16000).

HRMS (MALDI, DCTB) for $C_{52}H_{52}N_4Ni$ (M^+), calcd.: 790.3540, found: 790.3529.

TLC: R_f [%]: 0.70 (hexanes/CH_2Cl_2 3:1).

Nickel-5,15-Dimesityl-10-(3,5-di-*tert*-butylphenyl)-20-Bromoporphyrin 5

To a solution of $CHCl_3$ (25 mL), pyridine (600 µL) and Nickel-5,15-Dimesityl-10-(3,5-di-*tert*-butylphenyl)-porphyrin **4** (300 mg, 380 µmol, 1 equiv) NBS (68 mg, 380 µmol, 1 equiv) in $CHCl_3$ (7.5 mL) was added slowly at rt. The mixture was stirred for 15 min at rt before the reaction was quenched with acetone (8 mL). The solvents were removed under reduced pressure, and the crude was purified by silica plug filtration (hexanes/CH_2Cl_2 - 1:1, Ø 3 x 12 cm). The product **5** was obtained as a dark-orange solid in 91% yield (301 mg, 346 µmol).

^{1}H NMR (400 MHz, CDCl$_3$, rt): δ [ppm]: 9.47 (d, J = 4.9 Hz, 2H), 8.70 (d, J = 4.9 Hz, 2H), 8.63 (d, J = 4.9 Hz, 2H), 8.53 (d, J = 4.9 Hz, 2H), 7.86 (d, J = 1.8 Hz, 2H), 7.70 (t, J = 1.9 Hz, 1H), 7.20 (s, 4H), 2.56 (s, 6H), 1.80 (s, 12H), 1.45 (s, 18H).

^{13}C{^{1}H} NMR (101 MHz, CDCl$_3$, rt): δ [ppm]: 148.95, 143.42, 142.88, 142.67, 142.36, 139.68, 138.95, 137.80, 136.91, 133.34, 133.20, 132.17, 131.42, 128.80, 127.77, 121.13, 120.61, 117.81, 101.68, 35.00, 31.65, 21.38, 21.34.

UV/Vis (CH$_2$Cl$_2$): *λ* [nm] (*ε* [M^{-1}cm^{-1}]): 417 (180000), 531 (13000).

HRMS (MALDI, DCTB) for $C_{52}H_{51}BrN_4Ni$ (M$^+$), calcd.: 868.2645, found: 868.2624.

TLC: R$_f$ [%]: 0.80 (hexanes/CH$_2$Cl$_2$ 3:1).

Fused Nickel-5,15-Dimesityl-10-(3,5-di-*tert*-butylphenyl)-20-Boronic-Ester-Porphyrin PhBpin

A 20 mL vial was filled with a solution of Nickel-5,15-Dimesityl-10-(3,5-di-*tert*-butylphenyl)-20-bromoporphyrin **5** (100 mg, 115 µmol, 1 equiv) in CH_2Cl_2 (20 mL) and cooled with an ice bath. The solution was degassed (bubbling N_2 through the solution for 15 min). The N_2 flow through the solution was increased, and a solution of dry $FeCl_3$ (149 mg, 920 µmol, 8 equiv) in CH_3NO_2 (0.5 mL) was added. The N_2 bubbling through the solution was stopped 15 min after $FeCl_3$ was added, and the solution was stirred under slow warming to rt for 24 h. MeOH (10 mL) was added to quench the reaction. After adding NEt_3 (1 mL), the solvent was removed, and the crude was purified by filtration through silica (SiO_2, hexanes/ CH_2Cl_2 - 1:1, Ø 3 x 12 cm). The obtained dark-green solid was used in the next step without further purification. The porphyrin mixture **6** from the previous step, $PdCl_2(PPh_3)_2$ (4.2 mg, 5.9 µmol, 0.05 equiv) and NEt_3 (0.4 mL) were dissolved in dry 1,2 dichloroethane (10 mL) in a 20 mL microwave vial. The vial was sealed, and the reaction mixture was degassed before pinacolborane (142 µL, 983 µmol, 8.33 equiv) was added via a syringe. The reaction mixture was stirred for 18 h at 90 °C under the exclusion of light. The solvent was removed under reduced pressure, and the crude product was subjected to column chromatography (SiO_2, hexanes/CH_2Cl_2 - 4:1 → 2:1, Ø 7 x 25 cm, 3rd band). **PhBpin** was obtained in 55% yield over the two reaction steps (58 mg, 63 µmol).

^{1}H NMR (400 MHz, CD_2Cl_2, rt): δ [ppm]: 9.27 (d, *J* = 4.9 Hz, 1H), 9.21 (d, *J* = 4.9 Hz, 1H), 9.12 (d, *J* = 5.0 Hz, 1H), 8.45 (d, *J* = 4.9 Hz, 1H), 8.26 (d, *J* = 4.9 Hz, 2H), 7.97 (d, *J* = 1.7 Hz, 1H), 7.68 (s, 1H), 7.22 (d, *J* = 4.4 Hz, 4H), 7.04 (d, *J* = 1.6 Hz, 1H), 2.57 (s, 6H), 1.92 (s, 6H), 1.82 (s, 6H), 1.68 (s, 12H), 1.56 (s, 9H), 1.47 (s, 9H).

^{13}C{^{1}H} NMR (151 MHz, CD_2Cl_2, rt): δ [ppm]: 153.88, 153.02, 150.91, 148.84, 147.77, 147.65, 147.17, 146.54, 145.26, 144.71, 144.17, 140.40, 139.16, 138.94, 138.27, 138.18, 137.42, 135.91, 134.52, 133.74, 133.46, 132.32, 131.38, 130.59, 128.12, 127.39, 126.27, 123.45, 121.50, 121.15, 117.85, 113.96, 85.51, 35.51, 35.32, 31.17, 29.01, 25.30, 21.53, 21.49, 21.46, 21.33.

UV/Vis (CH_2Cl_2): λ [nm] (ε [$M^{-1}cm^{-1}$]): 377 (39000),437 (93000), 572 (9000), 620 (6000)

HRMS (MALDI, DCTB) for $C_{58}H_{61}BN_4NiO_2$ (M^+), calcd.: 914.4236, found: 914.4235.

TLC: R_f [%]: 0.40 (hexanes/CH_2Cl_2 2:1).

2.2 Synthesis of Pyrene-Fused Porphyrin-Precursor

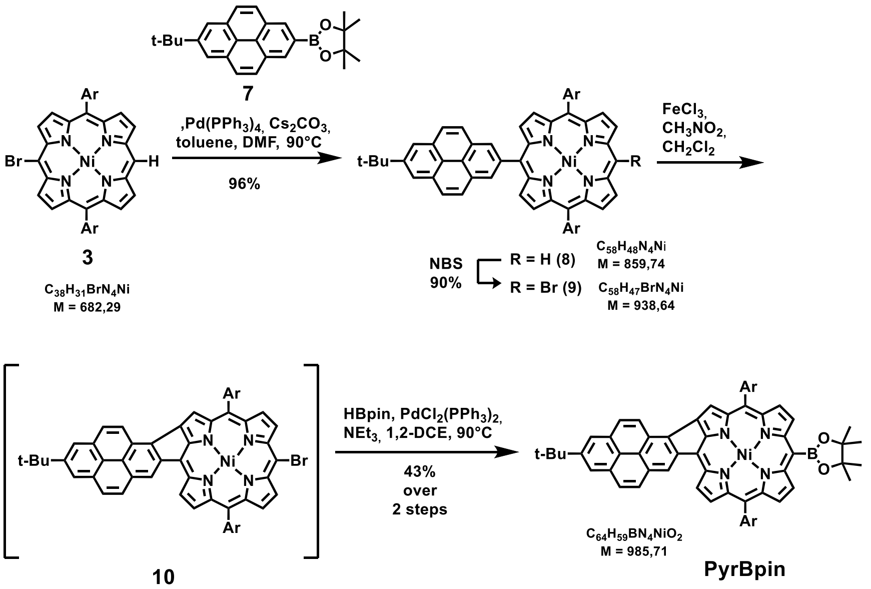

Scheme S2. Synthesis of pyrene-fused porphyrin building block **PyrBpin**. Ar = mesityl.

Nickel-5,15-Dimesityl-10-(2-*tert*-butyl-Pyrene)-Porphyrin 8

Nickel-5,15-Dimesityl-10-Bromoporphyrin **3** (160 mg, 235 µmol, 1 equiv), 2-(7-(*tert*-butyl)pyren-2-yl)-4,4,5,5-tetramethyl-1,3,2-dioxaborolane **7*** (108 mg, 281 µmol, 1.2 equiv), Cs_2CO_3 (229 mg, 704 µmol, 3 equiv) and $Pd(PPh_3)_4$ (54 mg, 47 µmol, 0.2 equiv) were dissolved in toluene (12 mL) and DMF (6 mL) and were degassed. The reaction mixture was heated to 90 °C for 18 h. After cooling to rt, the solvent was removed under reduced pressure, and the crude was subjected to silica plug filtration (hexanes/CH_2Cl_2 - 1:1, Ø 3 x 12 cm). The product **8** was obtained in 96% yield (194 mg, 226 µmol).

^{1}H NMR (400 MHz, $CDCl_3$, rt): δ [ppm]: 9.83 (s, 1H), 9.12 (d, *J* = 4.7 Hz, 2H), 8.85 (s, 2H), 8.76 (d, *J* = 4.8 Hz, 2H), 8.68 (d, *J* = 4.9 Hz, 2H), 8.64 (d, *J* = 4.9 Hz, 2H), 8.36 (s, 2H), 8.23 (d, *J* = 9.0 Hz, 2H), 8.17 (d, *J* = 9.0 Hz, 2H), 7.23 (s, 4H), 2.58 (s, 6H), 1.83 (s, 12H), 1.67 (s, 9H).

^{13}C{^{1}H} NMR (101 MHz, $CDCl_3$, rt): δ [ppm]: 149.43, 143.14, 142.98, 142.75, 142.73, 139.04, 138.58, 137.68, 137.33, 132.74, 132.44, 131.44, 131.21, 130.94, 130.04, 129.26, 128.62, 127.74, 127.39, 124.01, 122.94, 122.65, 119.16, 117.23, 104.40, 35.35, 32.00, 21.39, 21.38.

UV/Vis (CH_2Cl_2): λ [nm] (ε [$M^{-1}cm^{-1}$]): 324 (38000), 339 (36000), 410 (254000), 521 (23000).

HRMS (MALDI, DCTB) for $C_{58}H_{48}N_4Ni$ (M^+), calcd.: 858.3227, found: 858.3236.

TLC: R_f [%]: 0.70 (hexanes/CH_2Cl_2 3:1).

*Synthesized according to [3]

Nickel-5,15-Dimesityl-10-(2-*tert*-butyl-Pyrene)-20-Bromoporphyrin 9

To a solution of $CHCl_3$ (20 mL), pyridine (300 µL) and Nickel-5,15-Dimesityl-10-(2-*tert*-butyl-pyrene)-porphyrin **8** (192 mg, 223 µmol, 1 equiv) NBS (43 mg, 223 µmol, 1 equiv) in $CHCl_3$ (4.5 mL) was added slowly at rt. The mixture was stirred for 15 min at rt before the reaction was quenched with acetone (5 mL). The solvents were removed under reduced pressure, and the crude was purified by silica plug filtration (hexanes/CH_2Cl_2 - 1:1, Ø 3 x 12 cm). The product **9** was obtained as a dark-orange solid in 90% yield (189 mg, 201 µmol)

^1H NMR (400 MHz, $CDCl_3$, rt): δ [ppm]: 9.50 (d, J = 4.9 Hz, 2H), 8.77 (s, 2H), 8.64 (d, J = 5.0 Hz, 2H), 8.57 (d, J = 4.9 Hz, 2H), 8.51 (d, J = 4.9 Hz, 2H), 8.33 (s, 2H), 8.22 (d, J = 9.0 Hz, 2H), 8.16 (d, J = 8.9 Hz, 2H), 7.19 (s, 4H), 2.54 (s, 6H), 1.80 (s, 12H), 1.63 (s, 9H).

$^{13}C\{^1H\}$ NMR (101 MHz, $CDCl_3$, rt): δ [ppm]: 143.75, 143.15, 142.77, 142.51, 138.95, 138.00, 137.85, 136.86, 133.46, 133.19, 132.23, 131.59, 131.20, 129.89, 129.36, 128.70, 127.79, 127.33, 122.72, 118.13, 32.00, 21.38, 21.33.

UV/Vis (CH_2Cl_2): λ [nm] (ε [$M^{-1}cm^{-1}$]): 324 (34000), 339 (35000), 420 (281000), 531 (22000).

HRMS (MALDI, DCTB) for $C_{58}H_{47}BrN_4Ni$ (M^+), calcd.: 936.2332, found:936.2340.

TLC: R_f [%]: 0.75 (hexanes/CH_2Cl_2 3:1).

Fused Nickel-5,15-Dimesityl-10-(2-*tert*-butyl-Pyrene)-20-Boronic-Ester-Porphyrin PyrBpin

A 20 mL vial was filled with a solution of Nickel-5,15-Dimesityl-10-(2-*tert*-butyl-pyrene)-20-Bromoporphyrin **9** (40 mg, 43 µmol, 1 equiv) in CH_2Cl_2 (15 mL) and cooled with an ice bath. The solution was degassed (bubbling N_2 through the solution for 15 min). The N_2 flow through the solution was increased, and a solution of dry $FeCl_3$ (55 mg, 341 µmol, 8 equiv) in CH_3NO_2 (0.5 mL) was added. The N_2 bubbling through the solution was stopped 15 min after $FeCl_3$ was added, and the solution was stirred under slow warming to rt for 1 h. MeOH (10 mL) was added to quench the reaction. After adding NEt_3 (1 mL), the solvent was removed, and the crude was purified by filtration through silica (SiO_2, hexanes/ CH_2Cl_2 - 1:1, Ø 3 x 12 cm). The obtained dark-brown solid was used in the next step without further purification. The porphyrin mixture **10** from the previous step, $PdCl_2(PPh_3)_2$ (1.5 mg, 2.2 µmol, 0.05 equiv) and NEt_3 (0.2 mL) were dissolved in dry 1,2 dichloroethane (5 mL) in a 20 mL microwave vial. The vial was sealed, and the reaction mixture was degassed before pinacolborane (52 µL, 358 µmol, 8.33 equiv) was added via a syringe. The reaction mixture was stirred for 4 h at 90 °C under the exclusion of light. The solvent was removed under reduced pressure, and the crude product was subjected to column chromatography (SiO_2, hexanes/CH_2Cl_2 - 4:1 → 2:1, Ø 7 x 25 cm). **PyrBpin** was obtained in 43% yield over the two reaction steps (18 mg, 18 µmol).

^{1}H NMR (601 MHz, CD_2Cl_2, rt): δ [ppm]: 9.31 - 9.30 (m, 1H), 9.25 - 9.23 (m, 1H), 9.19 - 9.18 (m, 1H), 8.65 - 8.63 (m, 1H), 8.49 (d, *J* = 4.8 Hz, 1H), 8.28 - 8.26 (m, 2H), 8.11 - 8.09 (m, 1H), 8.05 (m, 1H), 8.02 (m, 1H), 7.98 - 7.95 (m, 1H), 7.92 - 7.89 (m, 2H), 7.88 - 7.85 (m, 1H), 7.27 (s, 2H), 7.24 (s, 2H), 2.61 (s, 3H), 2.58 (s, 3H), 1.97 (s, 6H), 1.84 (s, 6H), 1.69 (s, 9H), 1.55 (s, 12H).

^{13}C{^{1}H} NMR (151 MHz, CD_2Cl_2, rt): δ [ppm]: 155.66, 150.01, 147.90, 147.57, 147.48, 147.40, 145.73, 145.23, 144.64, 141.27, 139.15, 139.01, 138.35, 138.23, 137.37, 136.06, 134.62, 133.84, 133.06, 132.89, 132.34, 131.77, 131.52, 130.73, 129.44, 128.24, 128.19, 128.14, 127.73, 127.48, 126.53, 124.82, 124.62, 123.86, 123.59, 122.01, 121.87, 118.28, 114.01, 31.79, 30.04, 30.01, 25.28, 23.06, 21.53, 21.49, 21.34, 21.11, 14.24.

UV/Vis (CH_2Cl_2): λ [nm] (ε [$M^{-1}cm^{-1}$]): 338 (30000), 401 (47000), 474 (71000), 498 (74000), 586 (10000), 634 (7000).

HRMS (MALDI, DCTB) for $C_{64}H_{57}BN_4NiO_2$ (M^+), calcd.: 982.3923, found: 982.3935.

TLC: R_f [%]: 0.25 (hexanes/CH_2Cl_2 3:1).

2.3 Synthesis of HBC-Fused Porphyrin-Precursor

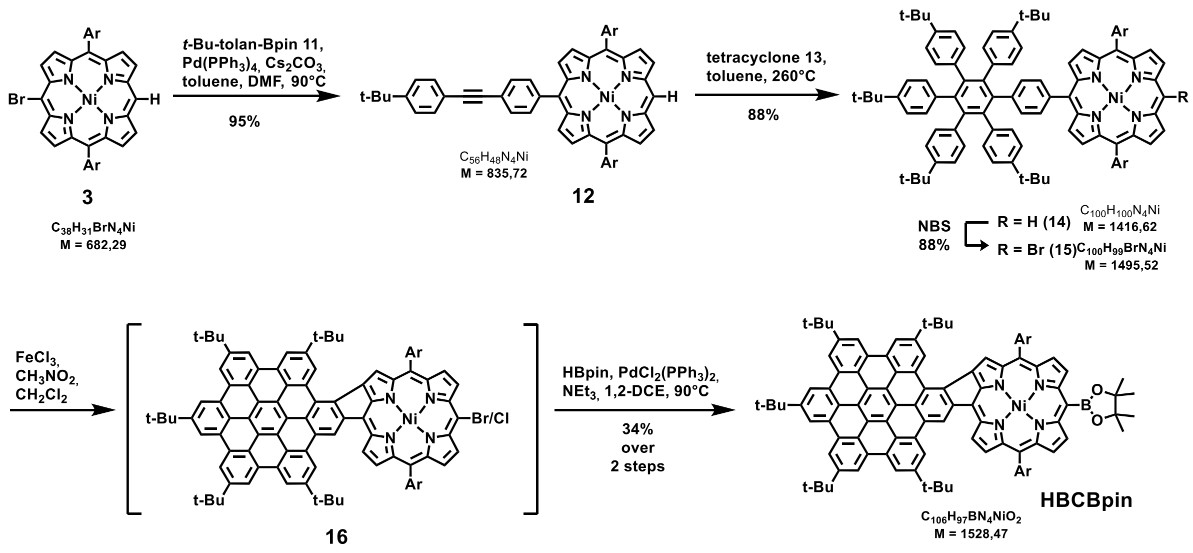

Scheme S3. Synthesis of HBC-fused porphyrin building block **HBCBpin**. Ar = mesityl.

Nickel-5,15-Dimesityl-10-(*tert*-butyl-Tolane)-Porphyrin 12

Nickel-5,15-Dimesityl-10-Bromoporphyrin **3** (277 mg, 406 µmol, 1 equiv), 2-(4-((4-(*tert*-butyl)phenyl)ethynyl)phenyl)-4,4,5,5-tetramethyl-1,3,2-dioxaborolane **11*** (175 mg, 487 µmol, 1.2 equiv), Cs_2CO_3 (398 mg, 1.22 mmol, 3 equiv) and $Pd(PPh_3)_4$ (94 mg, 81 µmol, 0.2 equiv) were dissolved in toluene (24 mL) and DMF (12 mL) and were degassed. The reaction mixture was heated to 90 °C for 18 h. After cooling to rt, the solvent was removed under reduced pressure, and the crude was subjected to silica plug filtration (hexanes/CH_2Cl_2 - 1:1, Ø 3 x 12 cm). The product **12** was obtained in 95% yield (322 mg, 386 µmol).

^{1}H NMR (400 MHz, $CDCl_3$, rt): δ [ppm]: 9.80 (s, 1H), 9.10 (d, *J* = 4.7 Hz, 2H), 8.76 - 8.72 (m, 4H), 8.66 (d, *J* = 4.9 Hz, 2H), 8.06 - 8.02 (m, 2H), 7.86 - 7.82 (m, 2H), 7.61 - 7.57 (m, 2H), 7.45 - 7.43 (m, 2H), 7.23 (s, 4H), 2.59 (s, 6H), 1.80 (s, 12H), 1.36 (s, 9H).

$^{13}C\{^{1}H\}$ NMR (101 MHz, $CDCl_3$, rt): δ [ppm]: 151.73, 142.97, 142.71, 142.30, 141.20, 139.04, 137.72, 137.27, 133.71, 133.04, 132.49, 132.11, 131.47, 131.22, 131.04, 129.96, 127.76, 125.45, 125.32, 122.92, 120.24, 118.19, 117.18, 116.37, 104.42, 90.57, 88.70, 31.22, 31.18, 21.41, 21.35.

UV/Vis (CH_2Cl_2): *λ* [nm] (*ε* [$M^{-1}cm^{-1}$]): 291 (44000), 408 (235000), 521 (17000).

HRMS (MALDI, DCTB) for $C_{56}H_{48}N_4Ni$ (M^+), calcd.: 834.3227, found:834.3244.

TLC: R_f [%]: 0.70 (hexanes/CH_2Cl_2 2:1).

*Synthesized according to [4]

Nickel-5-15-Dimesityl-10-HAB-Porphyrin 14

A pressure tube was charged with tetracyclone **13*** (874 mg, 1.44 mmol, 4 equiv), **12** (300 mg, 359 µmol, 1 equiv) and Ph_2O (2 mL). The mixture was heated for 28 h at 220 °C. After removal of the solvent, the residue was dissolved in CH_2Cl_2 (10 mL), and the product was precipitated via the addition of MeOH (50 mL). The precipitate was filtered off and dried *in vacuo*. **11** was obtained as a red-brown solid in 88% yield (448 mg, 316 µmol).

^{1}H NMR (400 MHz, $CDCl_3$, rt): δ [ppm]: 9.74 (s, 1H), 9.05 (d, *J* = 4.7 Hz, 2H), 8.66 (d, *J* = 4.7 Hz, 2H), 8.48 (d, *J* = 4.9 Hz, 2H), 8.37 (d, *J* = 4.9 Hz, 2H), 7.50 - 7.46 (m, 2H), 7.21 (s, 4H), 7.11 - 7.03 (m, 6H), 6.96 - 6.92 (m, 4H), 6.90 - 6.83 (m, 6H), 6.82 - 6.77 (m, 6H), 2.59 (s, 6H), 1.74 (s, 12H), 1.22 (s, 18H), 1.13 (s, 18H), 1.12 (s, 9H).

$^{13}C\{^{1}H\}$ NMR (101 MHz, $CDCl_3$, rt): δ [ppm]: 148.01, 147.55, 147.50, 142.76, 142.54, 142.50, 142.43, 141.05, 140.77, 140.52, 140.32, 140.15, 139.03, 138.08, 137.90, 137.85, 137.59, 137.45, 137.26, 132.43, 132.22, 131.70, 131.41, 131.28, 131.16, 131.13, 130.64, 129.75, 129.64, 129.07, 127.64, 124.89, 124.55, 123.40, 123.15, 123.13, 119.14, 116.84, 34.25, 34.10, 34.08, 31.35, 31.22, 21.42, 21.26.

UV/Vis (CH_2Cl_2): *λ* [nm] (*ε* [$M^{-1}cm^{-1}$]): 408 (200000), 521 (17000).

HRMS (MALDI, DCTB) for $C_{100}H_{100}N_4Ni$ (M^+), calcd.: 1414.7296, found: 1414.7270.

TLC: R_f [%]: 0.60 (hexanes/CH_2Cl_2 2:1).

*Synthesized according to [5]

Nickel-5-15-Dimesityl-10-HAB-20-Bromoporphyrin 15

To a solution of $CHCl_3$ (30 mL), pyridine (720 µL) and Nickel-5-15-Dimesityl-10-HAB-porphyrin **14** (475 mg, 335 µmol) NBS (60 mg, 335 µmol) in $CHCl_3$ (5 mL) was added slowly at rt. The mixture was stirred for 15 min at rt before the reaction was quenched with acetone (5 mL). The solvents were removed under reduced pressure, and the crude was purified by silica plug filtration (hexanes/CH_2Cl_2 - 1:1, Ø 3 x 12 cm). The product **15** was obtained as a dark-orange solid in 88% yield (441 mg, 295 µmol)

^{1}H NMR (400 MHz, $CDCl_3$, rt): δ [ppm]: 9.43 (d, *J* = 5.0 Hz, 2H), 8.56 (d, *J* = 4.9 Hz, 2H), 8.38 (d, *J* = 4.9 Hz, 2H), 8.28 (d, *J* = 4.9 Hz, 2H), 7.45 - 7.40 (m, 2H), 7.21 - 7.17 (m, 4H), 7.10 - 7.06 (m, 2H), 7.06 - 7.02 (m, 4H), 6.95 - 6.91 (m, 4H), 6.89 - 6.84 (m, 6H), 6.80 - 6.74 (m, 6H), 2.57 (s, 6H), 1.75 (s, 12H), 1.21 (s, 18H), 1.13 (s, 18H), 1.12 (s, 9H).

^{13}C{^{1}H} NMR (101 MHz, $CDCl_3$, rt): δ [ppm]: 148.00, 147.58, 147.52, 143.15, 142.85, 142.50, 142.26, 141.22, 140.81, 140.53, 140.28, 140.05, 138.93, 138.06, 137.87, 137.81, 137.77, 136.87, 136.77, 133.26, 132.89, 132.08, 131.57, 131.39, 131.30, 131.15, 131.11, 129.88, 127.69, 123.39, 123.16, 123.13, 119.33, 117.75, 34.25, 34.10, 34.08, 31.34, 31.22, 21.41, 21.23.

UV/Vis (CH_2Cl_2): λ [nm] (ε [$M^{-1}cm^{-1}$]): 417 (256000), 531 (22000).

HRMS (MALDI, DCTB) for $C_{100}H_{99}BrN_4Ni$ (M^+), calcd.: 1492.6401, found: 1492.6404.

TLC: R_f [%]: 0.60 (hexanes/CH_2Cl_2 2:1).

Fused Nickel-5-15-Dimesityl-10-HBC-20-Boronic-Ester-Porphyrin HBCBpin

A 20 mL vial was filled with a solution of Nickel-5-15-Dimesityl-10-HAB-20-bromoporphyrin **15** (100 mg, 67 µmol, 1 equiv) in CH_2Cl_2 (20 mL) and cooled with an ice bath. The solution was degassed (bubbling N_2 through the solution for 15 min). The

N_2 flow through the solution was increased, and a solution of dry $FeCl_3$ (325 mg, 2.01 mmol, 30 equiv) in CH_3NO_2 (1 mL) was added. The N_2 bubbling through the solution was stopped 15 min after $FeCl_3$ was added, and the solution was stirred under slow warming to rt for 48 h. MeOH (10 mL) was added to quench the reaction. After adding NEt_3 (1 mL), the solvent was removed, and the crude was purified by filtration through silica (SiO_2, hexanes/ CH_2Cl_2 - 1:1, Ø 3 x 12 cm). The obtained dark-brown solid was used in the next step without further purification. The porphyrin mixture **16** from the previous step, $PdCl_2(PPh_3)_2$ (2.2 mg, 3.2 µmol, 0.05 equiv.) and NEt_3 (0.4 mL) were dissolved in dry 1,2 dichloroethane (10 mL) in a 20 mL microwave vial. The vial was sealed, and the reaction mixture was degassed before pinacolborane (77 µL, 529 µmol, 8.33 equiv) was added via a syringe. The reaction mixture was stirred for 18 h at 90 °C under the exclusion of light. The solvent was removed under reduced pressure, and the crude product was subjected to column chromatography (SiO_2, hexanes/CH_2Cl_2 - 4:1 → 2:1, Ø 7 x 35 cm). **HBCBpin** was obtained in 34% yield over the two reaction steps (35 mg, 23 µmol).

^{1}H NMR (601 MHz, CD_2Cl_2, rt): δ [ppm]: 9.84 - 9.76 (m, 3H), 9.61 (d, *J* = 4.9 Hz, 1H), 9.41 (s, 1H), 9.36 - 9.33 (m, 5H), 9.29 (d, *J* = 4.8 Hz, 1H), 9.28 - 9.27 (m, 1H), 9.25 - 9.22 (m, 2H), 8.65 (d, *J* = 4.8 Hz, 1H), 8.42 (s, 1H), 8.34 - 8.33 (m, 2H), 7.29 (s, 2H), 7.24 (s, 2H), 2.62 (s, 3H), 2.60 (s, 3H), 1.96 (s, 6H), 1.92 - 1.90 (m, 15H), 1.84 (s, 18H), 1.71 (s, 9H), 1.58 (s, 9H).

^{13}C{^{1}H} NMR (151 MHz, CD_2Cl_2, rt): δ [ppm]: 155.50, 149.96, 149.85, 149.56, 149.25, 149.23, 148.26, 147.87, 146.87, 145.72, 145.12, 144.70, 140.94, 139.20, 138.76, 138.40, 138.35, 137.41, 135.93, 134.72, 134.09, 133.92, 132.66, 131.70, 131.50, 131.06, 131.05, 130.93, 130.85, 130.83, 130.73, 130.64, 130.45, 130.42, 128.66, 128.52, 128.18, 127.35, 125.09, 124.18, 124.12, 124.05, 123.89, 123.75, 123.05, 122.36, 121.70, 121.29, 121.24, 121.21, 121.13, 120.98, 120.68, 119.74, 119.67, 119.64, 119.59, 119.56, 119.47, 119.43, 119.37, 118.53, 113.71, 54.16, 36.05, 32.35, 32.06, 30.05, 25.31, 21.60, 21.39.

UV/Vis (CH_2Cl_2): *λ* [nm] (*ε* [$M^{-1}cm^{-1}$]): 379 (84000), 437 (64000), 509 (83000), 594 (14000), 641 (11000).

HRMS (MALDI, DCTB) for $C_{106}H_{97}BN_4NiO_2$ (M^+), calcd.: 1526.7053, found: 1562.7027.

TLC: R_f [%]: 0.40 (hexanes/CH_2Cl_2 3:1).

2.4 Synthesis of Naphthalene- and Perylenediimides

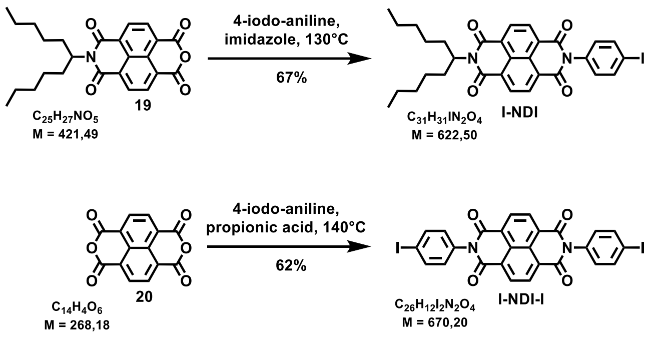

Scheme S4. Synthesis of halogenated naphthalenediimides.

Iodo-Phenyl-Naphthalenediimide I-NDI

To a 20 mL microwave vial, naphthalenemonoimide **19*** (100 mg, 237 µmol), 4-iodo-aniline (78 mg, 356 µmol, 1.5 equiv) and imidazole (1.00 g) were added, and the vial was sealed and evacuated and refilled with nitrogen three times. Then, the reaction mixture was heated to 130 °C. When the imidazole was fully molten, the reaction mixture was stirred at 130 °C for 2 h. Following, aqueous HCl (2M, 15 mL) was added, and the reaction was stirred at room temperature overnight. Then, the formed precipitate was collected *via* filtration, and the solid residue was washed with H_2O until neutrality and dried *in vacuo*, yielding the desired **I-NDI** as a beige solid (99 mg, 159 µmol, 67%).

^{1}H NMR (400 MHz, $CDCl_3$, rt): δ [ppm]: 8.85 - 8.72 (m, 4H), 7.95 - 7.86 (m, 2H), 7.12 - 7.02 (m, 2H), 5.22 - 5.12 (m, 1H), 2.28 - 2.15 (m, 2H), 1.93 - 1.80 (m, 2H), 1.37 - 1.17 (m, 12H), 0.84 (t, *J* = 6.8 Hz, 6H).

^{13}C{^{1}H} NMR (101 MHz, $CDCl_3$, rt): δ [ppm]: 163.01, 138.93, 134.48, 131.64, 130.59, 127.16, 126.37, 95.12, 55.50, 32.36, 31.79, 26.71, 22.66, 14.14.

UV/Vis (CH_2Cl_2): λ [nm] (ε [$M^{-1}cm^{-1}$]): 343 (14000), 360 (26000), 381 (27000).

HRMS (APPI) for $C_{31}H_{32}IN_2O_4$ $[M+H]^+$, calcd.: 623.1401, found: 623.1433.

*Synthesized according to [6]

Bis-Iodo-Phenyl-Naphthalenediimide I-NDI-I

Adapting a procedure from Spitler *et al.*,[7] in a 20 mL microwave vial, naphthalenetetracarboxylic dianhydride **20** (268 mg, 1.00 mmol, 1 equiv) and 4-iodo-aniline (948 mg, 4.32 mmol, 4.32 equiv) were dissolved in propionic acid (16 mL) under nitrogen atmosphere and stirred for 48 h at 140 °C. After cooling to room temperature, the then-formed precipitate was collected by filtration, and the crude product was washed with MeOH (3 x 10 mL) and hexane (20 mL) and dried *in vacuo*, yielding the desired **I-NDI-I** as a beige powder (423 mg, 623 µmol, 62%).

^{1}H NMR (400 MHz, DMSO-d6, rt): δ [ppm]: 8.72 (s, 4H), 7.96 - 7.91 (m, 4H), 7.33 - 7.26 (m, 4H).

^{13}C{^{1}H} NMR (101 MHz, $CDCl_3$, rt): δ [ppm]: *The poor solubility of the compound precluded the acquisition of a ^{13}C-NMR spectrum*.

UV/Vis (CH_2Cl_2): λ [nm] (ε [$M^{-1}cm^{-1}$]): 343 (16000), 359 (26000), 380 (29000).

HRMS (APPI) for $C_{26}H_{13}I_2N_2O_4$ [M+H]$^+$, calcd.: 670.8959, found: 670.8969.

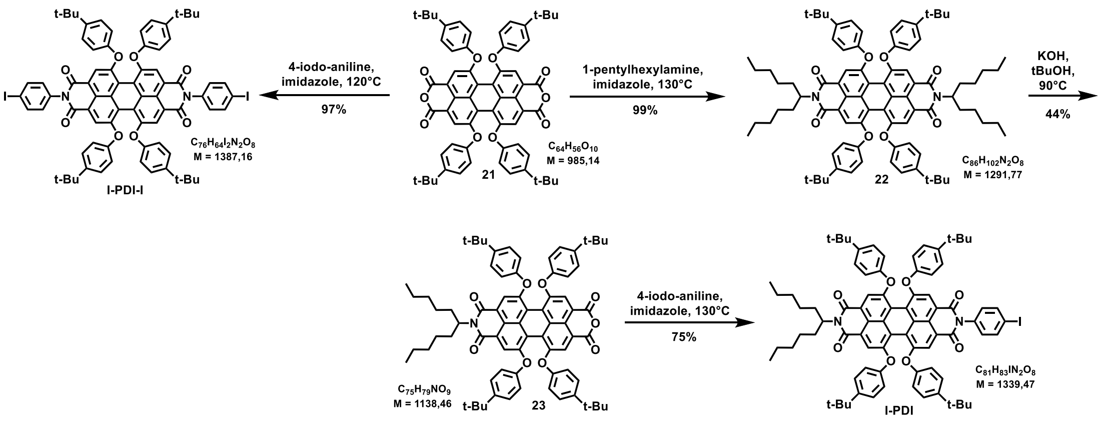

Scheme S5. Synthesis of halogenated perylenediimides.

Tetra-(*tert*-butyl-phenoxy)perylenediimide 22

In a 20 mL microwave vial, perylenebisanhydride **21*** (100 mg, 101 µmol, 1 equiv), 1-pentylhexylamine (86.5 mg, 505 µmol, 5 equiv) and Imidazole (1.00 g) were added and the vial was evacuated and refilled with nitrogen three times. Then, the reaction mixture was headed to 130 °C and was stirred once the imidazole was molten for 4 h. Subsequently, aqueous HCl (1M, 10 mL) was added, and the reaction was stirred at room temperature overnight. The formed precipitate was collected by filtration and washed with water until neutrality. Drying *in vacuo* yielded the desired perylenediimide **22** as a red solid (130 mg, 100 µmol, 99%).

^{1}H NMR (400 MHz, $CDCl_3$, rt): δ [ppm]: 8.21 (d, *J* = 10.9 Hz, 4H), 7.25 - 7.20 (m, 8H), 6.90 - 6.79 (m, 8H), 5.13 - 5.03 (m, 2H), 2.21 - 2.04 (m, 4H), 1.85 - 1.70 (m, 4H), 1.29 (s, 36H), 1.27 - 1.13 (m, 24H), 0.85 - 0.73 (m, 12H).

^{13}C{^{1}H} NMR (101 MHz, $CDCl_3$, rt): δ [ppm]: 156.08, 152.96, 147.34, 133.01, 126.79, 122.48, 120.50, 120.36, 120.32, 119.86, 54.87, 34.51, 32.56, 31.88, 31.62, 26.77, 22.69, 14.18.

HRMS (APPI) for $C_{86}H_{102}N_2O_8$ $[M+H]^+$, calcd.: 1291.7709, found: 1291.7716.

*Synthesized according to [8]

Tetra-(*tert*-butyl-phenoxy)perylenemonoimide 23

In a 100 mL round-bottom flask equipped with a condenser, perylenediimide **22** (124 g, 96 µmol, 1 equiv) and KOH (16.2 mg, 288 µmol, 3 equiv) were dissolved in tBuOH (15 mL). The reaction mixture was stirred at 90 °C for 3 h. Following, the mixture was allowed to cool to room temperature, and subsequently, AcOH (3 mL) and aqueous HCl (1M, 15 mL) were added, and the mixture was stirred overnight. The formed precipitate was collected via filtration and washed with H_2O until neutrality. The obtained solid was further purified by silica gel plug filtration (SiO_2, hexanes/CH_2Cl_2 - 1:1 → 1:2), eluting the desired product as the second fraction. Evaporation of the solvents afforded **23** as a red powder (48 mg, 42 µmol, 44%)

^{1}H NMR (400 MHz, $CDCl_3$, rt): δ [ppm]: 8.25 - 8.18 (m, 4H), 7.25 - 7.22 (m, 8H), 6.88 - 6.77 (m, 8H), 5.13 - 5.00 (m, 1H), 2.20 - 2.04 (m, 2H), 1.84 - 1.72 (m, 2H), 1.29 (s, 36H), 1.26 - 1.15 (m, 12H), 0.86 - 0.76 (m, 6H).

$^{13}C\{^1H\}$ NMR (101 MHz, $CDCl_3$, rt): δ [ppm]: 160.21, 156.81, 155.90, 152.78, 152.64, 147.85, 147.70, 133.31, 133.28, 126.96, 126.90, 122.46, 121.92, 121.74, 119.59, 119.44, 118.04, 77.48, 77.16, 76.84, 55.01, 34.55, 31.86, 31.60, 31.58, 26.77, 22.68, 14.17.

HRMS (APPI) for $C_{75}H_{79}NO_9$ $[M+H]^+$, calcd.: 1138.5828, found: 1138.5814.

Tetra-(*tert*-butyl-phenoxy)-Iodo-Phenyl-Perylenebisimide I-PDI

To a 20 mL sealable vial, perylenemonoimide **23** (45 mg, 40 µmol, 1 equiv), 4-iodo-aniline (13 mg, 59 µmol, 1.5 eq.) and imidazole (500 mg) were added, and the reaction vessel was evacuated and refilled with nitrogen three times. After that, the reaction mixture was heated to 130 °C, until the imidazole was molten, and then the reaction mixture was stirred at that temperature for 3 h. Following, aqueous HCl (1M, 15 mL) was added, and the reaction was stirred at room temperature overnight. Then, the red precipitate was collected via filtration and washed with more aqueous HCl (1M, 10 mL) and subsequently with H_2O until neutrality. The crude product was further purified by a silica plug filtration (SiO_2, hexanes/CH_2Cl_2 - 1:1), yielding the perylenediimide **I-PDI** as a dark purple solid (40 mg, 29.9 µmol, 75%)

^{1}H NMR (400 MHz, $CDCl_3$, rt): δ [ppm]: 8.17 (s, 1H), 8.13 - 8.04 (m, 1H), 7.87 - 7.81 (m, 2H), 7.31 - 7.22 (m, 8H), 7.06 - 6.99 (m, 2H), 6.88 - 6.80 (m, 8H), 5.09 - 5.02 (m, 1H), 2.19 - 2.07 (m, 2H), 1.81 - 1.68 (m, 2H), 1.30 (s, 18H), 1.28 (s, 18H), 1.27 - 1.19 (m, 12H), 0.84 - 0.77 (m, 6H).

$^{13}C\{^1H\}$ NMR (101 MHz, $CDCl_3$, rt): δ [ppm]: 163.51, 156.38, 155.99, 153.00, 147.52, 147.48, 138.60, 135.12, 133.30, 132.96, 130.70, 126.85, 126.80, 122.18, 121.35, 120.46, 119.98, 119.74, 119.52, 119.45, 94.46, 54.93, 34.52, 34.50, 32.56, 31.88, 31.61, 31.56, 26.78, 22.69, 14.18.

UV/Vis (CH_2Cl_2): λ [nm] (ε [$M^{-1}cm^{-1}$]): 452 (15000), 542 (27000), 581 (44000).

HRMS (APPI) for $C_{81}H_{84}IN_2O_8$ $[M+H]^+$, calcd.: 1339.5267, found: 1339.5282.

Tetra-(*tert*-butyl-phenoxy)-Bis-Iodo-Phenyl-Perylenediimide I-PDI-I

Adapting a procedure from Schlosser *et al.*,[9] to a 10 mL sealable vial, perylenebisanhydride **21** (40 mg, 41 µmol, 1 equiv), 4-iodo-aniline (177 mg, 8.12 mmol, 20 equiv), and imidazole (500 mg) were added, and the vessel was sealed and evacuated and refilled with nitrogen three times. Then, the reaction mixture was heated to 120 °C until the imidazole was molten and stirred at that temperature for 5 h. After that, aqueous HCl (2M, 7 mL) was added, and the mixture was stirred overnight at room temperature. Then, the formed precipitate was collected by filtration, and the solid residue was washed until neutrality. The crude product was then redissolved in DCM (50 mL), washed with aqueous HCl (2M, 2 x 100 mL) and H_2O (2 x 100 mL), and dried over $MgSO_4$. Removal of the solvent under reduced pressure gave the desired **I-PDI-I** as a dark purple powder (55 mg, 39.6 µmol, 97%)

^{1}H NMR (400 MHz, $CDCl_3$, rt): δ [ppm]: 8.23 (s, 4H), 7.84 - 7.80 (m, 4H), 7.25 - 7.20 (m, 8H), 7.02 - 6.97 (m, 4H), 6.86 - 6.82 (m, 8H), 1.27 (s, 36H).

^{13}C{^{1}H} NMR (101 MHz, $CDCl_3$, rt): δ [ppm]: 163.46, 156.28, 152.88, 147.66, 138.62, 135.07, 133.26, 130.68, 126.86, 122.48, 120.98, 120.38, 119.86, 119.46, 94.52, 34.52, 31.56.

UV/Vis (CH_2Cl_2): *λ* [nm] (*ε* [$M^{-1}cm^{-1}$]): 454 (18000), 543 (31000), 583 (51000).

HRMS (MALDI, DCTB) for $C_{76}H_{64}I_2N_2O_8$ $[M+H]^+$, calcd.: 1386.2752, found: 1386.2935.

2.5 Synthesis of Donor-Acceptor Dyads

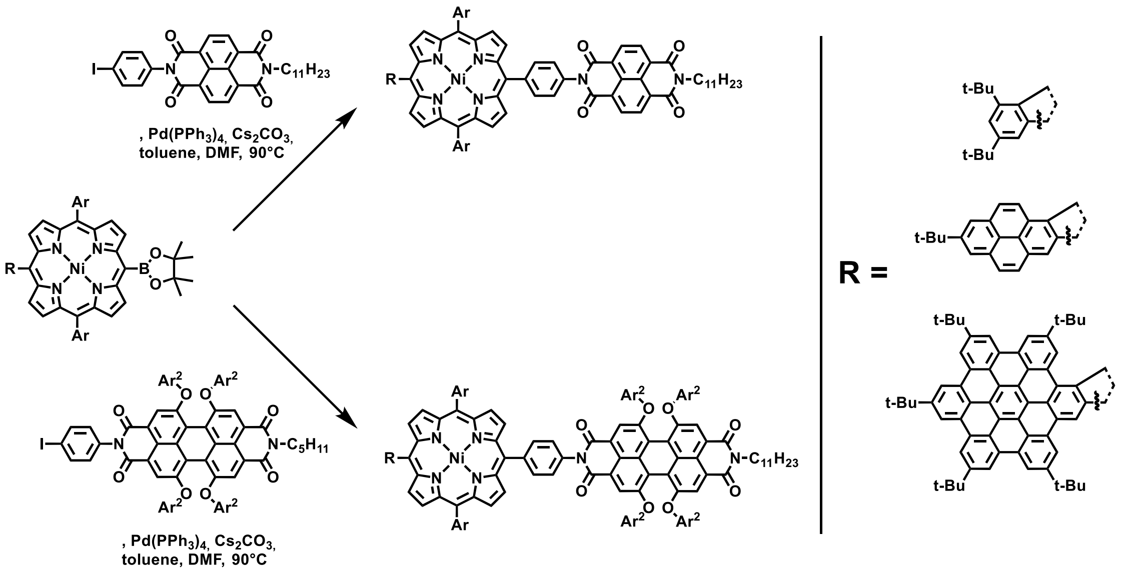

Scheme S6. General scheme for the Synthesis of the D-A dyads. Ar = mesityl; Ar^2 = 4-*t*Bu-phenyl.

General Procedure for Synthesis of the D-A Dyads

Fused boronic-ester porphyrin, rylenediimide**,** Cs_2CO_3, and $Pd(PPh_3)_4$ were dissolved in toluene and DMF and were degassed under sonication. The reaction mixture was heated to 90 °C for 18 h. After cooling to rt, the solvent was removed under reduced pressure, and the crude was subjected to silica plug filtration (hexanes/CH_2Cl_2 - 1:1, Ø 3 x 12 cm) to remove the inorganics. The crude was further purified by size exclusion chromatography (Biobeads SX1, toluene, Ø 5 x 120 cm). After filtration through silica (hexanes/CH_2Cl_2 - 1:1, Ø 3 x 12 cm), the product was obtained as a dark solid.

Phenyl-Fused-Porphyrin-Naphthalenediimide Ph-NDI

Phenyl-fused boronic-ester porphyrin **PhBpin** (13 mg, 14 µmol, 1.05 equiv), 2-(4-iodophenyl)-7-undecylbenzo[lmn][3,8]phenanthroline-1,3,6,8(2H,7H)-tetraone **I-NDI** (8.3 mg, 13 µmol, 1 equiv), Cs_2CO_3 (13 mg, 40 µmol, 3 equiv), and $Pd(PPh_3)_4$ (3.1 mg, 2.7 µmol, 0.2 equiv) were dissolved in toluene (2 mL) and DMF (1 mL) and the **General Procedure for Synthesis of the D-A Dyads** was followed. **Ph-NDI** was obtained in 41% yield (6.9 mg, 5.4 µmol).

^{1}H NMR (601 MHz, CD_2Cl_2, rt): δ [ppm]: 9.12 - 9.10 (m, 1H), 8.88 (d, *J* = 7.5 Hz, 2H), 8.81 (s, 2H), 8.47 - 8.45 (m, 2H), 8.39 (d, *J* = 4.8 Hz, 1H), 8.21 (d, *J* = 4.9 Hz, 1H), 8.17 (d, *J* = 4.8 Hz, 1H), 8.16 - 8.12 (m, 2H), 7.97 (d, *J* = 1.9 Hz, 1H), 7.69 (s, 1H), 7.65 - 7.60 (m, 2H), 7.25 - 7.18 (m, 4H), 7.04 (d, *J* = 1.7 Hz, 1H), 2.56 (m, 6H), 1.93 (s, 6H), 1.84 (s, 6H), 1.61 (s, 9H), 1.47 (s, 9H), 1.36 - 0.81 (m, 23H).

^{13}C{^{1}H} NMR (151 MHz, CD_2Cl_2, rt): δ [ppm]: 163.63, 154.79, 153.17, 150.91, 148.66, 147.91, 146.55, 145.89, 144.85, 144.53, 143.65, 141.71, 141.21, 139.11, 138.88, 138.33, 138.23, 137.32, 135.78, 135.14, 134.35, 133.98, 133.37, 133.16, 131.59, 131.01, 130.80, 130.04, 128.15, 127.69, 127.48, 127.31, 126.96, 126.64, 123.29, 122.07, 121.33, 120.97, 118.26, 113.09, 35.52, 35.34, 32.60, 32.09, 31.17, 28.98, 26.94, 22.93, 21.51, 21.47, 21.33, 14.15.

UV/Vis (CH_2Cl_2): *λ* [nm] (*ε* [$M^{-1}cm^{-1}$]): 362 (43000), 381 (57000), 441 (92000), 571 (9000), 612 (5000).

HRMS (MALDI, DCTB) for $C_{83}H_{80}N_6NiO_4$ (M^+), calcd.: 1282.5589, found: 1282.5566.

TLC: R_f [%]: 0.40 (hexanes/CH_2Cl_2 1:1).

Phenyl-Fused-Porphyrin-Perylenediimide Ph-PDI

Phenyl-fused boronic-ester porphyrin **PhBpin** (10 mg, 11 µmol, 1.05 equiv), 5,6,12,13-tetrakis(4-(*tert*-butyl)phenoxy)-2-(4-iodophenyl)-9-undecylanthra[2,1,9-def:6,5,10-d'e'f']diisoquinoline-1,3,8,10(2H,9H)-tetraone **I-PDI** (14 mg, 13 µmol, 1 equiv), Cs_2CO_3 (10 mg, 31 µmol, 3 equiv) and $Pd(PPh_3)_4$ (2.4 mg, 2.1 µmol, 0.2 equiv) were dissolved in toluene (2 mL) and DMF (1 mL) and the **General Procedure for Synthesis of the D-A Dyads** was followed. **Ph-PDI** was obtained in 57% yield (12 mg, 5.9 µmol).

^{1}H NMR (601 MHz, CD_2Cl_2, rt): δ [ppm]: 9.10 (d, *J* = 5.1 Hz, 1H), 8.45 (d, *J* = 4.9 Hz, 1H), 8.40 (d, *J* = 4.9 Hz, 1H), 8.34 (d, *J* = 4.8 Hz, 1H), 8.30 (s, 2H), 8.16 (d, *J* = 4.9 Hz, 2H), 8.12 (d, *J* = 4.8 Hz, 2H), 8.08 - 8.05 (m, 2H), 7.97 (d, *J* = 1.6 Hz, 1H), 7.68 (s, 1H), 7.57 - 7.54 (m, 2H), 7.34 - 7.26 (m, 8H), 7.22 - 7.17 (m, 4H), 7.04 (d, *J* = 1.6 Hz, 1H), 6.94 - 6.85 (m, 8H), 2.55 (s, 6H), 1.91 (s, 6H), 1.81 (s, 6H), 1.56 (s, 9H), 1.47 (s, 9H), 1.32 (s, 18H), 1.30 (s, 18H), 1.28 - 0.81 (m, 23H).

$^{13}C\{^1H\}$ NMR (601 MHz, CD_2Cl_2, rt): δ [ppm]: 163.87, 156.54, 156.05, 154.73, 153.64, 153.12, 150.92, 148.60, 147.87, 147.81, 147.72, 146.49, 145.85, 144.82, 144.47, 143.68, 143.67, 141.27, 141.17, 139.09, 138.86, 138.29, 138.18, 137.32, 135.78, 135.65, 134.19, 133.93, 133.62, 133.37, 133.22, 133.16, 131.06, 130.73, 130.20, 129.98, 128.13, 128.12, 127.71, 127.25, 127.03, 126.60, 123.25, 122.95, 122.02, 121.64, 121.51, 120.94, 120.80, 120.41, 120.01, 119.74, 119.72, 119.45, 118.21, 113.01, 35.51, 35.33, 34.65, 34.63, 32.67, 32.12, 31.57, 31.56, 31.17, 30.05, 28.97, 27.53, 26.90, 22.94, 21.50, 21.45, 21.31.

UV/Vis (CH_2Cl_2): λ [nm] (ε [$M^{-1}cm^{-1}$]): 377 (37000), 441 (95000), 540 (34000), 580 (54000).

HRMS (MALDI, DCTB) for $C_{133}H_{132}N_6NiO_8$ (M^+), calcd.: 1998.9455, found: 1998.9482.

TLC: R_f [%]: 0.70 (hexanes/CH_2Cl_2 1:2).

Pyrene-Fused-Porphyrin-Naphthalenediimide Pyr-NDI

Pyrene-fused boronic-ester porphyrin **PyrBpin** (10 mg, 10 µmol, 1.05 equiv), 2-(4-iodophenyl)-7-undecylbenzo[lmn][3,8]phenanthroline-1,3,6,8(2H,7H)-tetraone **I-NDI** (6.0 mg, 9.7 µmol, 1 equiv), Cs_2CO_3 (9.5 mg, 29 µmol, 3 equiv) and $Pd(PPh_3)_4$ (2.3 mg, 1.9 µmol, 0.2 equiv) were dissolved in toluene (2 mL) and DMF (1 mL) and the **General Procedure for Synthesis of the D-A Dyads** was followed. **Pyr-NDI** was obtained in 34% yield (4.5 mg, 3.3 µmol).

^{1}H NMR (601 MHz, CD_2Cl_2, rt): δ [ppm]: 9.33 - 9.31 (m, 1H), 8.86 (d, *J* = 7.4 Hz, 2H), 8.80 (s, 2H), 8.67 (s, 1H), 8.52 (d, *J* = 4.9 Hz, 1H), 8.44 (d, *J* = 4.8 Hz, 1H), 8.38 (d, *J* = 4.7 Hz, 1H), 8.21 - 8.20 (m, 2H), 8.16 - 8.11 (m, 3H), 8.07 (d, *J* = 1.8 Hz, 1H), 8.04 (d, *J* = 1.7 Hz, 1H), 8.00 (d, *J* = 8.8 Hz, 1H), 7.95 (s, 1H), 7.95 - 7.92 (m, 1H), 7.91 - 7.88 (m, 1H), 7.66 - 7.61 (m, 2H), 7.27 (s, 2H), 7.25 (s, 2H), 2.61 (s, 3H), 2.58 (s, 3H), 2.01 (s, 6H), 1.87 (s, 6H), 1.56 (s, 9H), 0.99 - 0.69 (m, 23H).

^{13}C{^{1}H} NMR (151 MHz, CD_2Cl_2, rt): δ [ppm]: 164.27, 163.49, 163.05, 156.23, 149.77, 147.62, 147.23, 146.99, 146.06, 145.01, 144.76, 143.85, 143.79, 141.83, 141.35, 138.90, 138.75, 138.22, 138.10, 137.06, 135.71, 134.93, 134.21, 134.18, 133.20, 132.89, 132.26, 132.00, 131.49, 131.43, 131.41, 131.17, 130.78, 130.02, 129.31, 128.06, 128.01, 127.85, 127.54, 127.30, 127.20, 127.15, 127.09, 126.64, 126.58, 126.10, 124.62, 124.28, 123.79, 123.50, 122.44, 122.14, 121.68, 121.57, 118.54, 112.93, 35.23, 32.36, 32.16, 31.96, 31.62, 29.94, 29.90, 26.80, 22.96, 22.85, 21.43, 21.38, 21.28, 14.19, 14.11.

UV/Vis (CH_2Cl_2): *λ* [nm] (*ε* [$M^{-1}cm^{-1}$]): 357 (39000), 382 (47000), 400 (48000), 448 (53000), 474 (72000), 501 (75000), 586 (11000), 630 (7000), 684 (5000).

HRMS (MALDI, DCTB) for $C_{89}H_{76}N_6NiO_4$ (M^+), calcd.: 1350.5276, found: 1350.5258.

TLC: R_f [%]: 0.50 (hexanes/CH_2Cl_2 1:2).

Pyrene-Fused-Porphyrin-Perylenediimide Pyr-PDI

Pyrene-fused boronic-ester porphyrin **PyrBpin** (10 mg, 11 µmol, 1.05 equiv), 5,6,12,13-tetrakis(4-(*tert*-butyl)phenoxy)-2-(4-iodophenyl)-9-undecylanthra[2,1,9-def:6,5,10-d'e'f']diisoquinoline-1,3,8,10(2H,9H)-tetraone **I-PDI** (13 mg, 9.7 µmol, 1 equiv), Cs_2CO_3 (9.5 mg, 29 µmol, 3 equiv) and $Pd(PPh_3)_4$ (2.3 mg, 1.9 µmol, 0.2 equiv) were dissolved in toluene (2 mL) and DMF (1 mL) and the **General Procedure for Synthesis of the D-A Dyads** was followed. **Ph-PDI** was obtained in 20% yield (4.0 mg, 1.9 µmol).

^{1}H NMR (601 MHz, CD_2Cl_2/CS_2, rt): δ [ppm]: 9.28 (d, J = 5.0 Hz, 1H), 8.62 (s, 1H), 8.52 (d, J = 4.8 Hz, 1H), 8.40 (d, J = 4.9 Hz, 1H), 8.35 (d, J = 4.7 Hz, 1H), 8.28 (s, 2H), 8.19 - 8.17 (m, 2H), 8.16 - 8.10 (m, 3H), 8.09 - 8.06 (m, 2H), 8.06 - 8.03 (m, 2H), 7.99 - 7.98 (m, 1H), 7.94 - 7.89 (m, 3H), 7.57 - 7.52 (m, 2H), 7.39 - 7.34 (m, 8H), 7.29 - 7.27 (m, 2H), 7.26 - 7.24 (m, 2H), 6.99 - 6.90 (m, 8H), 2.69 (s, 3H), 2.65 (s, 3H), 2.08 (s, 6H), 1.95 (s, 6H), 1.68 (s, 9H), 1.42 (m, 36H), 1.33 - 0.94 (m, 23H).

^{13}C{^{1}H} NMR (151 MHz, CD_2Cl_2/CS_2, rt): δ [ppm]: 164.03, 163.05, 162.86, 156.48, 156.40, 156.00, 153.42, 153.23, 153.18, 149.37, 147.68, 147.61, 147.40, 147.32, 147.20, 146.03, 145.00, 144.70, 144.07, 143.97, 141.98, 141.09, 138.90, 138.77, 138.19, 137.98, 137.36, 136.01, 135.34, 134.05, 133.85, 133.56, 133.32, 133.20, 133.08, 132.75, 132.28, 131.76, 131.38, 130.92, 130.15, 129.49, 128.44, 128.41, 128.38, 128.24, 127.88, 127.77, 127.31, 127.06, 127.04, 126.42, 124.95, 124.71, 123.87, 123.82, 123.63, 123.07, 122.94, 122.41, 122.11, 121.98, 121.79, 121.43, 121.42, 120.63, 120.38, 120.32, 120.19, 120.16, 119.85, 119.77, 119.59, 118.57, 113.37, 35.20, 34.47, 32.75, 32.47, 32.05, 31.77, 31.77, 30.51, 27.21, 23.52, 22.02, 21.98, 21.91, 21.79, 14.80.

UV/Vis (CH_2Cl_2): λ [nm] (ε [$M^{-1}cm^{-1}$]): 400 (45000), 473 (69000), 501 (71000), 536 (37000), 583 (48000).

HRMS (MALDI, DCTB) for $C_{139}H_{128}N_6NiO_8$ (M^+), calcd.: 2066.9142, found: 2066.9146.

TLC: R_f [%]: 0.55 (hexanes/CH_2Cl_2 1:1).

HBC-Fused-Porphyrin-Naphthalenediimide HBC-NDI

HBC-fused boronic-ester porphyrin **HBCBpin** (10 mg, 6.5 µmol, 1.05 equiv), 2-(4-iodophenyl)-7-undecylbenzo[lmn][3,8]phenanthroline-1,3,6,8(2H,7H)-tetraone **I-NDI** (3.9 mg, 6.2 µmol, 1 equiv), Cs_2CO_3 (6.1 mg, 19 µmol, 3 equiv) and $Pd(PPh_3)_4$ (1.5 mg, 1.3 µmol, 0.2 equiv) were dissolved in toluene (2 mL) and DMF (1 mL) and the **General Procedure for Synthesis of the D-A Dyads** was followed. **HBC-NDI** was obtained in 40% yield (4.7 mg, 2.5 µmol).

^{1}H NMR (601 MHz, CD_2Cl_2, rt): δ [ppm]: 9.82 - 9.81 (m, 2H), 9.61 (d, *J* = 5.0 Hz, 1H), 9.42 (s, 1H), 9.39 - 9.33 (m, 6H), 9.29 - 9.26 (m, 2H), 8.78 (d, *J* = 7.4 Hz, 2H), 8.72 (s, 2H), 8.68 (d, *J* = 4.8 Hz, 1H), 8.48 - 8.45 (m, 2H), 8.41 (d, *J* = 4.7 Hz, 1H), 8.29 - 8.27 (m, 2H), 8.14 - 8.10 (m, 2H), 7.59 - 7.55 (m, 2H), 7.30 (s, 2H), 7.25 (s, 2H), 2.62 (s, 3H), 2.59 (s, 3H), 2.00 (s, 6H), 1.94 (s, 6H), 1.92 (s, 9H), 1.86 - 1.81 (m, 27H), 1.60 (s, 9H).

^{13}C{^{1}H} NMR (151 MHz, CD_2Cl_2, rt): δ [ppm]: 163.48, 156.35, 149.99, 149.88, 149.56, 149.24, 149.09, 146.87, 146.33, 145.37, 144.94, 144.40, 144.31, 141.76, 141.52, 139.18, 138.72, 138.48, 138.41, 137.35, 135.86, 135.13, 134.62, 134.27, 133.53, 133.38, 131.59, 131.46, 131.15, 131.13, 130.88, 130.85, 130.74, 130.63, 130.53, 130.43, 130.42, 130.19, 128.73, 128.33, 128.25, 127.72, 127.38, 127.27, 126.80, 125.08, 124.18, 124.11, 124.05, 123.89, 123.08, 122.99, 121.74, 121.24, 121.22, 121.18, 121.06, 120.91, 120.67, 119.79, 119.70, 119.64, 119.61, 119.57, 119.46, 119.40, 119.31, 119.04, 112.88, 32.36, 32.15, 32.07, 30.05, 26.94, 22.93, 21.62, 21.52, 21.50, 21.42, 14.14.

UV/Vis (CH_2Cl_2): *λ* [nm] (*ε* [$M^{-1}cm^{-1}$]): 362 (83000), 381 (100000), 439 (67000), 510 (90000), 592 (17000), 638 (11000).

HRMS (MALDI, DCTB) for $C_{113}H_{116}N_6NiO_4$ (M^+), calcd.: 1894.8406, found: 1894.8384.

TLC: R_f [%]: 0.40 (hexanes/CH_2Cl_2 1:1).

HBC-Fused-Porphyrin-Perylenediimide HBC-PDI

HBC-fused boronic-ester porphyrin **HBCBpin** (10 mg, 6.5 µmol, 1.05 equiv), 5,6,12,13-tetrakis(4-(*tert*-butyl)phenoxy)-2-(4-iodophenyl)-9-undecylanthra[2,1,9-def:6,5,10-d'e'f']diisoquinoline-1,3,8,10(2H,9H)-tetraone **I-PDI** (8.3 mg, 6.2 µmol, 1 equiv), Cs_2CO_3 (6.1 mg, 19 µmol, 3 equiv) and $Pd(PPh_3)_4$ (1.5 mg, 1.3 µmol, 0.2 equiv) were dissolved in toluene (2 mL) and DMF (1 mL) and the **General Procedure for Synthesis of the D-A Dyads** was followed. **Ph-PDI** was obtained in 68% yield (11 mg, 4.4 µmol).

^{1}H NMR (601 MHz, CD_2Cl_2, rt): δ [ppm]: 9.84 - 9.78 (m, 2H), 9.61 (d, *J* = 4.9 Hz, 1H), 9.41 (s, 1H), 9.39 - 9.32 (m, 6H), 9.28 (s, 1H), 9.25 (s, 1H), 8.67 (d, J = 4.7 Hz, 1H), 8.48 - 8.43 (m, 2H), 8.38 (d, *J* = 4.7 Hz, 1H), 8.32 (s, 2H), 8.25 - 8.22 (m, 2H), 8.19 - 8.10 (m, 4H), 7.59 - 7.58 (m, 2H), 7.33 - 7.30 (m, 8H), 7.28 (s, 2H), 7.23 (s, 2H), 6.94 - 6.92 (m, 4H), 6.92 - 6.86 (m, 4H), 2.61 - 2.59 (m, 6H), 1.98 (s, 6H), 1.92 (s, 6H), 1.90 (s, 9H), 1.84 (s, 18H), 1.82 (s, 9H), 1.58 (s, 9H), 1.33 (s, 18H), 1.31 (s, 18H), 1.23 – 0.81 (m, 23H).

^{13}C{^{1}H} NMR (151 MHz, CD_2Cl_2, rt): δ [ppm]: 163.89, 156.54, 156.32, 156.07, 153.66, 153.41, 149.96, 149.84, 149.59, 149.22, 149.07, 147.83, 147.74, 146.82, 146.30, 145.35, 144.88, 144.45, 144.35, 141.72, 141.21, 139.16, 138.70, 138.44, 138.38, 137.35, 135.86, 135.75, 134.56, 134.22, 133.64, 133.54, 133.41, 133.23, 131.60, 131.48, 131.16, 131.08, 130.90, 130.83, 130.73, 130.65, 130.49, 130.43, 130.19, 129.71, 128.70, 128.34, 128.23, 127.80, 127.23, 127.04, 125.10, 124.18, 124.07, 123.05, 122.97, 122.94, 121.91, 121.73, 121.67, 121.27, 121.25, 121.18, 121.09, 120.94, 120.83, 120.69, 120.44, 120.02, 119.76, 119.67, 119.65, 119.60, 119.56, 119.47, 119.43, 119.40, 119.28, 118.99, 112.84, 36.05, 34.64, 32.68, 32.35, 32.15, 32.07, 31.57, 30.06, 26.91, 22.94, 21.61, 21.51, 21.48, 21.40, 14.25, 14.17.

UV/Vis (CH_2Cl_2): *λ* [nm] (*ε* [$M^{-1}cm^{-1}$]): 380 (80000), 439 (70000), 511 (86000), 525 (88000), 584 (58000), 635 (11000).

HRMS (MALDI, DCTB) for $C_{181}H_{168}N_6NiO_8$ (M^+), calcd.: 2611.2272, found: 2611.2258.

TLC: R_f [%]: 0.50 (hexanes/CH_2Cl_2 3:1).

2.6 Synthesis of Donor-Acceptor-Donor Triads

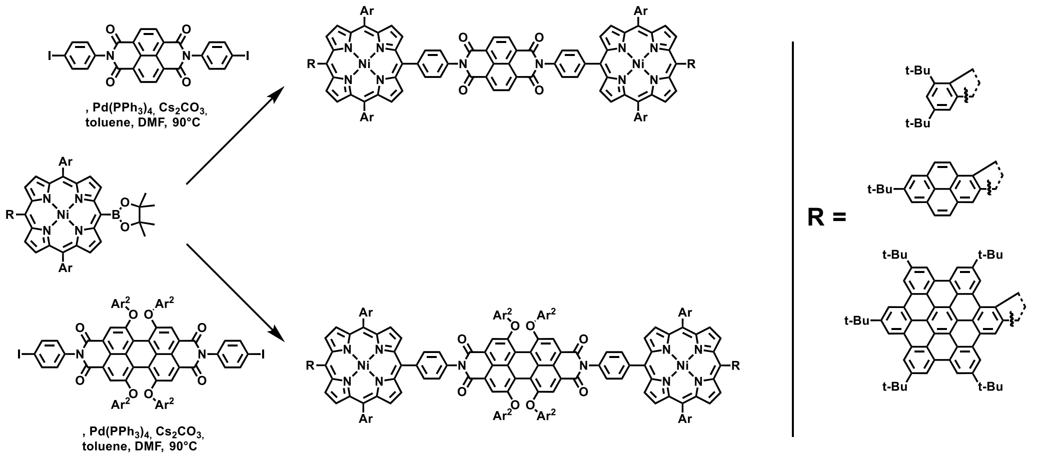

Scheme S7. General scheme for the Synthesis of the D-A-D Triads. Ar = mesityl; Ar^2 = 4-*t*Bu-phenyl.

General Procedure for Synthesis of the D-A-D Triads

Fused boronic-ester porphyrin, rylenediimide**,** Cs_2CO_3, and $Pd(PPh_3)_4$ were dissolved in toluene and DMF and were degassed under sonication. The reaction mixture was heated to 90 °C for 18 h. After cooling to rt, the solvent was removed under reduced pressure, and the crude was subjected to silica plug filtration (hexanes/CH_2Cl_2 - 1:1, Ø 3 x 12 cm) to remove the inorganics. The crude was further purified by size exclusion chromatography (Biobeads SX1, toluene, Ø 5 x 120 cm). After filtration through silica (hexanes/CH_2Cl_2 - 1:1, Ø 3 x 12 cm), the product was obtained as a dark solid.

Bis-(Phenyl-Fused-Porphyrin)-Naphthalenediimide Ph-NDI-Ph

Phenyl-fused boronic-ester porphyrin **PhBpin** (10 mg, 11 µmol, 2.1 equiv), 2,7-bis(4-iodophenyl)benzo[lmn][3,8]phenanthroline-1,3,6,8(2H,7H)-tetraone **I-NDI-I** (3.5 mg, 5.2 µmol, 1 equiv), Cs_2CO_3 (5.1 mg, 16 µmol, 3 equiv), and $Pd(PPh_3)_4$ (1.2 mg, 1.0 µmol, 0.2 equiv) were dissolved in toluene (2 mL) and DMF (1 mL) and the **General Procedure for Synthesis of the D-A-D Triads** was followed. **Ph-NDI-Ph** was obtained in 28% yield (2.9 mg, 1.5 µmol).

^{1}H NMR (601 MHz, CD_2Cl_2, rt): **δ [ppm]:** 9.12 (d, *J* = 5.0 Hz, 2H), 8.98 (s, 4H), 8.49 - 8.47 (m, 4H), 8.42 (d, *J* = 4.7 Hz, 2H), 8.23 (d, *J* = 4.9 Hz, 2H), 8.20 - 8.16 (m, 6H),

7.98 (d, J = 1.6 Hz, 2H), 7.70 (s, 2H), 7.69 - 7.66 (m, 4H), 7.25 - 7.21 (m, 8H), 7.05 (d, J = 1.6 Hz, 2H), 2.57 (m, 12H), 1.94 (s, 12H), 1.85 (s, 12H), 1.57 (s, 18H), 1.48 (s, 18H).

$^{13}C\{^1H\}$ NMR (151 MHz, CD_2Cl_2, rt): δ [ppm]: 163.53, 154.80, 153.18, 150.92, 148.68, 147.92, 146.57, 145.91, 144.86, 144.54, 143.66, 141.80, 141.22, 139.12, 138.89, 138.34, 138.24, 137.33, 135.79, 135.07, 134.40, 133.99, 133.38, 133.13, 131.79, 131.02, 130.83, 130.07, 128.16, 127.76, 127.70, 127.64, 127.33, 126.65, 123.30, 122.09, 121.32, 120.98, 118.28, 113.11, 35.52, 35.34, 32.29, 31.18, 30.05, 28.98, 21.52, 21.48, 21.34.

UV/Vis (CH_2Cl_2): λ [nm] (ε [$M^{-1}cm^{-1}$]): 380 (77000), 441 (149000), 571 (15000).

HRMS (MALDI, DCTB) for $C_{130}H_{110}N_{10}Ni_2O_4$ (M^+), calcd.: 1990.7413, found: 1990.7429.

TLC: R_f [%]: 0.20 (hexanes/CH_2Cl_2 1:2).

Bis-(Phenyl-Fused-Porphyrin)-Perylenediimide Ph-PDI-Ph

Phenyl-fused boronic-ester porphyrin **PhBpin** (10 mg, 11 µmol, 2.1 equiv), 5,6,12,13-tetrakis(4-(*tert*-butyl)phenoxy)-2,9-bis(4-iodophenyl)anthra[2,1,9-def:6,5,10-d'e'f']diisoquinoline-1,3,8,10(2H,9H)-tetraone **I-PDI-I** (7.2 mg, 5.2 µmol, 1 equiv), Cs_2CO_3 (5.1 mg, 16 µmol, 3 equiv), and $Pd(PPh_3)_4$ (1.2 mg, 1.0 µmol, 0.2 equiv) were dissolved in toluene (2 mL) and DMF (1 mL) and the **General Procedure for Synthesis of the D-A-D Triads** was followed. **Ph-PDI-Ph** was obtained in 19% yield (2.7 mg, 1.0 µmol).

^1H NMR (601 MHz, CD_2Cl_2, rt): δ [ppm]: 9.10 (d, J = 5.0 Hz, 2H), 8.45 (d, J = 4.8 Hz, 2H), 8.41 (d, J = 4.8 Hz, 2H), 8.36 - 8.32 (m, 6H), 8.17 (d, J = 4.8 Hz, 2H), 8.13 (d, J = 4.8 Hz, 2H), 8.09 - 8.06 (m, 4H), 7.97 (d, J = 1.6 Hz, 2H), 7.69 (s, 2H), 7.59 - 7.55 (m, 4H), 7.36 - 7.32 (m, 8H), 7.21 (d, J = 7.2 Hz, 8H), 7.04 (d, J = 1.6 Hz, 2H), 6.97 - 6.93 (m, 8H), 2.56 - 2.55 (m, 12H), 1.92 (s, 12H), 1.82 (s, 12H), 1.56 (s, 18H), 1.47 (s, 18H), 1.32 (s, 36H).

$^{13}C\{^1H\}$ NMR (151 MHz, CD_2Cl_2, rt): δ [ppm]: 163.87, 156.40, 154.74, 153.57, 153.13, 150.92, 148.61, 147.88, 147.85, 146.50, 145.86, 144.82, 144.48, 143.68, 141.31,

141.17, 139.09, 138.86, 138.29, 138.19, 137.33, 135.78, 135.63, 134.21, 133.94, 133.63, 133.37, 133.16, 131.06, 130.74, 129.99, 128.14, 127.72, 127.26, 127.07, 126.61, 123.26, 123.23, 122.03, 121.50, 121.38, 120.95, 120.72, 120.34, 119.57, 118.22, 113.02, 53.98, 31.57, 31.17, 28.98, 21.45, 21.32.

UV/Vis (CH_2Cl_2): λ [nm] (ε [$M^{-1}cm^{-1}$]) = 377 (80000), 441 (196000), 542 (50000), 582 (80000).

HRMS (MALDI, DCTB) for $C_{180}H_{162}N_{10}Ni_2O_8$ (M^+), calcd: 2707.1278, found: 2707.1226.

TLC: R_f [%] = 0.20 (hexanes/CH_2Cl_2 1:1).

Bis-(Pyrene-Fused-Porphyrin)-Naphthalenediimide Pyr-NDI-Pyr

Pyrene-fused boronic-ester porphyrin **PyrBpin** (10 mg, 10 µmol, 2.1 equiv), 2,7-bis(4-iodophenyl)benzo[lmn][3,8]phenanthroline-1,3,6,8(2H,7H)-tetraone **I-NDI-I** (3.2 mg, 4.8 µmol, 1 equiv), Cs_2CO_3 (4.7 mg, 14 µmol, 3 equiv), and $Pd(PPh_3)_4$ (1.1 mg, 1.0 µmol, 0.2 equiv) were dissolved in toluene (2 mL) and DMF (1 mL) and the **General Procedure for Synthesis of the D-A-D Triads** was followed. **Pyr-NDI-Pyr** was obtained in 21% yield (2.1 mg, 1.0 µmol).

^1H NMR (601 MHz, CD_2Cl_2/CS_2, rt): δ [ppm]: 9.28 (d, *J* = 4.9 Hz, 2H), 8.99 (s, 2H), 8.61 (s, 2H), 8.52 (d, *J* = 4.8 Hz, 2H), 8.43 (d, *J* = 4.7 Hz, 2H), 8.38 (s, 2H), 8.22 - 8.21 (m, 4H), 8.15 (s, 2H), 8.11 (s, 2H), 8.04 (s, 2H), 8.01 (s, 2H), 7.92 - 7.89 (m, 6H), 7.66 - 7.63 (m, 4H), 7.35 (s, 4H), 7.27 (s, 4H), 7.25 (s, 4H), 2.65 (s, 6H), 2.63 (s, 6H), 2.06 (s, 12H), 1.93 (s, 12H), 1.61 (s, 18H).

$^{13}C\{^1H\}$ NMR (151 MHz, CD_2Cl_2/CS_2, rt): δ [ppm]: 162.99, 156.69, 149.70, 147.89, 147.75, 147.43, 146.29, 145.26, 145.00, 144.16, 144.08, 142.19, 141.86, 139.07, 138.93, 138.40, 138.21, 136.10, 134.26, 134.20, 133.25, 132.80, 132.42, 131.89, 131.79, 131.09, 130.31, 129.62, 128.52, 128.45, 128.37, 128.04, 127.78, 127.65, 127.46, 126.56, 124.99, 124.81, 123.98, 123.74, 122.62, 122.26, 122.05, 121.64, 118.78, 113.56, 32.06, 30.45, 21.87, 21.74.

UV/Vis (CH_2Cl_2): λ [nm] (ε [$M^{-1}cm^{-1}$]) = 339 (49000), 402 (76000), 418 (76000), 475 (110000), 500 (109000), 586 (55000), 650 (15000).

HRMS (MALDI, DCTB) for $C_{142}H_{102}N_{10}Ni_2O_4$ (M^+), calcd.: 2126.6787, found: 2126.6781.

TLC: R_f [%]: 0.60 (hexanes/CH_2Cl_2 1:3).

Bis-(Pyrene-Fused-Porphyrin)-Perylenediimide Pyr-PDI-Pyr

Pyrene-fused boronic-ester porphyrin **PyrBpin** (10 mg, 10 µmol, 2.1 equiv), 5,6,12,13-tetrakis(4-(*tert*-butyl)phenoxy)-2,9-bis(4-iodophenyl)anthra[2,1,9-def:6,5,10-d'e'f']diisoquinoline-1,3,8,10(2H,9H)-tetraone **I-PDI-I** (6.7 mg, 4.8 µmol, 1 equiv), Cs_2CO_3 (4.7 mg, 14 µmol, 3 equiv), and $Pd(PPh_3)_4$ (1.1 mg, 1.0 µmol, 0.2 equiv) were dissolved in toluene (2 mL) and DMF (1 mL) and the **General Procedure for Synthesis of the D-A-D Triads** was followed. **Pyr-PDI-Pyr** was obtained in 23% yield (3.0 mg, 1.1 µmol).

^{1}H NMR (601 MHz, CD_2Cl_2, rt): δ [ppm]: 9.23 (d, *J* = 4.7 Hz, 2H), 8.56 (s, 2H), 8.48 (d, *J* = 4.8 Hz, 2H), 8.37 (d, *J* = 4.7 Hz, 2H), 8.31 (d, *J* = 4.7 Hz, 2H), 8.28 - 8.25 (m, 4H), 8.13 - 8.16 (m, 4H), 8.05 - 7.96 (m, 10H), 7.88 - 7.86 (m, 4H), 7.53 - 7.52 (m, 4H), 7.35 - 7.33 (m, 12H), 7.24 (s, 4H), 7.21 (s, 4H), 6.97 – 6.90 (m, 8H), 2.66 -2.58 (m 12H), 2.06 (s, 12H), 1.91 (s, 12H), 1.62 (s, 9H), 1.40 (s, 9H), 1.31 (s, 36H).

^{13}C{^{1}H} NMR (101 MHz, $CDCl_3$, rt): δ [ppm]: 162.95, 156.65, 156.45, 153.44, 149.42, 147.88, 147.78, 147.60, 147.37, 146.14, 145.18, 144.83, 144.26, 144.13, 142.11, 141.24, 139.05, 138.88, 138.28, 138.05, 138.02, 137.67, 137.51, 136.17, 134.11, 133.89, 133.75, 133.40, 133.26, 132.92, 132.44, 131.97, 131.47, 131.04, 130.88, 130.26, 129.82, 129.67, 129.60, 128.77, 128.58, 128.55, 128.51, 128.36, 128.04, 127.92, 127.38, 127.21, 126.59, 125.09, 124.90, 123.94, 123.71, 123.44, 122.49, 122.18, 122.11, 121.89, 121.19, 120.64, 120.30, 119.86, 118.64, 113.54, 32.81, 32.21, 31.92, 30.61, 30.57, 30.29, 23.76, 22.17, 22.13, 22.02, 21.90.

UV/Vis (CH_2Cl_2): λ [nm] (ε [$M^{-1}cm^{-1}$]): 341 (63000), 400 (78000), 475 (109000), 502 (114000), 579 (30000).

HRMS (MALDI, DCTB) for $C_{192}H_{154}N_{10}Ni_2O_8$ (M^+), calcd.: 2843.0652, found:2843.0636.

TLC: R_f [%]: 0.35 (hexanes/CH_2Cl_2 1:1).

Bis-(HBC-Fused-Porphyrin)-Naphthalenediimide HBC-NDI-HBC

HBC-fused boronic-ester porphyrin **HBCBpin** (12 mg, 7.9 µmol, 2.1 equiv), 2,7-bis(4-iodophenyl)benzo[lmn][3,8]phenanthroline-1,3,6,8(2H,7H)-tetraone **I-NDI-I** (2.5 mg, 3.7 µmol, 1 equiv), Cs_2CO_3 (3.7 mg, 11 µmol, 3 equiv), and $Pd(PPh_3)_4$ (0.9 mg, 0.8 µmol, 0.2 equiv) were dissolved in toluene (2 mL) and DMF (1 mL) and the **General Procedure for Synthesis of the D-A-D Triads** was followed. **HBC-NDI-HBC** was obtained in 21% yield (2.5 mg, 0.8 µmol).

^{1}H NMR (601 MHz, CD_2Cl_2/CS_2, rt): δ [ppm]: 9.75 - 9.71 (m, 4H), 9.53 (d, *J* = 5.0 Hz, 2H), 9.38 (s, 2H), 9.32 - 9.24 (m, 10H), 9.22 (s, 2H), 9.19 (s, 2H), 8.74 (s, 2H), 8.67 (d, *J* = 4.6 Hz, 2H), 8.42 (d, *J* = 4.6 Hz, 2H), 8.39 (s, 2H), 8.36 (d, *J* = 4.5 Hz, 2H), 8.25 - 8.24 (m, 4H), 8.03 (d, *J* = 7.5 Hz, 4H), 7.49 (d, *J* = 7.5 Hz, 4H), 7.33 (s, 4H), 7.28 (s, 4H), 7.25 (s, 4H), 2.73 - 2.66 (m, 12H), 2.06 (s, 12H), 2.01 - 1.99 (m, 30H), 1.92 (s, 18H), 1.89 (m, 36H), 1.65 (s, 18H).

^{13}C{^{1}H} NMR (151 MHz, CD_2Cl_2/CS_2, rt): δ [ppm]: 162.25, 156.53, 149.76, 149.36, 149.24, 149.18, 148.70, 146.75, 146.16, 145.18, 144.74, 144.38, 144.30, 141.87, 141.60, 139.14, 138.76, 138.23, 138.22, 138.19, 138.14, 137.59, 136.10, 134.42, 133.91, 133.85, 131.73, 131.43, 131.39, 131.27, 131.15, 131.07, 130.97, 130.88, 130.76, 130.40, 130.37, 129.83, 128.92, 128.90, 128.79, 128.63, 127.82, 127.49, 127.32, 125.41, 124.51, 124.45, 124.39, 124.22, 124.05, 123.25, 123.23, 122.79, 121.72, 121.59, 121.50, 121.40, 121.36, 121.26, 121.20, 120.86, 119.76, 119.71, 119.63, 119.61, 119.48, 119.45, 118.95, 113.42, 53.98, 32.69, 32.59, 32.52, 32.47, 30.65, 28.09, 22.16, 22.04.

UV/Vis (CH_2Cl_2): *λ* [nm] (*ε* [$M^{-1}cm^{-1}$]): 380 (139000), 439 (100000), 511 (138000), 594 (28000), 638 (17000).

HRMS (MALDI, DCTB) for $C_{226}H_{182}N_{10}Ni_2O_8$ (M^+), calcd.: 3215.3047, found: 3215.3004.

TLC: R_f [%]: 0.30 (CH_2Cl_2).

Bis-(HBC-Fused-Porphyrin)-Perylenediimide HBC-PDI-HBC

HBC-fused boronic-ester porphyrin **HBCBpin** (10 mg, 6.5 μmol, 2.1 equiv), 5,6,12,13-tetrakis(4-(*tert*-butyl)phenoxy)-2,9-bis(4-iodophenyl)anthra[2,1,9-def:6,5,10-d'e'f']diisoquinoline-1,3,8,10(2H,9H)-tetraone **I-PDI-I** (4.3 mg, 3.1 μmol, 1 equiv), Cs_2CO_3 (3.1 mg, 9.4 μmol, 3 equiv), and $Pd(PPh_3)_4$ (0.7 mg, 0.6 μmol, 0.2 equiv) were dissolved in toluene (2 mL) and DMF (1 mL) and the **General Procedure for Synthesis of the D-A-D Triads** was followed. **HBC-PDI-HBC** was obtained in 16% yield (2.0 mg, 0.5 μmol).

^{1}H NMR (601 MHz, CD_2Cl_2, rt): δ [ppm]: 9.77 - 9.73 (m, 4H), 9.54 (d, J = 4.9 Hz, 2H), 9.38 (s, 2H), 9.32 - 9.25 (m, 12H), 9.22 (s, 2H), 9.18 (s, 2H), 8.66 (d, J = 4.7 Hz, 2H), 8.42 (d, J = 4.7 Hz, 2H), 8.39 s 8.35 (m, 4H), 8.28 (s, 2H), 8.21 (d, J = 4.7 Hz, 2H), 8.09 - 8.06 (m, 4H), 7.56 - 7.55 (m, 4H), 7.41 - 7.30 (m, 12H), 7.26 - 7.23 (m, 8H), 6.98 - 6.92 (m, 8H), 2.66 - 2.64 (m, 12H), 2.04 (s, 12H), 1.97 (s, 21H), 1.91 -1.89 (m, 45H), 1.62 (s, 18H), 1.41 (s, 18H).

^{13}C{^{1}H} NMR (151 MHz, CD_2Cl_2, rt): δ [ppm]: 162.51, 156.08, 156.01, 152.99, 149.33, 148.89, 148.79, 148.72, 148.17, 147.13, 146.23, 145.68, 144.72, 144.22, 143.99, 143.89, 141.35, 140.77, 138.64, 138.25, 137.67, 137.63, 137.10, 135.61, 135.00, 133.88, 133.47, 133.38, 133.27, 132.96, 131.24, 131.11, 130.98, 130.71, 130.70, 130.57, 130.48, 130.46, 130.43, 130.40, 130.29, 130.13, 130.07, 128.37, 128.15, 128.07, 127.47, 126.80, 126.74, 126.70, 124.93, 124.03, 123.96, 123.91, 123.73, 123.51, 122.97, 122.73, 122.28, 121.39, 121.23, 121.09, 120.95, 120.83, 120.75, 120.39, 120.18, 119.80, 119.39, 119.25, 119.16, 119.11, 118.96, 118.43, 32.15, 32.05, 31.98, 31.95, 31.44, 21.65, 21.59, 21.47.

UV/Vis (CH_2Cl_2): λ [nm] (ε [$M^{-1}cm^{-1}$]): 380 (145000), 439 (121000), 511 (151000), 525 (150000), 589 (92000), 635 (22000).

HRMS (MALDI, DCTB) for $C_{276}H_{234}N_{10}Ni_2O_8$ (M^+), calcd.: 3931.6913, found: 3931.6970.

TLC: R_f [%]: 0.80 (hexanes/CH_2Cl_2 1:3).

3 Spectral Appendix

^1H NMR (400 MHz, CD_2Cl_2, rt)

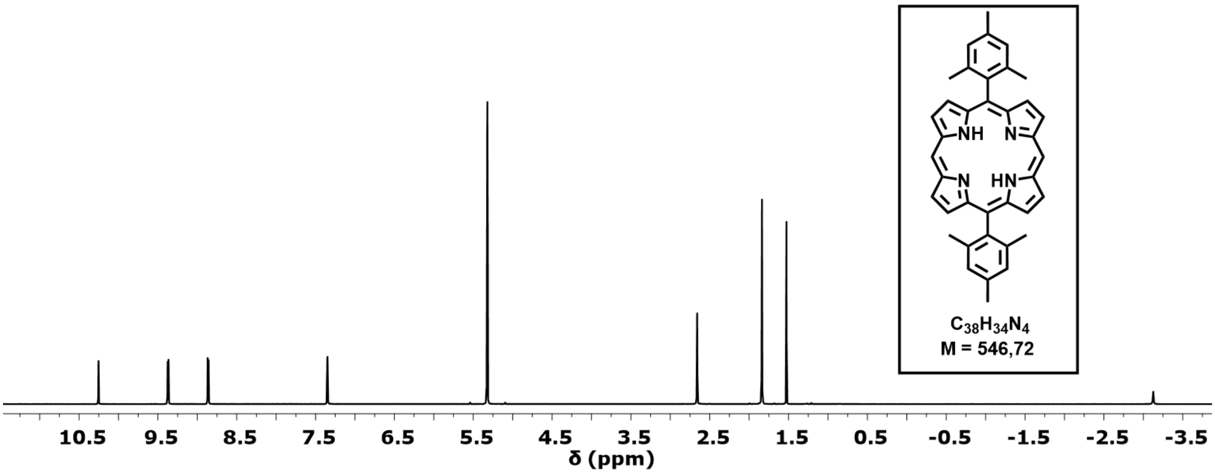

^{13}C NMR (101 MHz, CD_2Cl_2, rt)

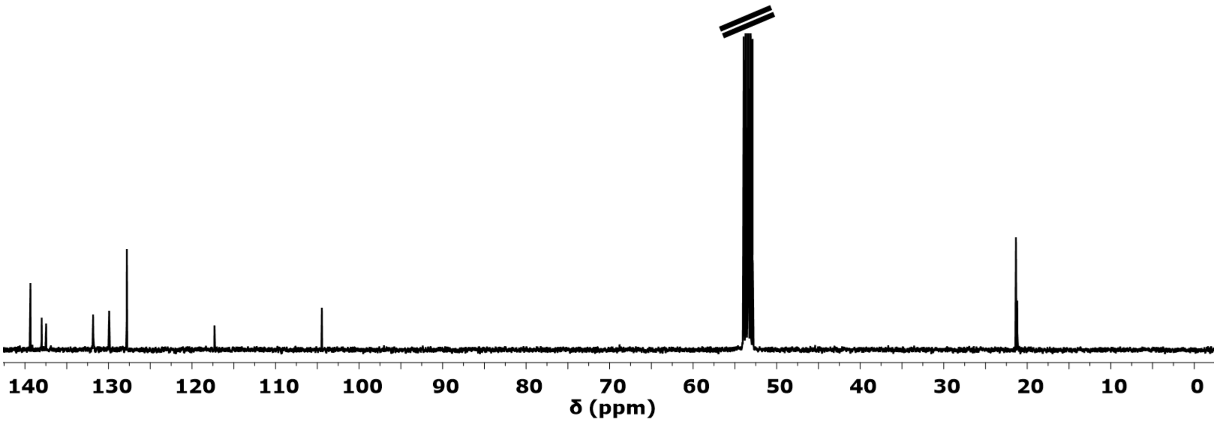

Figure S1. ^1H and ^{13}C NMR of **17**.

MS (MALDI)

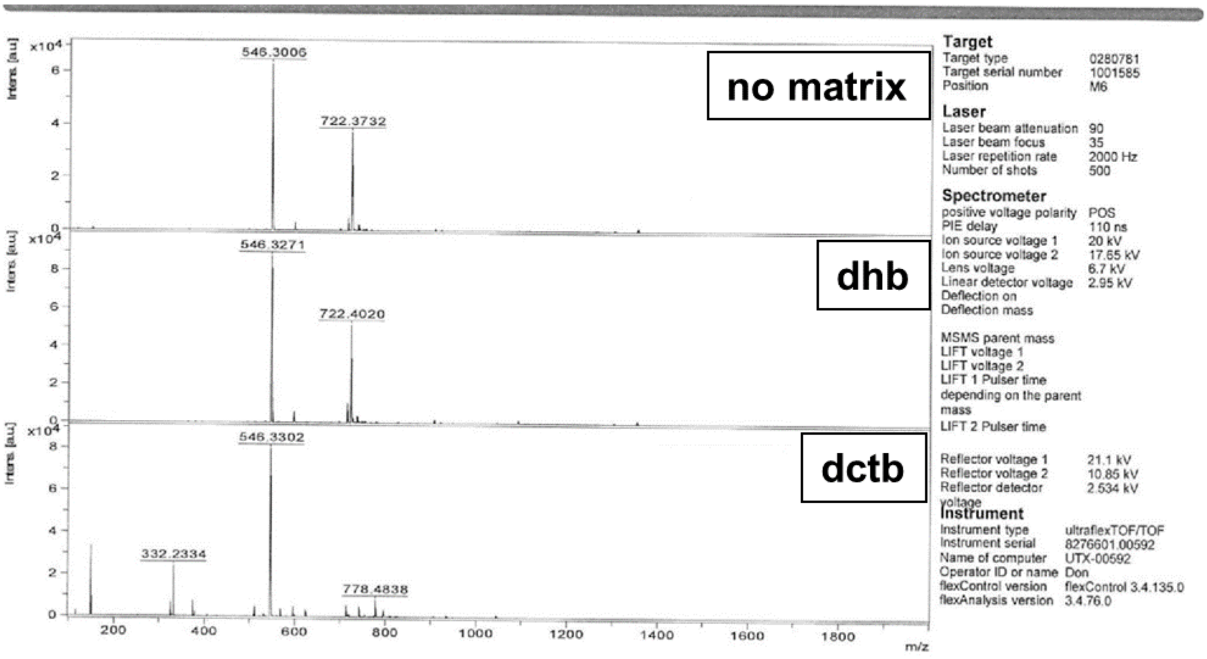

HRMS (MALDI)

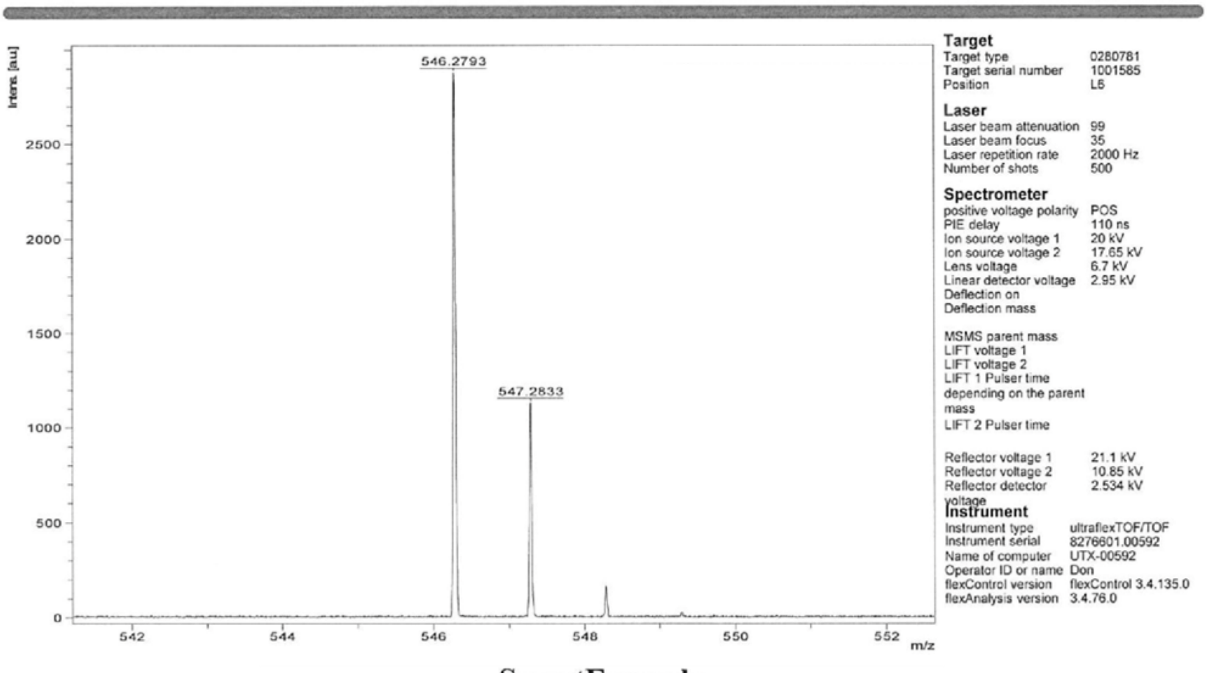

SmartFormula

Formula	Mass	Error	mSigma	DblEq	N rule	Electron Configuration
C 38 H 34 N 4	546.2778	2.8010	48.8215	24.00	ok	odd

Figure S2. MS/HRMS (MALDI) of **17**.

^{1}H NMR (400 MHz, CD_2Cl_2, rt)

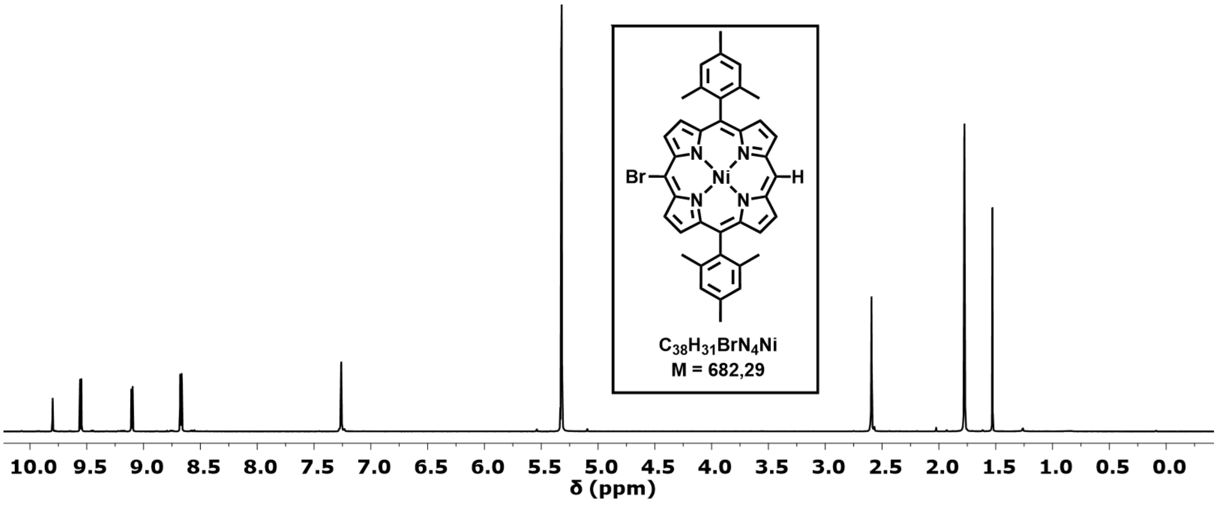

^{13}C NMR (101 MHz, CD_2Cl_2, rt)

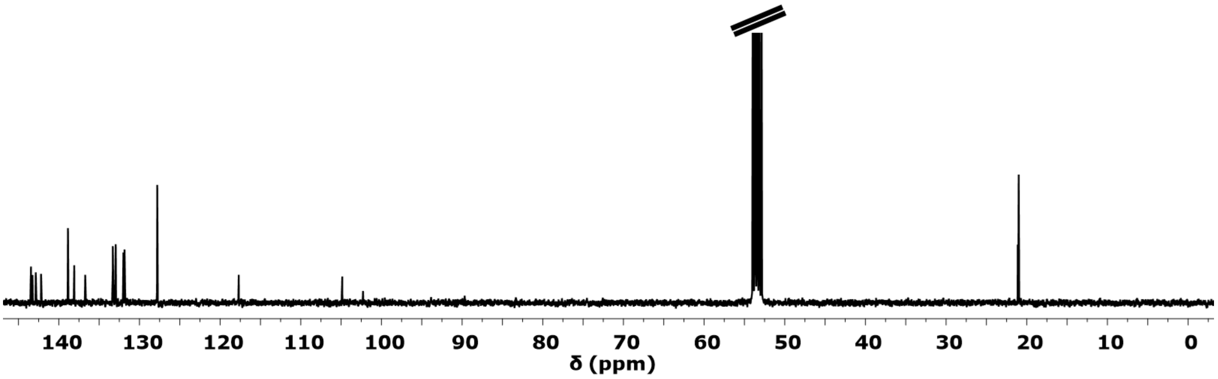

Figure S3. ^{1}H and ^{13}C NMR of **3**.

MS (MALDI)

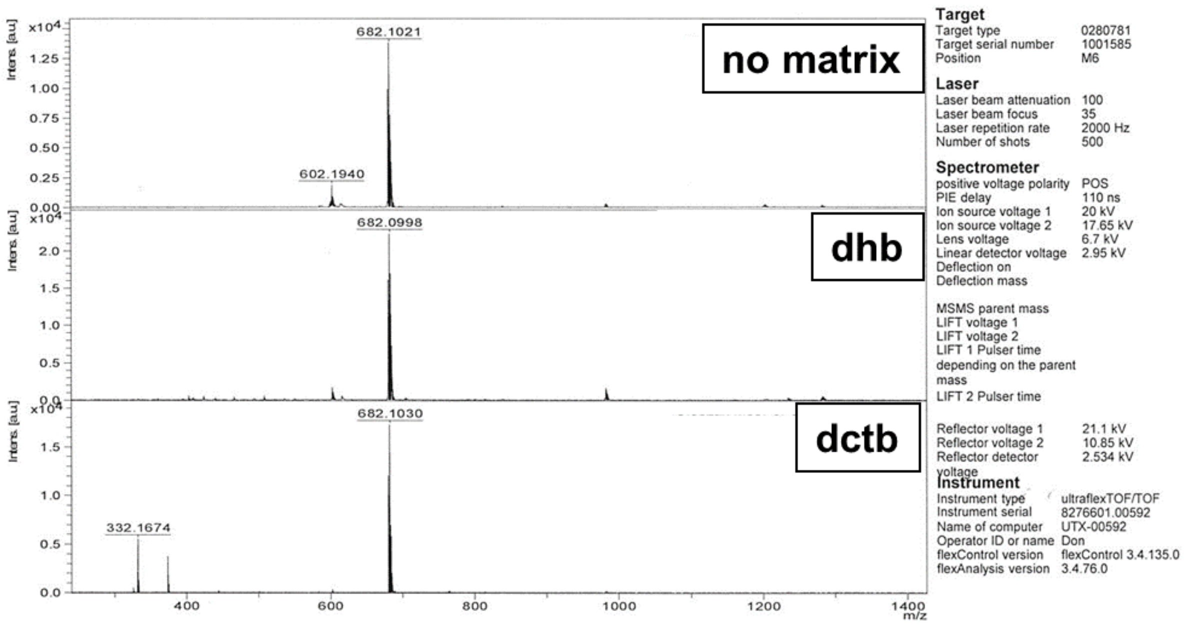

HRMS (MALDI)

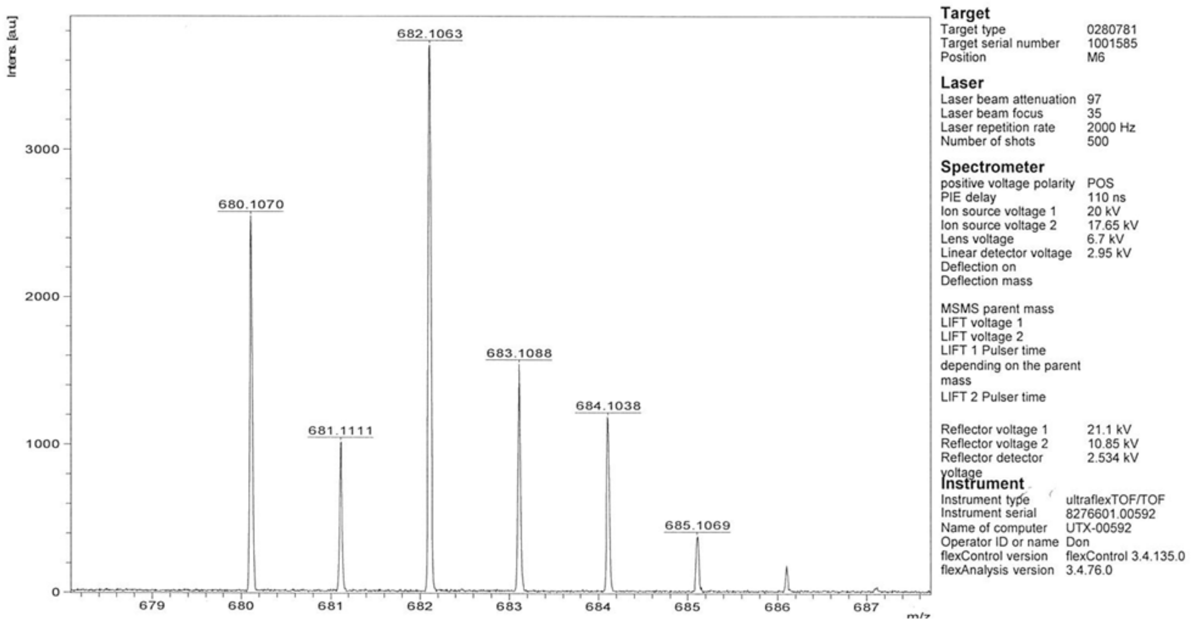

SmartFormula

Formula	Mass	Error	mSigma	DblEq	N rule	Electron Configuration
C 38 H 31 Br N 4 Ni	680.1080	1.4813	36.9206	25.00	ok	odd

Figure S4. MS/HRMS (MALDI) of **3**.

^{1}H NMR (400 MHz, $CDCl_3$, rt)

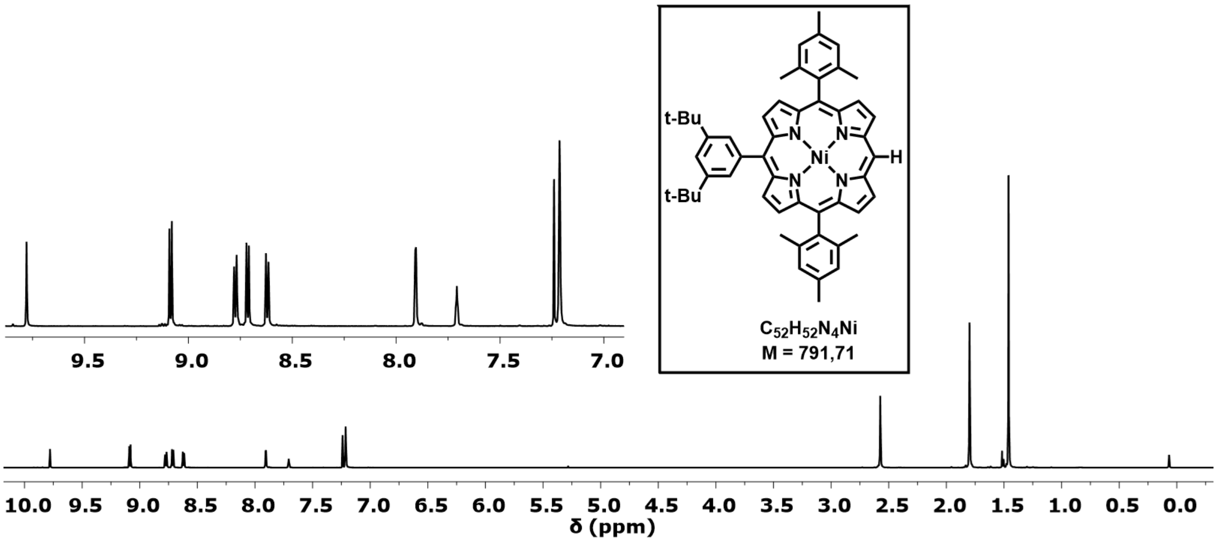

^{13}C NMR (101 MHz, $CDCl_3$, rt)

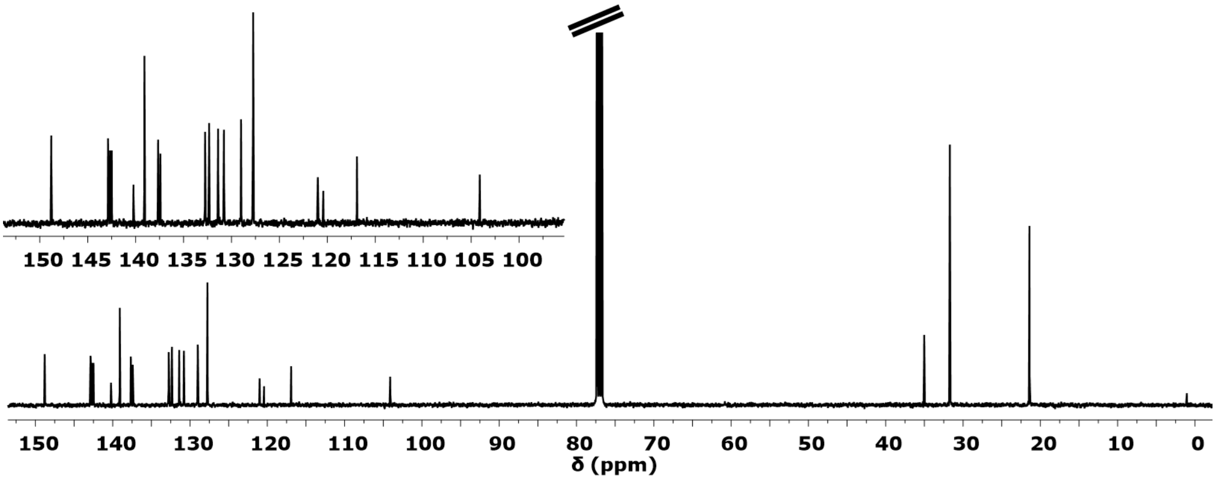

Figure S5. ^{1}H and ^{13}C NMR of **4**.

MS (MALDI)

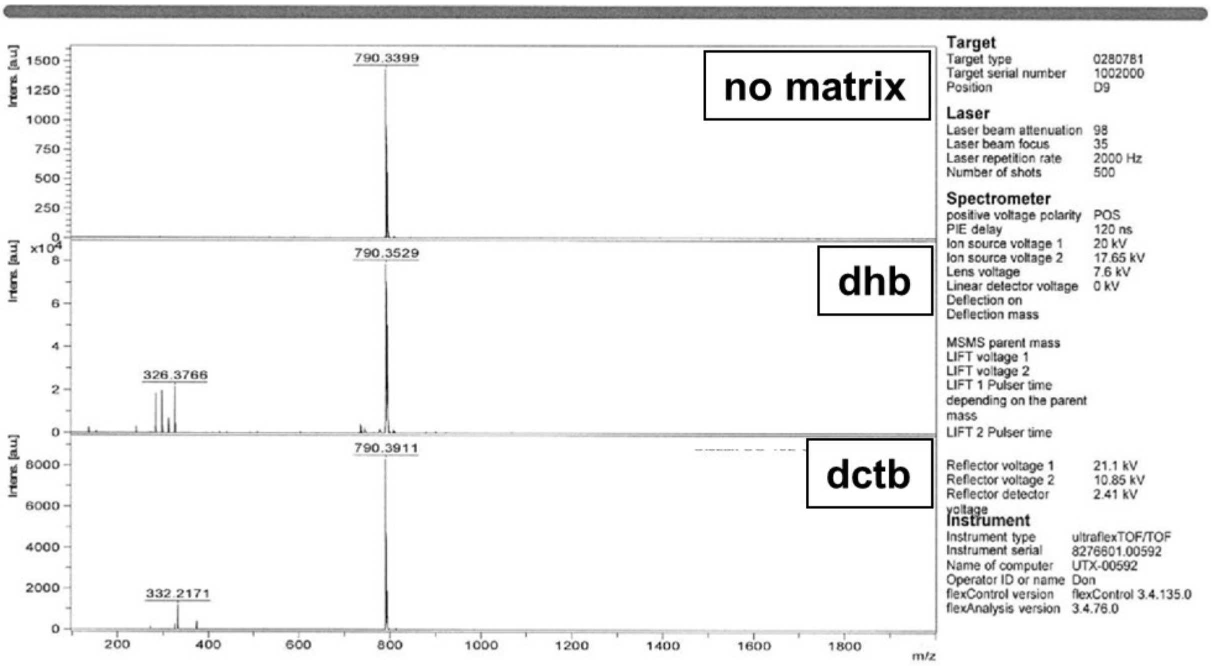

HRMS (MALDI)

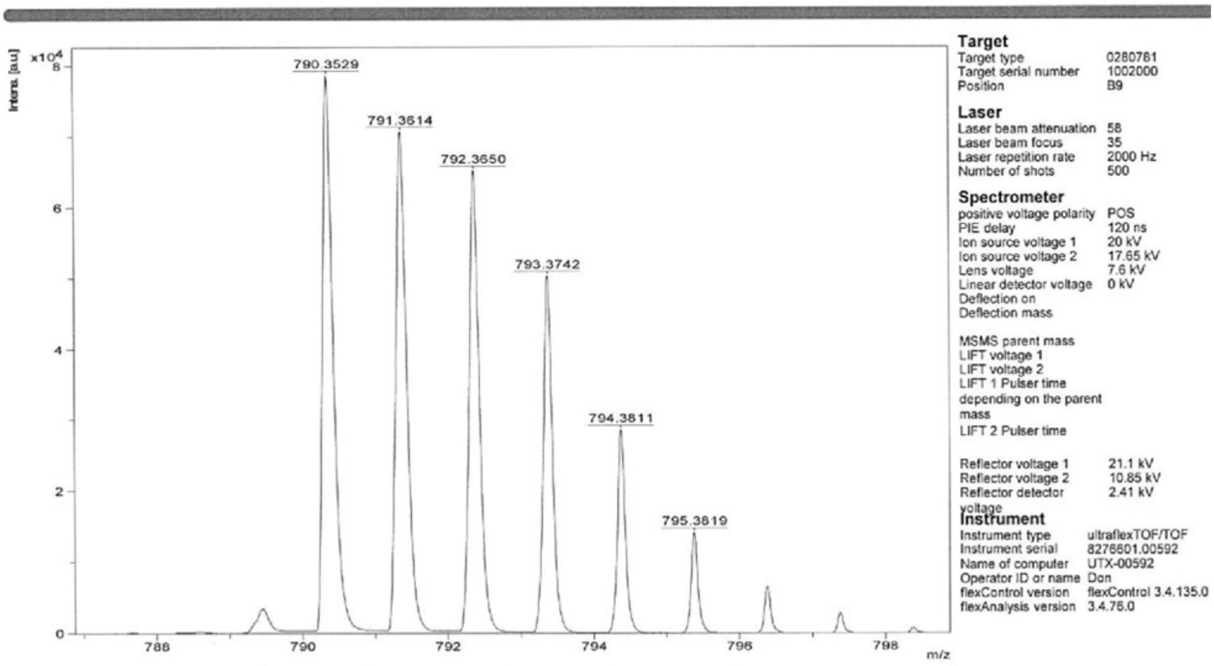

SmartFormula

Formula	Mass	Error	mSigma	DblEq	N rule	Electron Configuration
C 52 H 52 N 4 Ni	790.3540	1.3846	219.2905	29.00	ok	odd

Figure S6. MS/HRMS (MALDI) of **4**.

^{1}H NMR (400 MHz, $CDCl_3$, rt)

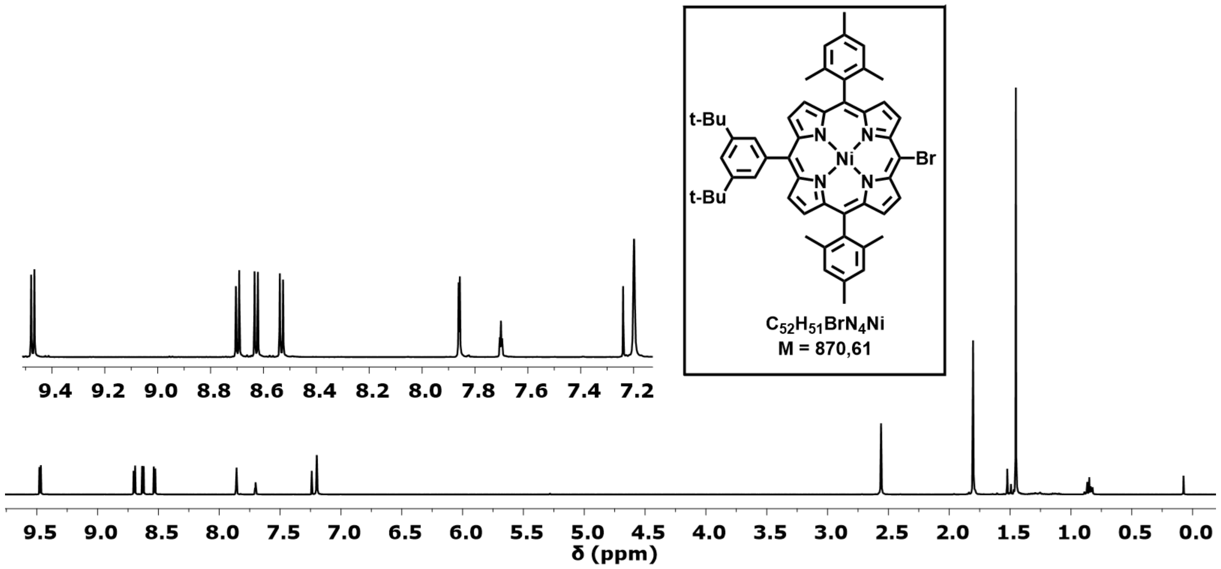

^{13}C NMR (101 MHz, $CDCl_3$, rt)

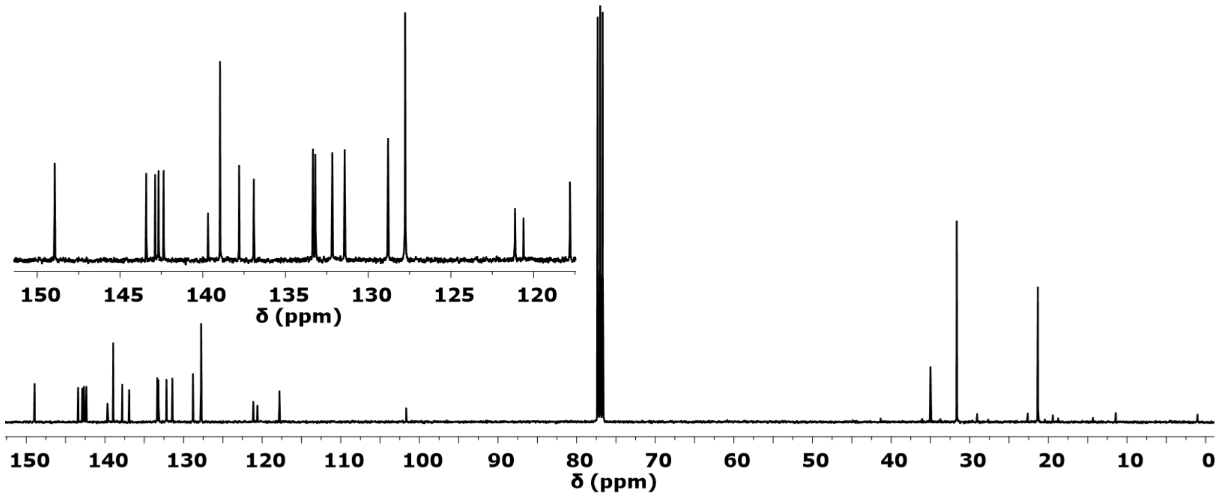

Figure S7. ^{1}H and ^{13}C NMR of **5**.

MS (MALDI)

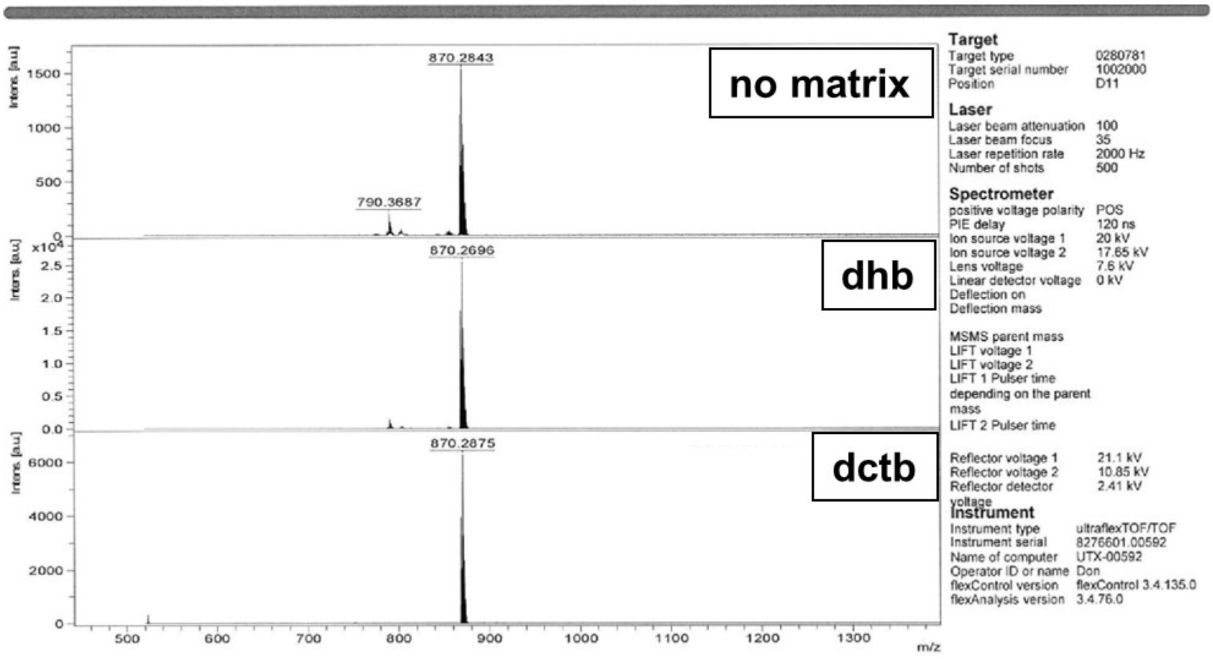

HRMS (MALDI)

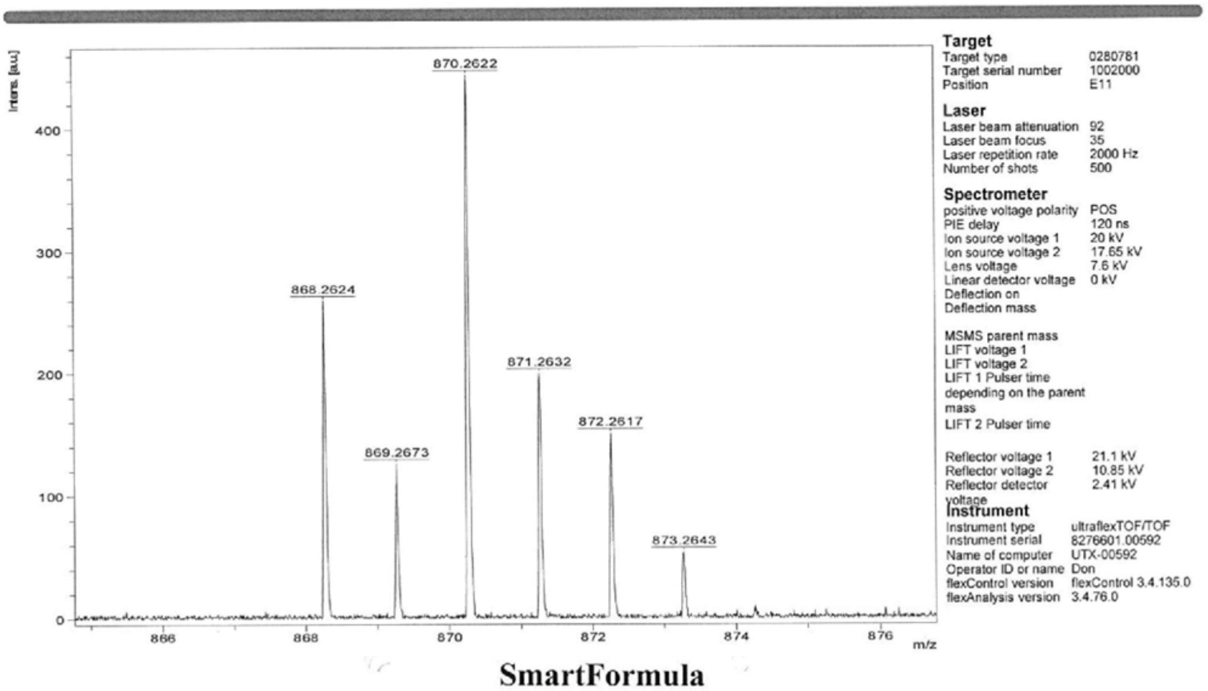

SmartFormula

Formula	Mass	Error	mSigma	DblEq	N rule	Electron Configuration
C 52 H 51 Br N 4 Ni	868.2645	2.4787	72.7265	29.00	ok	odd

Figure S8. MS/HRMS (MALDI) of **5**.

^{1}H NMR (600 MHz, CD_2Cl_2, rt)

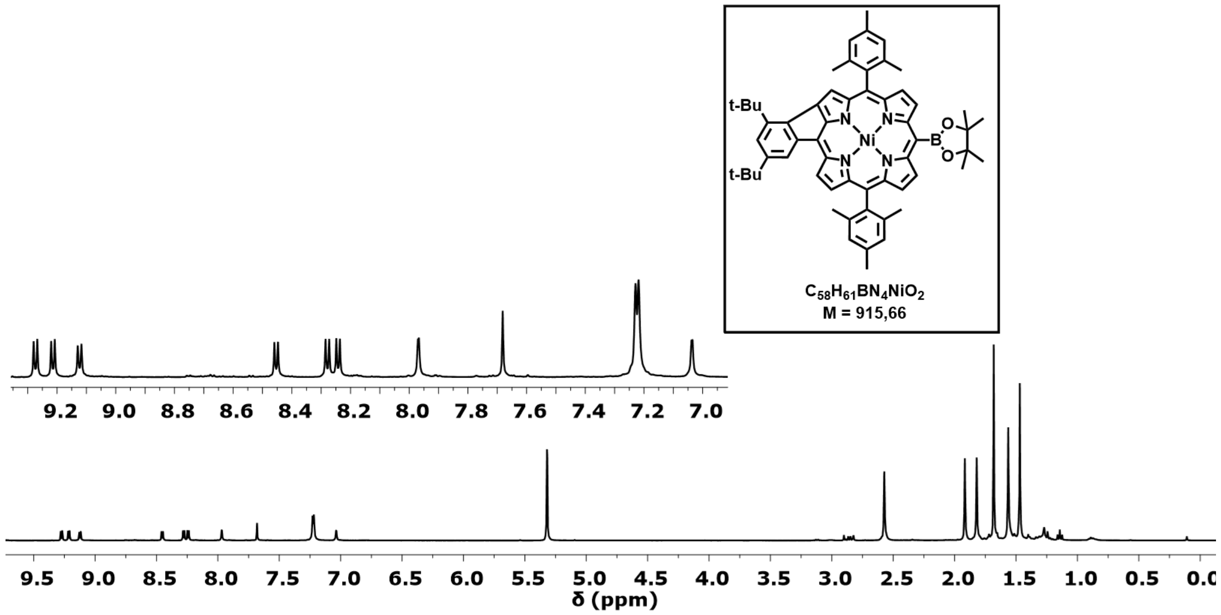

^{13}C NMR (101 MHz, CD_2Cl_2, rt)

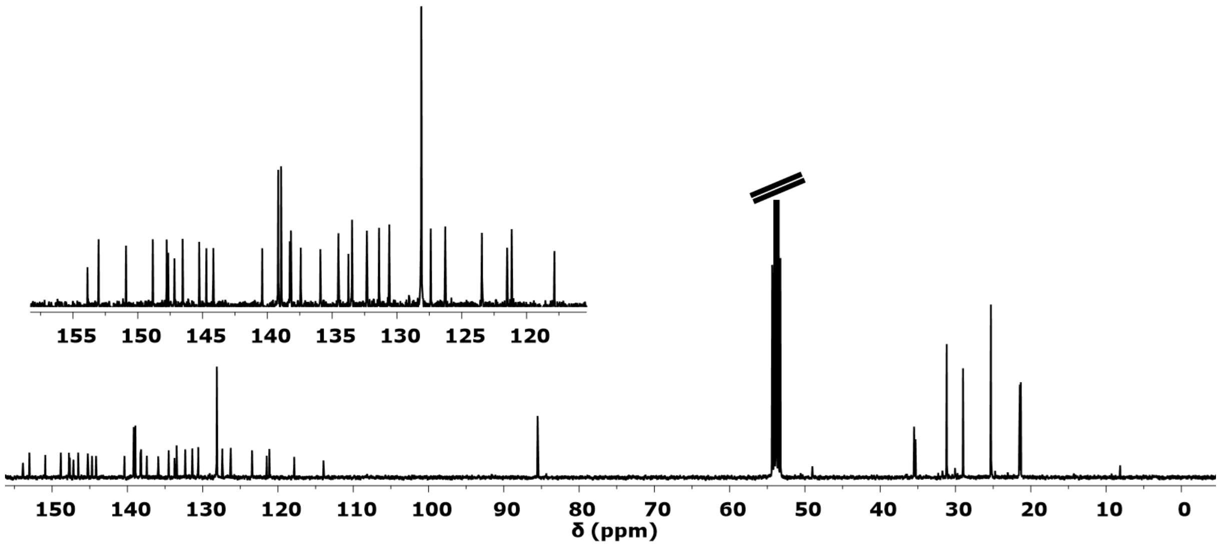

Figure S9. ^{1}H and ^{13}C NMR of **PhBpin**.

MS (MALDI)

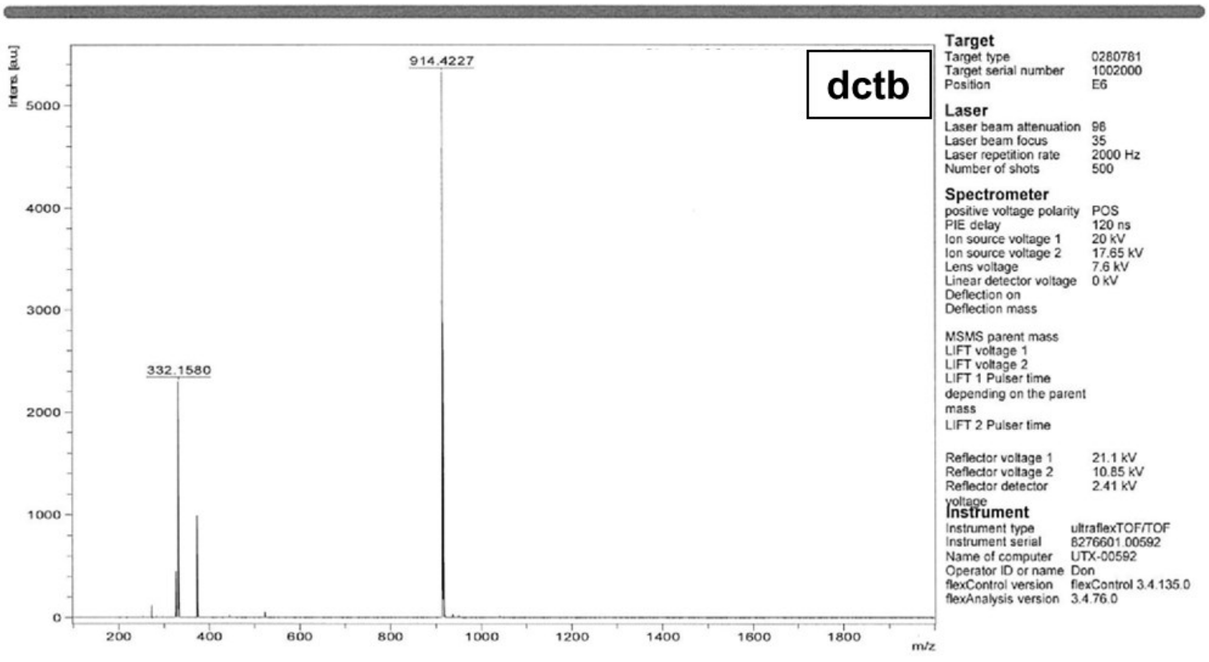

HRMS (MALDI)

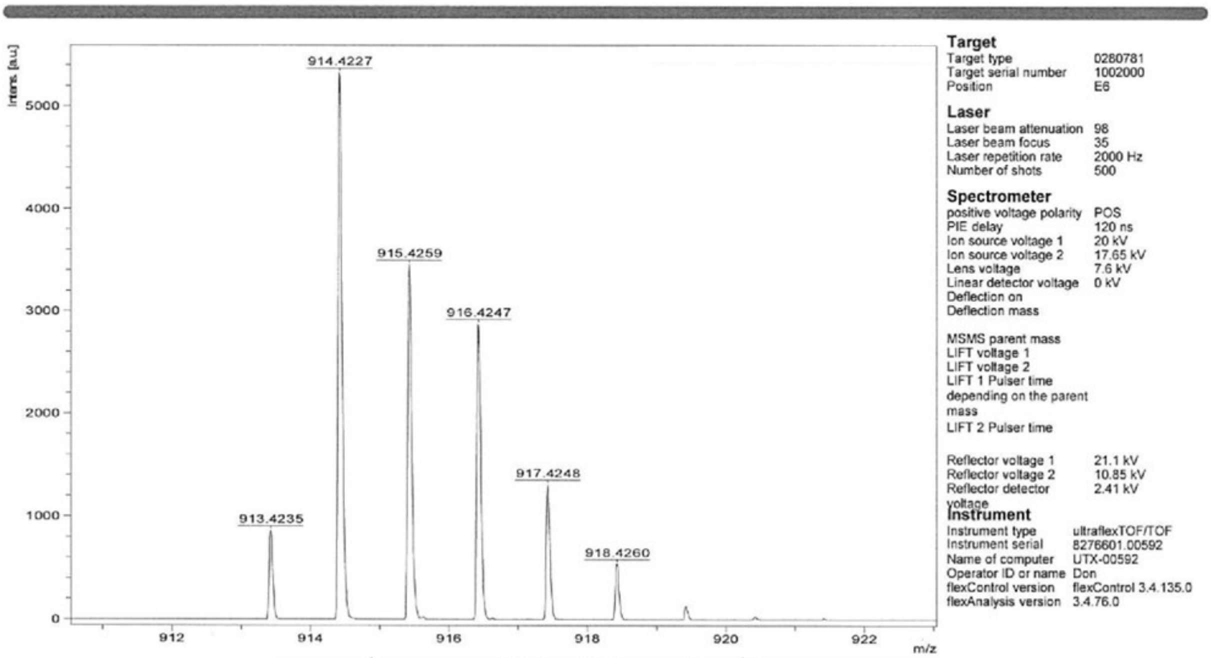

SmartFormula

Formula	Mass	Error	mSigma	DblEq	N rule	Electron Configuration
C 58 H 61 B N 4 Ni O 2	914.4236	0.9784	40.2851	31.00	ok	odd

Figure S10. MS/HRMS (MALDI) of **PhBpin**.

^{1}H NMR (400 MHz, $CDCl_3$, rt)

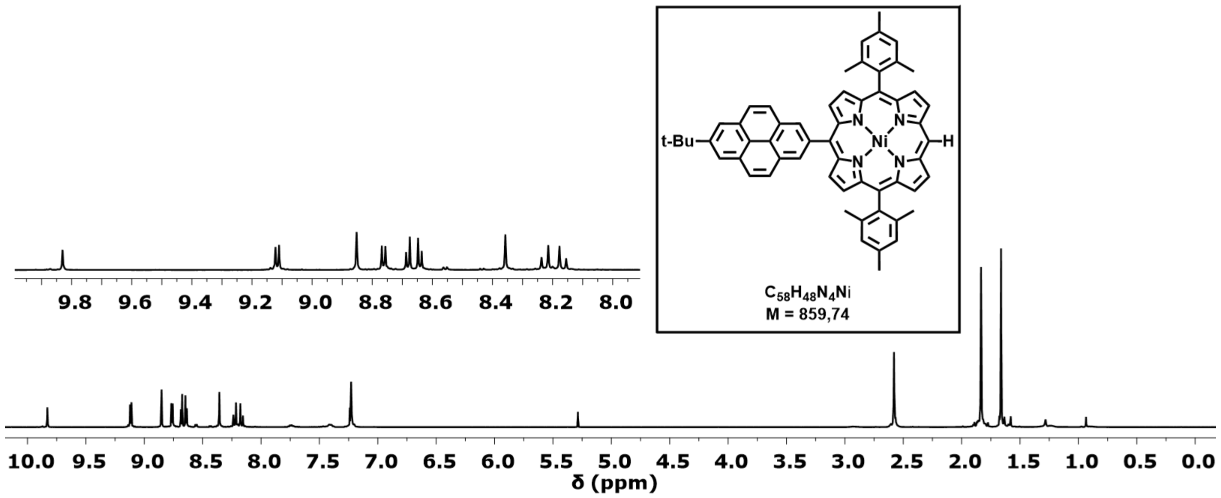

^{13}C NMR (101 MHz, $CDCl_3$, rt)

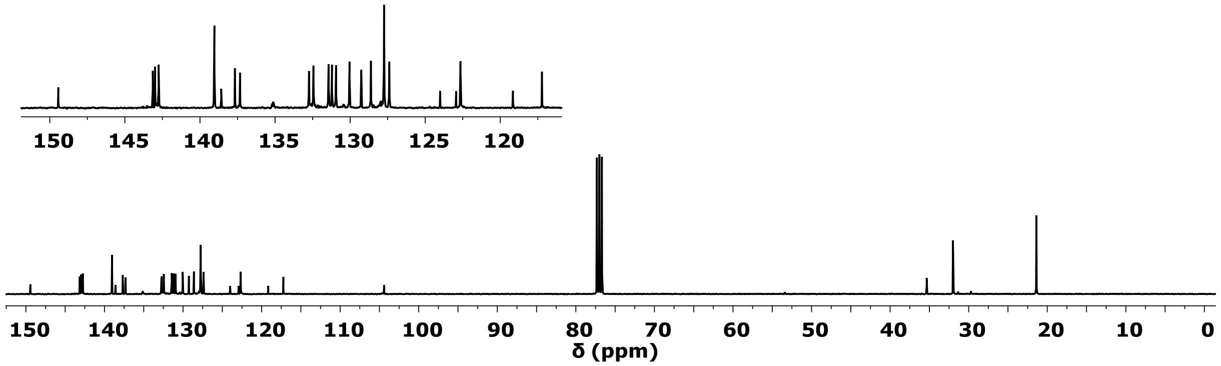

Figure S11. ^{1}H and ^{13}C NMR of **8**.

MS (MALDI)

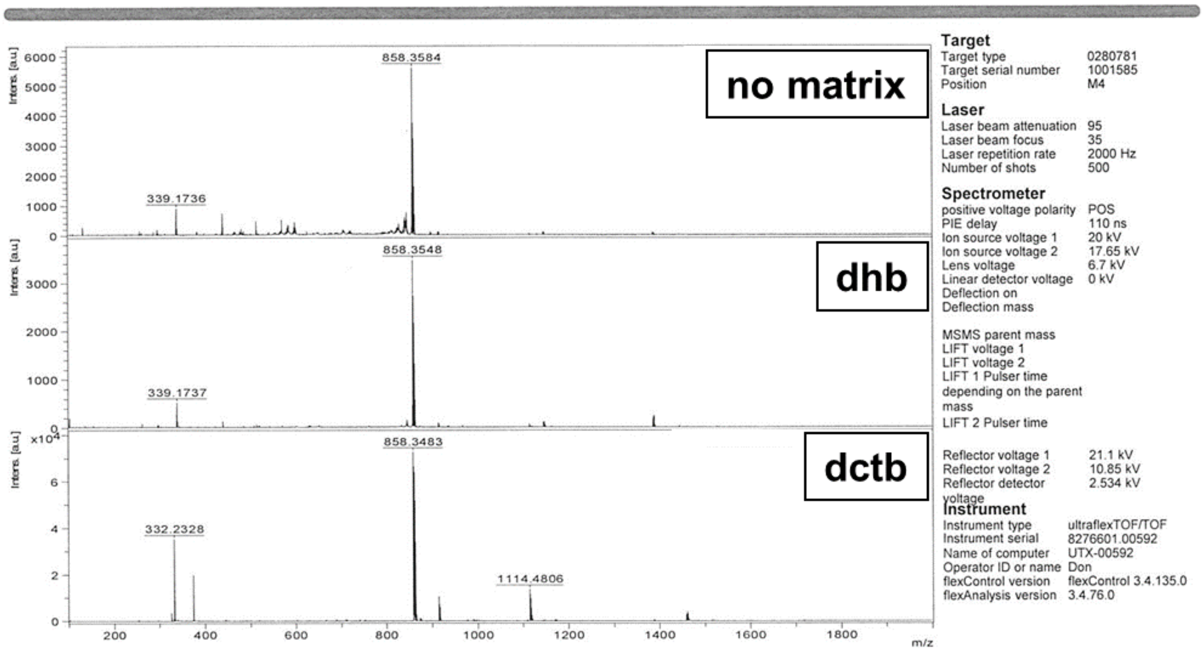

HRMS (MALDI)

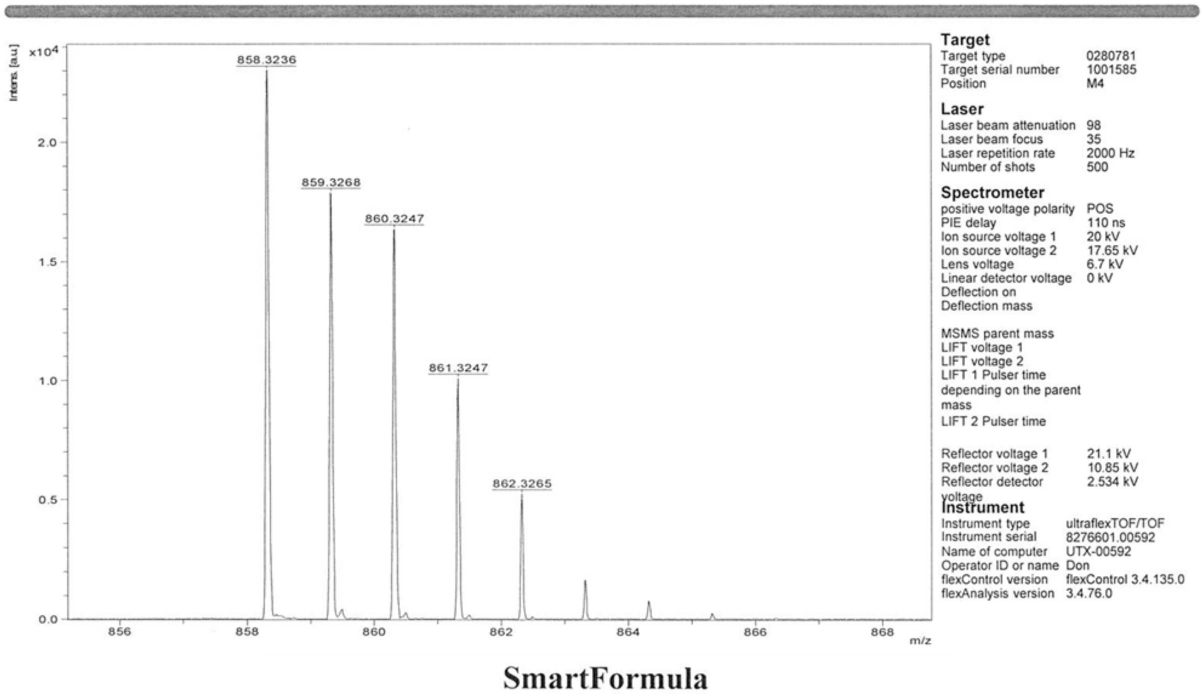

SmartFormula

Formula	Mass	Error	mSigma	DblEq	N rule	Electron Configuration
C 58 H 48 N 4 Ni	858.3227	1.0858	85.1614	37.00	ok	odd

Figure S12. MS/HRMS (MALDI) of **8**.

^{1}H NMR (400 MHz, $CDCl_3$, rt)

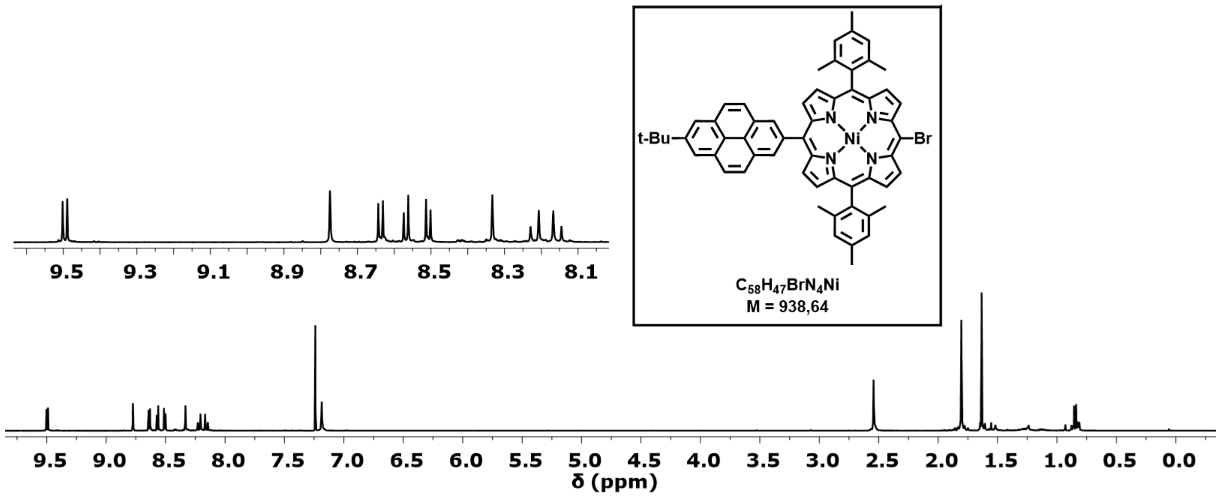

^{13}C NMR (101 MHz, $CDCl_3$, rt)

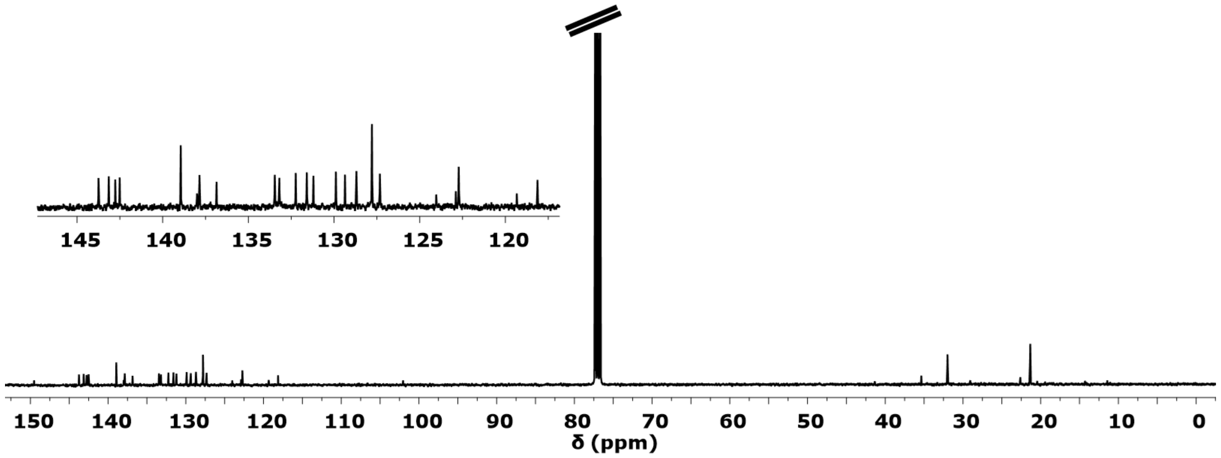

Figure S13. ^{1}H and ^{13}C NMR of **9**.

MS (MALDI)

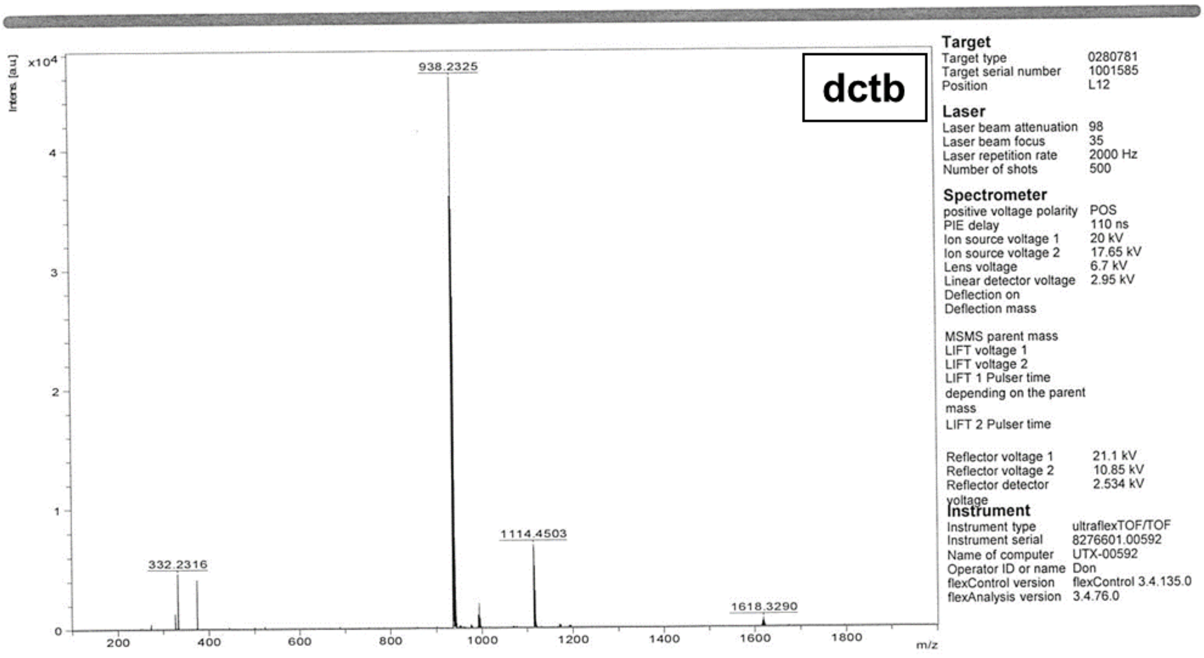

HRMS (MALDI)

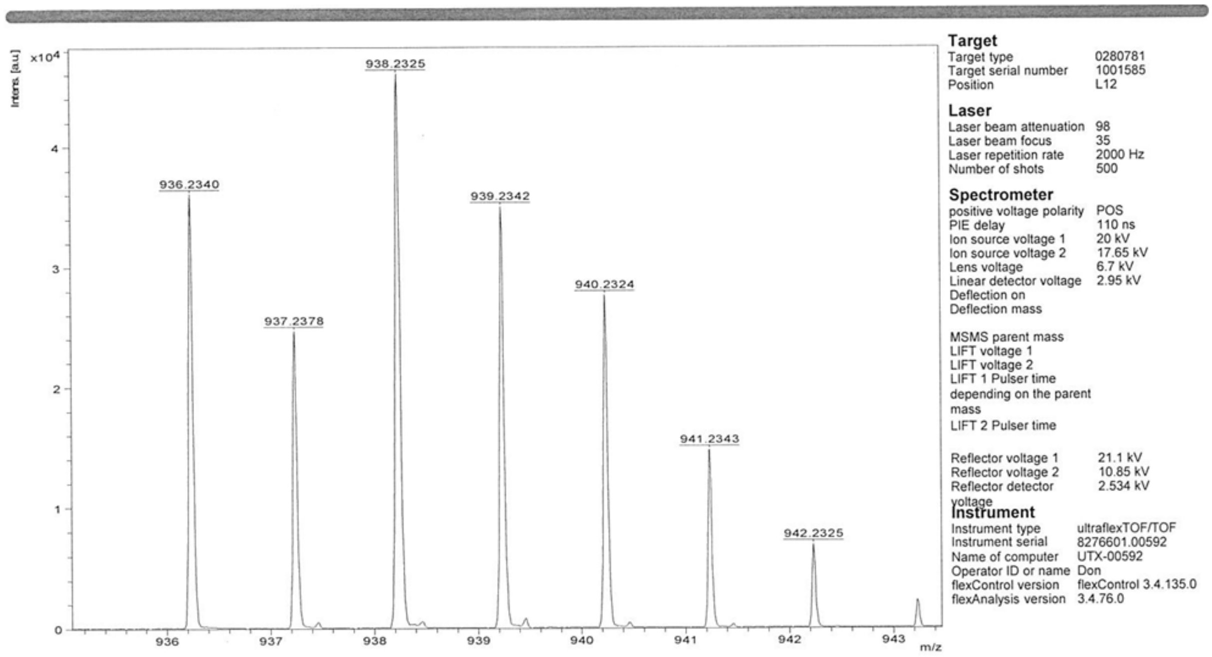

SmartFormula

Formula	Mass	Error	mSigma	DblEq	N rule	Electron Configuration
C 58 H 47 Br N 4 Ni	936.2332	0.8195	96.1846	37.00	ok	odd

Figure S14. MS/HRMS (MALDI) of **9**.

^{1}H NMR (601 MHz, CD_2Cl_2, rt)

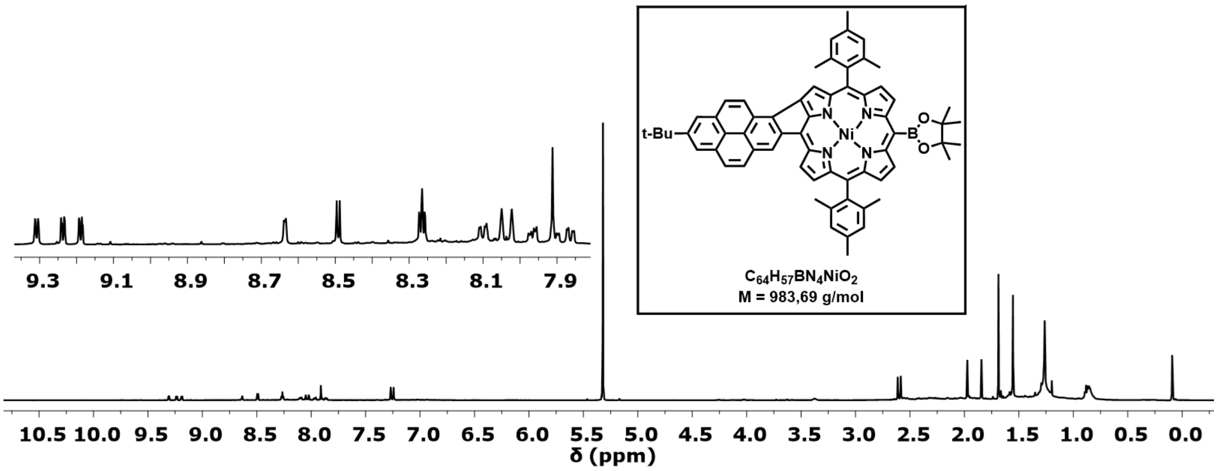

^{13}C NMR - DEPTQ135 (151 MHz, CD_2Cl_2, rt)

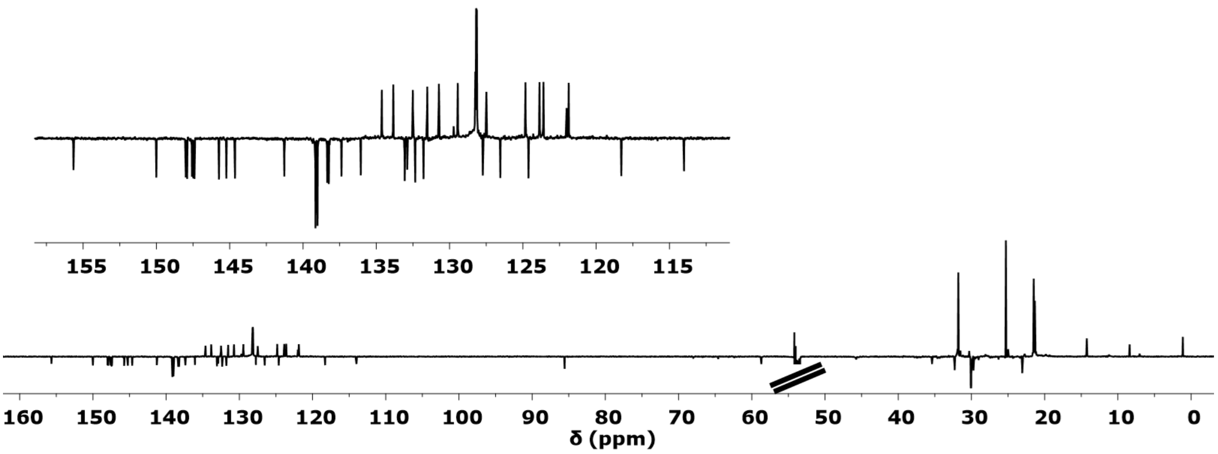

Figure S15. ^{1}H and ^{13}C NMR (DEPTQ135) of **PyrBpin**.

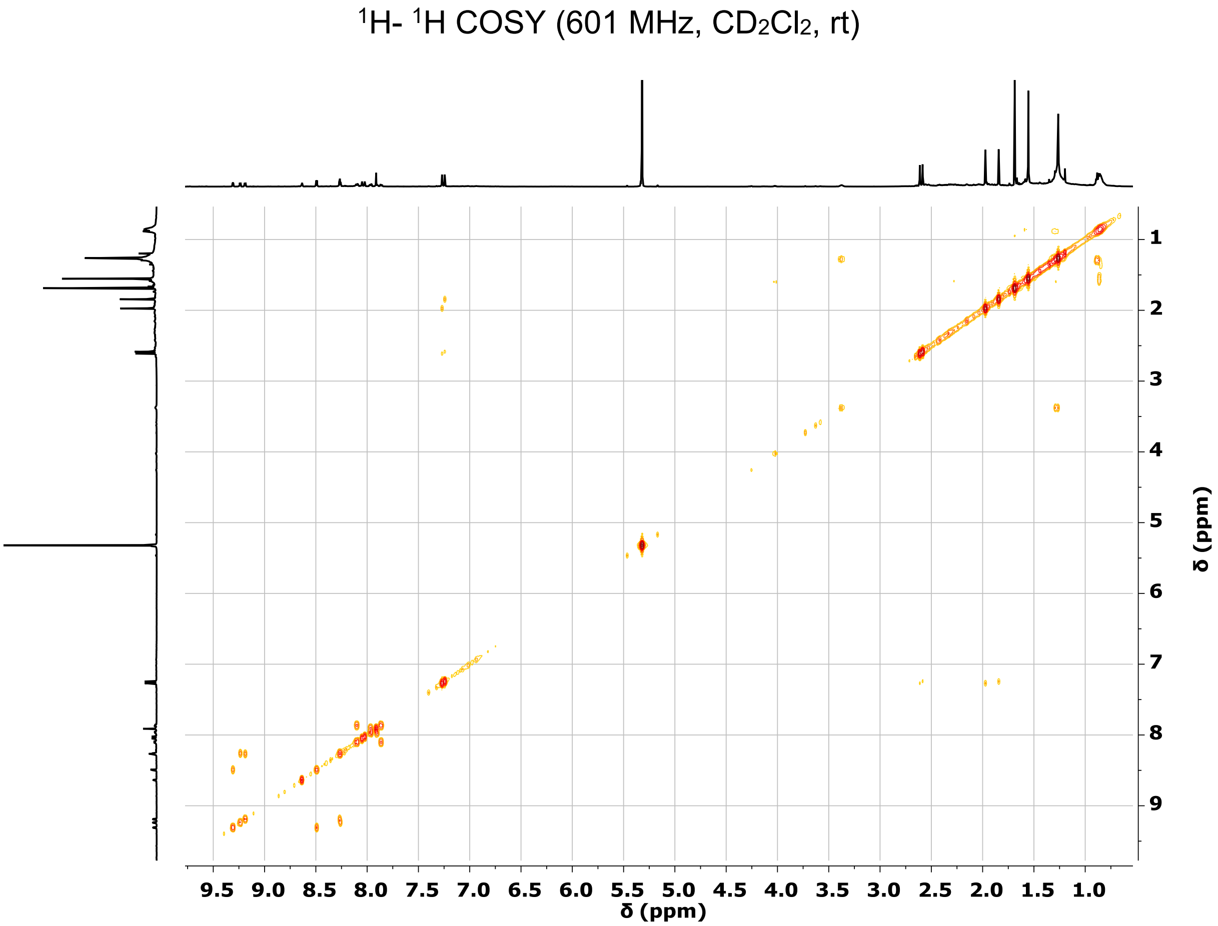

Figure S16. ^{1}H- ^{1}H COSY of **PyrBpin**.

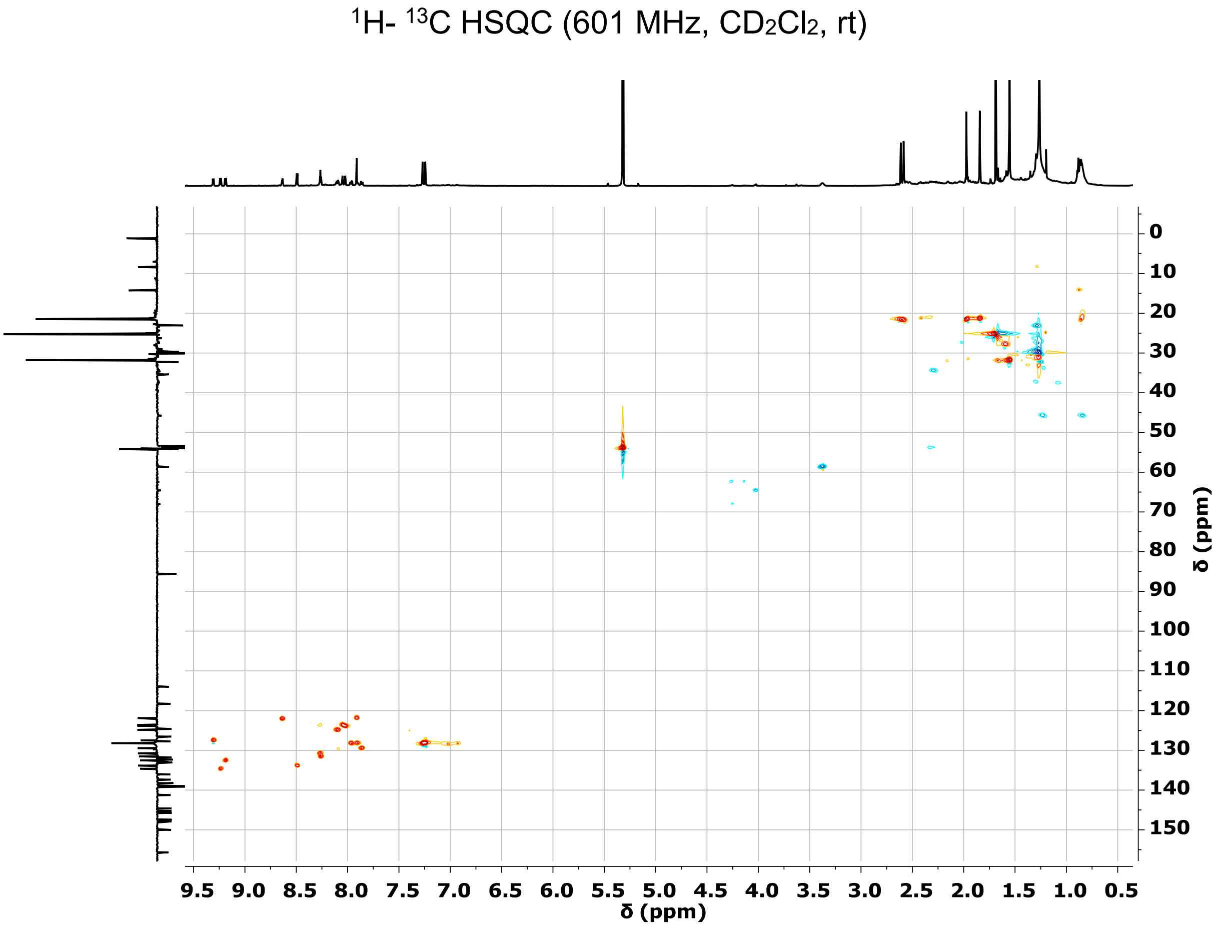

Figure S17. ^{1}H- ^{13}C HSQC of **PyrBpin**.

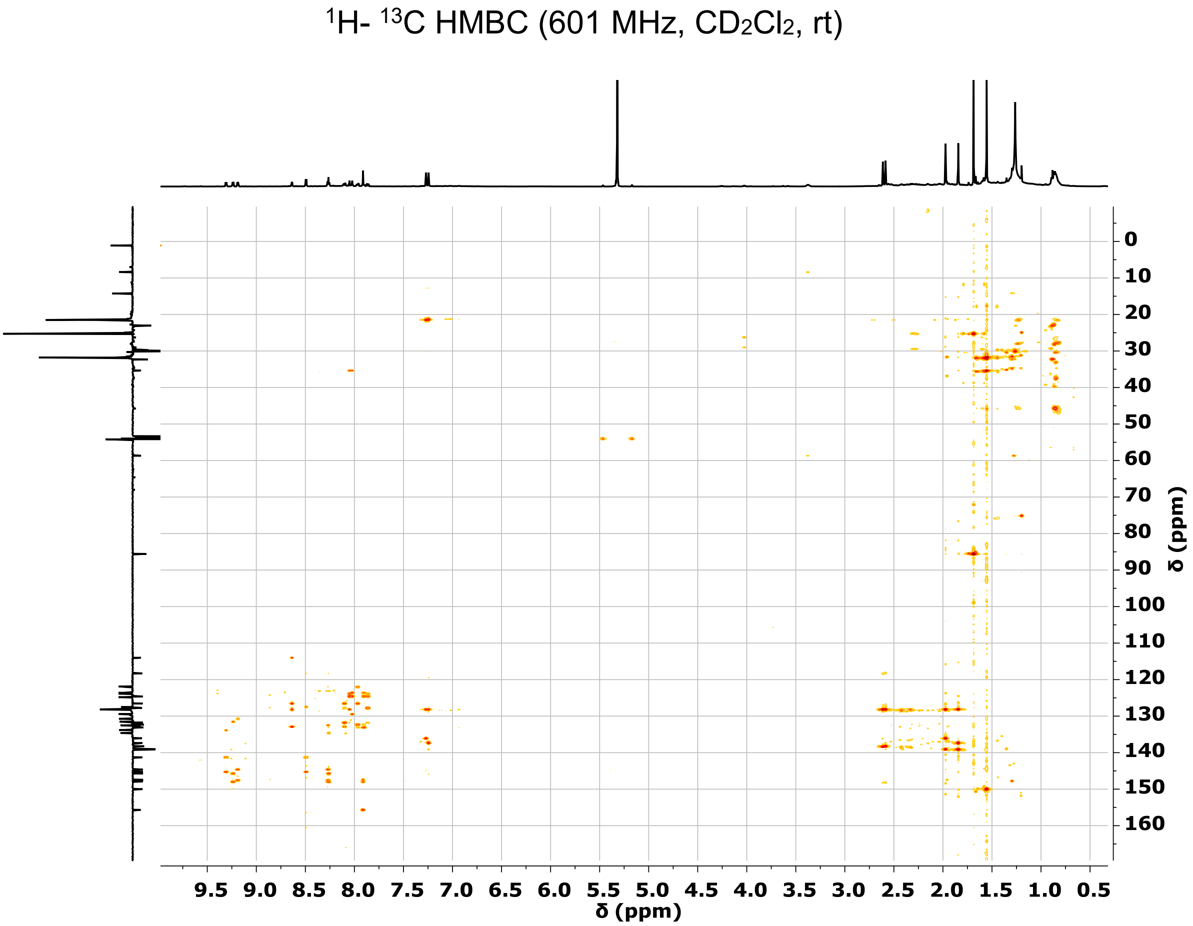

Figure S18. ^{1}H- ^{13}C HMBC of **PyrBpin**.

MS (MALDI)

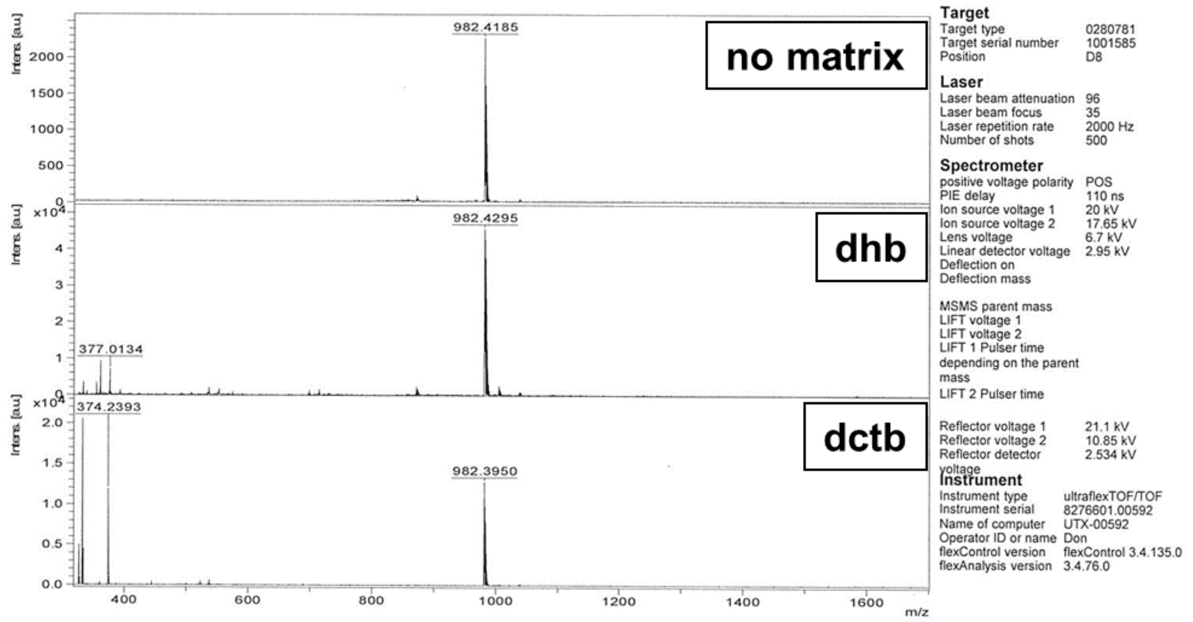

HRMS (MALDI)

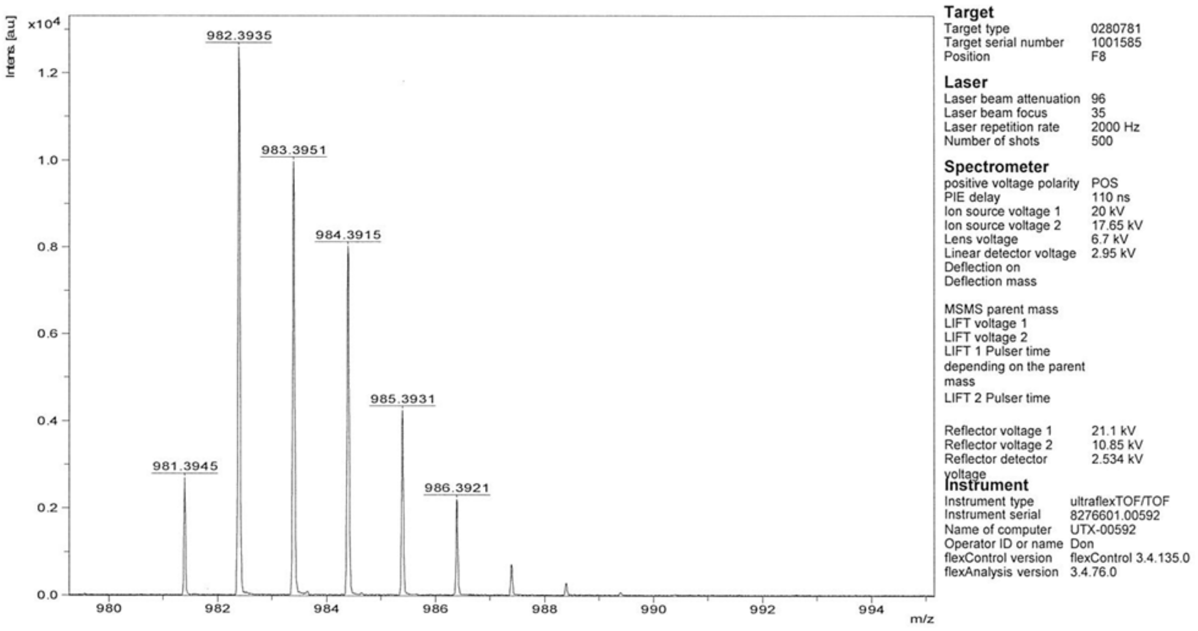

SmartFormula

Formula	Mass	Error	mSigma	DblEq	N rule	Electron Configuration
C 64 H 57 B N 4 Ni O 2	982.3923	1.2436	28.7257	39.00	ok	odd

Figure S19. MS/HRMS (MALDI) of **PyrBpin.**

^{1}H NMR (400 MHz, $CDCl_3$, rt)

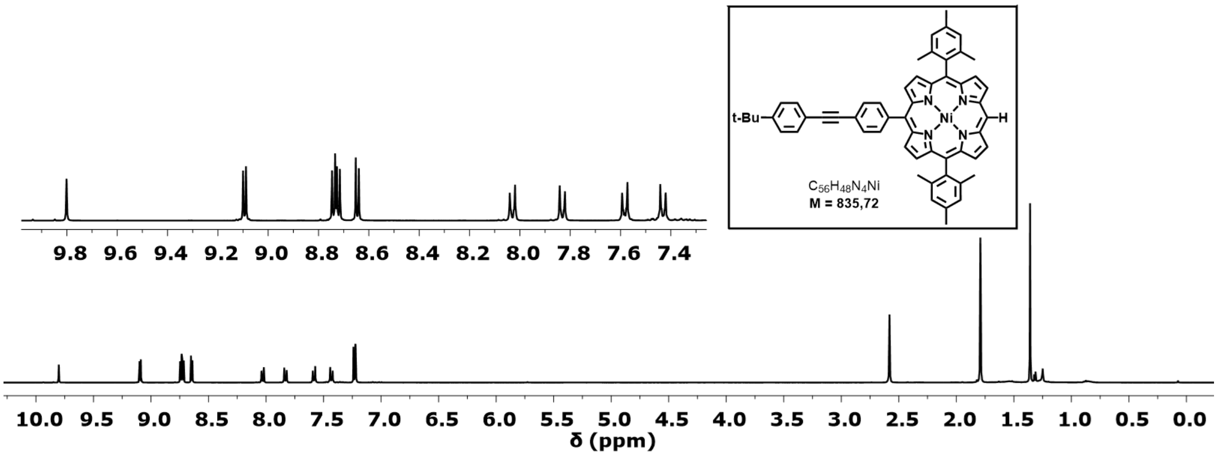

^{13}C NMR (101 MHz, $CDCl_3$, rt)

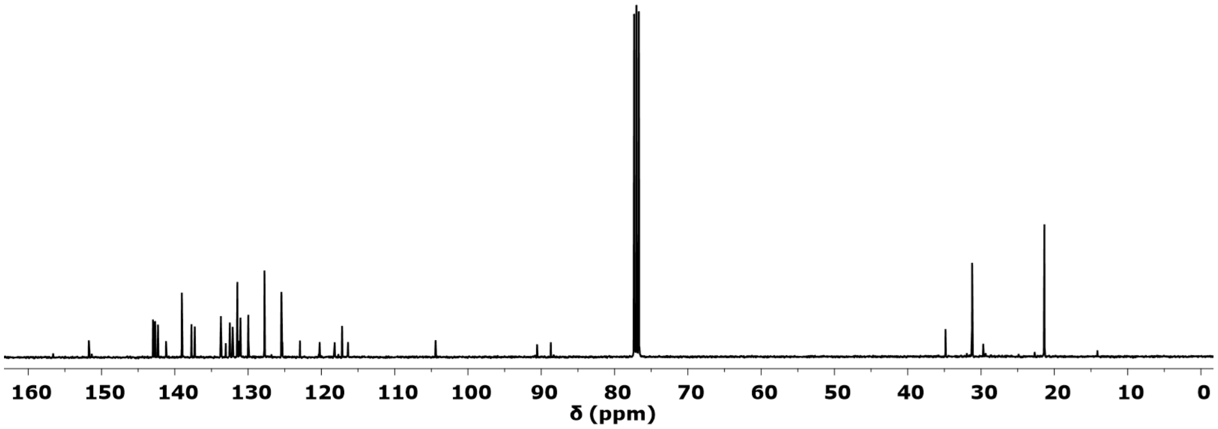

Figure S20. ^{1}H and ^{13}C NMR of **12**.

MS (MALDI)

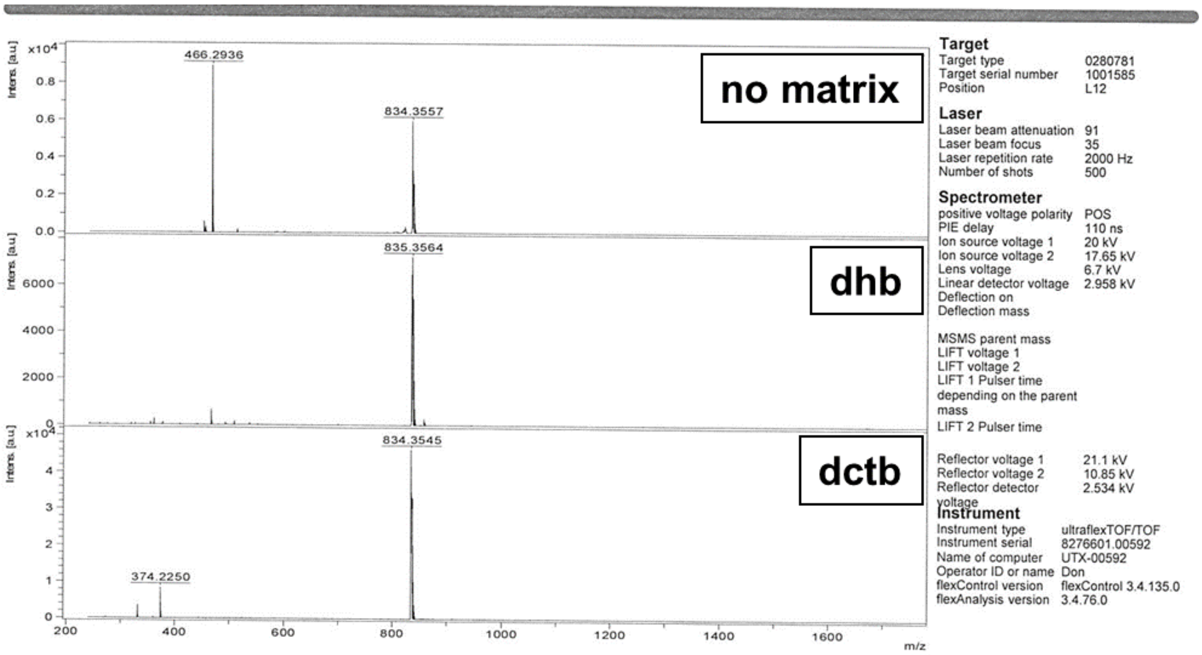

HRMS (MALDI)

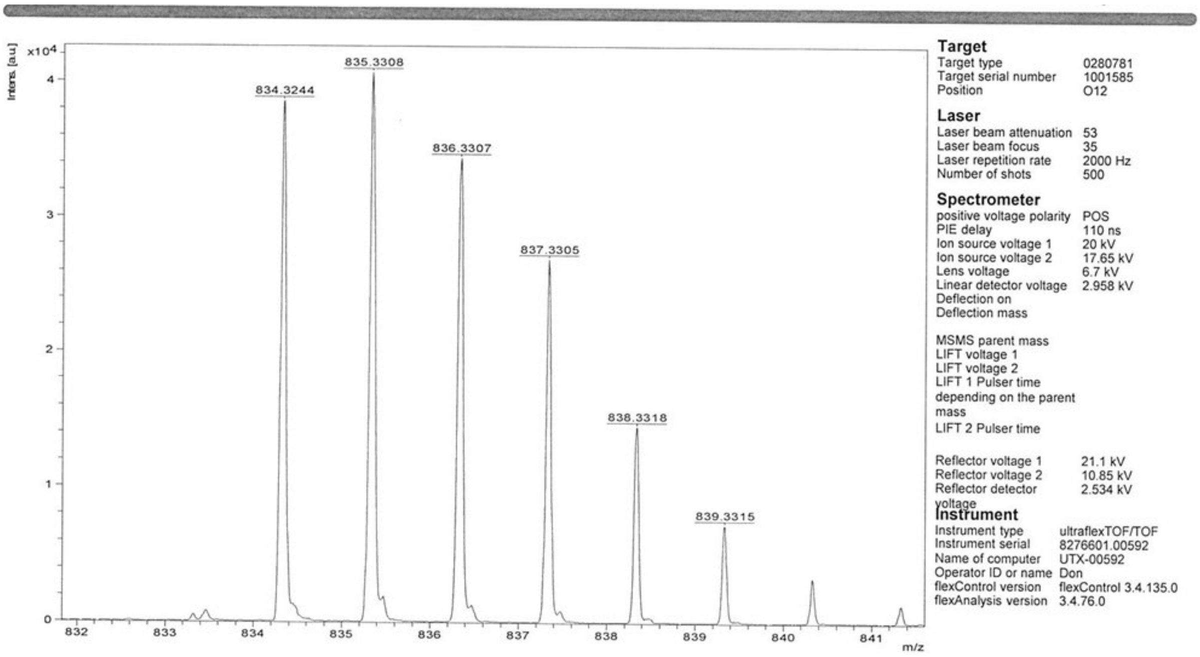

SmartFormula

Formula	Mass	Error	mSigma	DblEq	N rule	Electron Configuration
C 56 H 48 N 4 Ni	834.3227	2.0783	226.0732	35.00	ok	odd

Figure S21. MS/HRMS (MALDI) of **12**.

^{1}H NMR (400 MHz, $CDCl_3$, rt)

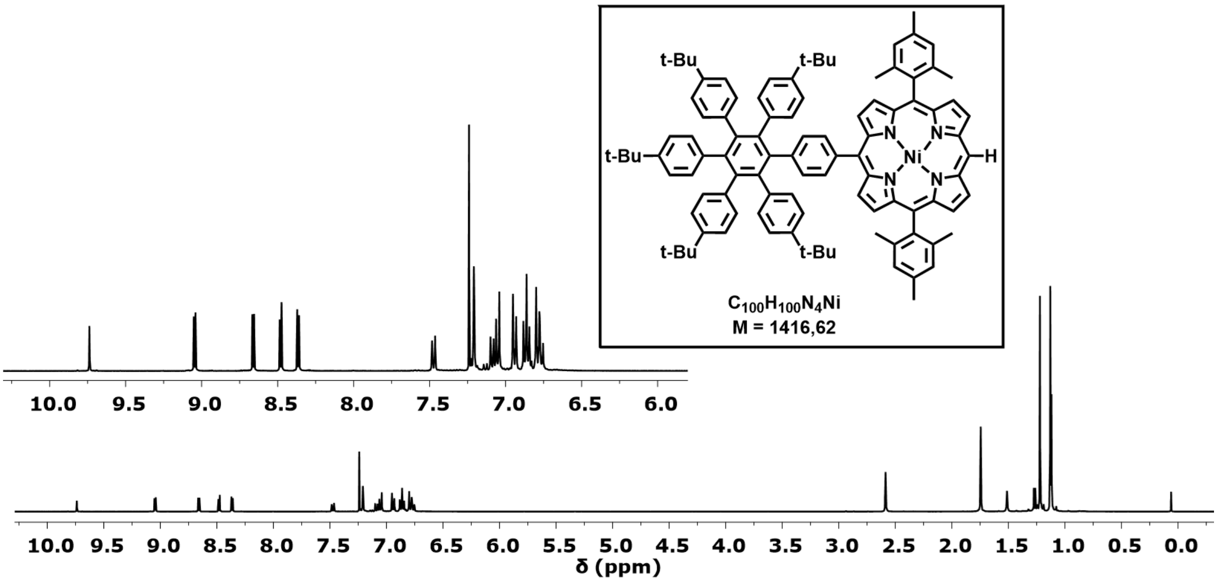

^{13}C NMR (101 MHz, $CDCl_3$, rt)

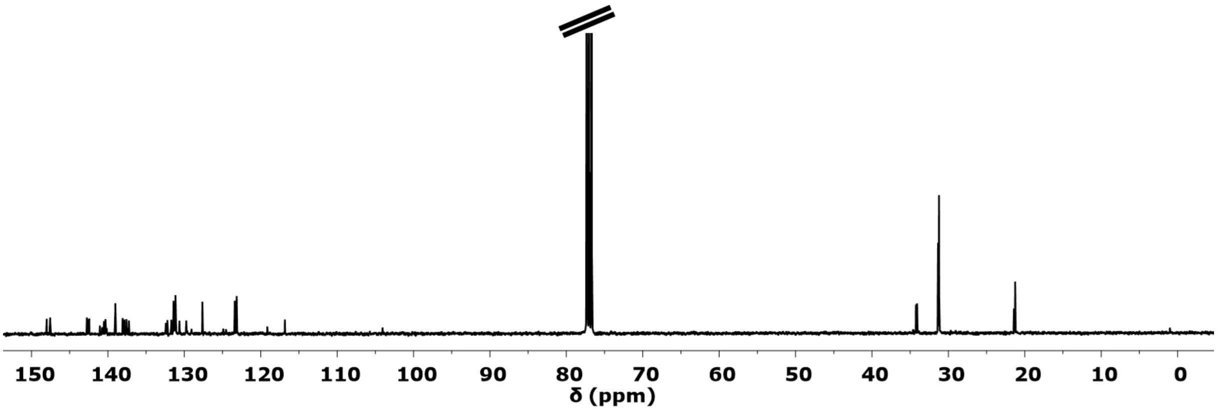

Figure S22. ^{1}H and ^{13}C NMR of **14**.

MS (MALDI)

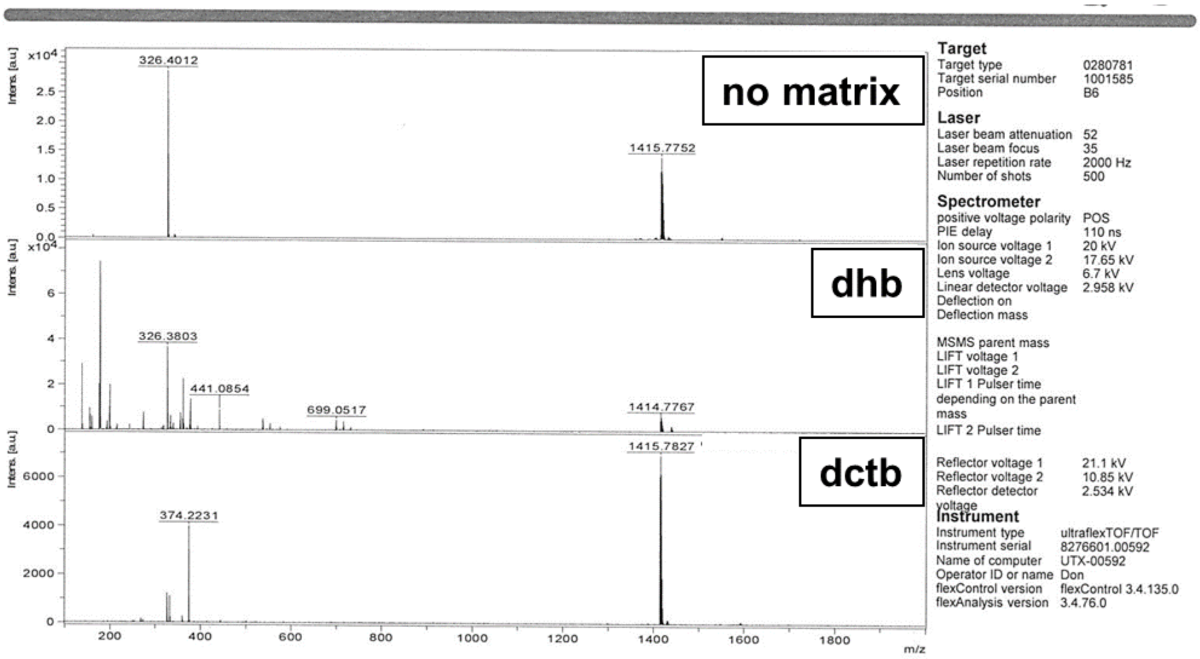

HRMS (MALDI)

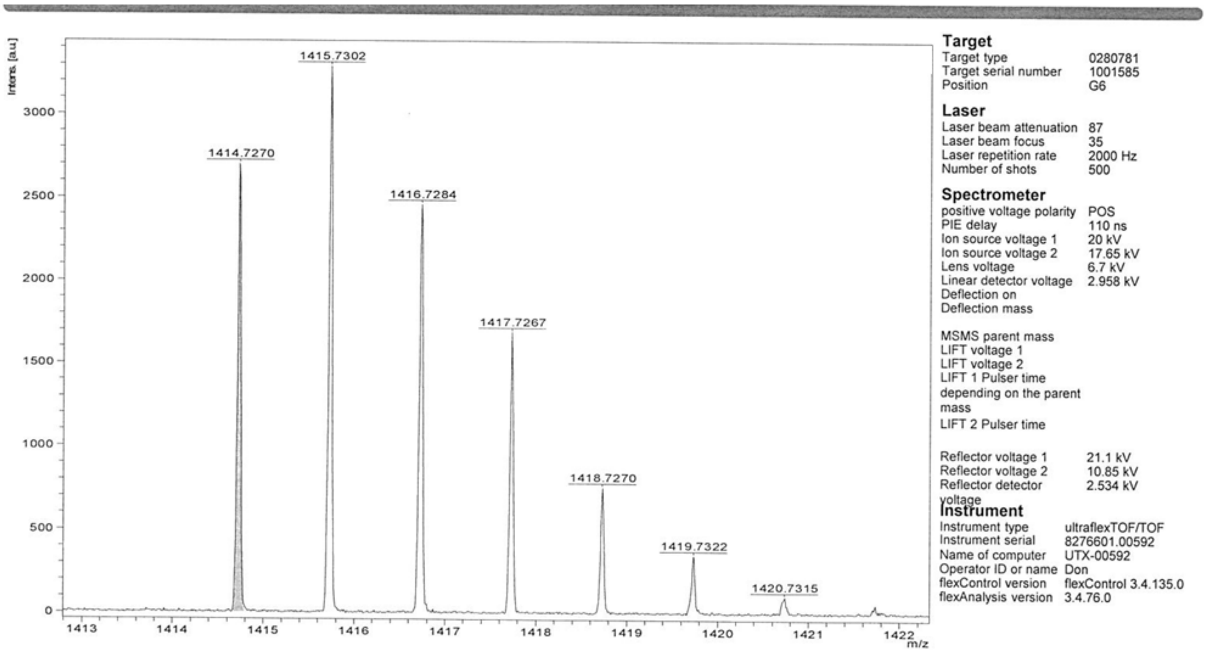

SmartFormula

Formula	Mass	Error	mSigma	DblEq	N rule	Electron Configuration
C 100 H 100 N 4 Ni	1,414.7296	1.8215	74.2823	53.00	ok	odd

Figure S23. MS/HRMS (MALDI) of **14**.

^{1}H NMR (400 MHz, $CDCl_3$, rt)

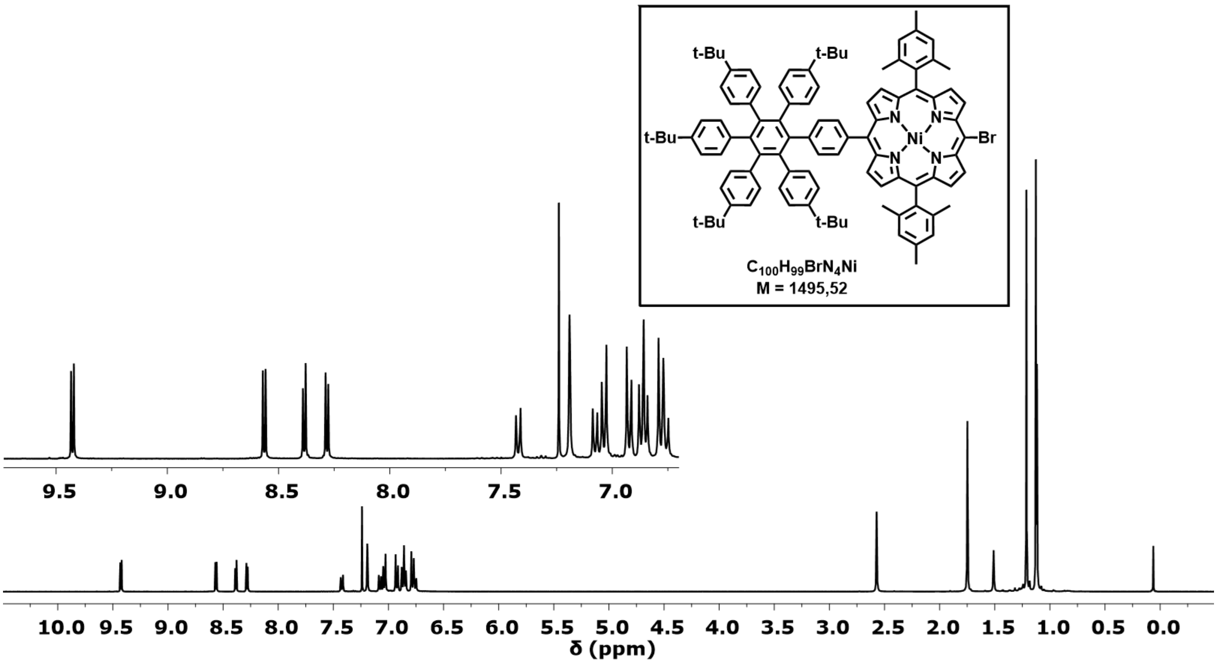

^{13}C NMR (101 MHz, $CDCl_3$, rt)

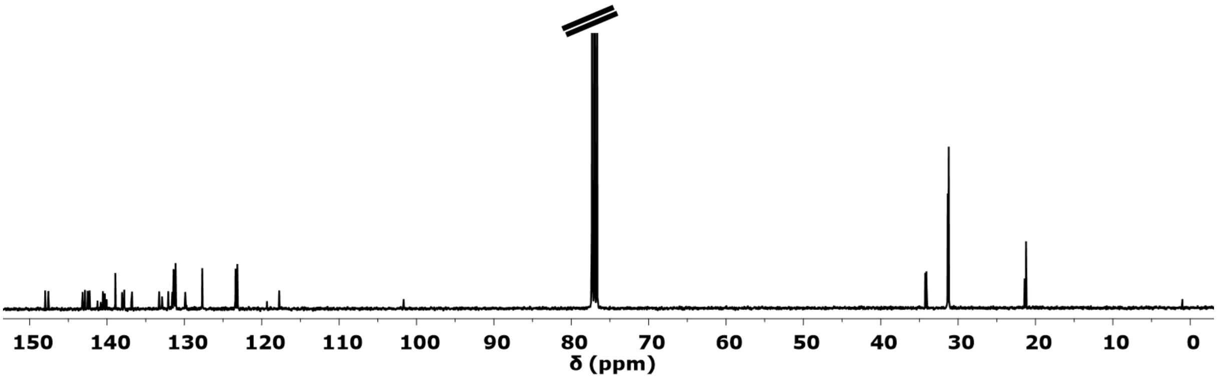

Figure S24. ^{1}H and ^{13}C NMR of **15**.

MS (MALDI)

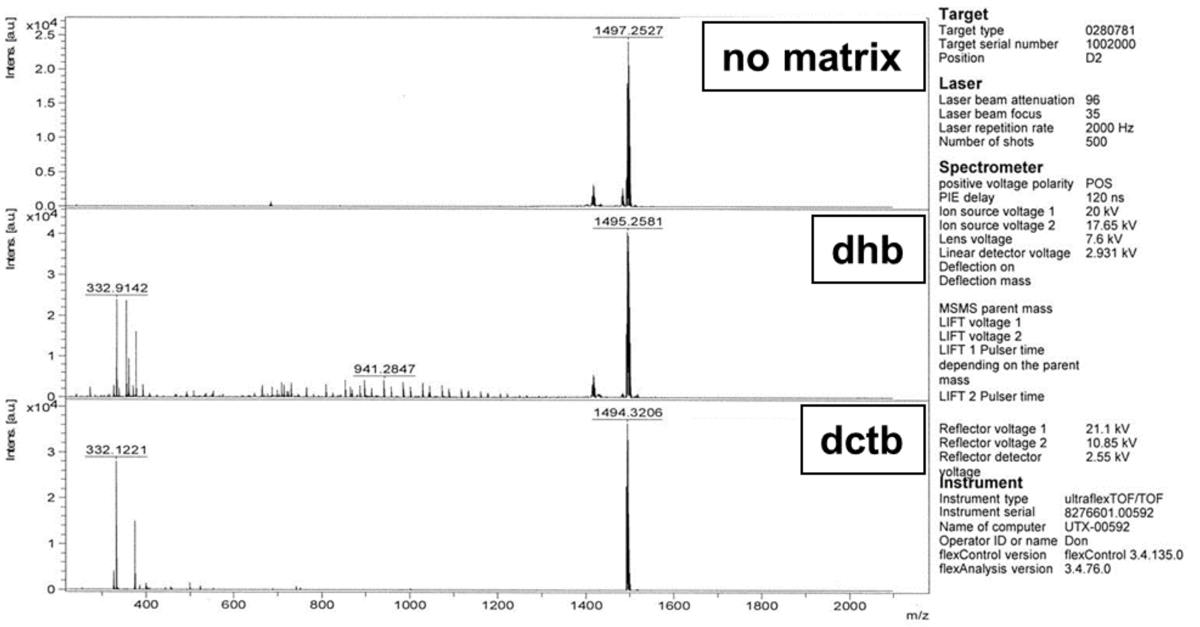

HRMS (MALDI)

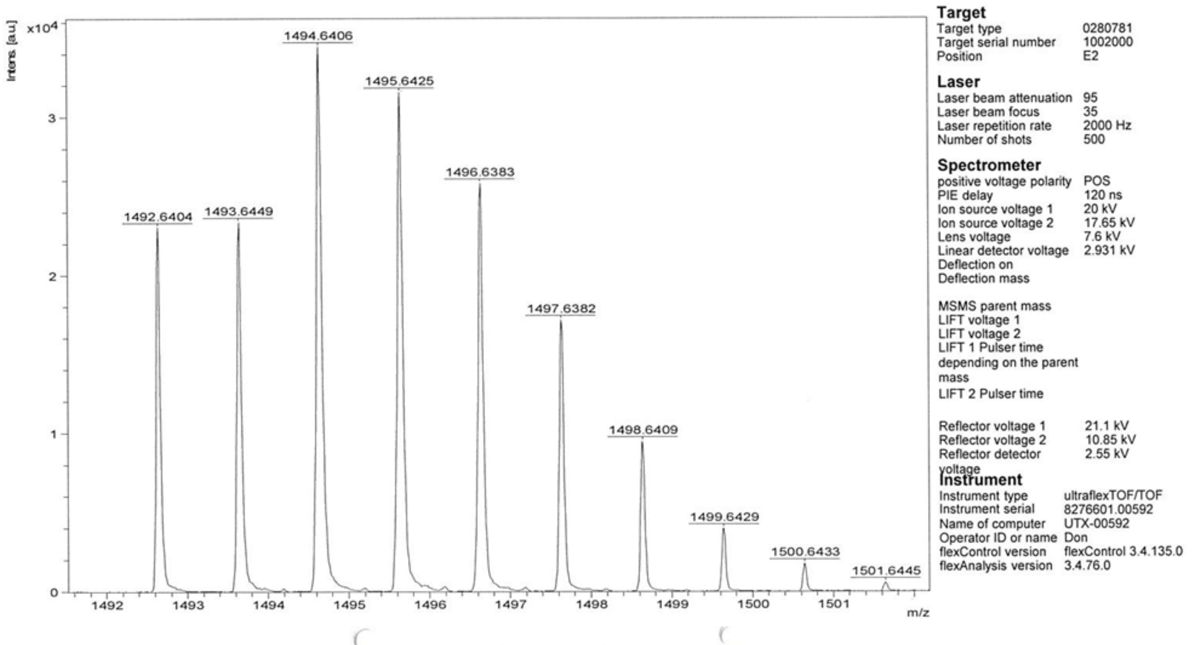

SmartFormula

Formula	Mass	Error	mSigma	DblEq	N rule	Electron Configuration
C 100 H 99 Br N 4 Ni	1,492.6401	0.1725	71.1901	53.00	ok	odd

Figure S25. MS/HRMS (MALDI) of **15**.

^{1}H NMR (601 MHz, CD_2Cl_2, rt)

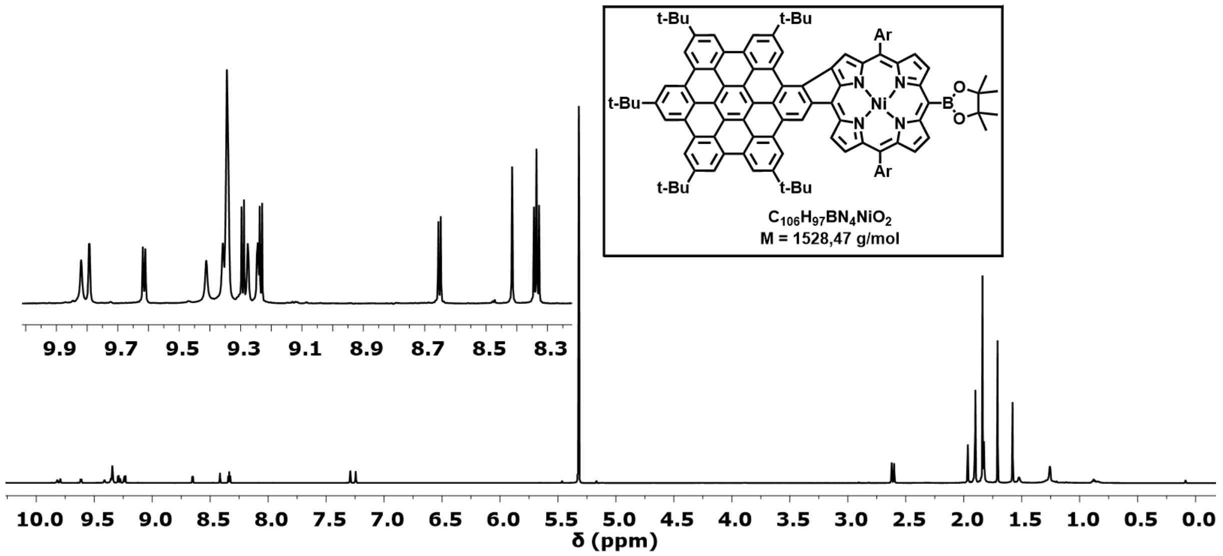

^{13}C NMR - DEPTQ135 (151 MHz, CD_2Cl_2, rt)

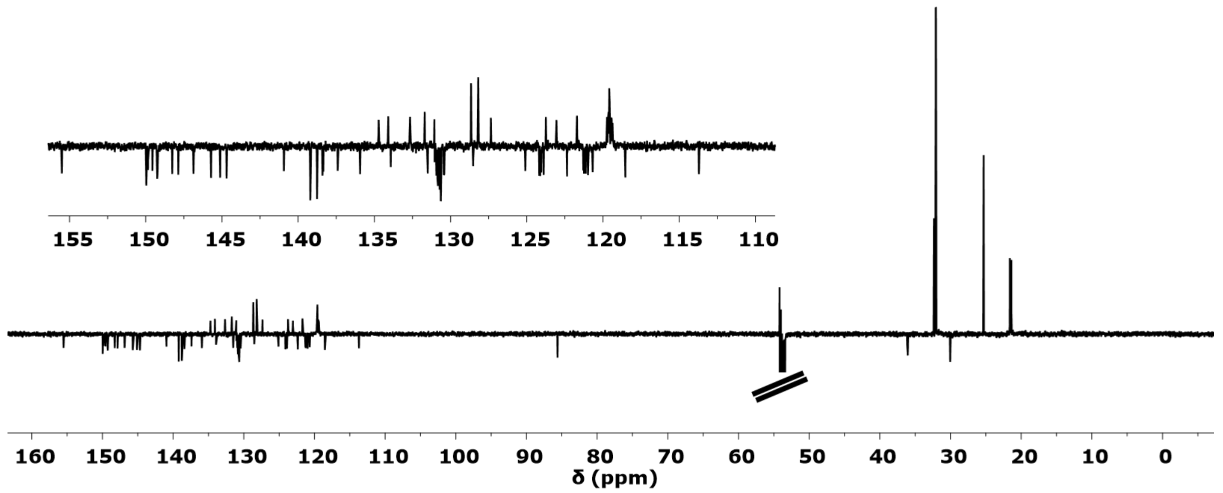

Figure S26. ^{1}H and ^{13}C NMR (DEPTQ135) of **HBCBpin**.

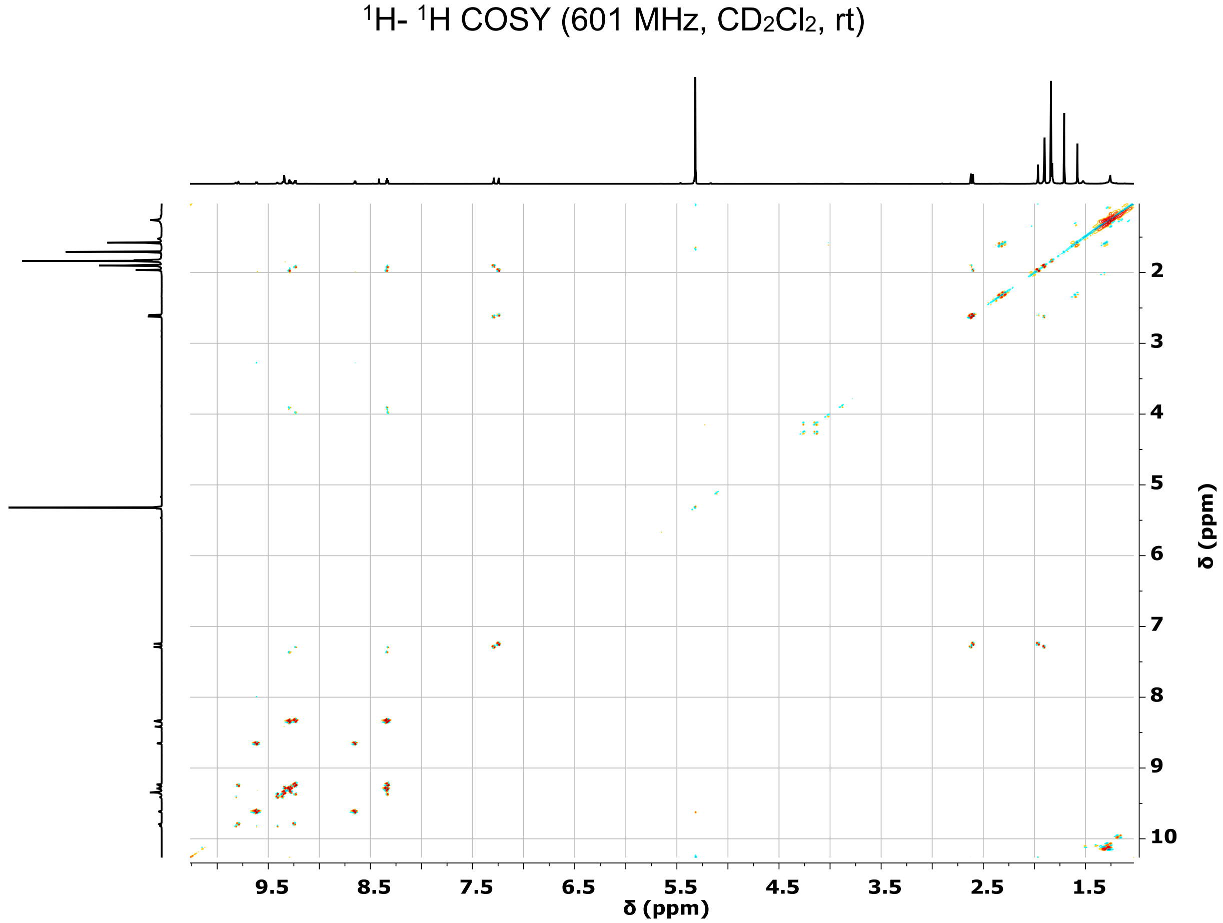

Figure S27. ^{1}H- ^{1}H COSY of **HBCBpin**.

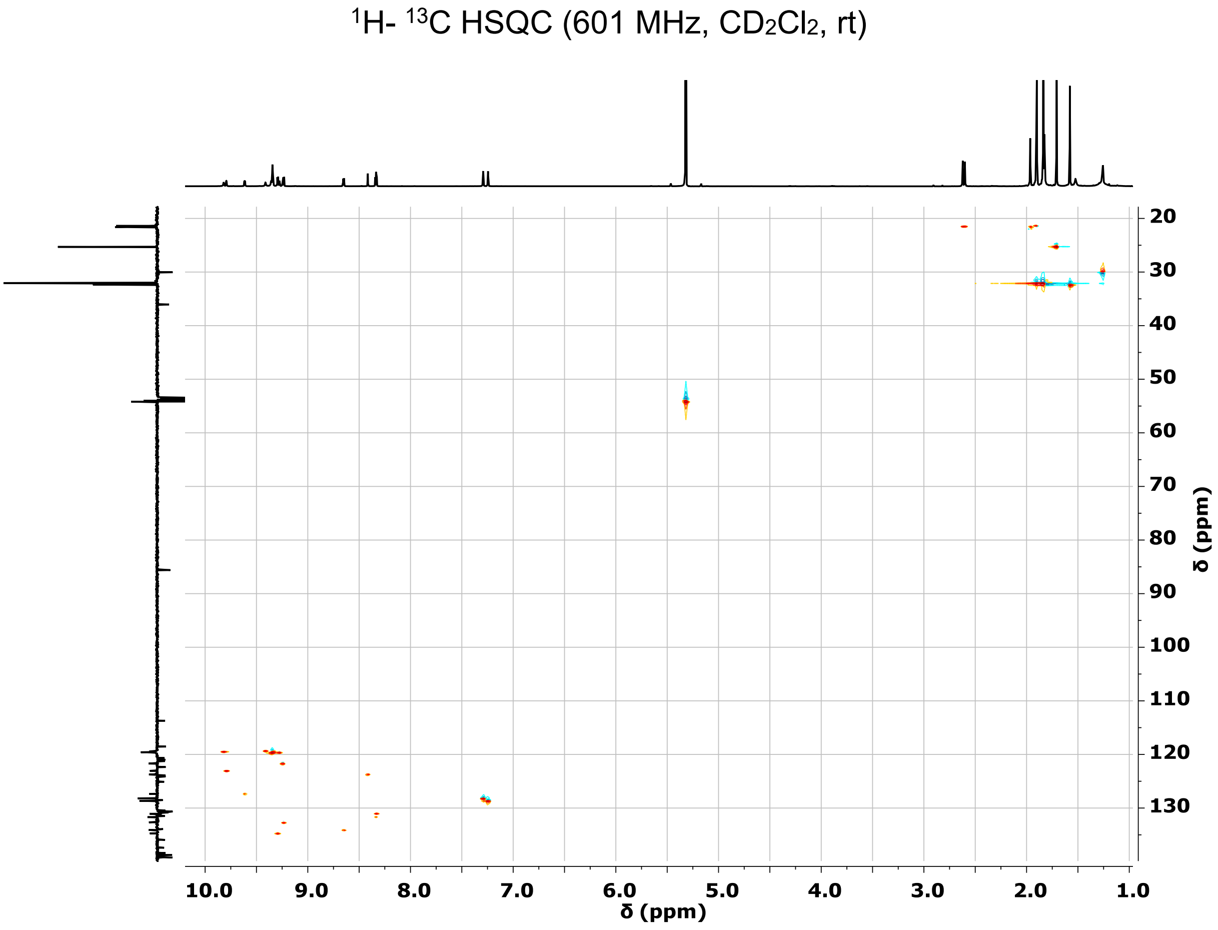

Figure S28. ^{1}H- ^{13}C HSQC of **HBCBpin**.

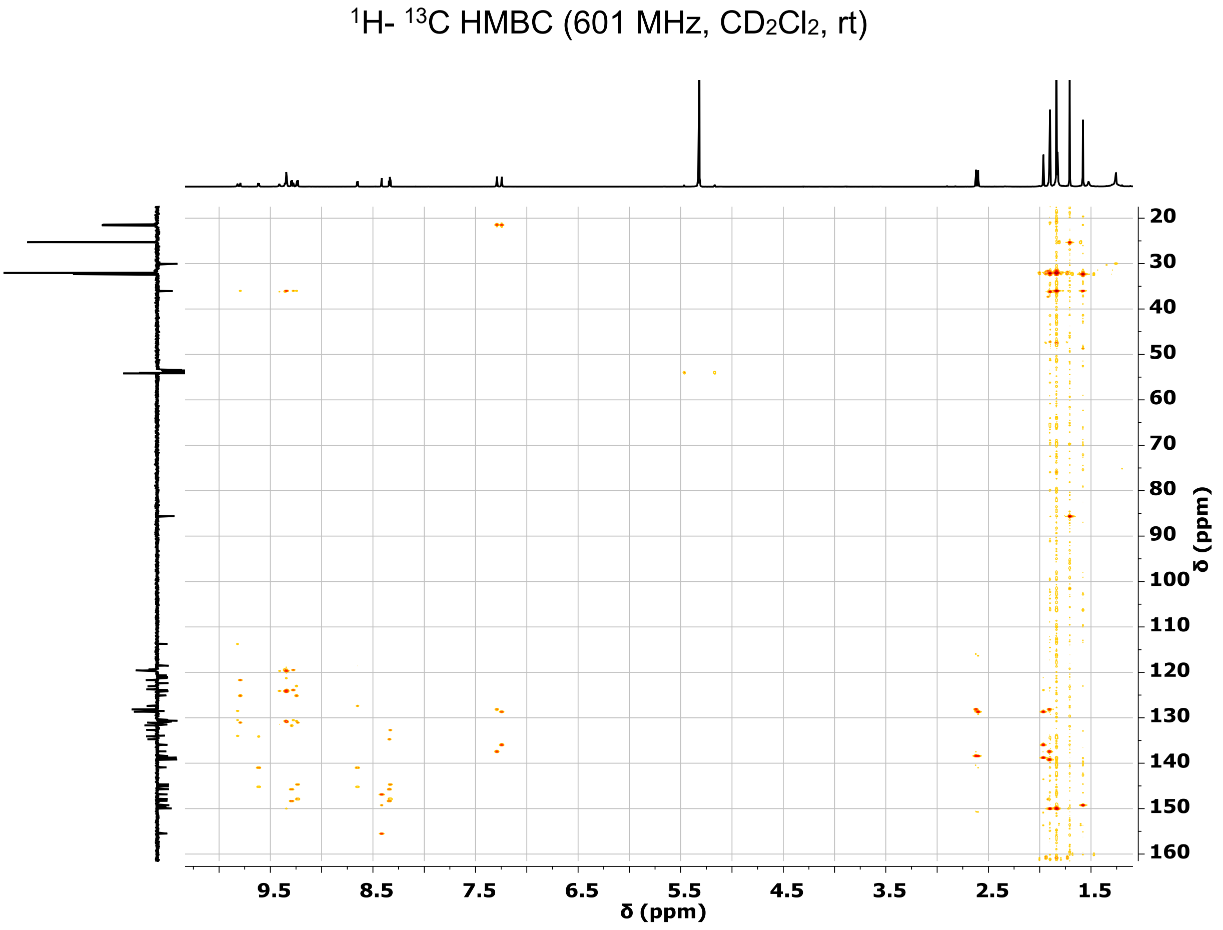

Figure S29. ^{1}H- ^{13}C HMBC of **HBCBpin**.

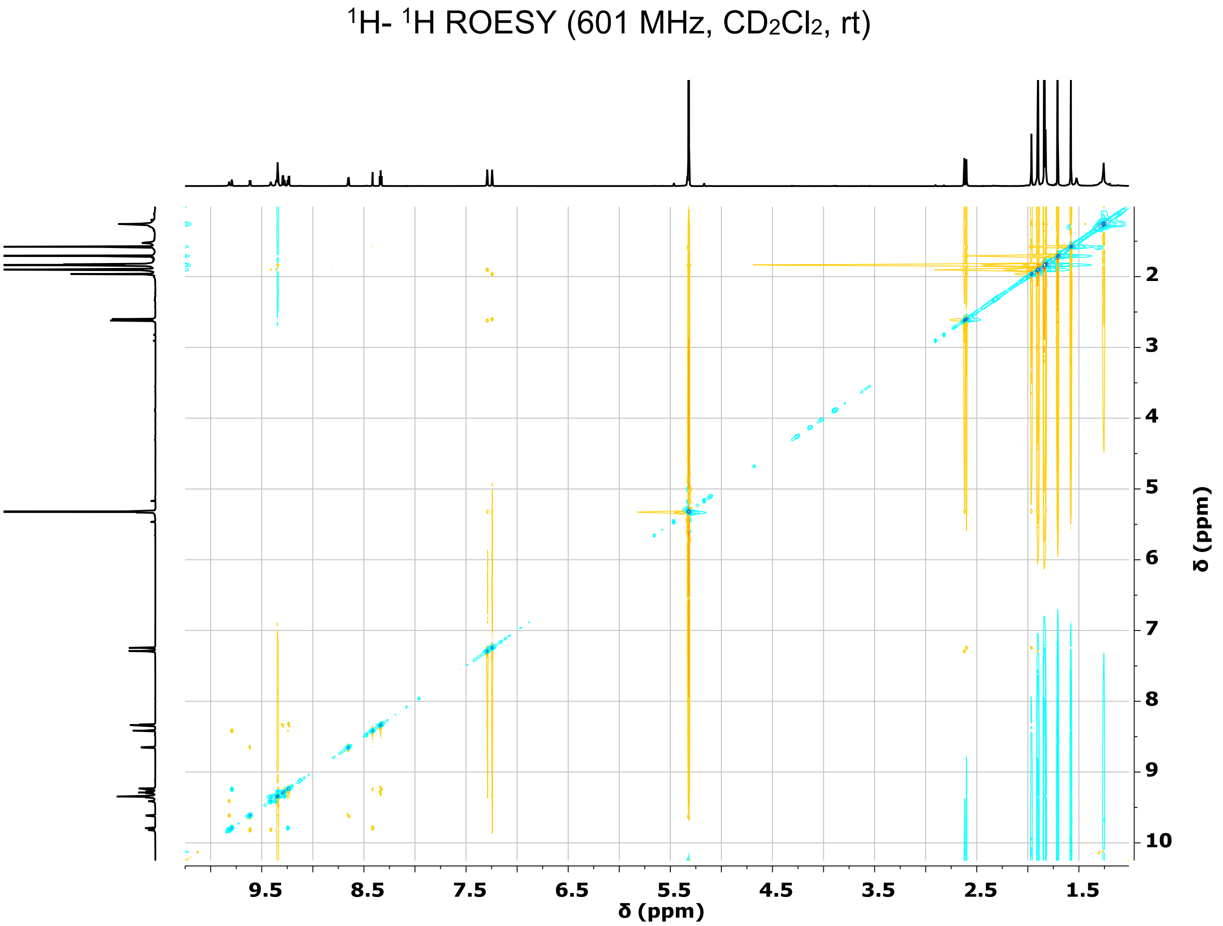

Figure S30. ^{1}H- ^{1}H ROESY of **HBCBpin**.

MS (MALDI)

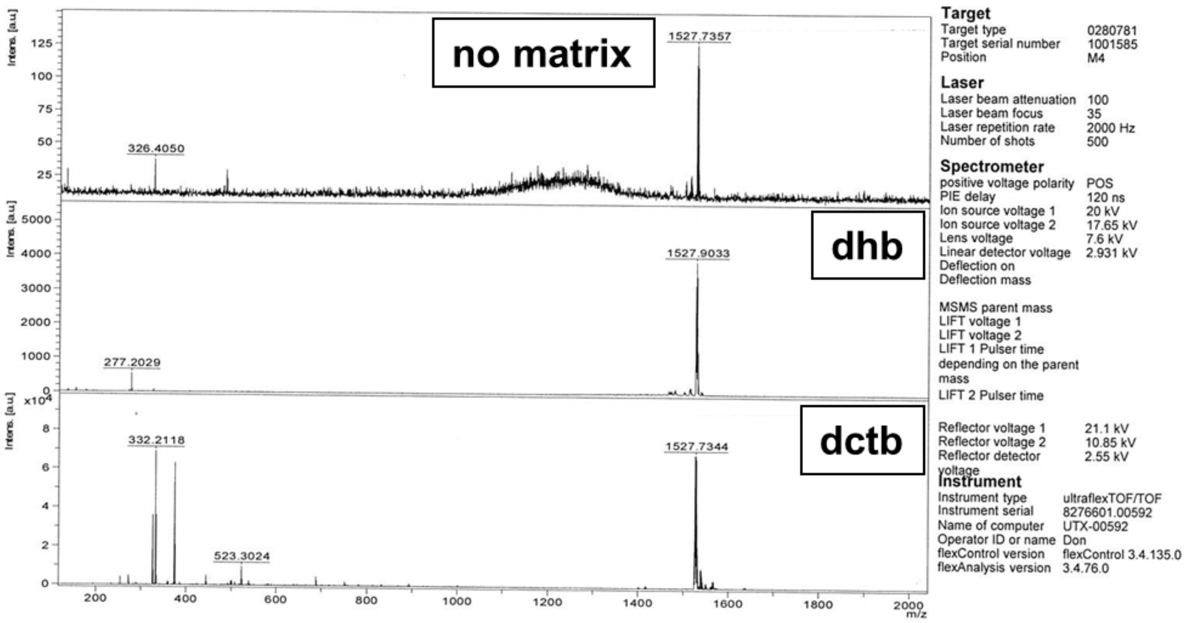

HRMS (MALDI)

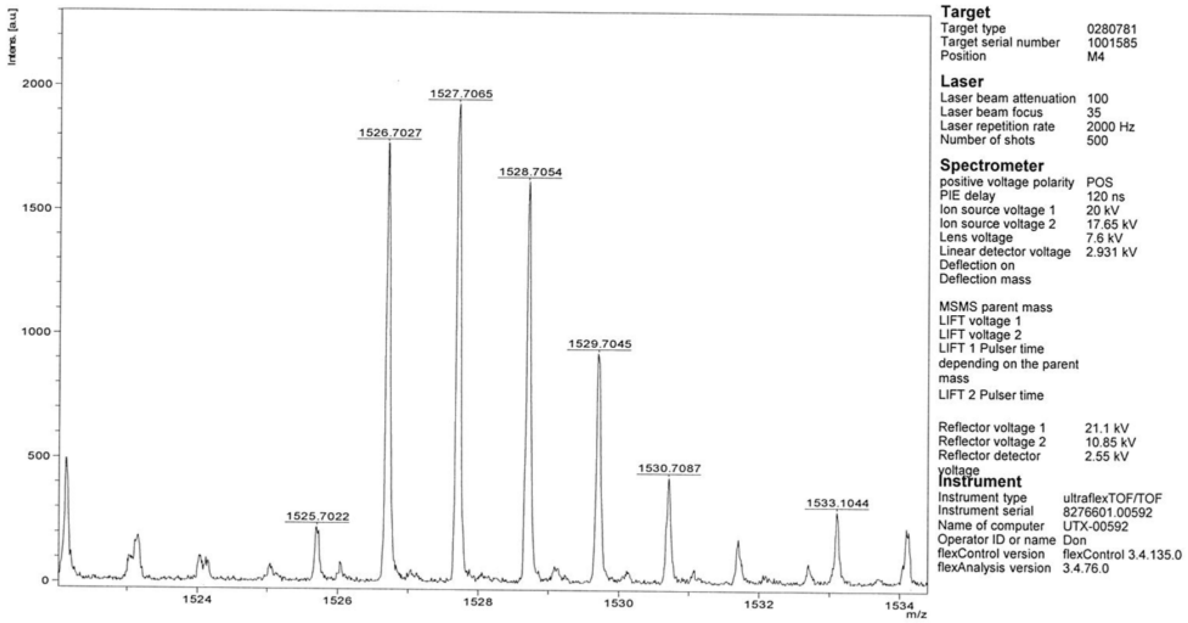

SmartFormula

Formula	Mass	Error	mSigma	DblEq	N rule	Electron Configuration
C 106 H 97 B N 4 Ni O 2	1,526.7053	1.6935	69.4832	61.00	ok	odd

Figure S31. MS/HRMS (MALDI) of **HBCBpin**.

^{1}H NMR (400 MHz, $CDCl_3$, rt)

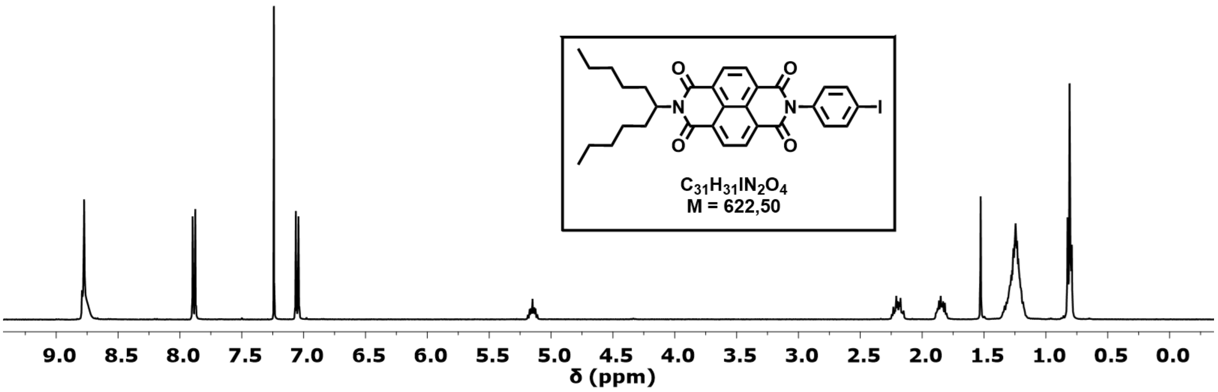

^{13}C NMR (101 MHz, $CDCl_3$, rt)

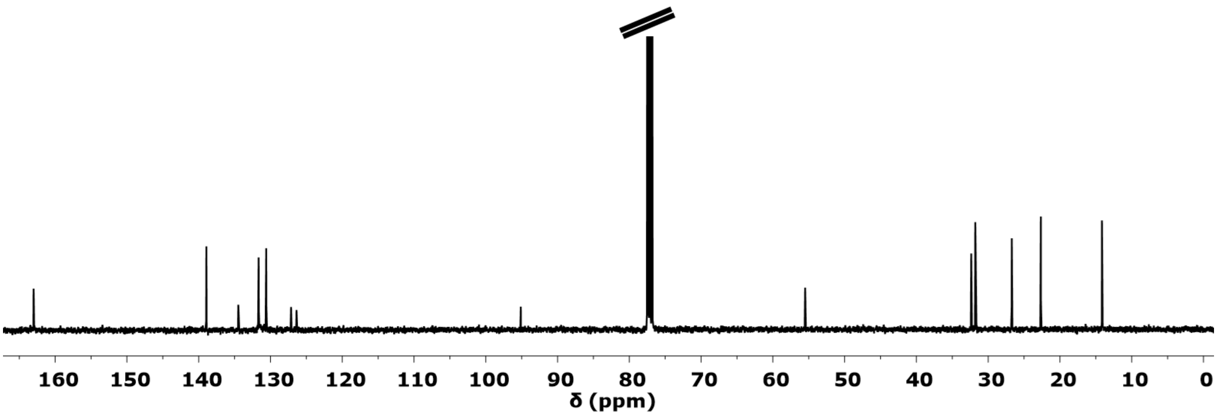

Figure S32. ^{1}H and ^{13}C NMR of **I-NDI**.

HRMS (APPI)

Display Report

Analysis Info

Analysis Name	D:\Data\2024\Hirsch -2024\Schulze-ES 132-test.d	Acquisition Date	6/4/2024 10:12:31 AM
Method	APPI_pos_low_t3.d.m	Operator	MD
Sample Name	Low Concentration Tunemix	Instrument	maXis 288882.20183
Comment	CH2Cl2		

Acquisition Parameter

Source Type	APPI	Ion Polarity	Positive	Set Nebulizer	5.2 Bar		
Focus	Not active	Set Capillary	700 V	Set Dry Heater	220 °C		
Scan Begin	80 m/z	Set End Plate Offset	-500 V	Set Dry Gas	1.2 l/min		
Scan End	1550 m/z	Set Charging Voltage	0 V	Set Divert Valve	Waste		
		Set Corona	0 nA	Set APCI Heater	250 °C		

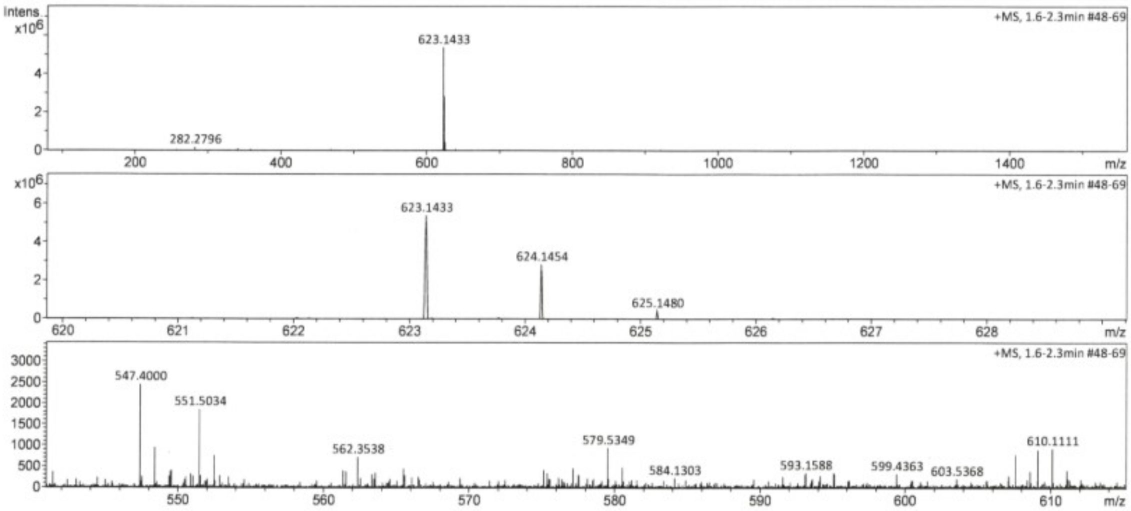

Schulze-ES 132-test.d
Bruker Compass DataAnalysis 4.2 printed: 6/4/2024 10:17:11 AM by: MD Page 1 of 1

Figure S33. MS/HRMS (APPI) of **I-NDI**.

^{1}H NMR (400 MHz, DMSO-d6, rt)

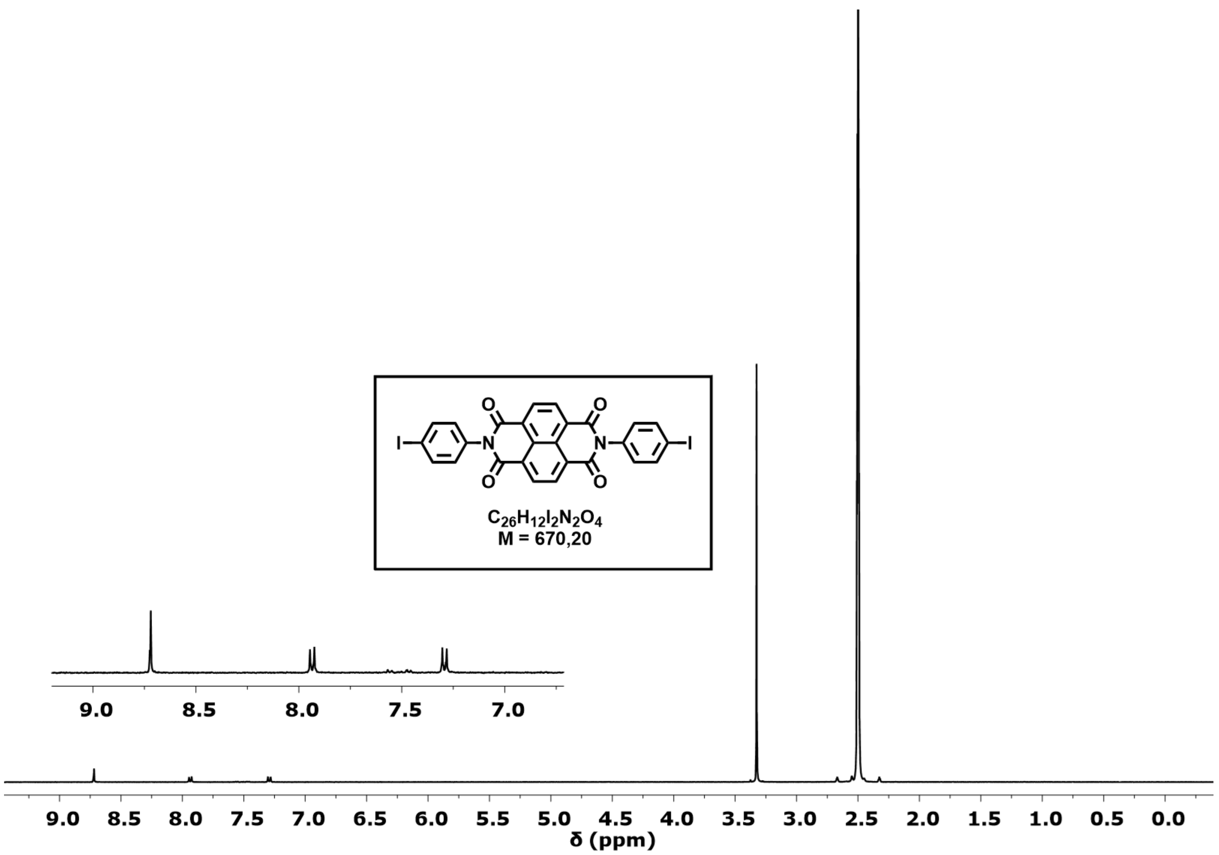

Figure S34. ^{1}H NMR of **I-NDI-I**.

HRMS (APPI)

Display Report

Analysis Info

Analysis Name	D:\Data\2024\Hirsch-2024\Schulze-KS-4-test--appi-.d	Acquisition Date	6/4/2024 3:04:55 PM
Method	APPI_pos_low_t3.d.m	Operator	MD
Sample Name	Low Concentration Tunemix	Instrument	maXis 288882.20183
Comment	CH2Cl2		

Acquisition Parameter

Source Type	APPI	Ion Polarity	Positive	Set Nebulizer	5.2 Bar
Focus	Not active	Set Capillary	700 V	Set Dry Heater	220 °C
Scan Begin	80 m/z	Set End Plate Offset	-500 V	Set Dry Gas	1.2 l/min
Scan End	1550 m/z	Set Charging Voltage	0 V	Set Divert Valve	Waste
		Set Corona	0 nA	Set APCI Heater	250 °C

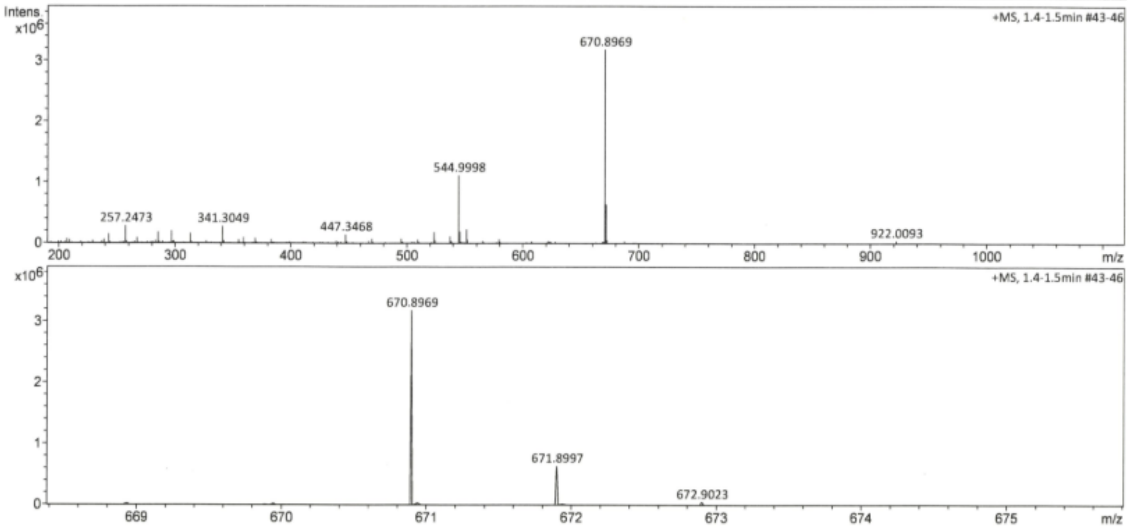

Schulze-KS-4-test--appi-.d
Bruker Compass DataAnalysis 4.2 printed: 6/4/2024 3:08:45 PM by: MD Page 1 of 1

Figure S35. MS/HRMS (APPI) of **I-NDI-I**.

^{1}H NMR (400 MHz, $CDCl_3$, rt)

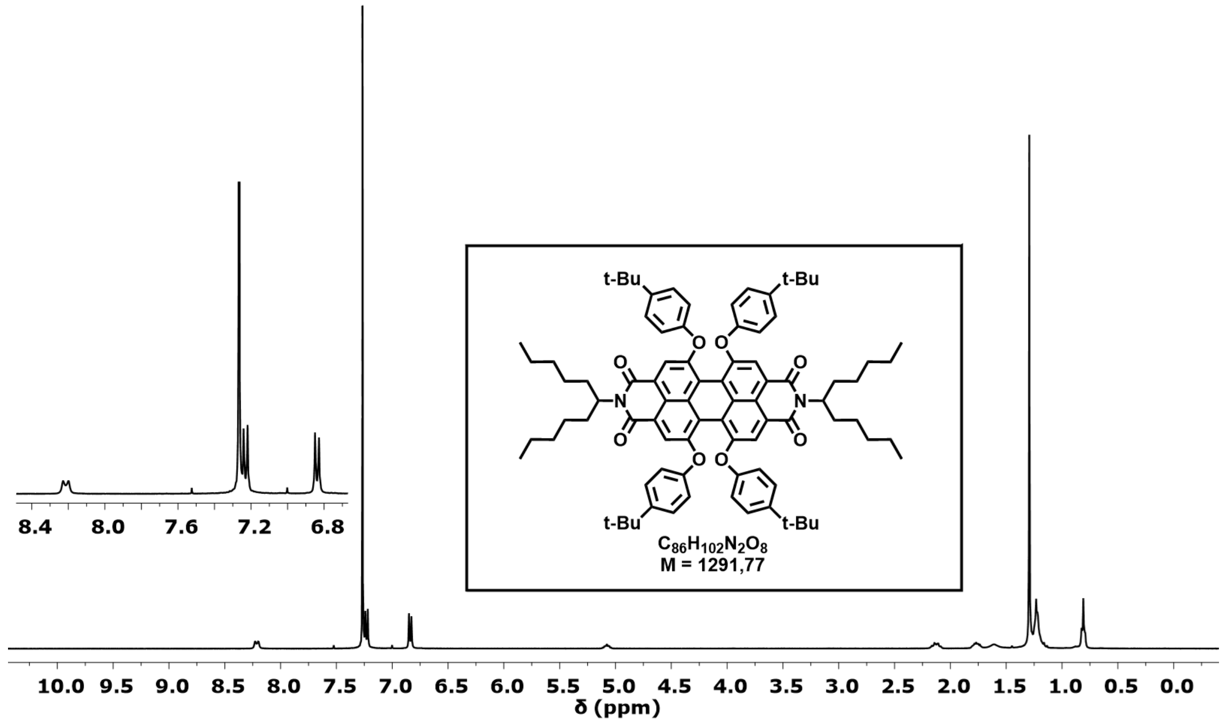

^{13}C NMR (101 MHz, $CDCl_3$, rt)

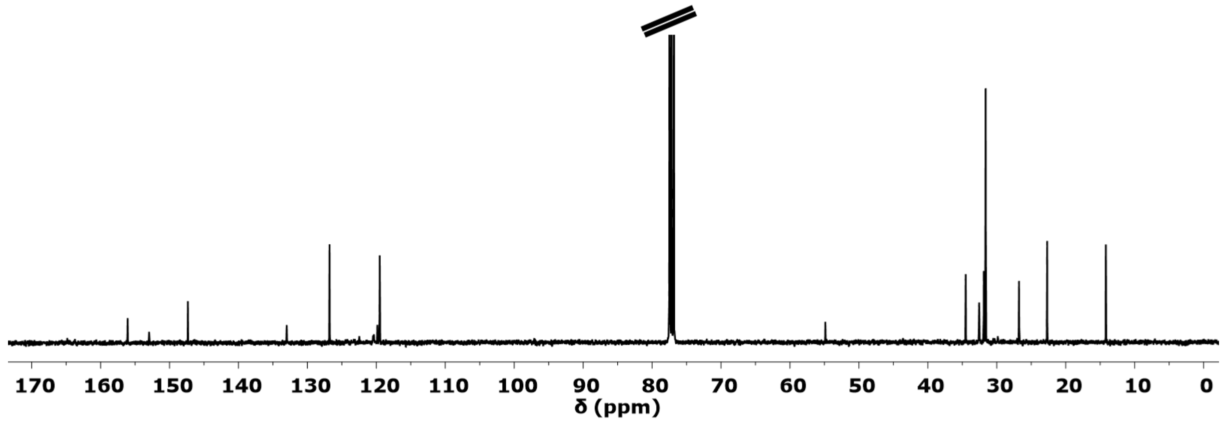

Figure S36. ^{1}H and ^{13}C NMR of **22**.

HRMS (APPI)

Display Report

Analysis Info

Analysis Name	D:\Data\2024\Hirsch-2024\Schulze-244-test-appi-.d	Acquisition Date	6/4/2024 3:14:10 PM
Method	APPI_pos_low_t3.d.m	Operator	MD
Sample Name	Low Concentration Tunemix	Instrument	maXis 288882.20183
Comment	CH2Cl2		

Acquisition Parameter

Source Type	APPI	Ion Polarity	Positive	Set Nebulizer	5.2 Bar
Focus	Not active	Set Capillary	700 V	Set Dry Heater	220 °C
Scan Begin	80 m/z	Set End Plate Offset	-500 V	Set Dry Gas	1.2 l/min
Scan End	1550 m/z	Set Charging Voltage	0 V	Set Divert Valve	Waste
		Set Corona	0 nA	Set APCI Heater	300 °C

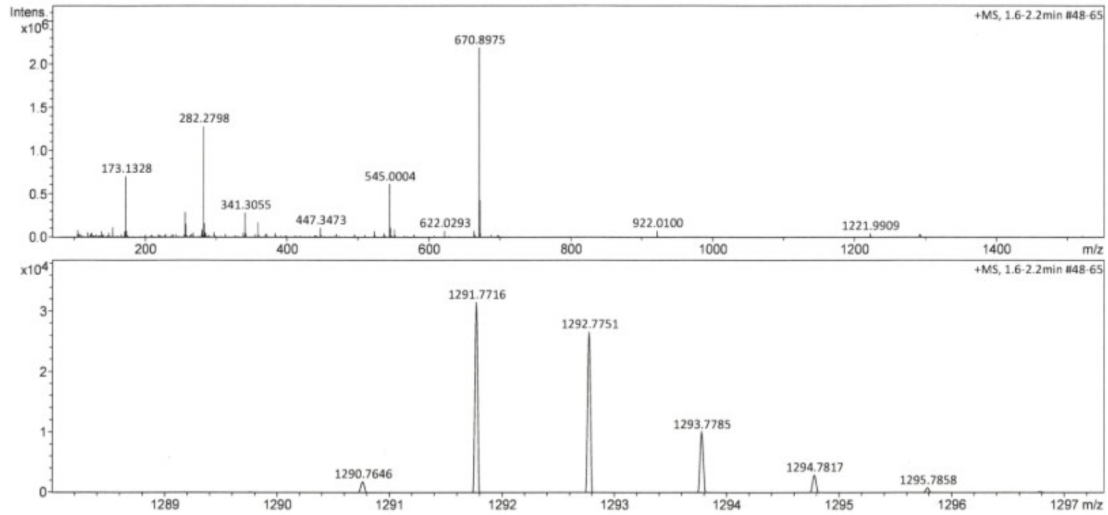

Schulze-244-test-appi-.d

Bruker Compass DataAnalysis 4.2 printed: 6/4/2024 3:26:39 PM by: MD Page 1 of 1

Figure S37. MS/HRMS (APPI) of **22**.

^{1}H NMR (400 MHz, $CDCl_3$, rt)

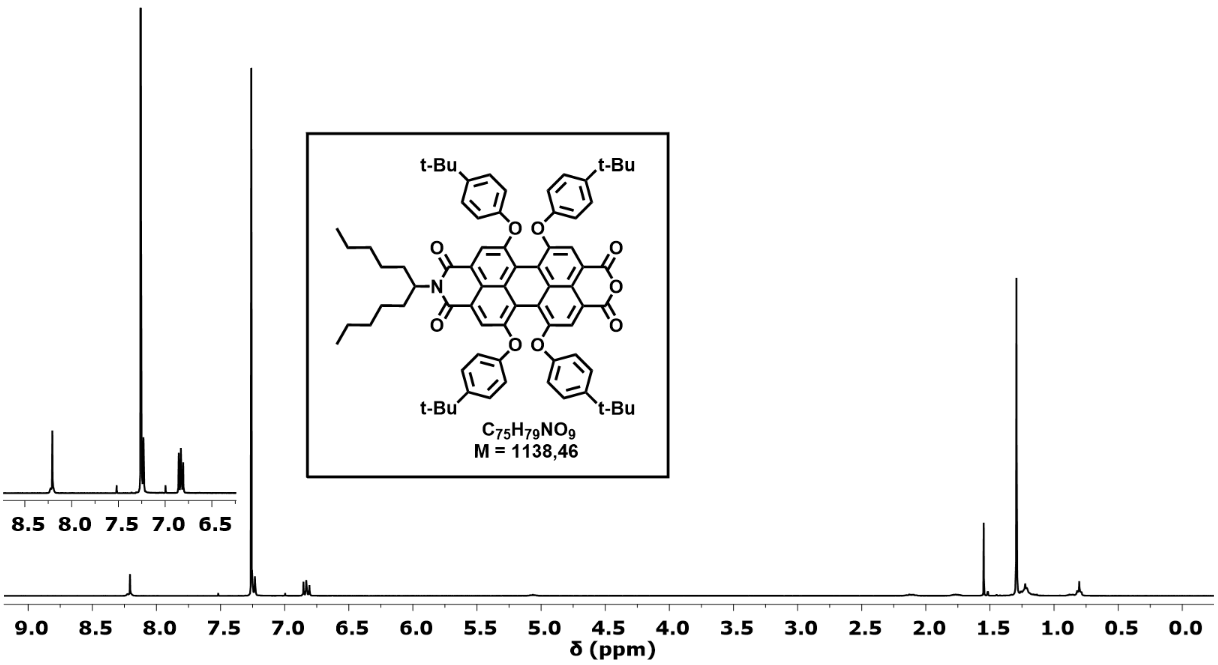

^{13}C NMR (101 MHz, $CDCl_3$, rt)

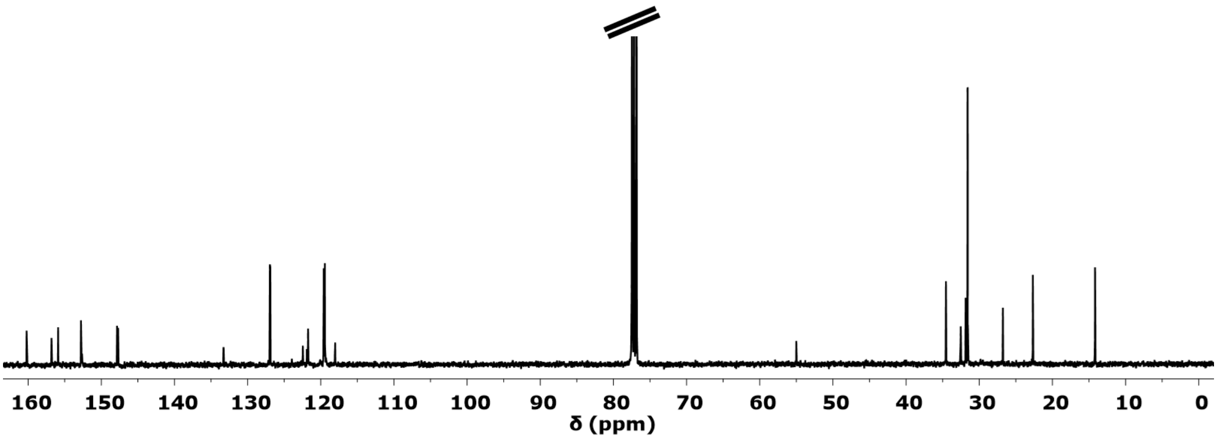

Figure S38. ^{1}H and ^{13}C NMR of **23**.

HRMS (APPI)

Display Report

Analysis Info

Analysis Name	D:\Data\2024\Hirsch -2024\Schulze-ES 245-test.d	Acquisition Date	6/4/2024 10:22:47 AM
Method	APPI_pos_low_t3.d.m	Operator	MD
Sample Name	Low Concentration Tunemix	Instrument	maXis 288882.20183
Comment	CH2Cl2		

Acquisition Parameter

Source Type	APPI	Ion Polarity	Positive	Set Nebulizer	5.2 Bar
Focus	Not active	Set Capillary	700 V	Set Dry Heater	220 °C
Scan Begin	80 m/z	Set End Plate Offset	-500 V	Set Dry Gas	1.2 l/min
Scan End	1550 m/z	Set Charging Voltage	0 V	Set Divert Valve	Waste
		Set Corona	0 nA	Set APCI Heater	250 °C

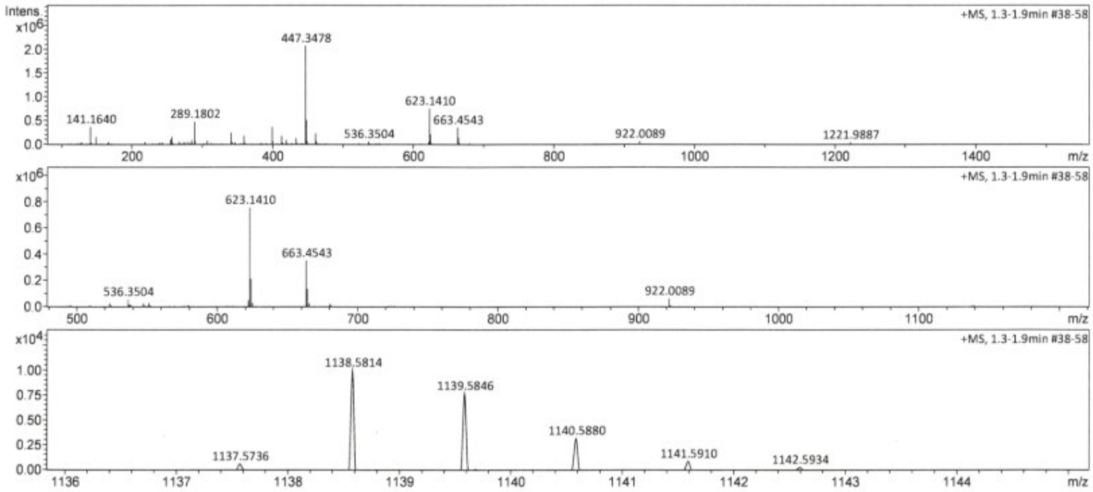

Schulze-ES 245-test.d
Bruker Compass DataAnalysis 4.2 printed: 6/4/2024 10:27:46 AM by: MD Page 1 of 1

Figure S39. MS/HRMS (APPI) of **23**.

^{1}H NMR (400 MHz, $CDCl_3$, rt)

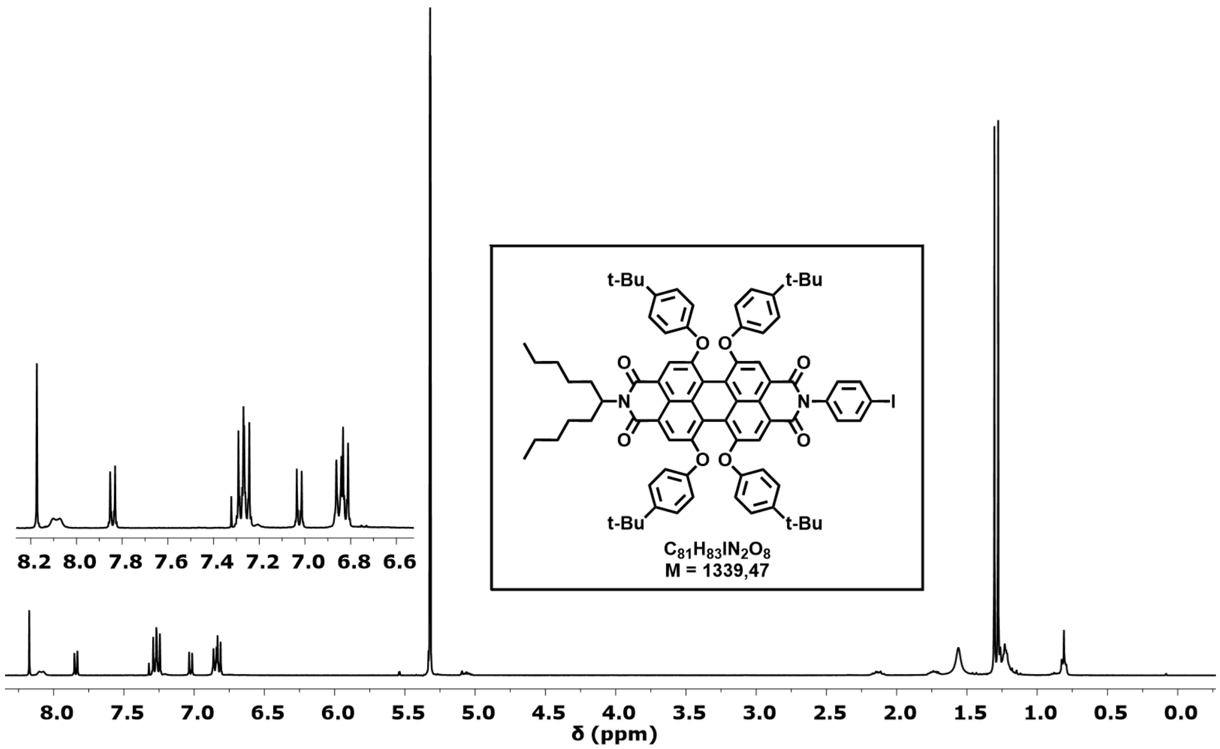

^{13}C NMR (101 MHz, $CDCl_3$, rt)

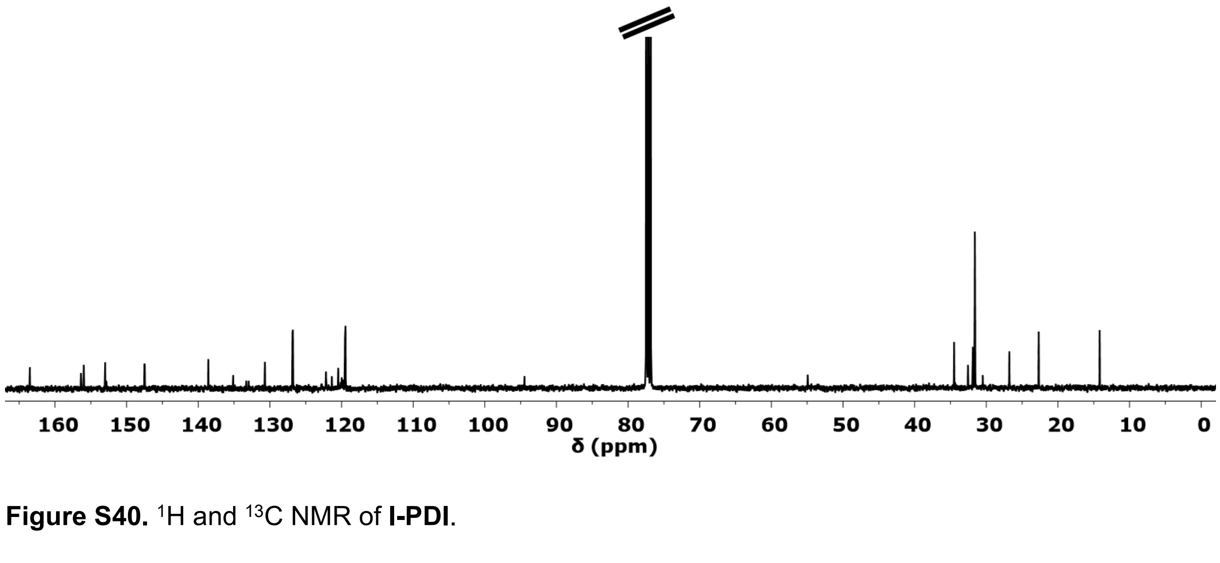

Figure S40. ^{1}H and ^{13}C NMR of **I-PDI**.

HRMS (APPI)

Display Report

Analysis Info			
Analysis Name	D:\Data\2024\Hirsch -2024\Schulze-ES 246-appi-2.d	Acquisition Date	6/4/2024 11:08:04 AM
Method	tune_mid_pos_APPI.m	Operator	MD
Sample Name	Low Concentration Tunemix	Instrument	maXis 288882.20183
Comment	CH2Cl2		

Acquisition Parameter

Source Type	APPI	Ion Polarity	Positive	Set Nebulizer	2.5 Bar
Focus	Not active	Set Capillary	750 V	Set Dry Heater	200 °C
Scan Begin	300 m/z	Set End Plate Offset	-500 V	Set Dry Gas	1.5 l/min
Scan End	2900 m/z	Set Charging Voltage	0 V	Set Divert Valve	Waste
		Set Corona	0 nA	Set APCI Heater	300 °C

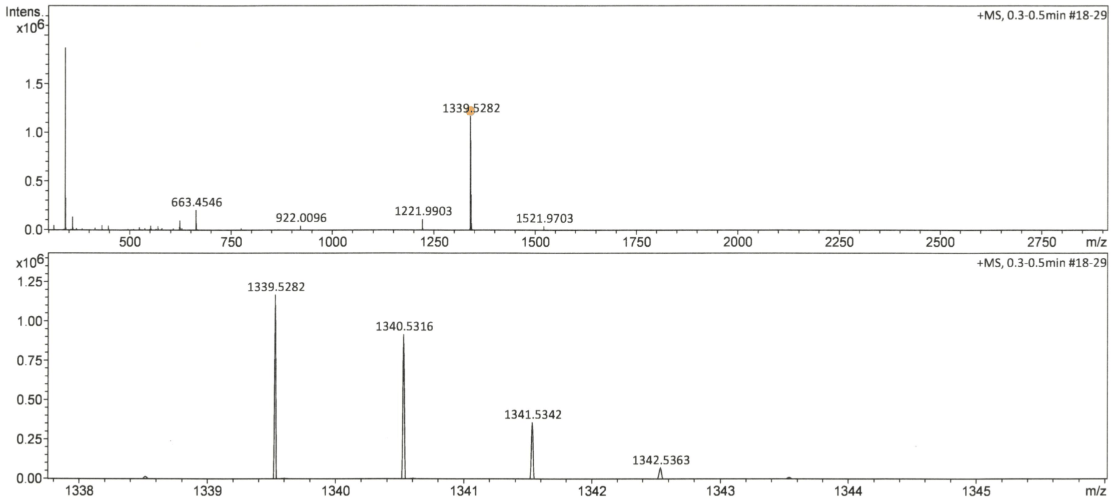

Mass Spectrum SmartFormula Report

Analysis Info			
Analysis Name	D:\Data\2024\Hirsch -2024\Schulze-ES 246-appi-2.d	Acquisition Date	6/4/2024 11:08:04 AM
Method	tune_mid_pos_APPI.m	Operator	MD
Sample Name	Low Concentration Tunemix	Instrument	maXis 288882.20183
Comment	CH2Cl2		

Acquisition Parameter

Source Type	APPI	Ion Polarity	Positive	Set Nebulizer	2.5 Bar
Focus	Not active	Set Capillary	750 V	Set Dry Heater	200 °C
Scan Begin	300 m/z	Set End Plate Offset	-500 V	Set Dry Gas	1.5 l/min
Scan End	2900 m/z	Set Charging Voltage	0 V	Set Divert Valve	Waste
		Set Corona	0 nA	Set APCI Heater	300 °C

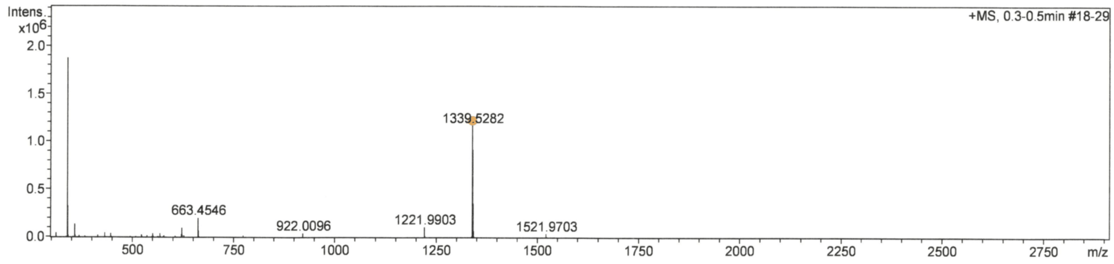

Meas. m/z	#	Ion Formula	m/z	err [ppm]	mSigma	# mSigma	Score	rdb	e⁻ Conf	N-Rule
1339.5282	1	C81H84IN2O8	1339.5267	-1.1	76.7	1	100.00	40.5	even	ok

Figure S41. MS/HRMS (APPI) of **I-PDI**.

^1H NMR (400 MHz, $CDCl_3$, rt)

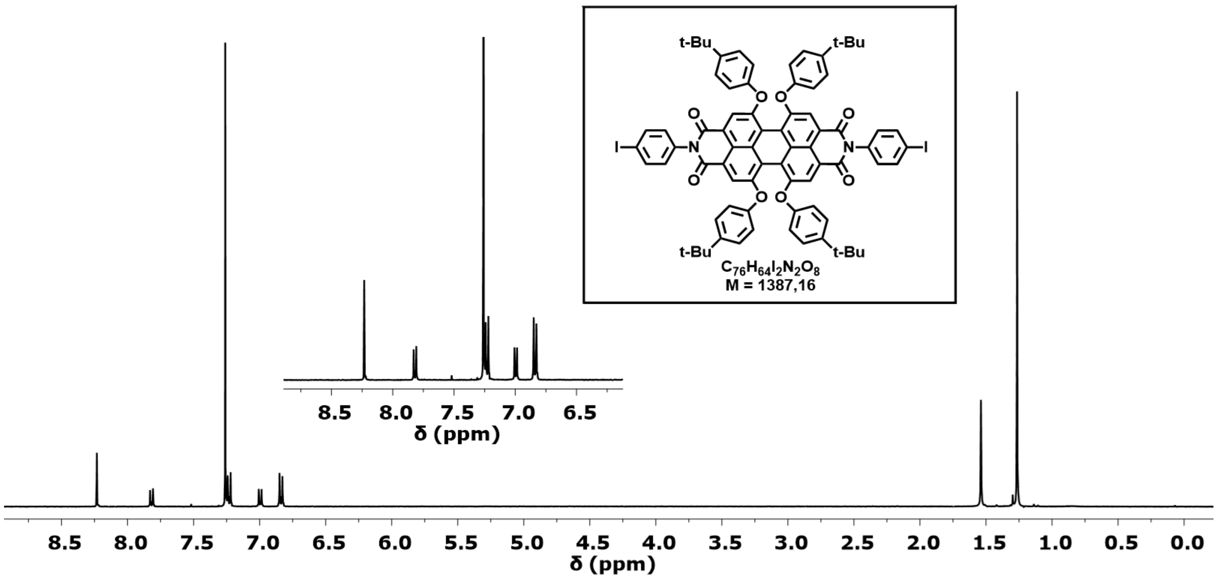

^{13}C NMR (101 MHz, $CDCl_3$, rt)

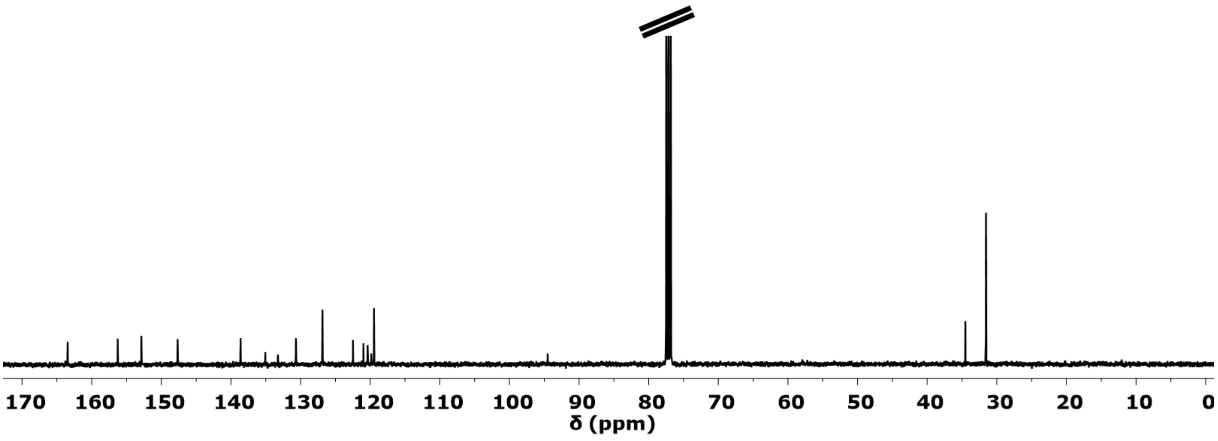

Figure S42. ^1H and ^{13}C NMR of **I-PDI-I**.

MS (MALDI)

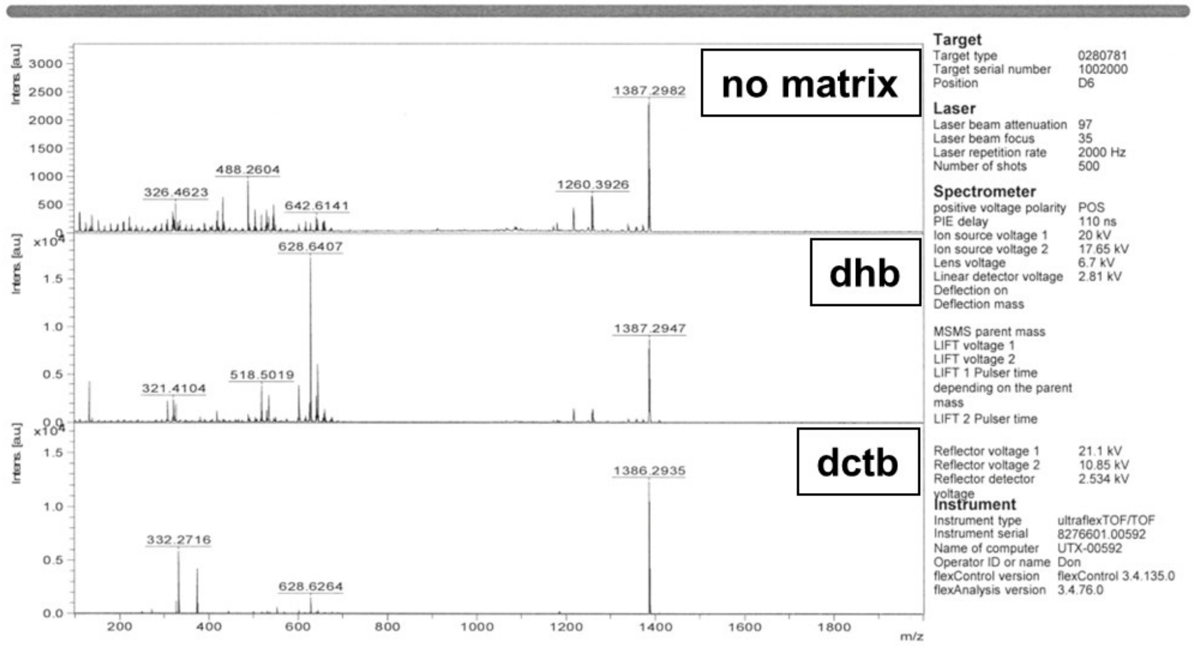

HRMS (MALDI)

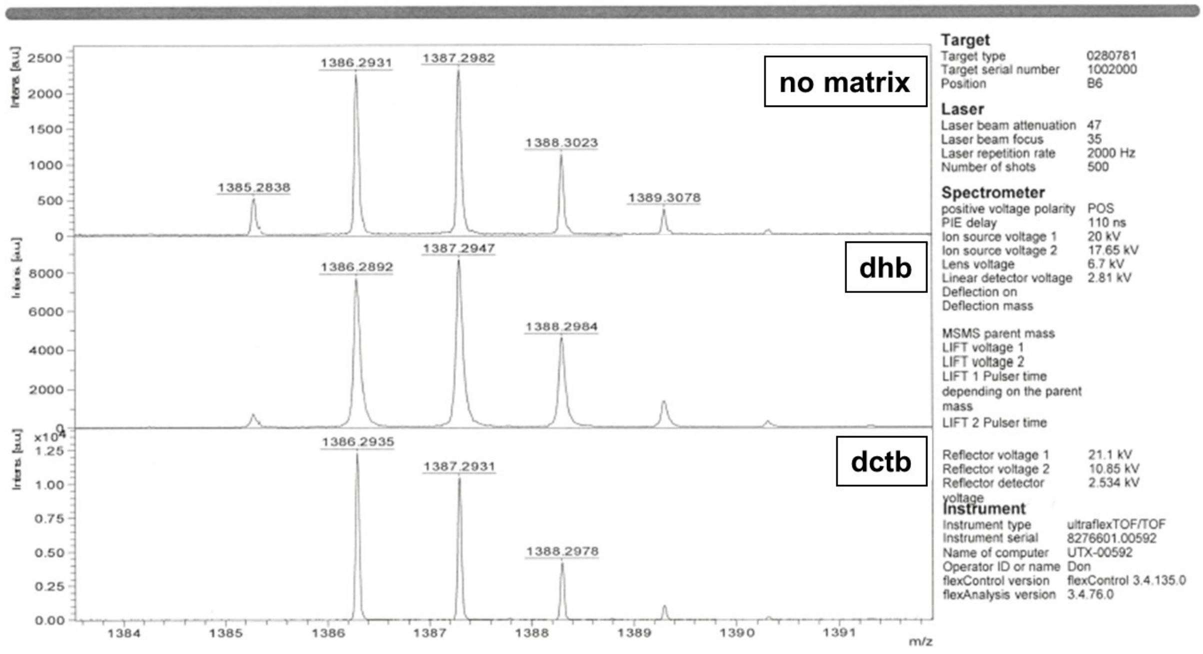

Figure S43. MS/HRMS (MALDI) of **I-PDI-I**.

^{1}H NMR (601 MHz, CD_2Cl_2, rt)

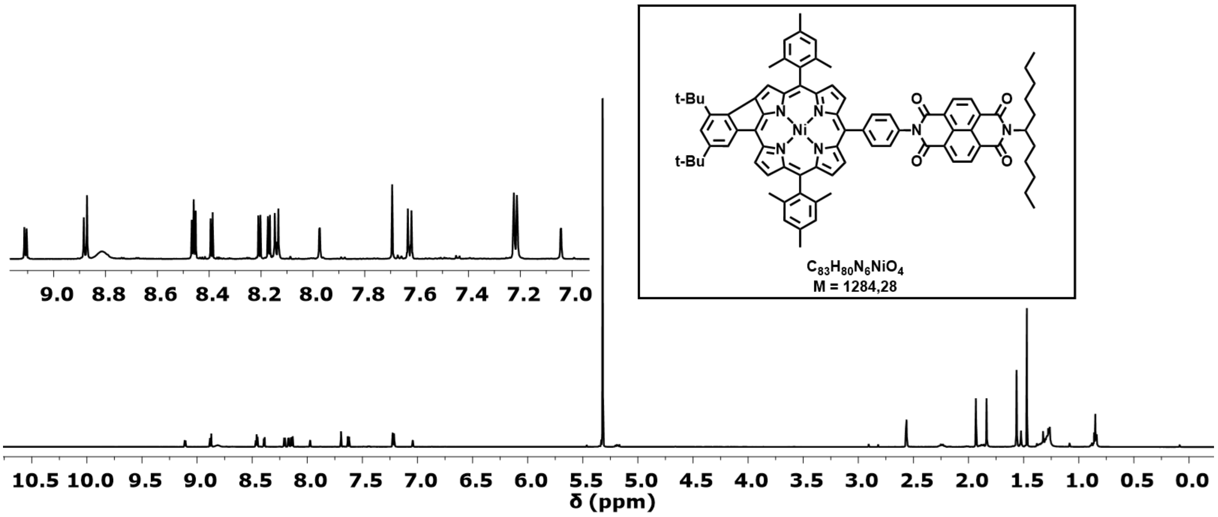

^{13}C NMR - DEPTQ135 (151 MHz, CD_2Cl_2, rt)

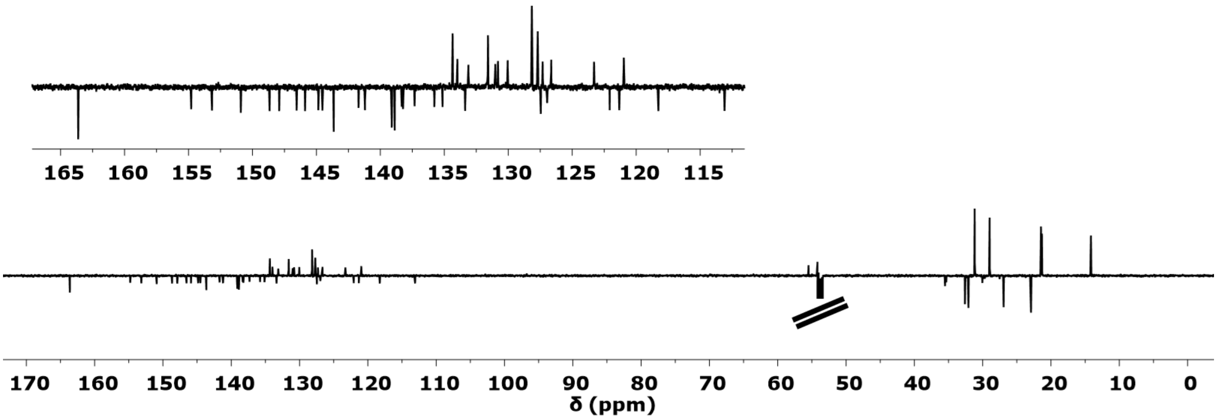

Figure S44. ^{1}H and ^{13}C NMR (DEPTQ135) of **Ph-NDI**.

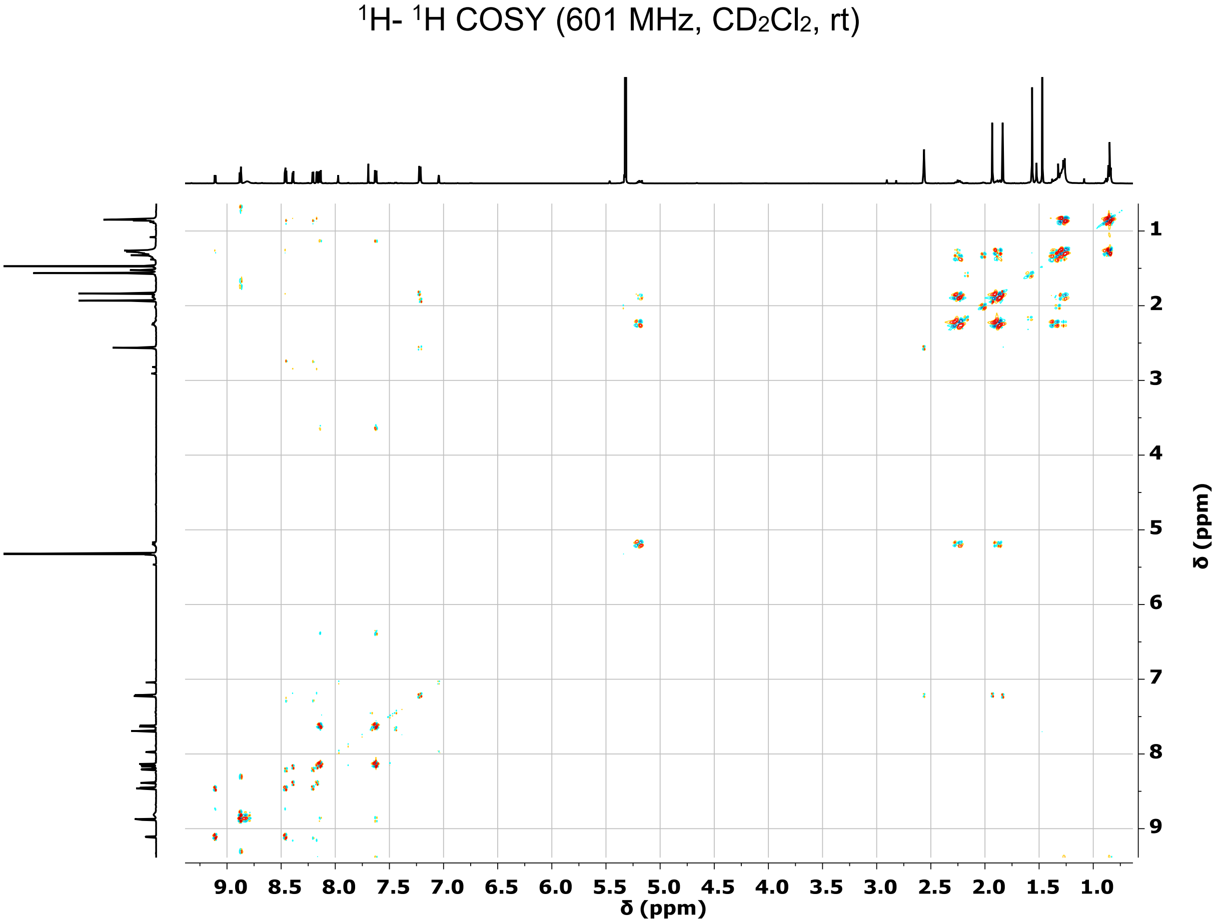

Figure S45. ^1H- ^1H COSY of **Ph-NDI**.

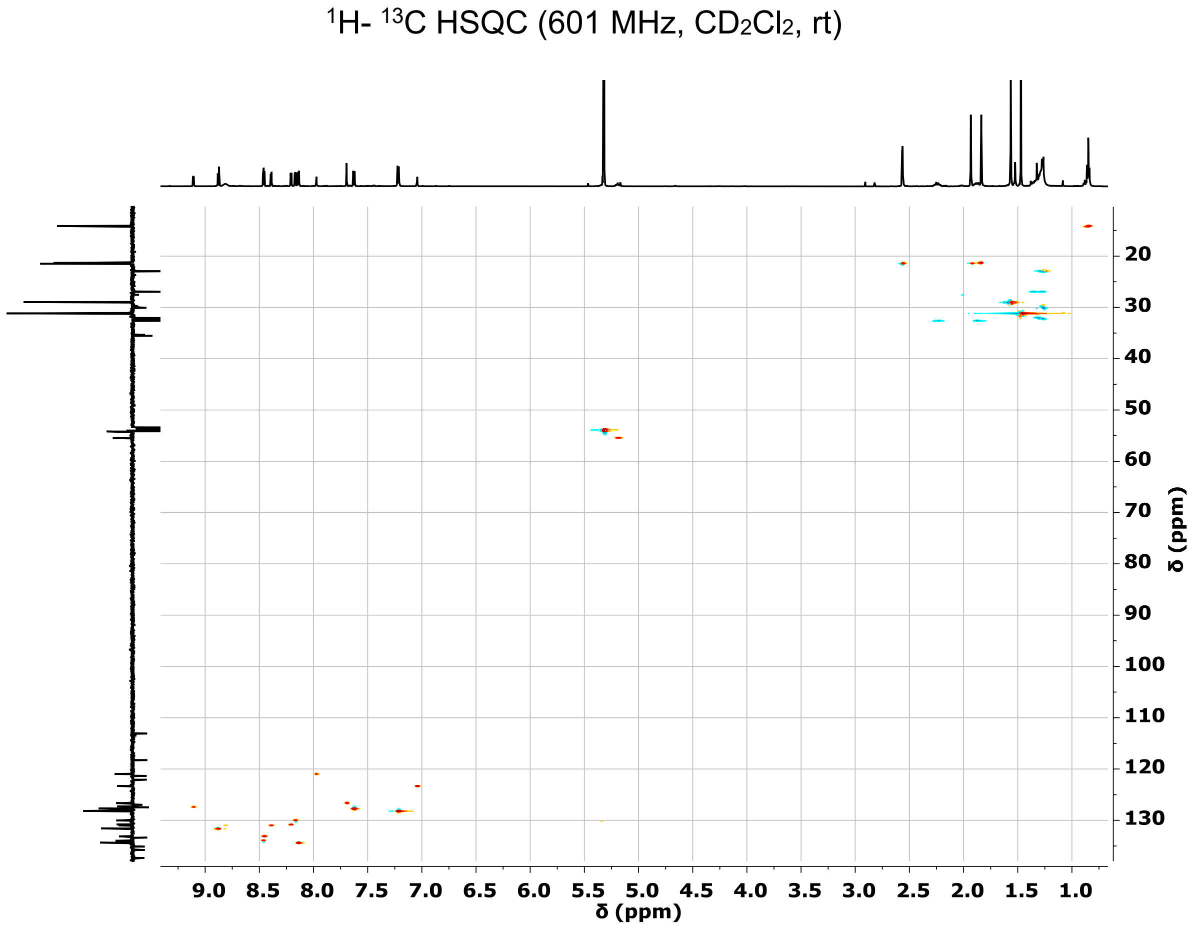

Figure S46. ^{1}H- ^{13}C HSQC of **Ph-NDI**.

¹H- ¹³C HMBC (601 MHz, CD_2Cl_2, rt)

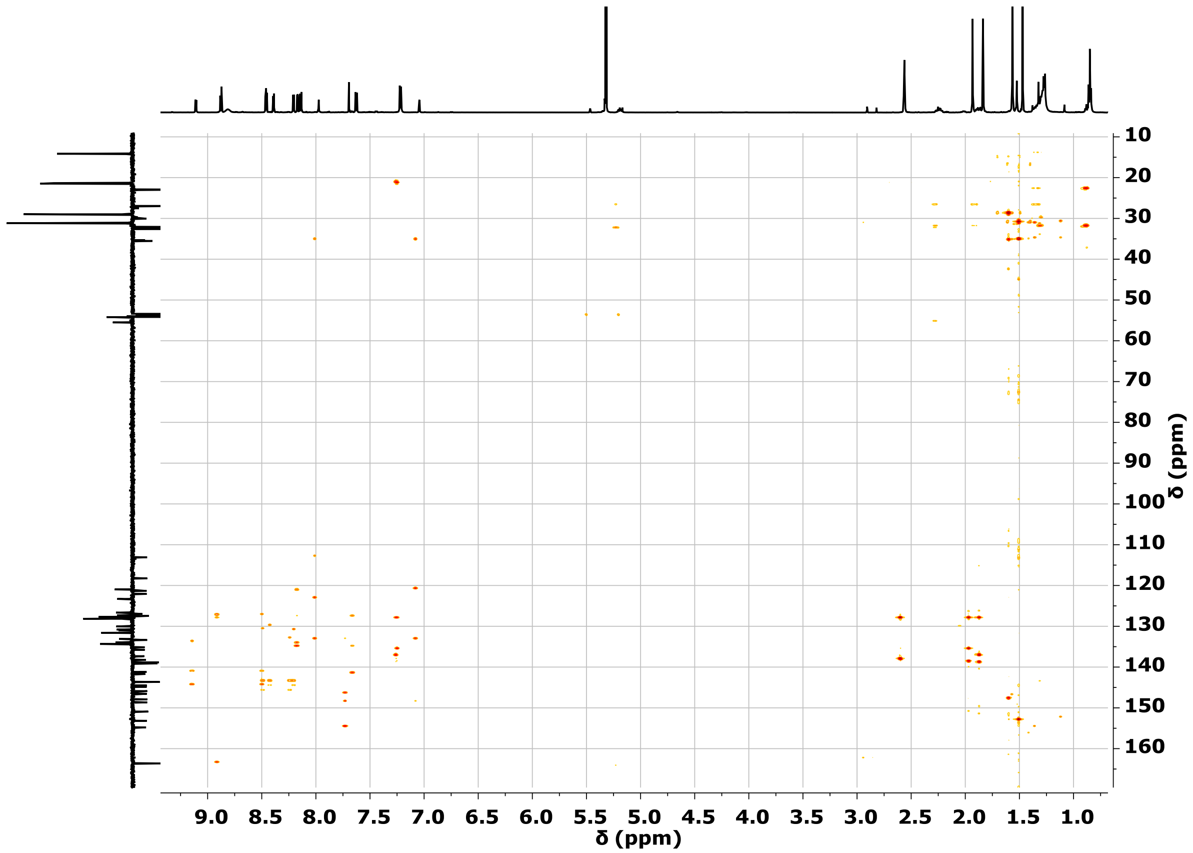

Figure S47. ¹H- ¹³C HMBC of **Ph-NDI**.

MS (MALDI)

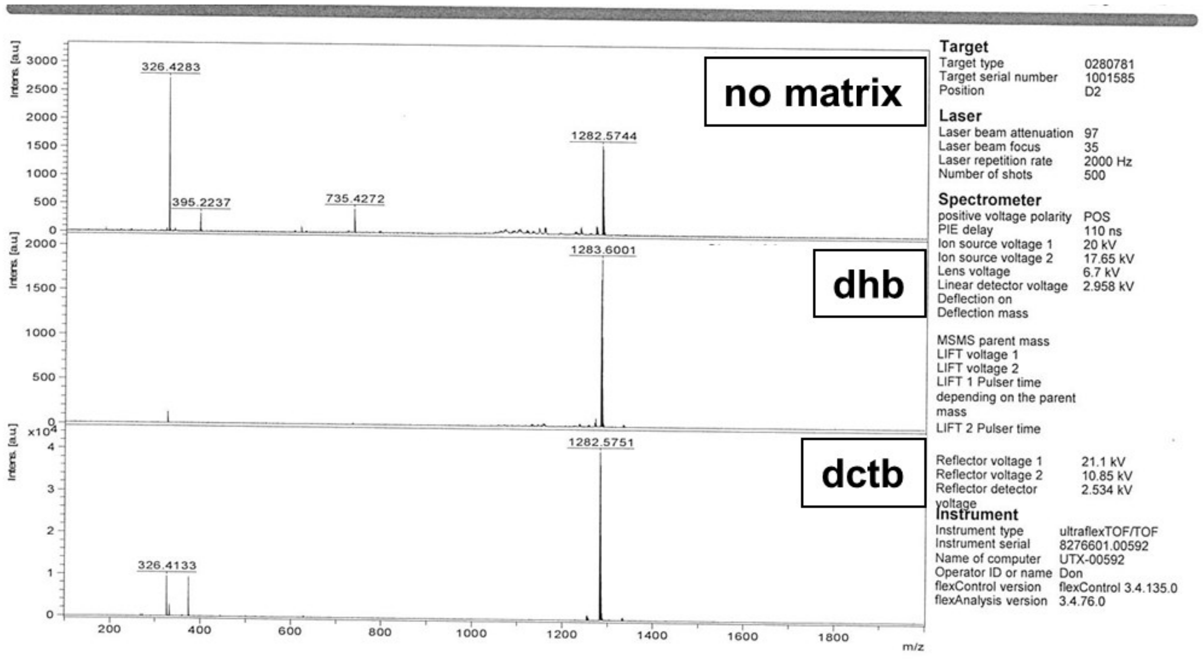

HRMS (MALDI)

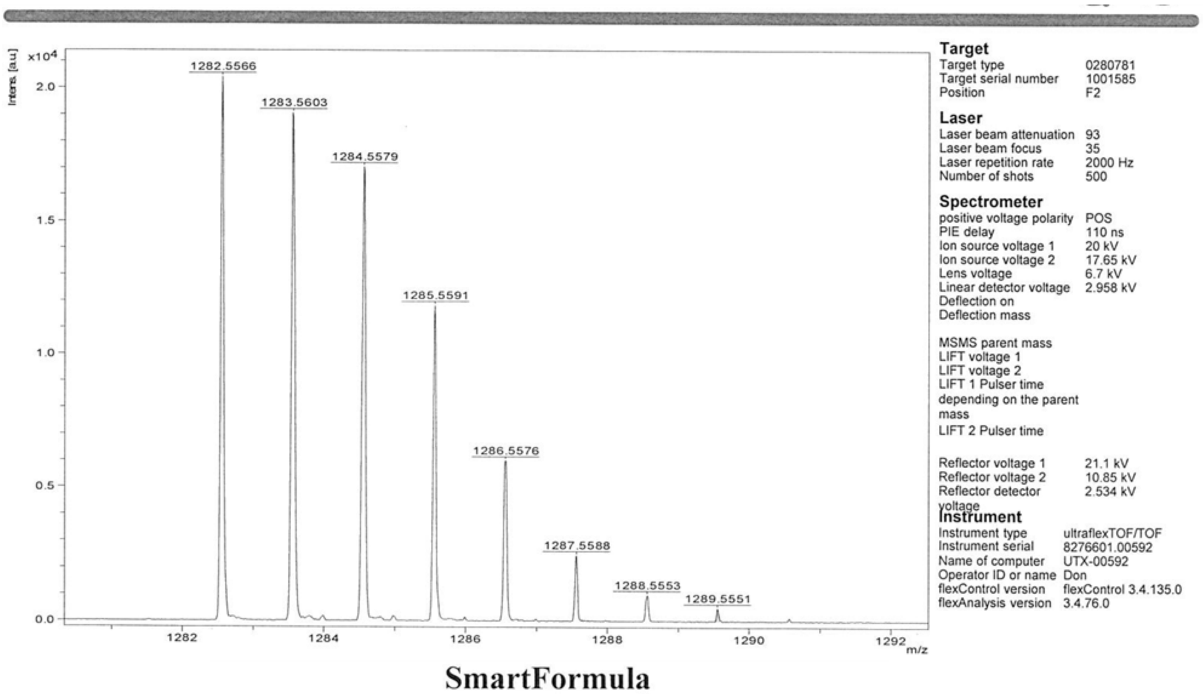

SmartFormula

Formula	Mass	Error	mSigma	DblEq	N rule	Electron Configuration
C 83 H 80 N 6 Ni O 4	1,282.5589	1.8171	24.7208	47.00	ok	odd

Figure S48. MS/HRMS (MALDI) of **Ph-NDI**.

^{1}H NMR (601 MHz, CD_2Cl_2, rt)

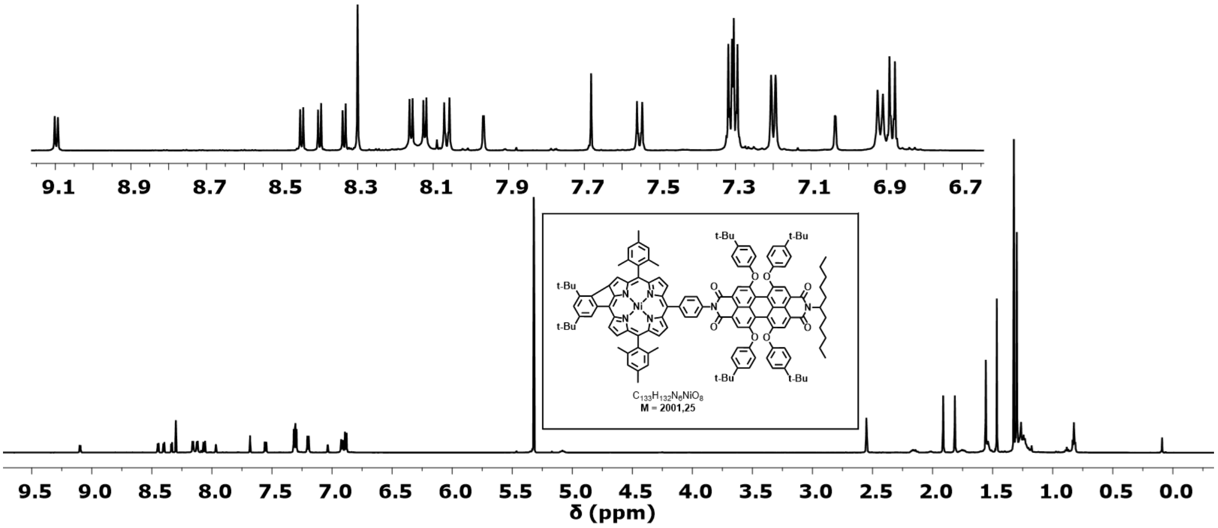

^{13}C NMR - DEPTQ135 (151 MHz, CD_2Cl_2, rt)

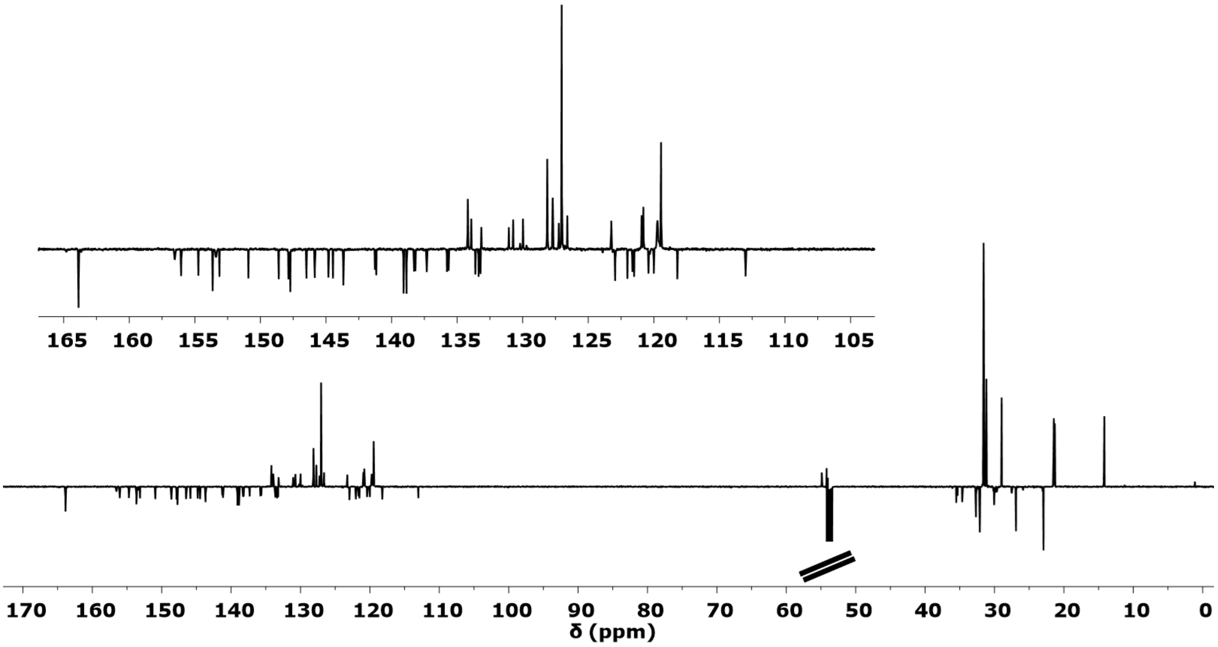

Figure S49. ^{1}H and ^{13}C NMR (DEPTQ135) of **Ph-PDI**.

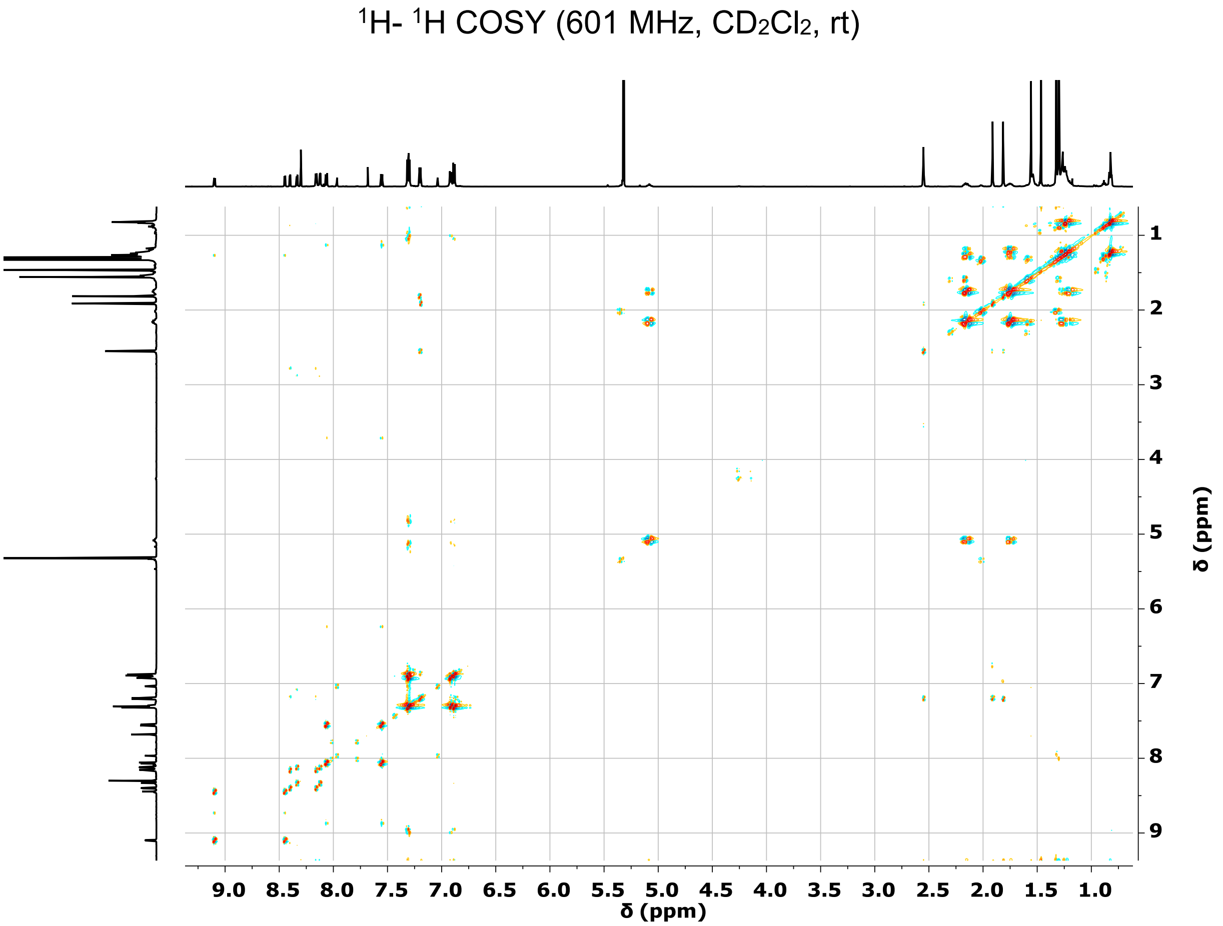

Figure S50. ${}^{1}H$- ${}^{1}H$ COSY of **Ph-PDI**.

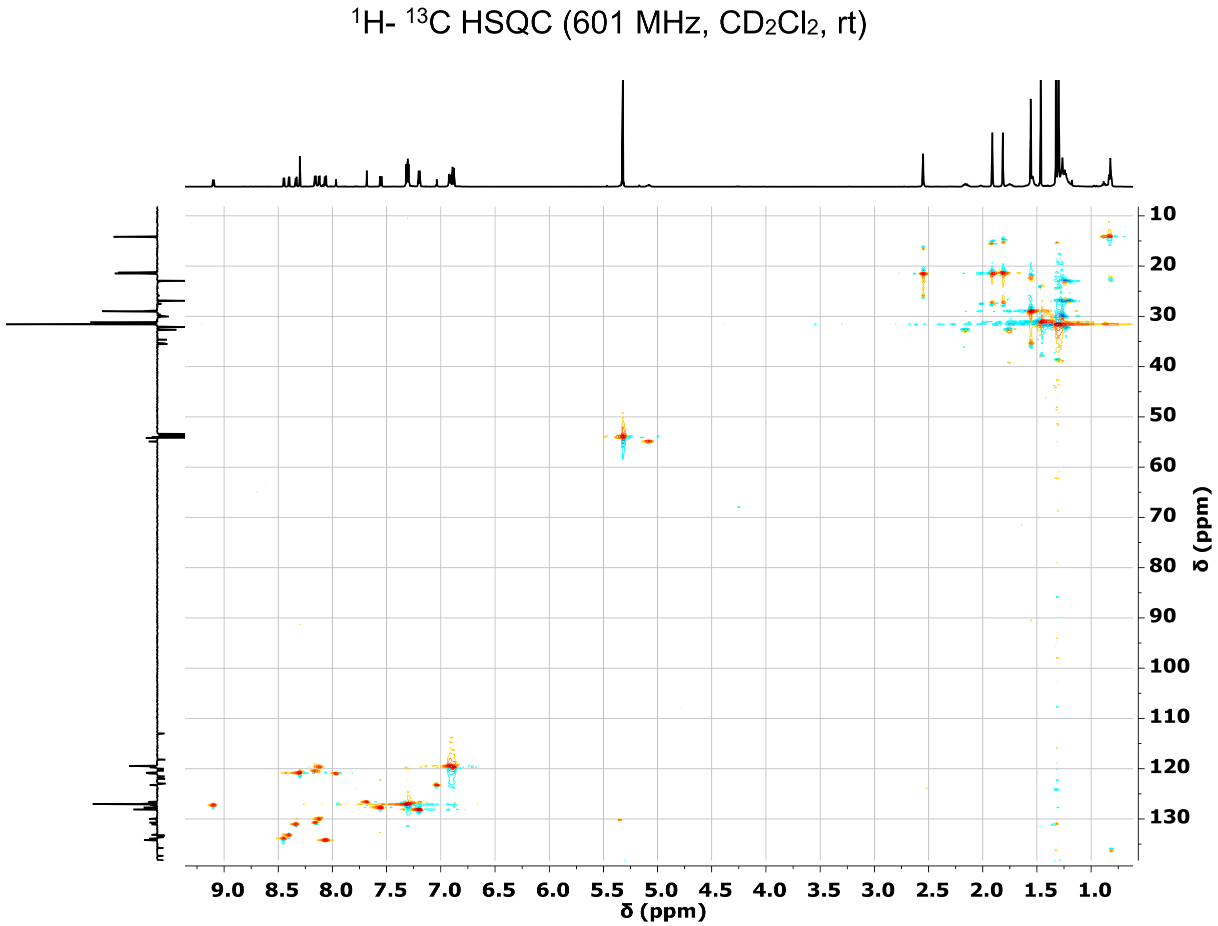

Figure S51. ^{1}H- ^{13}C HSQC of **Ph-PDI**.

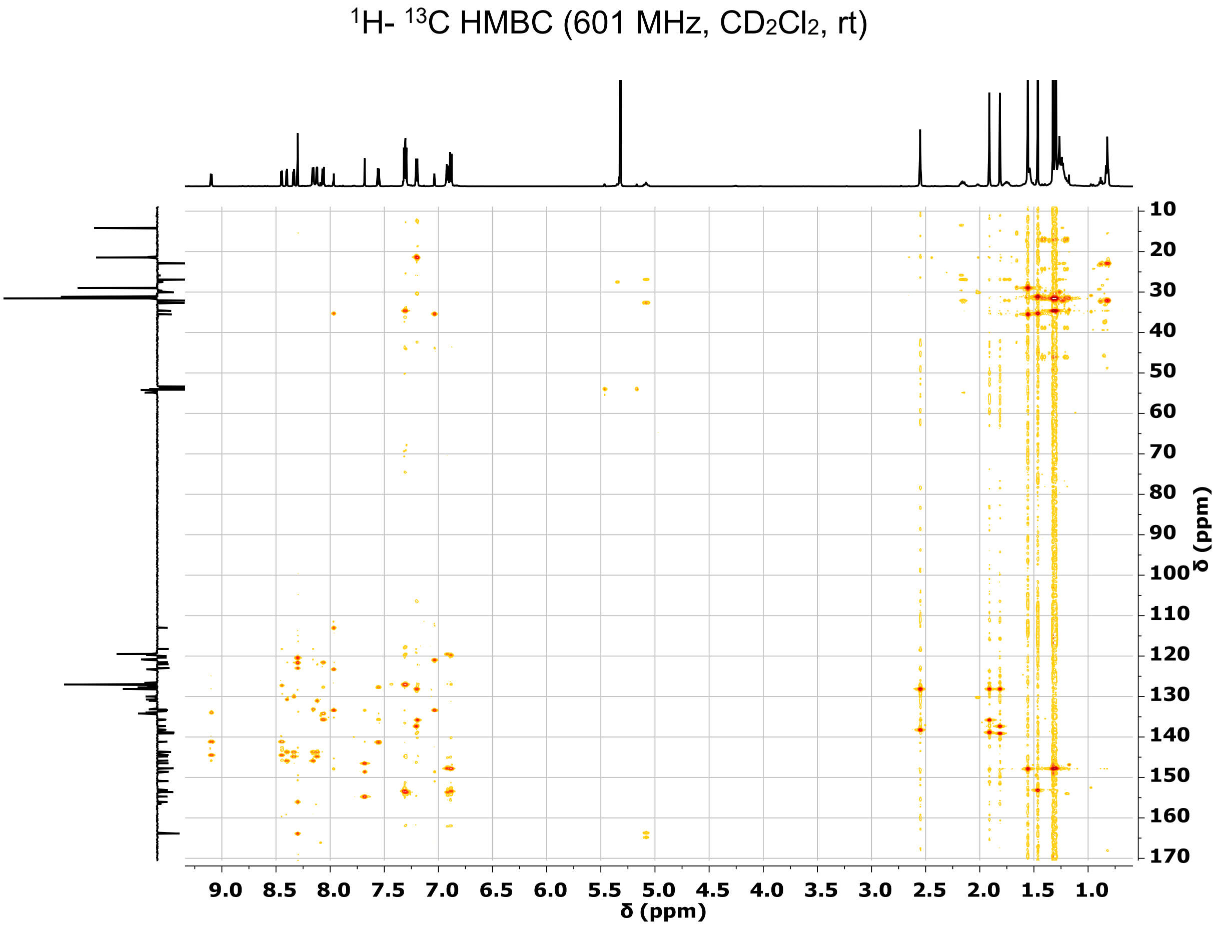

Figure S52. ^{1}H- ^{13}C HMBC of **Ph-PDI**.

MS (MALDI)

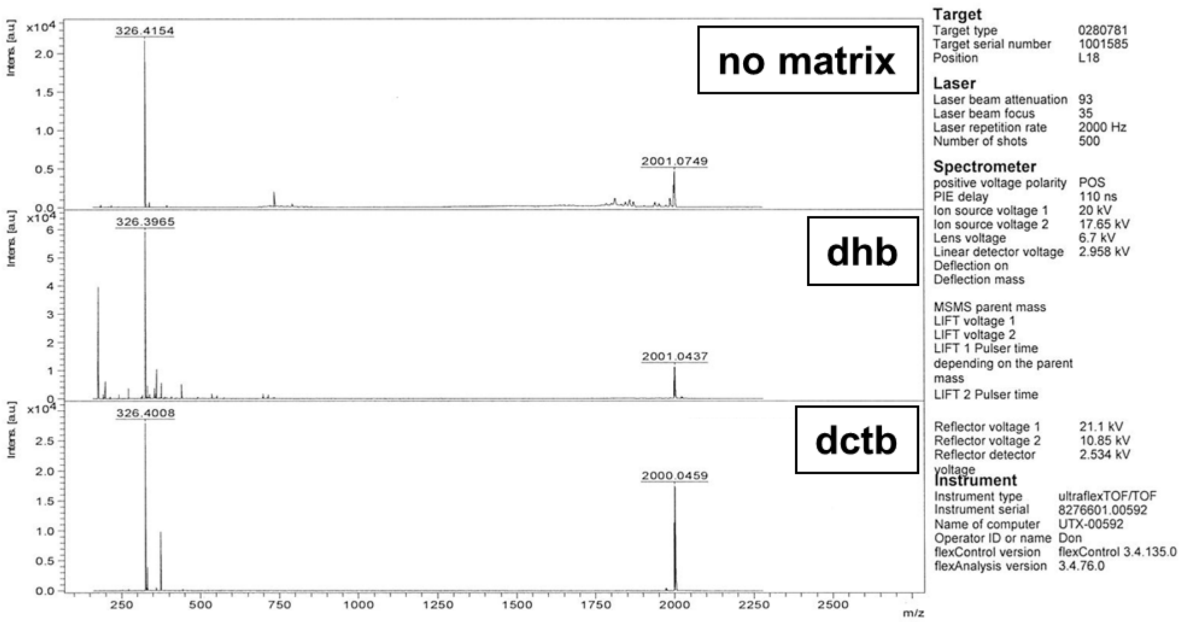

HRMS (MALDI)

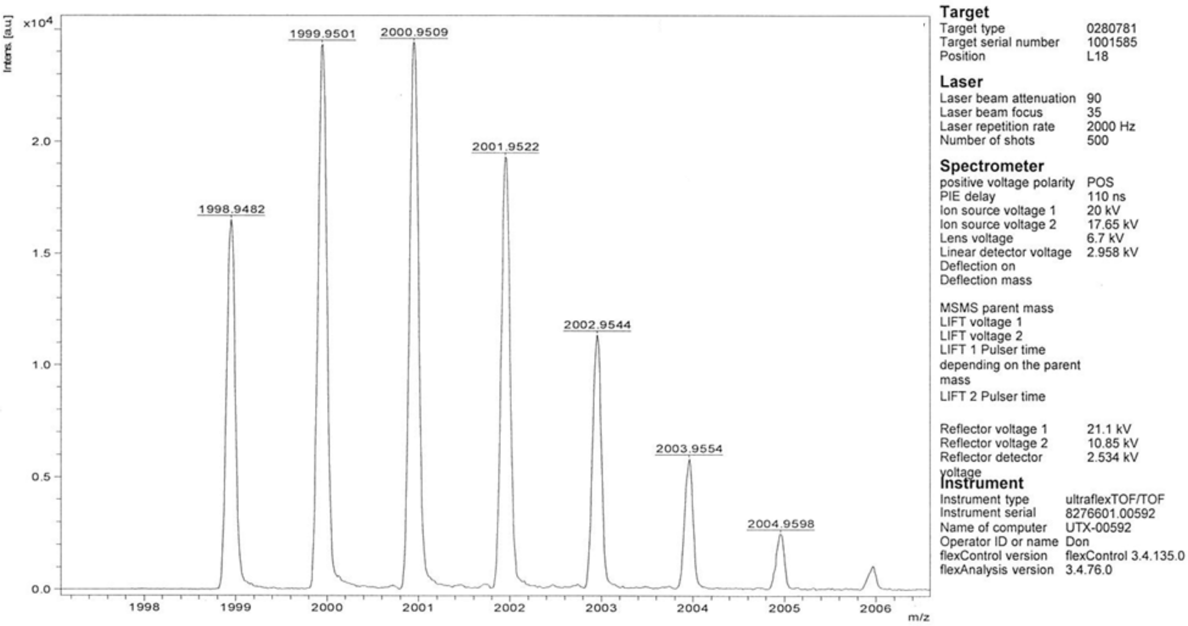

SmartFormula

Formula	Mass	Error	mSigma	DblEq	N rule	Electron Configuration
C 133 H 132 N 6 Ni O 8	1,998.9455	1.3711	22.1727	71.00	ok	odd

Figure S53. MS/HRMS (MALDI) of **Ph-PDI**.

^{1}H NMR (601 MHz, CD_2Cl_2, rt)

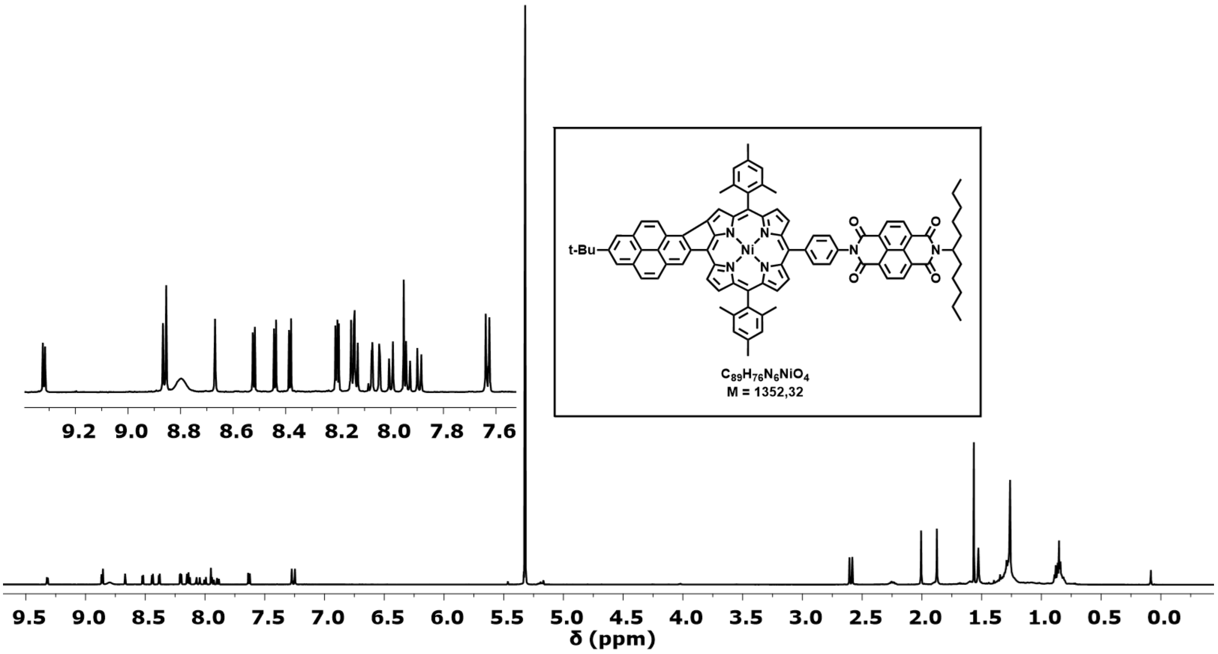

^{13}C NMR - DEPTQ135 (151 MHz, CD_2Cl_2, rt)

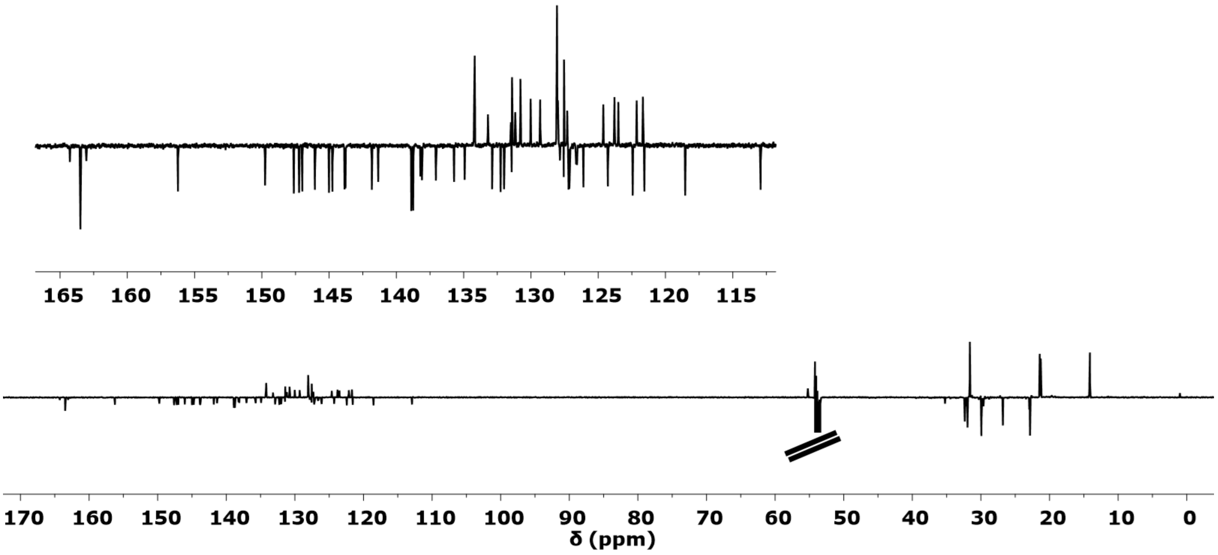

Figure S54. ^{1}H and ^{13}C NMR (DEPTQ135) of **Pyr-NDI**.

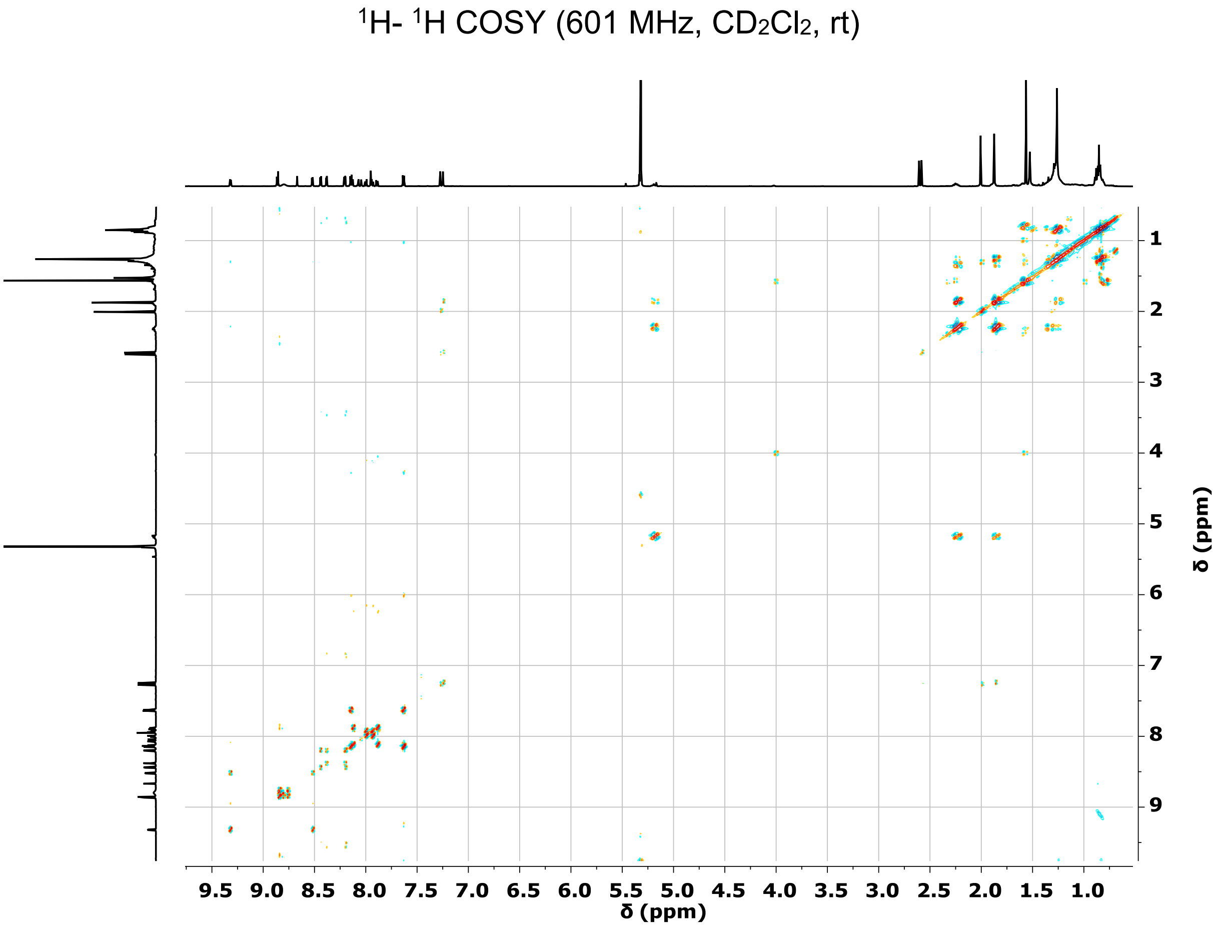

Figure S55. ^{1}H- ^{1}H COSY of **Pyr-NDI**.

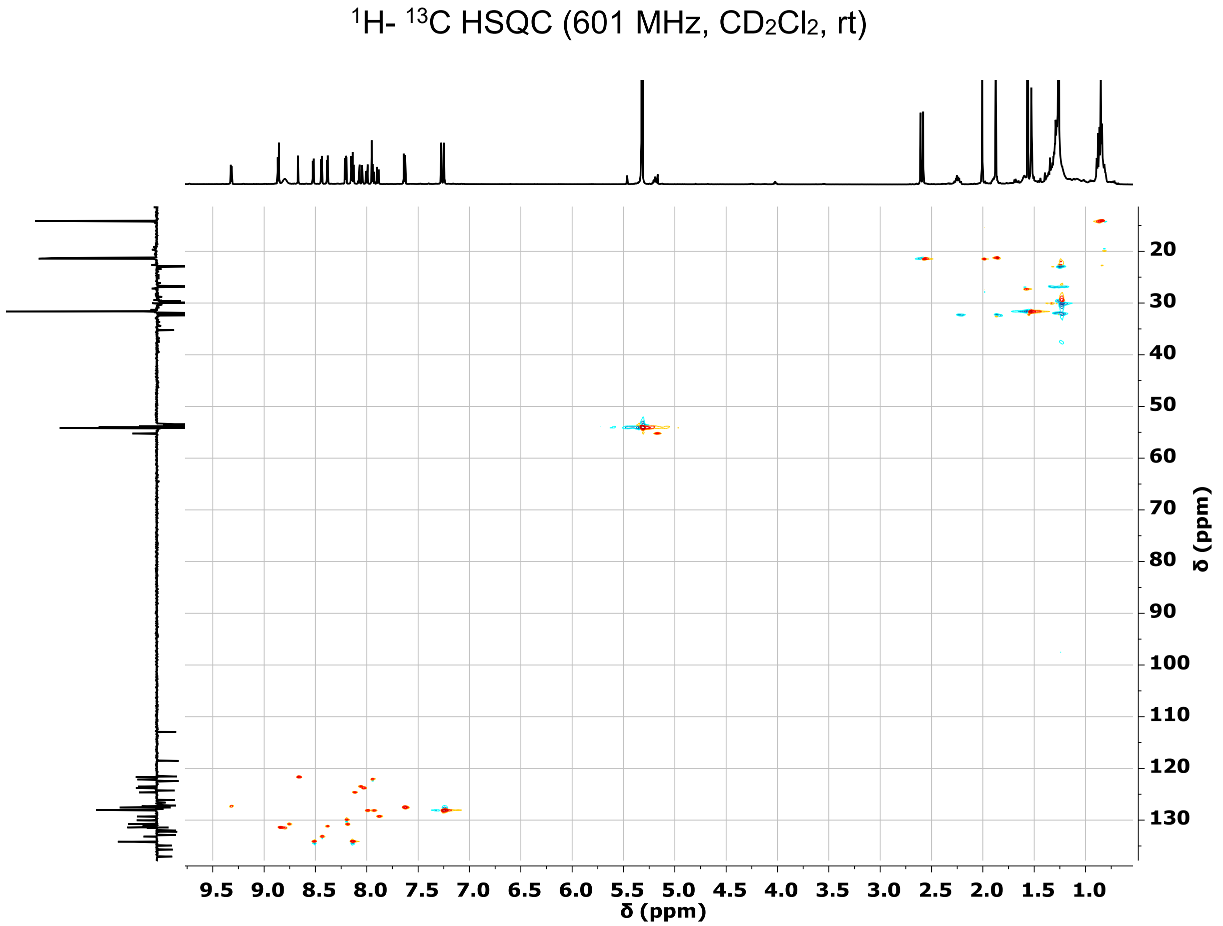

Figure S56. ^{1}H- ^{13}C HSQC of **Pyr-NDI**.

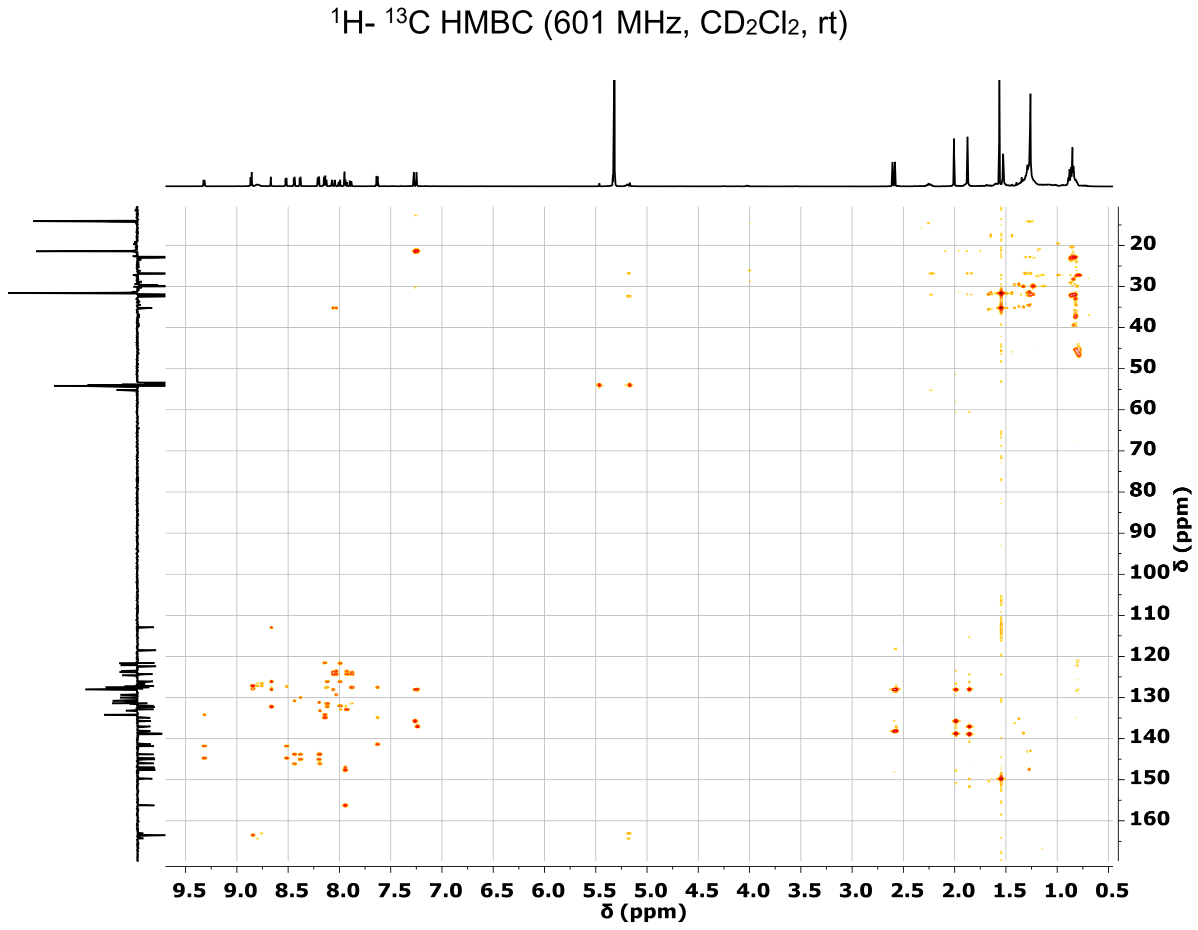

Figure S57. ^{1}H- ^{13}C HMBC of **Pyr-NDI**.

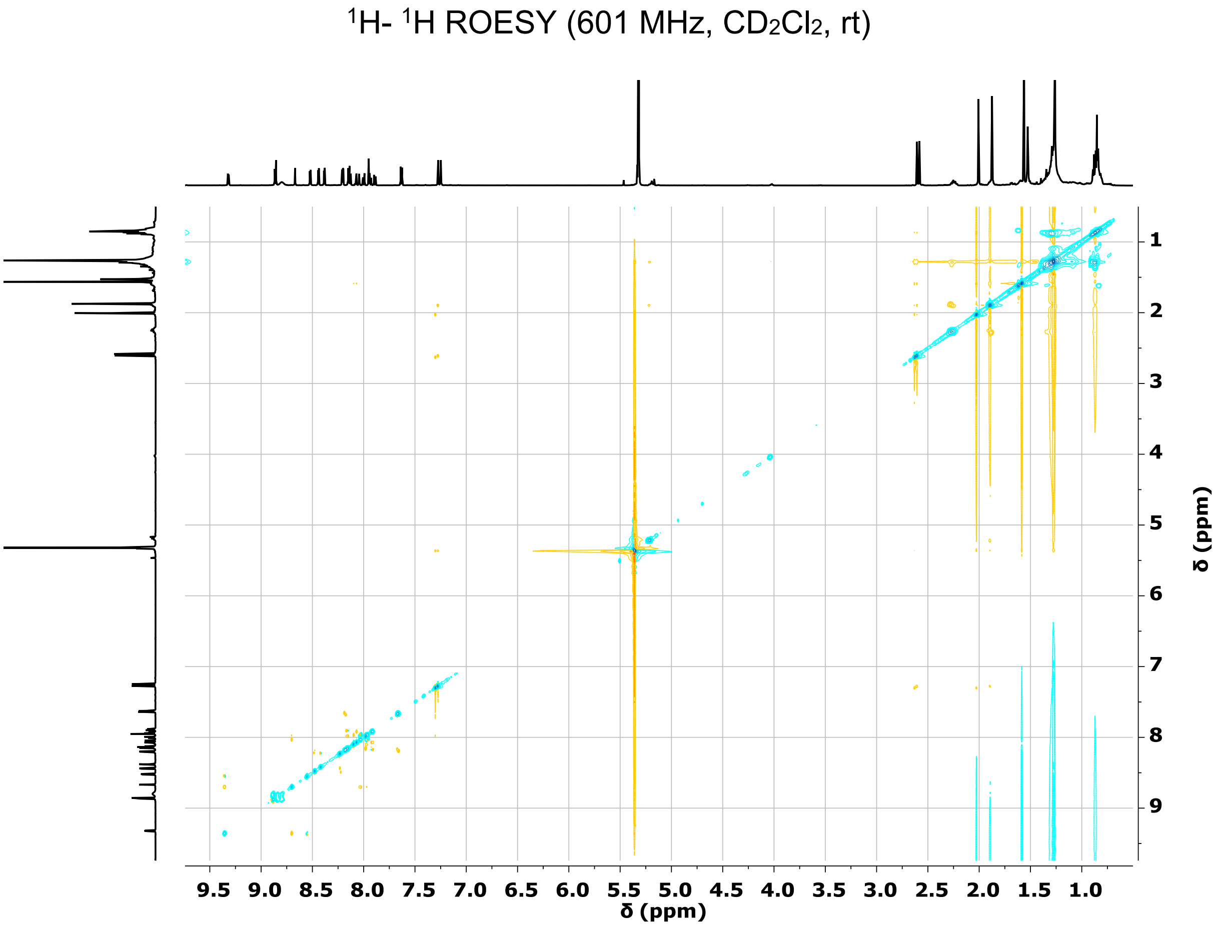

Figure S58. ^{1}H- ^{1}H ROESY of **Pyr-NDI**.

MS (MALDI)

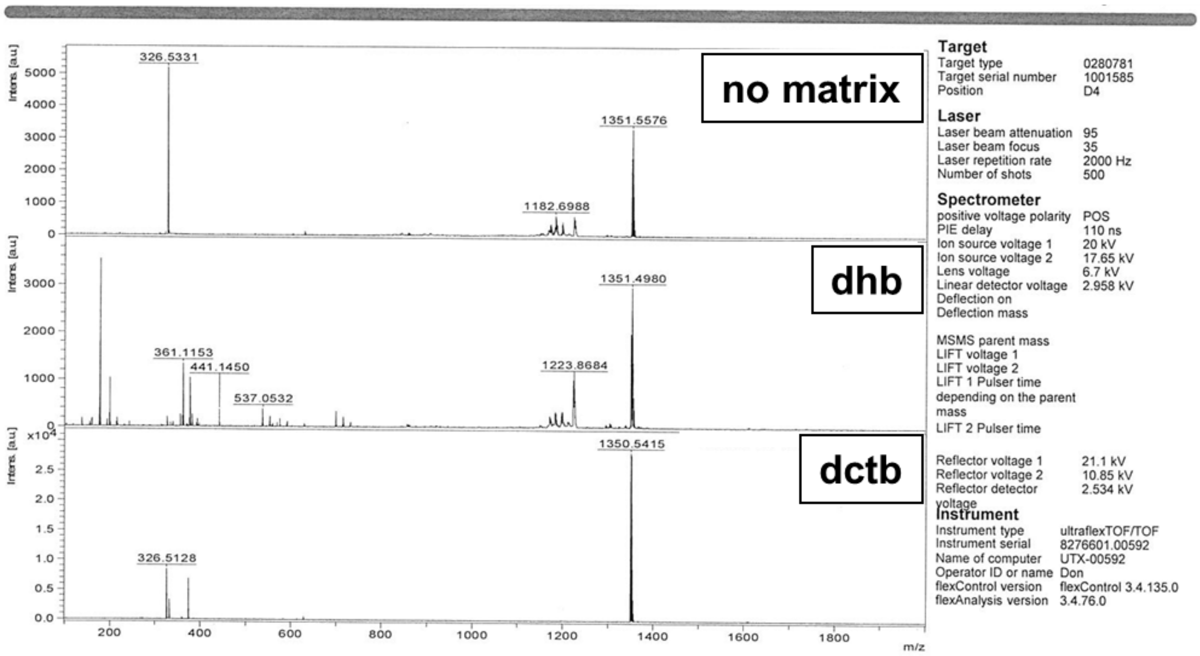

HRMS (MALDI)

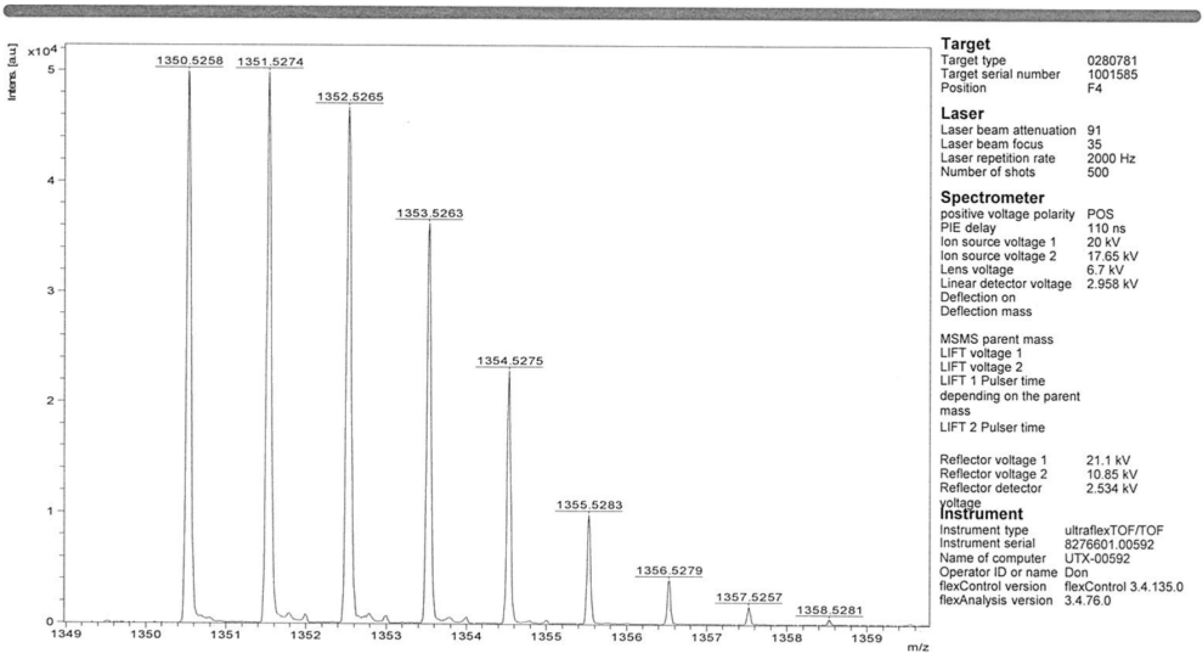

SmartFormula

Formula	Mass	Error	mSigma	DblEq	N rule	Electron Configuration
C 89 H 76 N 6 Ni O 4	1,350.5276	1.3678	79.0430	55.00	ok	odd

Figure S59. MS/HRMS (MALDI) of **Pyr-NDI**.

^1H NMR (601 MHz, CD_2Cl_2/CS_2, rt)

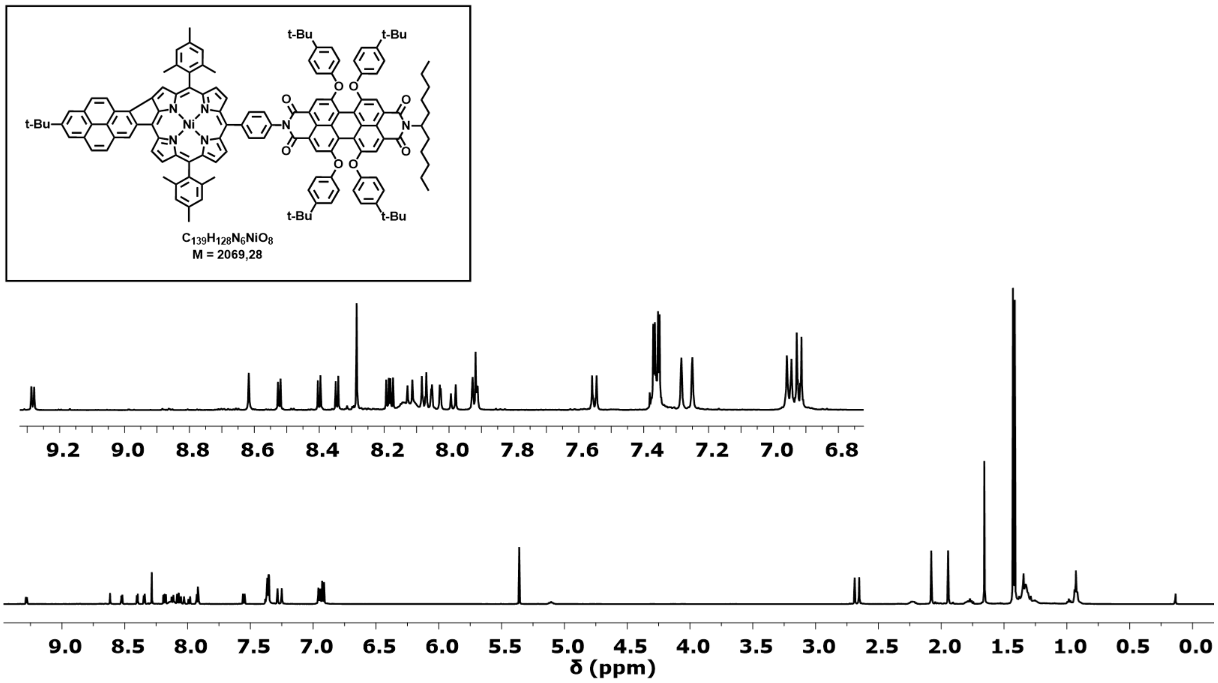

^{13}C NMR - DEPTQ135 (151 MHz, CD_2Cl_2/CS_2, rt)

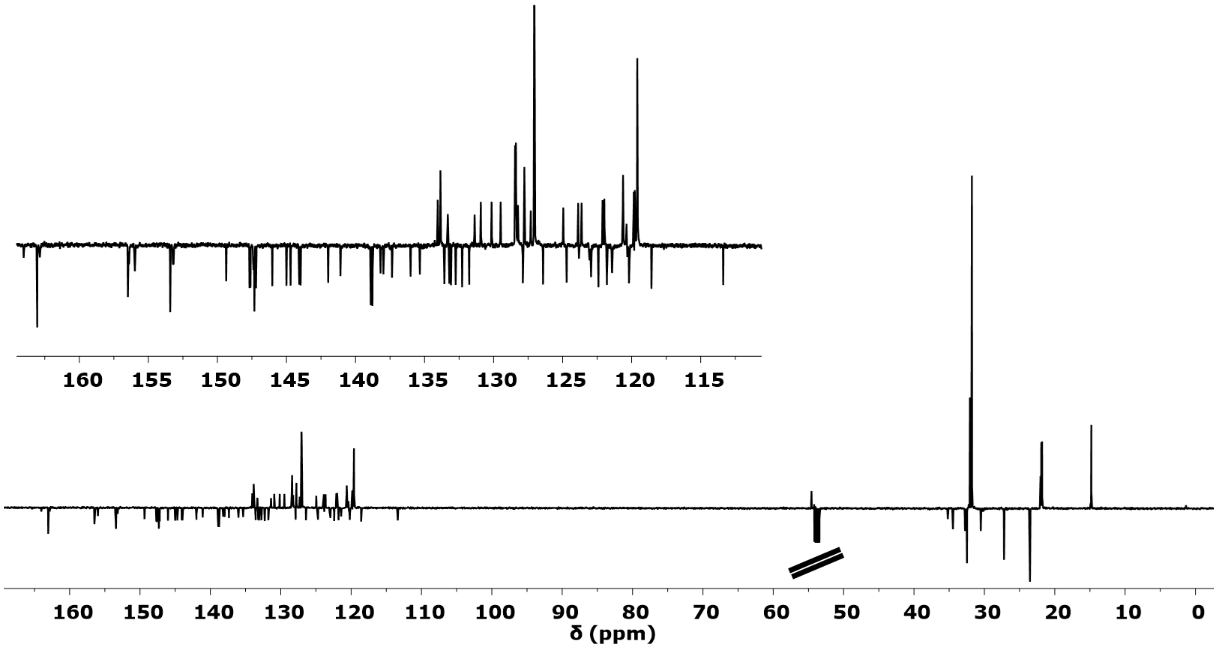

Figure S60. ^1H and ^{13}C NMR (DEPTQ135) of **Pyr-PDI**.

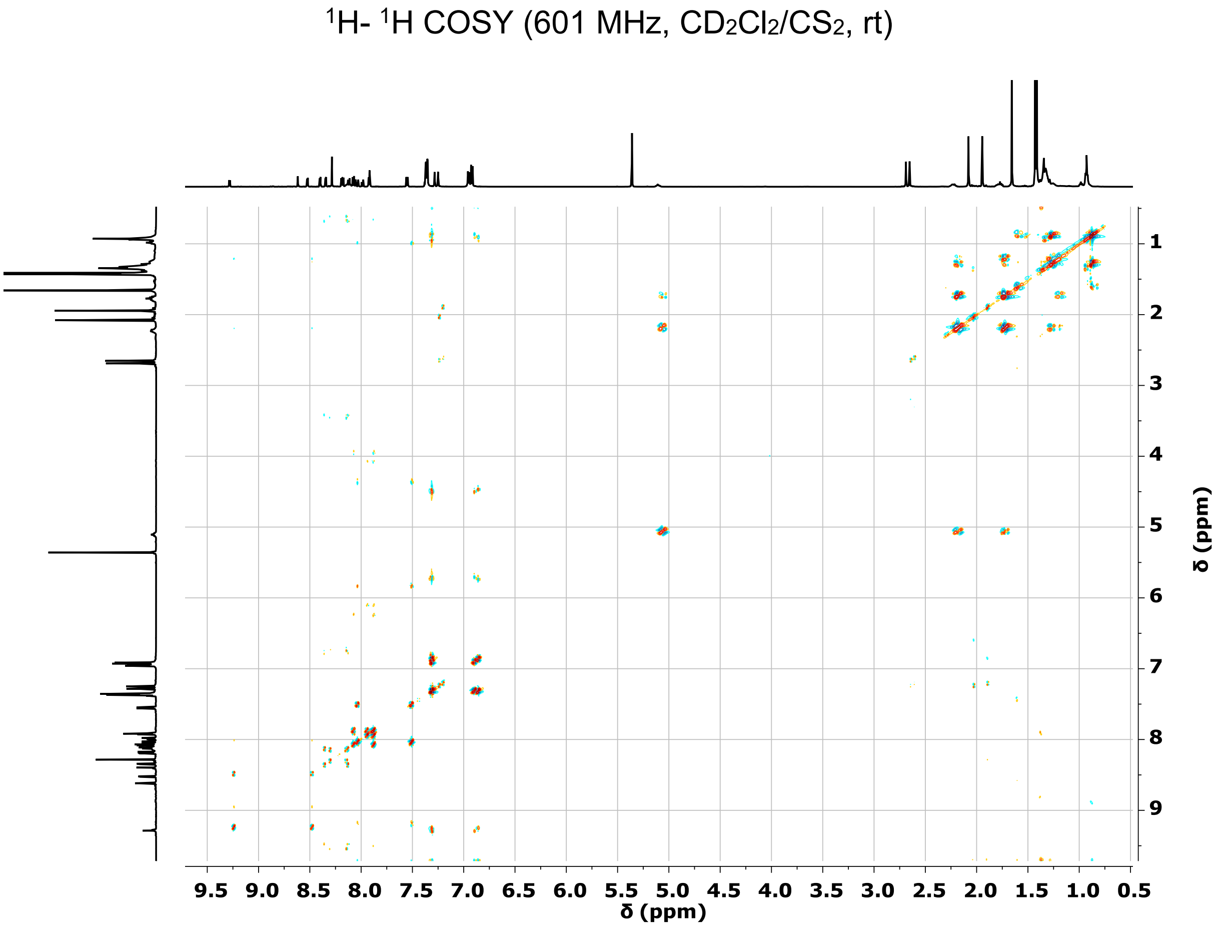

Figure S61. ¹H- ¹H COSY of **Pyr-PDI**.

^1H- ^{13}C HSQC (601 MHz, CD_2Cl_2/CS_2, rt)

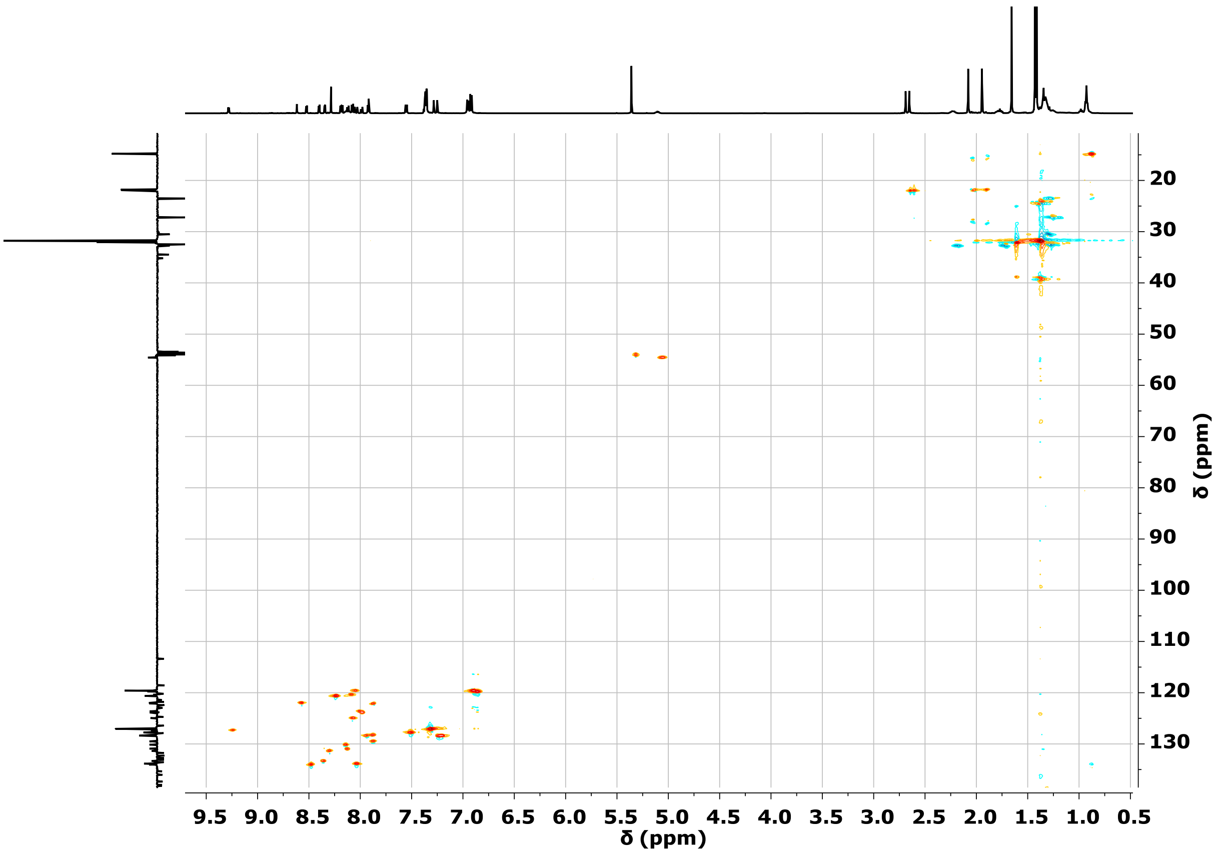

Figure S62. ^1H- ^{13}C HSQC of **Pyr-PDI**.

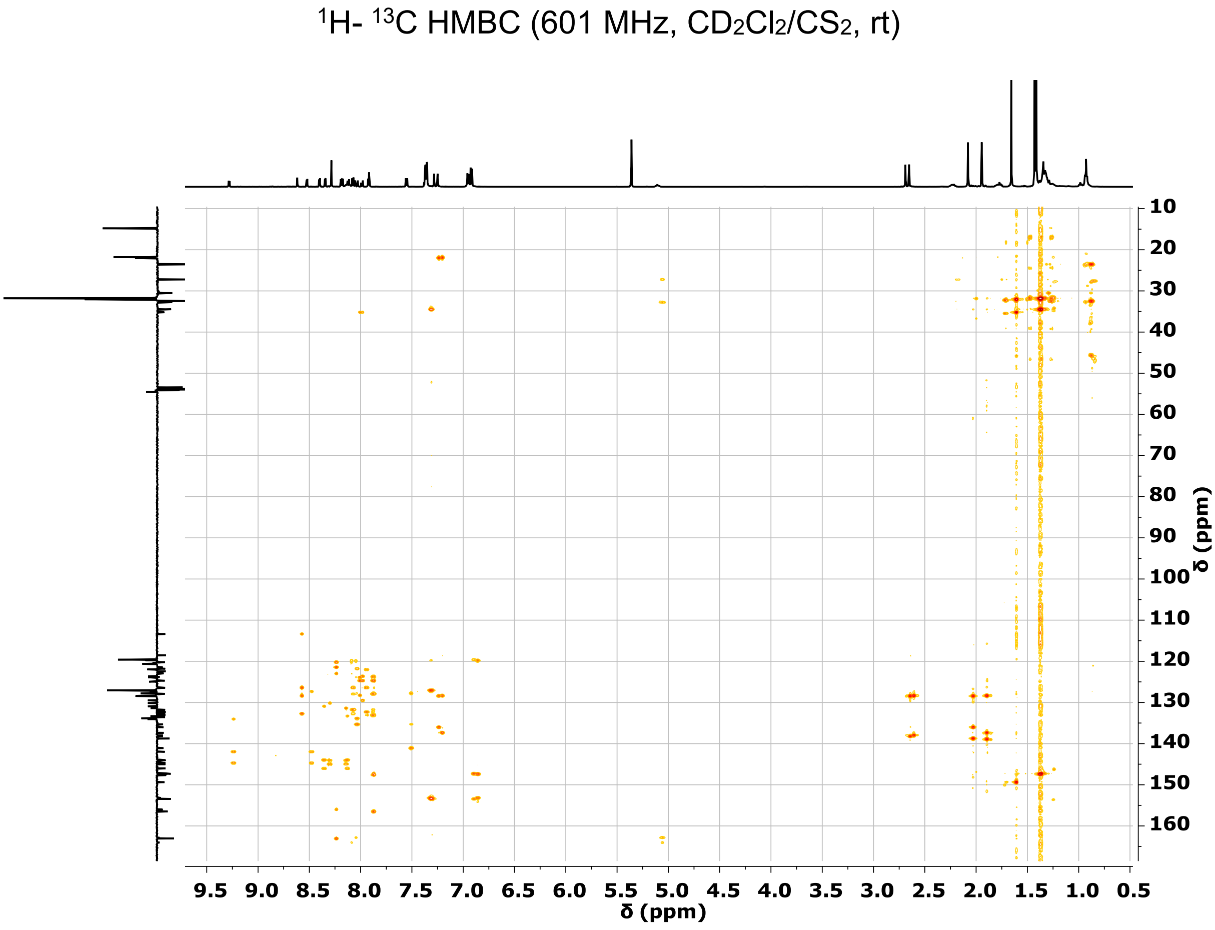

Figure S63. ^{1}H- ^{13}C HMBC of **Pyr-PDI**.

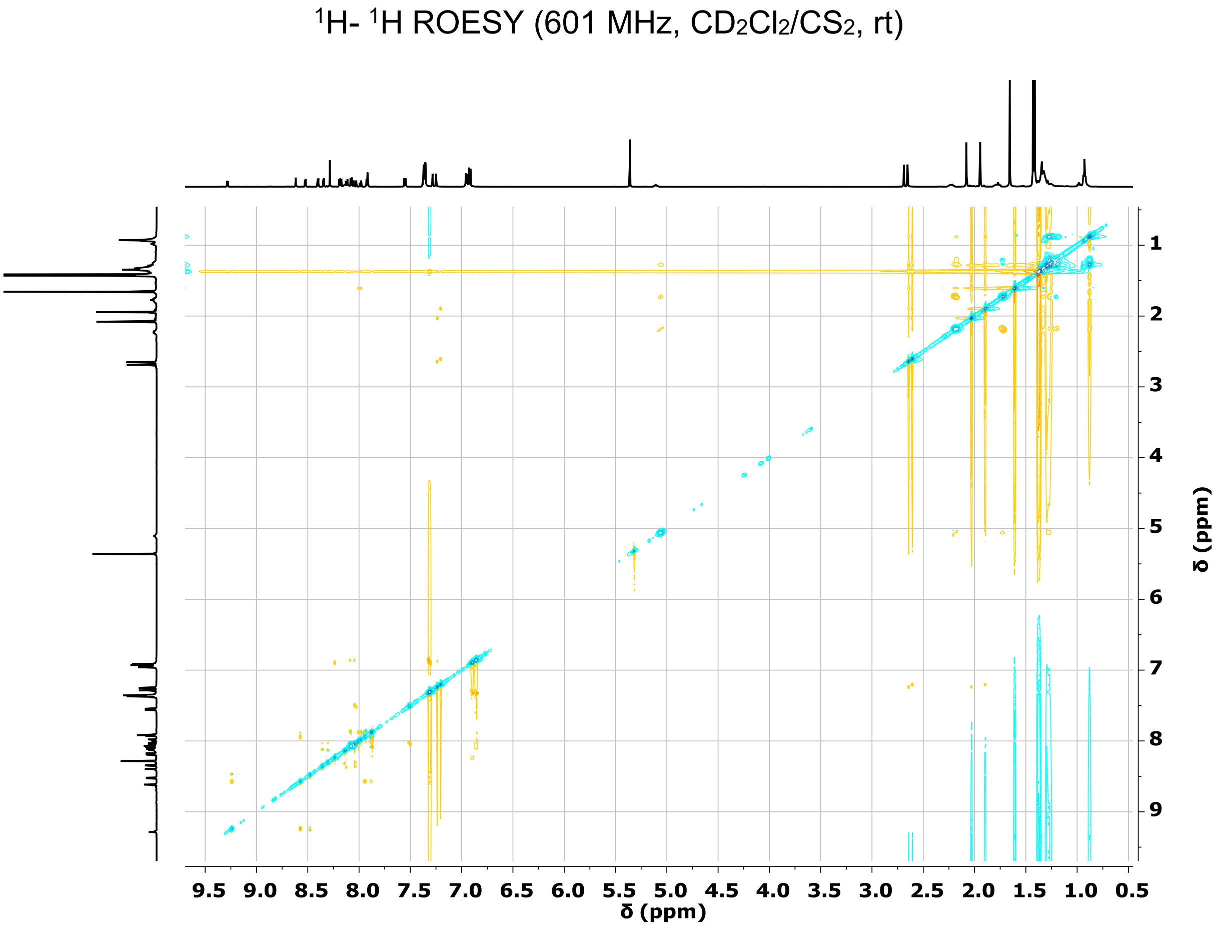

Figure S64. ^1H- ^1H ROESY of **Pyr-PDI**.

MS (MALDI)

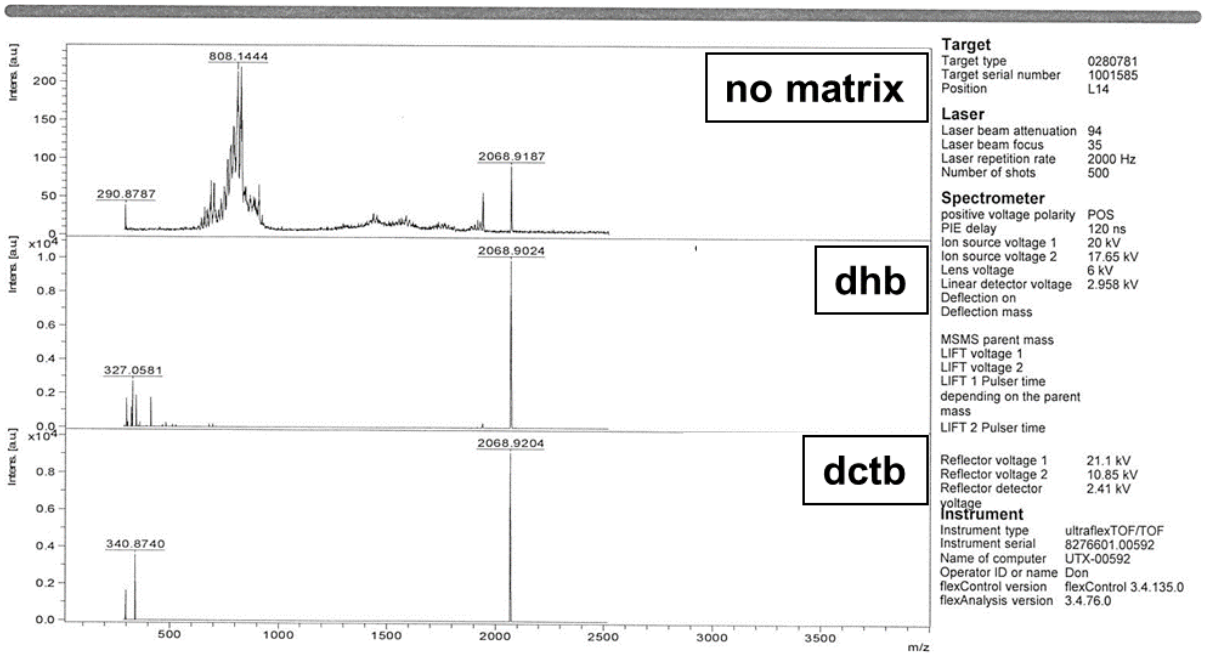

HRMS (MALDI)

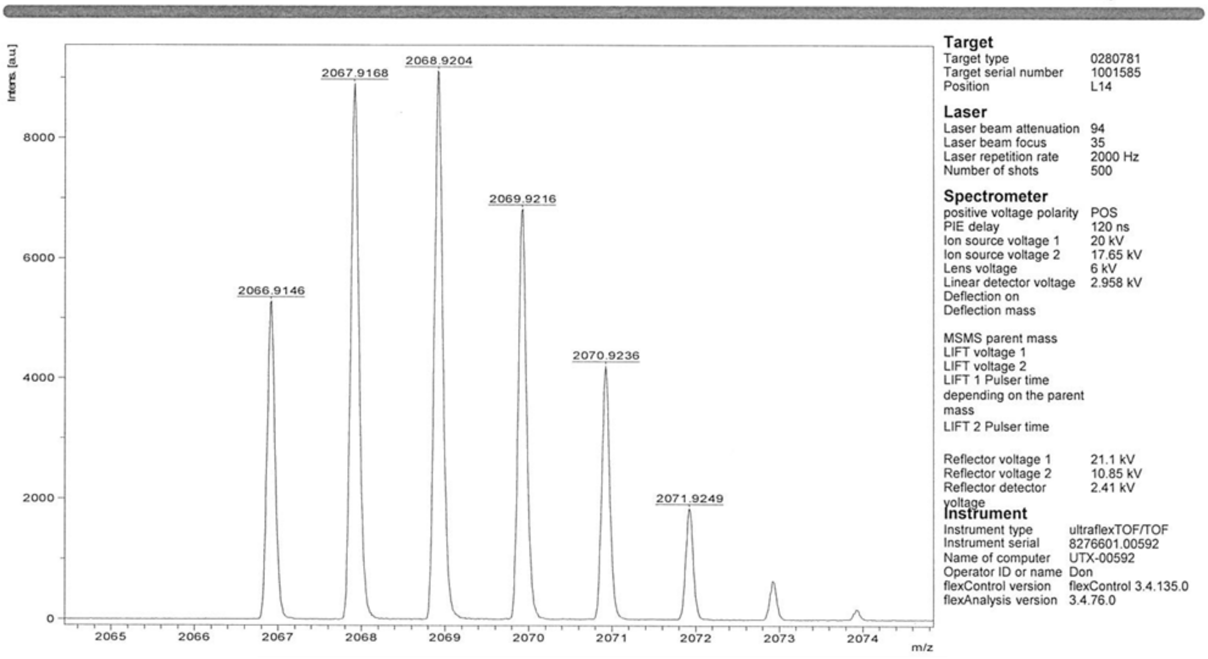

SmartFormula

Formula	Mass	Error	mSigma	DblEq	N rule	Electron Configuration
C 139 H 128 N 6 Ni O 8	2,066.9142	0.2354	54.2301	79.00	ok	odd

Figure S65. MS/HRMS (MALDI) of **Pyr-PDI**.

^{1}H NMR (601 MHz, CD_2Cl_2, rt)

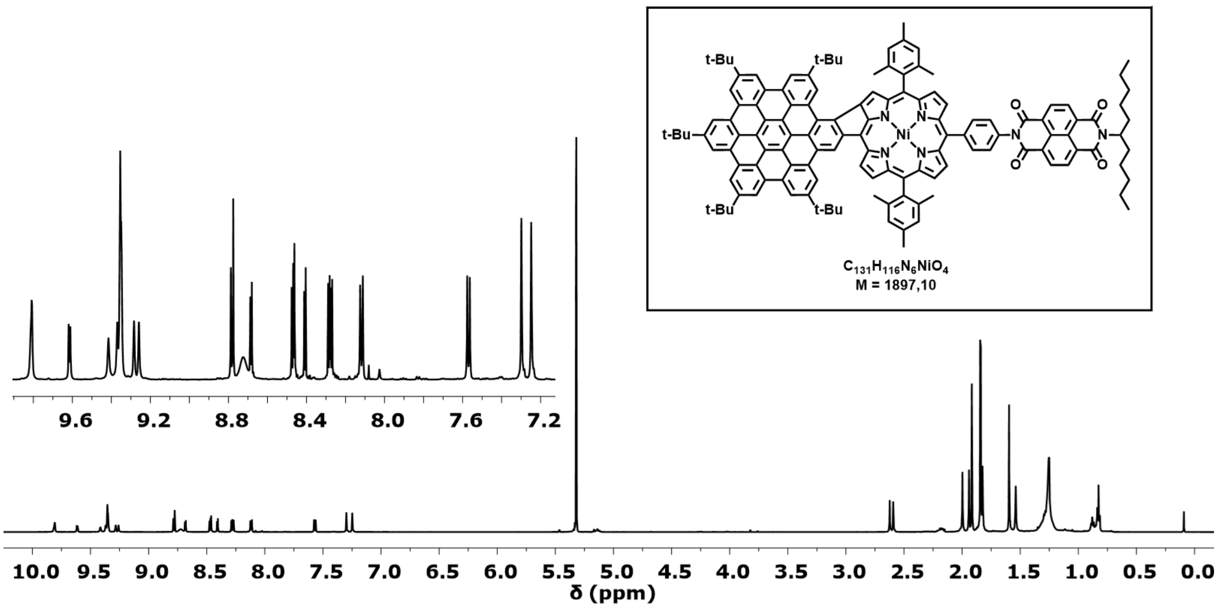

^{13}C NMR - DEPTQ135 (151 MHz, CD_2Cl_2, rt)

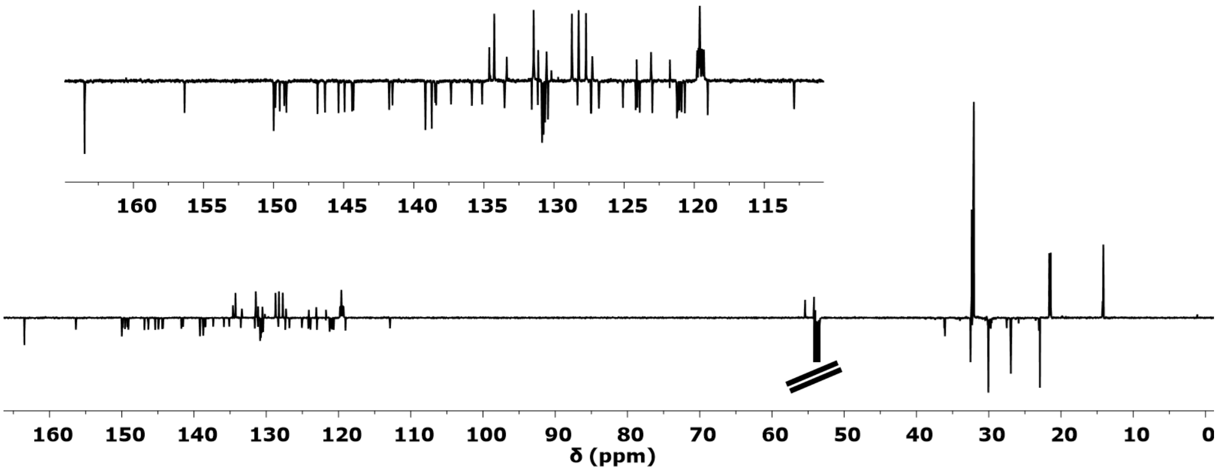

Figure S66. ^{1}H and ^{13}C NMR (DEPTQ135) of **HBC-NDI**.

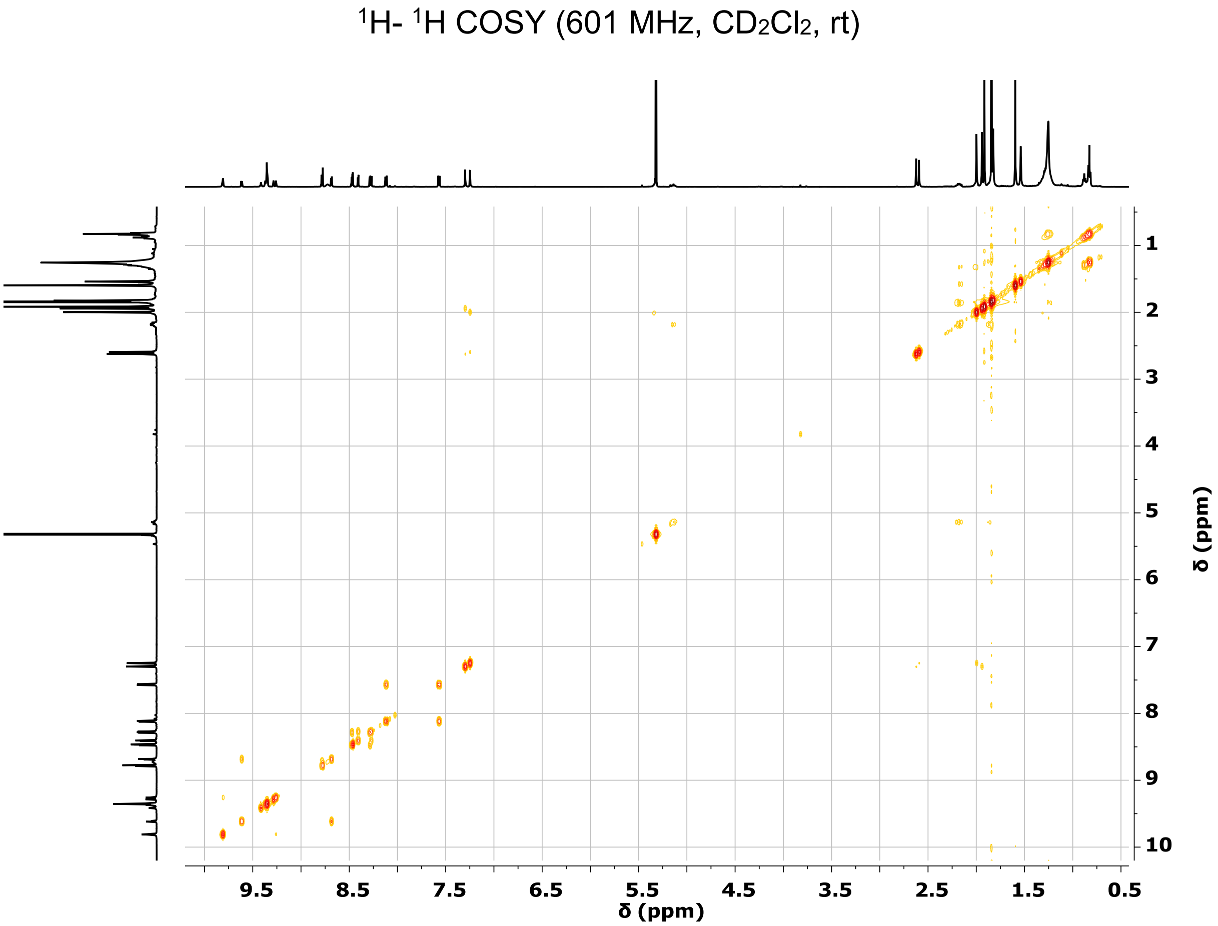

Figure S67. ^1H- ^1H COSY of **HBC-NDI**.

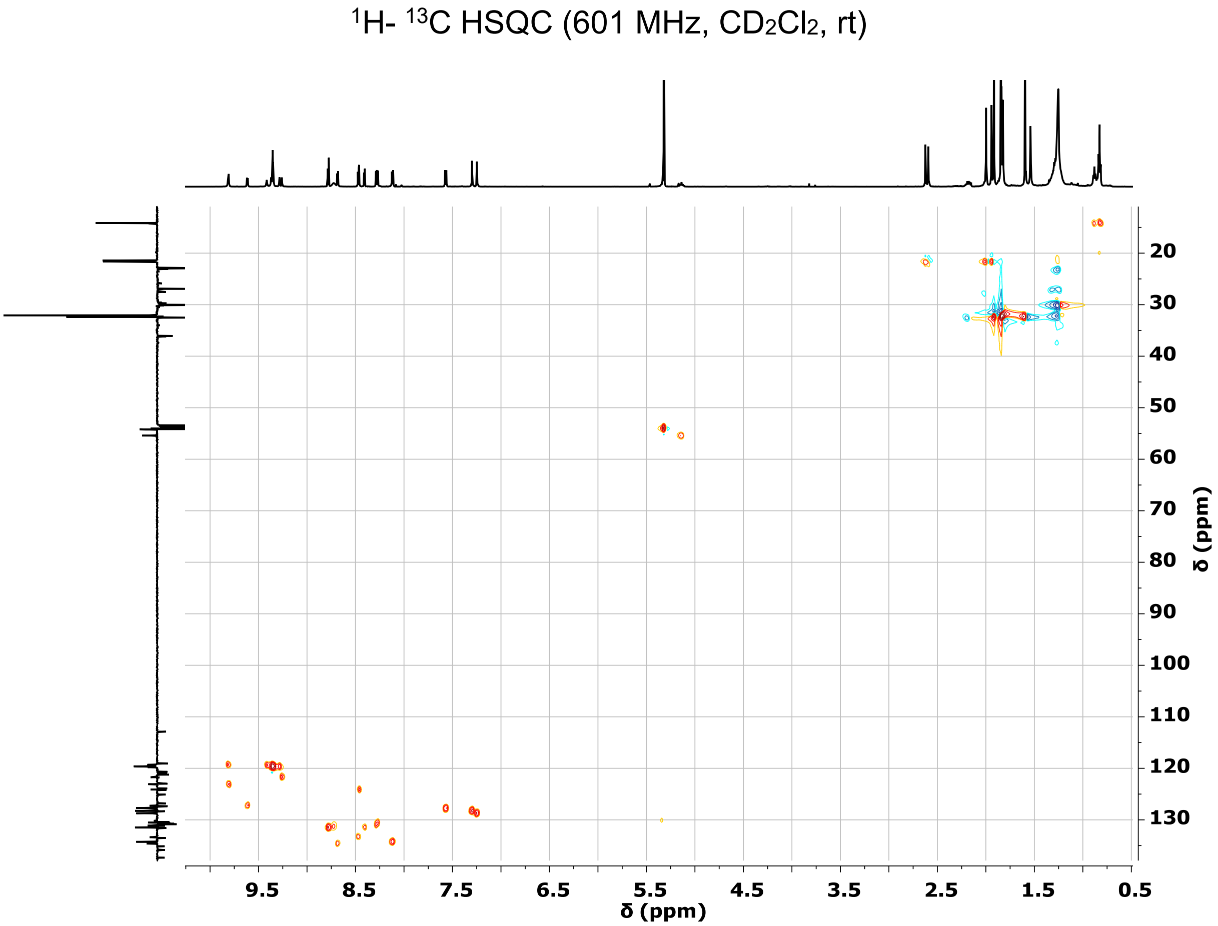

Figure S68. ^{1}H- ^{13}C HSQC of **HBC-NDI**.

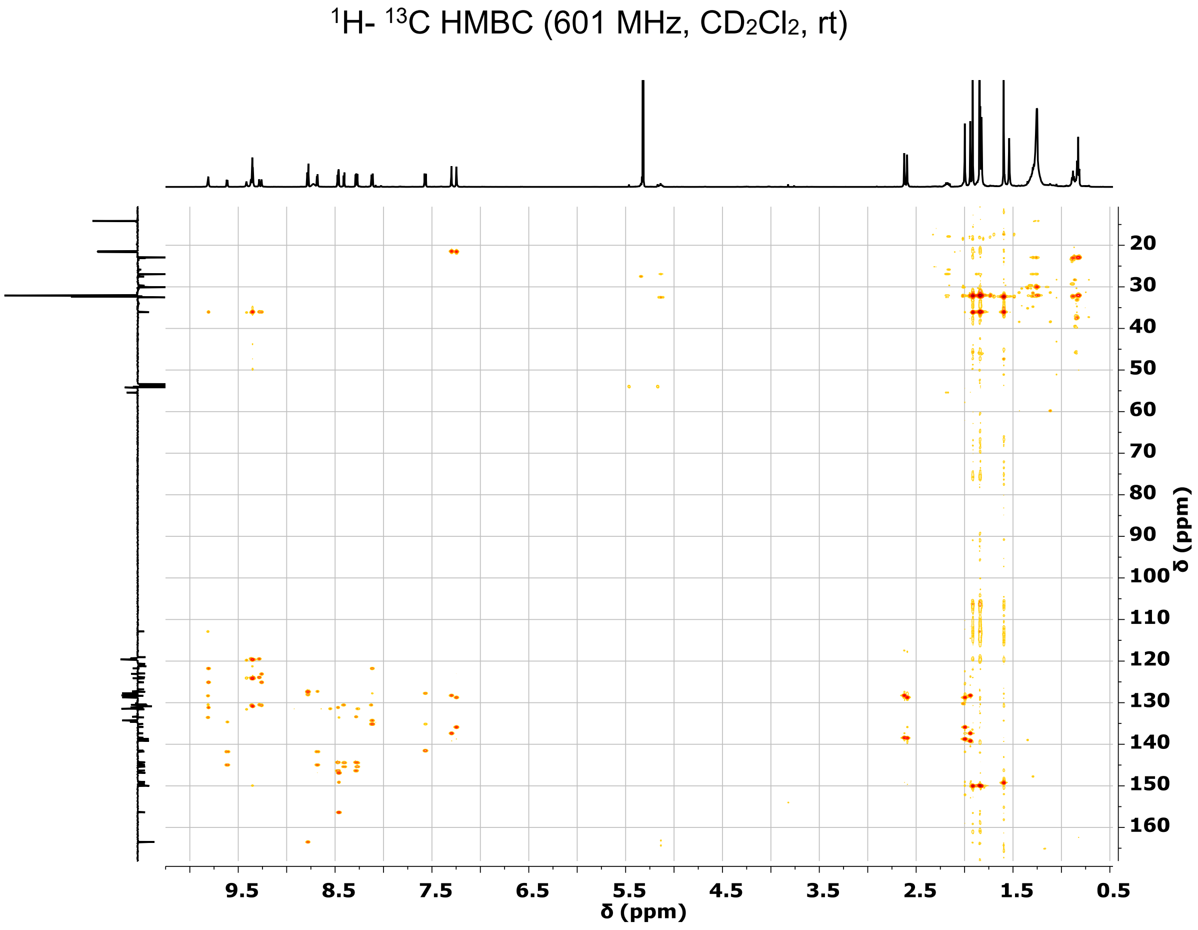

Figure S69. ¹H- ¹³C HMBC of **HBC-NDI**.

MS (MALDI)

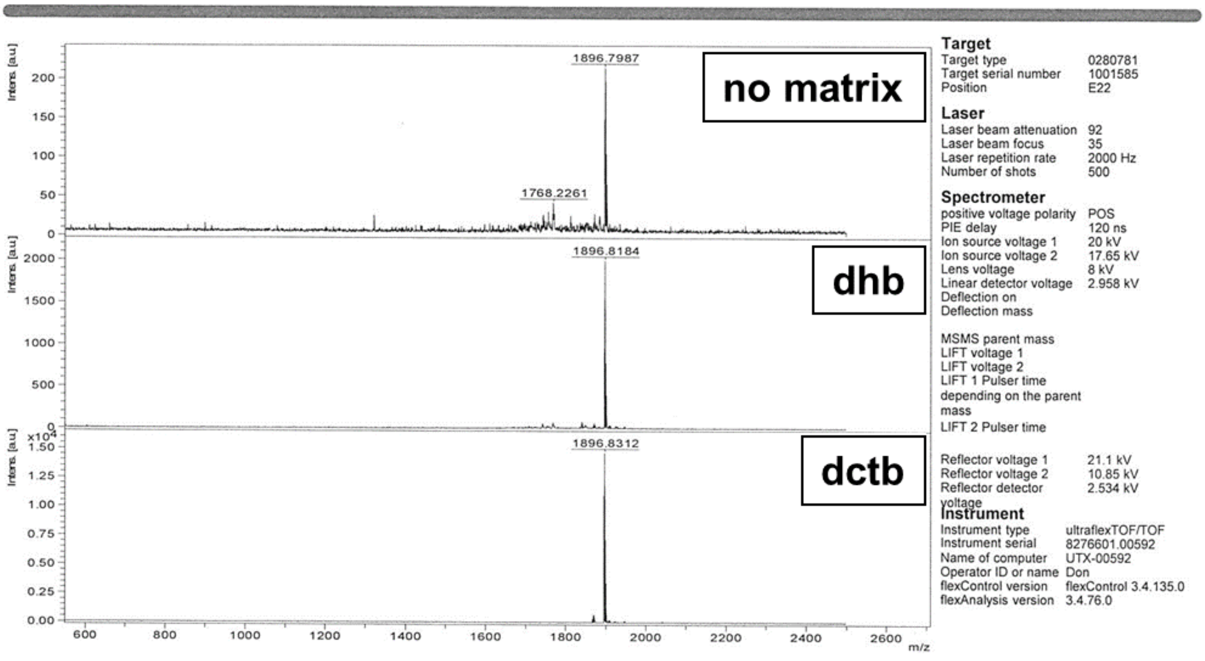

HRMS (MALDI)

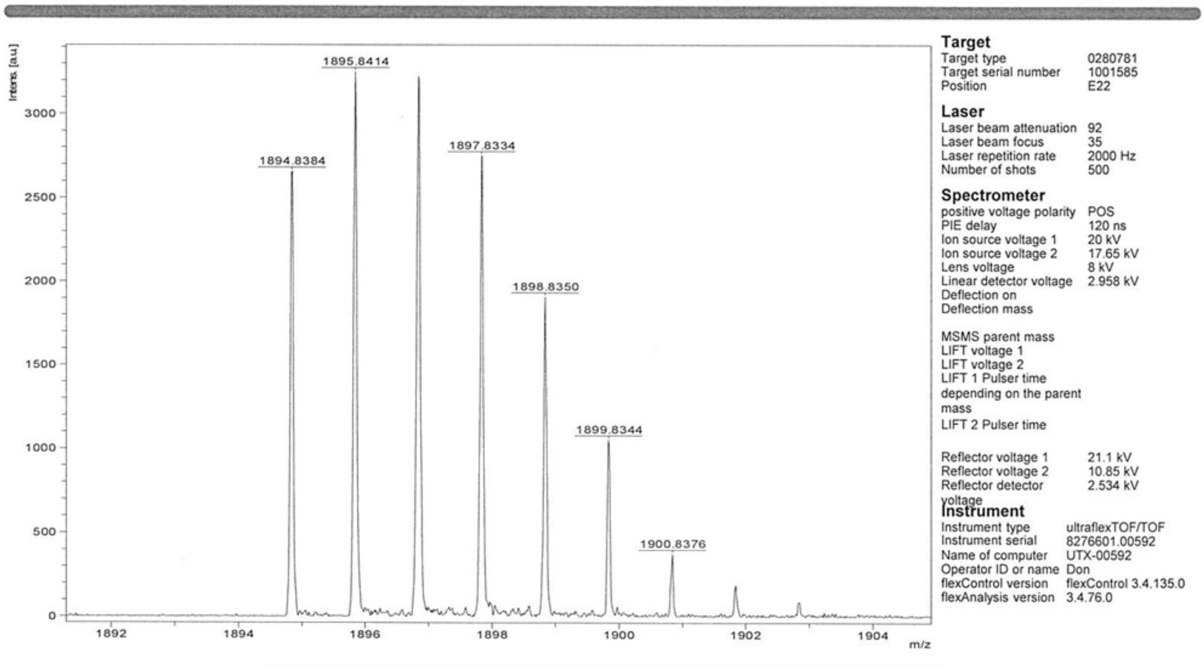

SmartFormula

Formula	Mass	Error	mSigma	DblEq	N rule	Electron Configuration
C 131 H 116 N 6 Ni O 4	1,894.8406	1.1652	72.2838	77.00	ok	odd

Figure S70. MS/HRMS (MALDI) of **HBC-NDI.**

^1H NMR (601 MHz, CD_2Cl_2, rt)

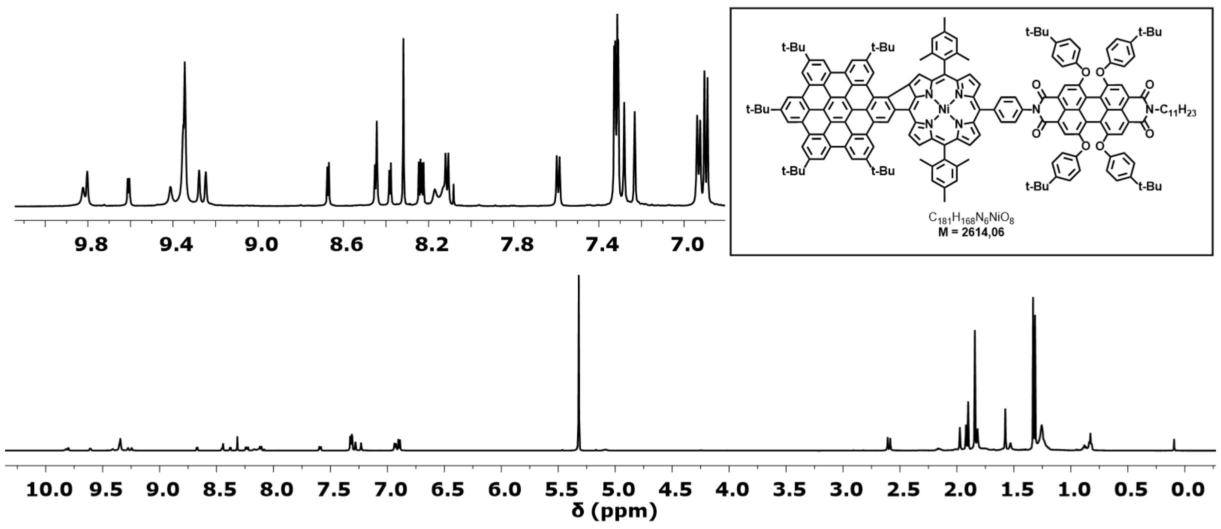

^{13}C NMR - DEPTQ135 (151 MHz, CD_2Cl_2, rt)

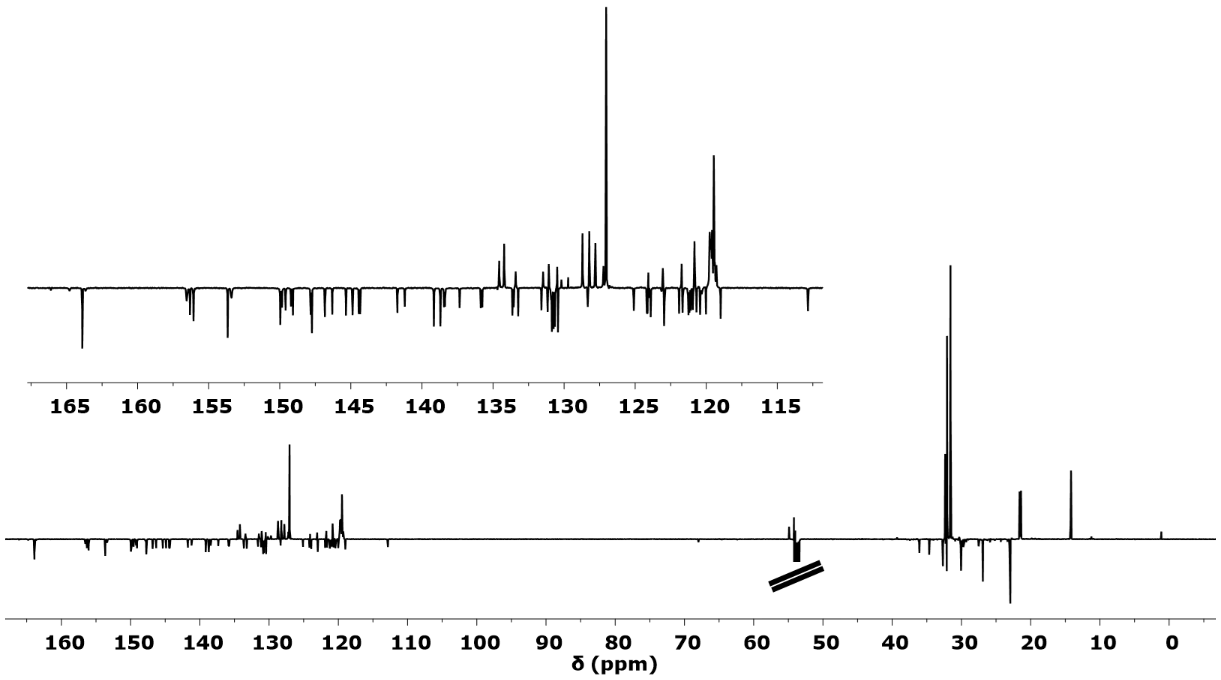

Figure S71. ^1H and ^{13}C NMR (DEPTQ135) of **HBC-PDI**.

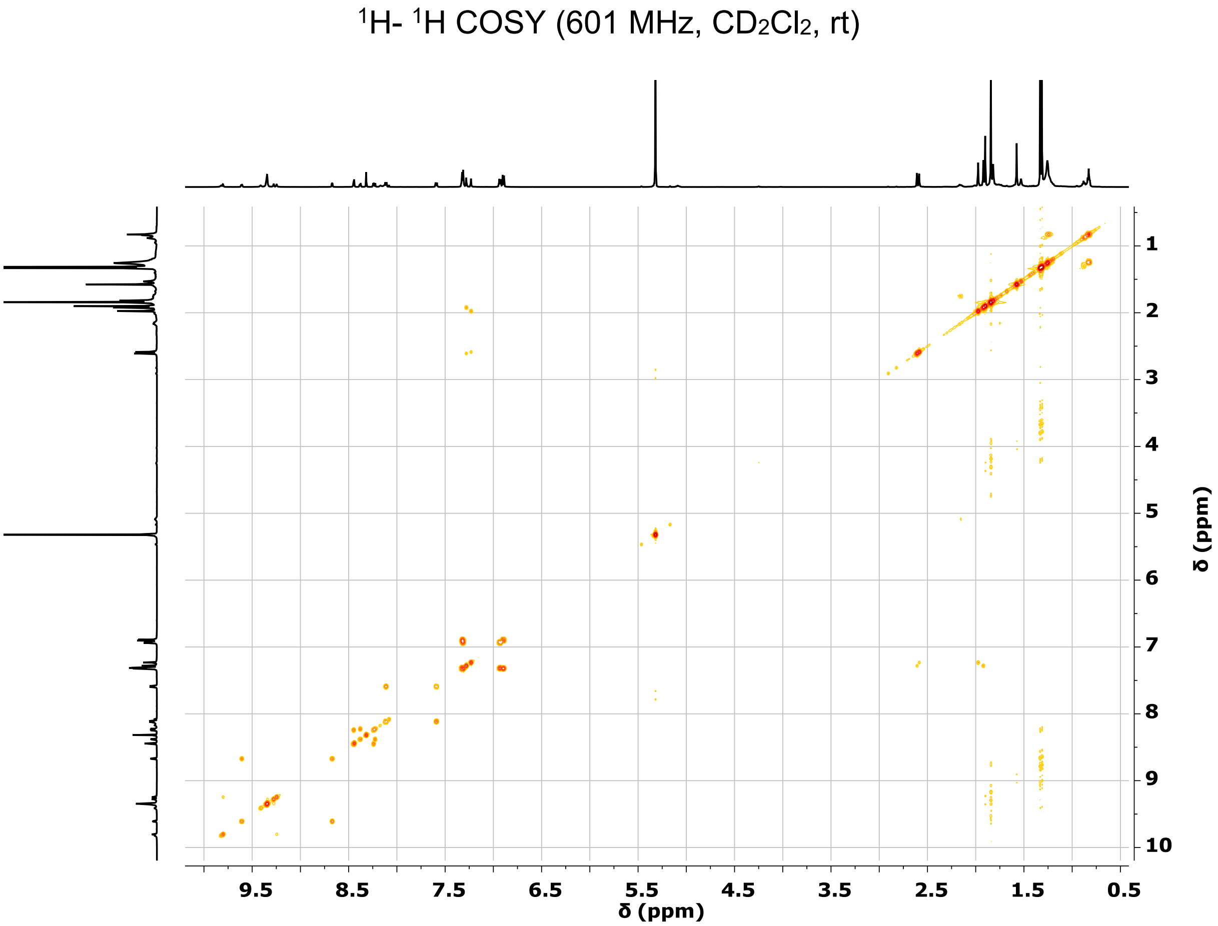

Figure S72. ^{1}H- ^{1}H COSY of **HBC-PDI**.

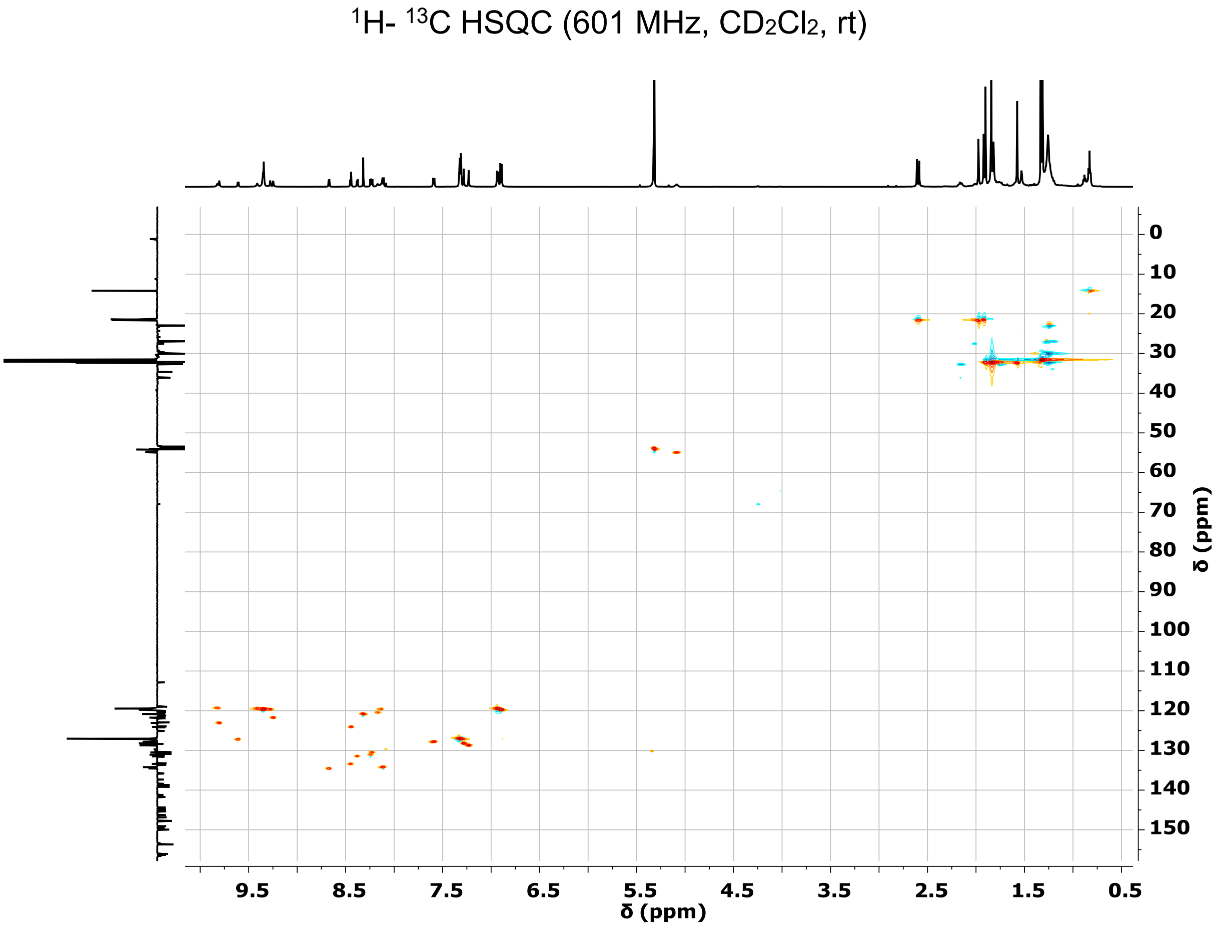

Figure S73. ^{1}H- ^{13}C HSQC of **HBC-PDI**.

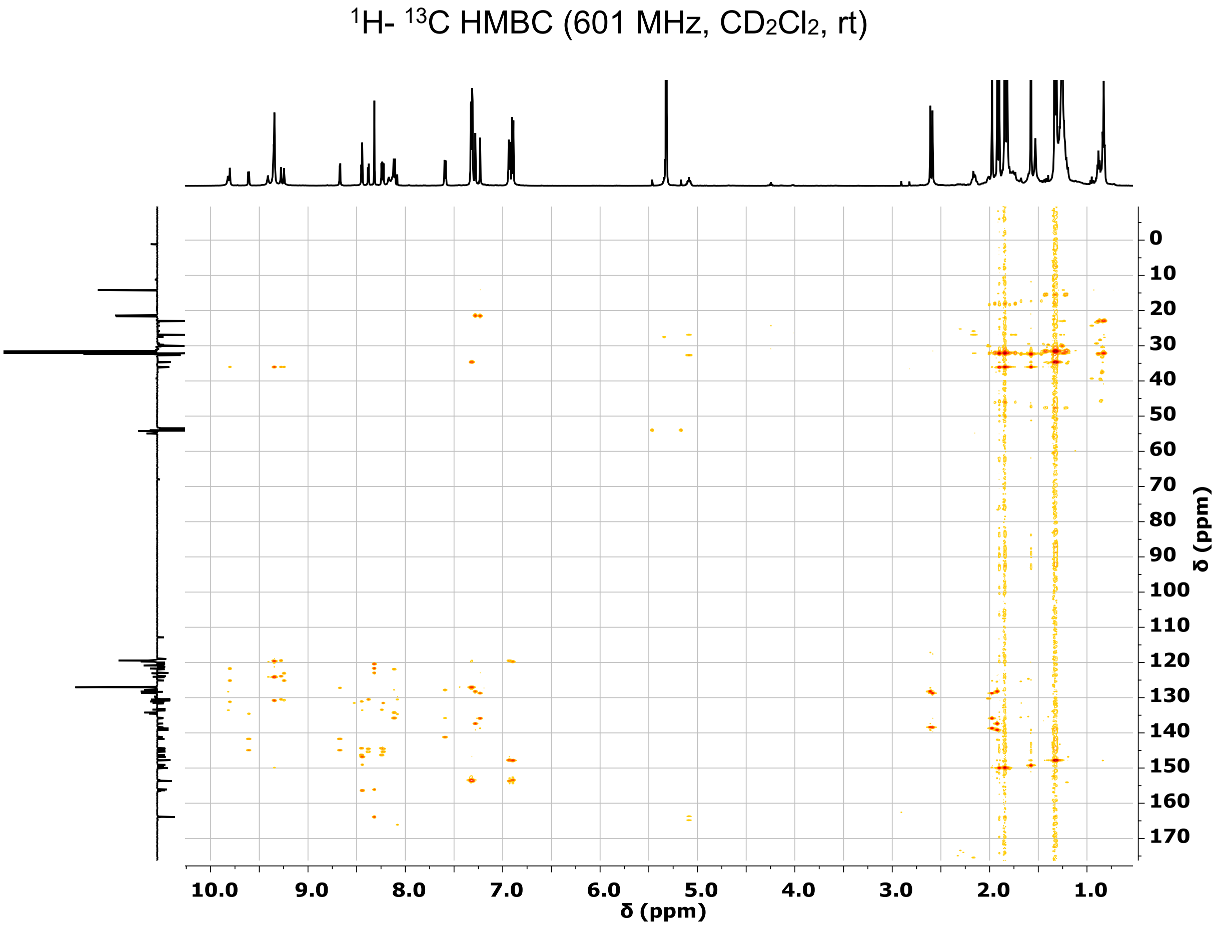

Figure S74. ¹H- ¹³C HMBC of **HBC-PDI**.

MS (MALDI)

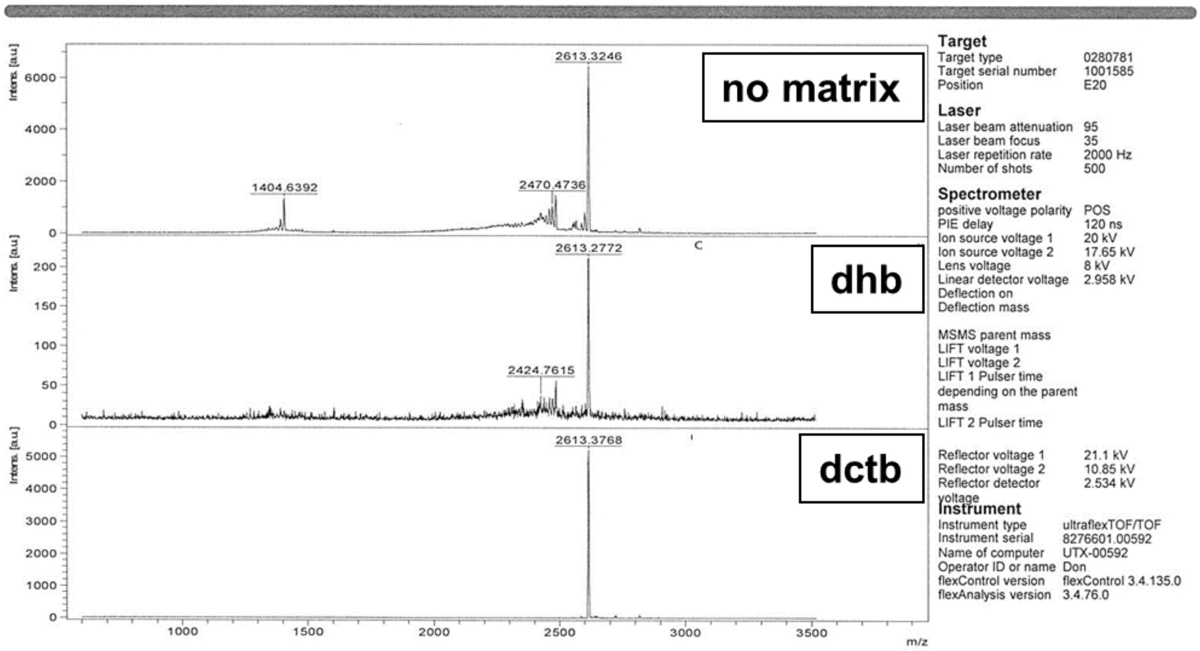

HRMS (MALDI)

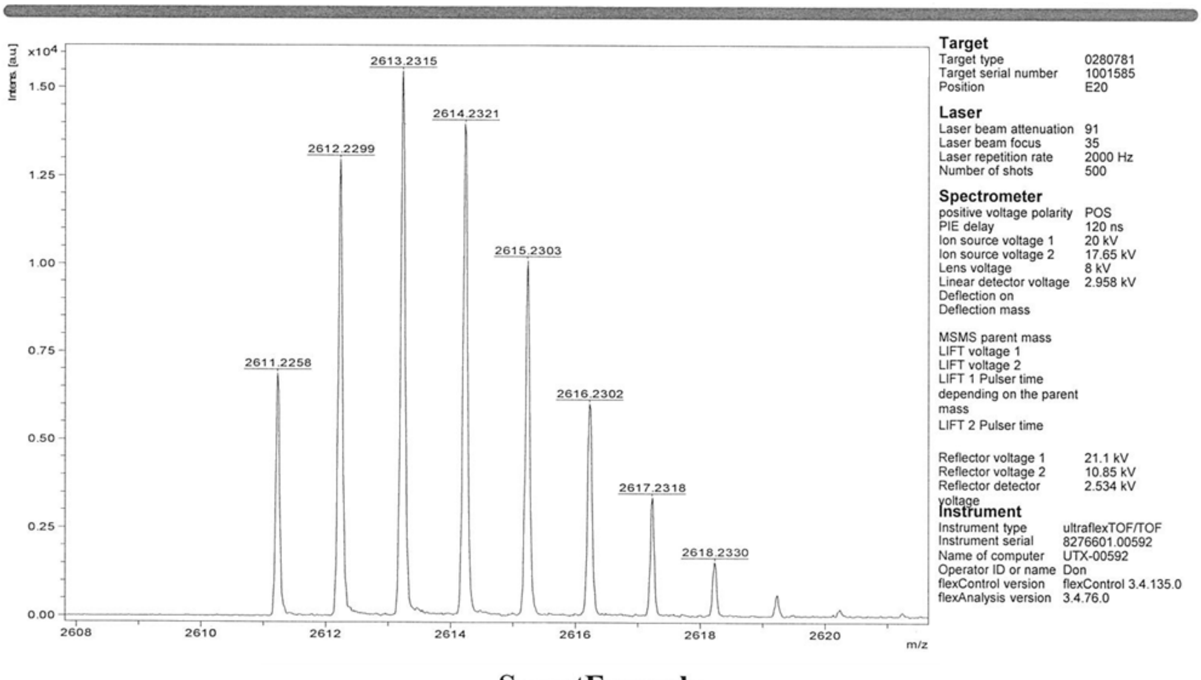

SmartFormula

Formula	Mass	Error	mSigma	DblEq	N rule	Electron Configuration
C 181 H 168 N 6 Ni O 8	2,611.2272	0.5386	18.2456	101.00	ok	odd

Figure S75. MS/HRMS (MALDI) of **HBC-PDI.**

^{1}H NMR (601 MHz, CD_2Cl_2, rt)

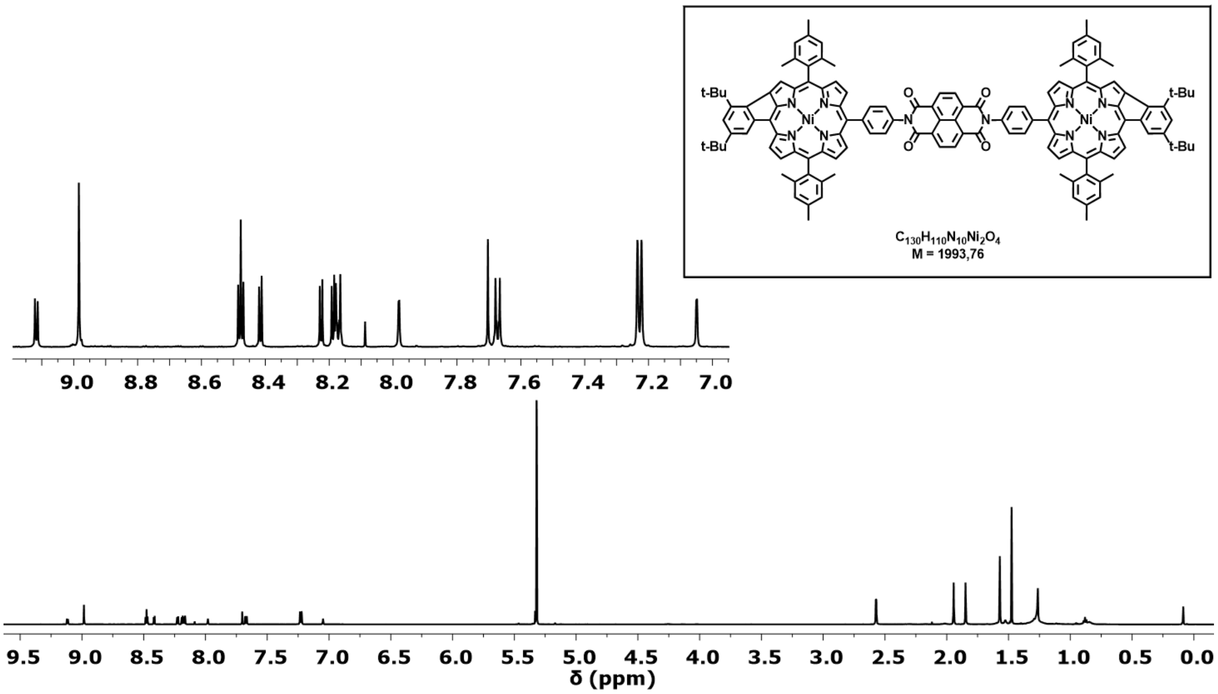

^{13}C NMR - DEPTQ135 (151 MHz, CD_2Cl_2, rt)

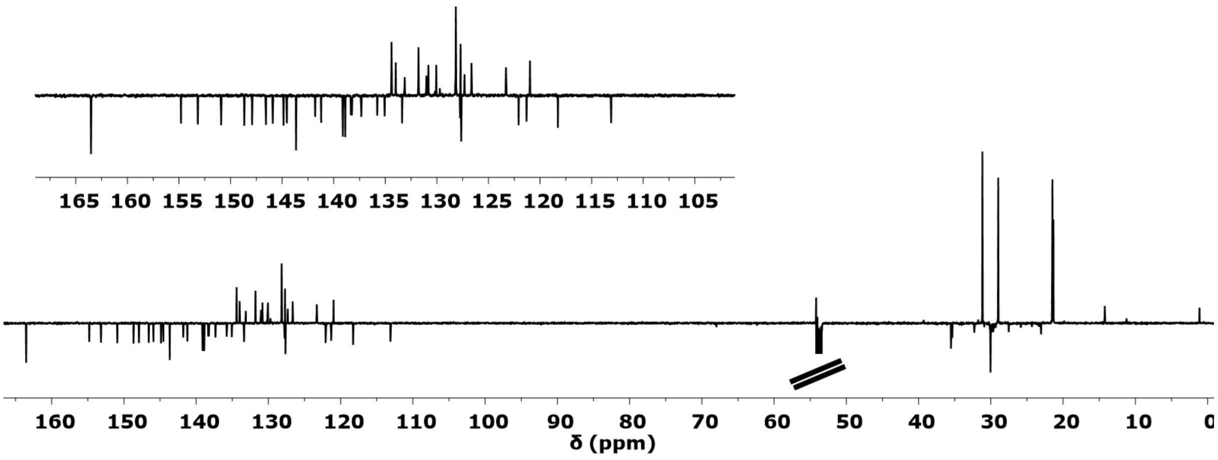

Figure S76. ^{1}H and ^{13}C NMR (DEPTQ135) of **Ph-NDI-Ph**.

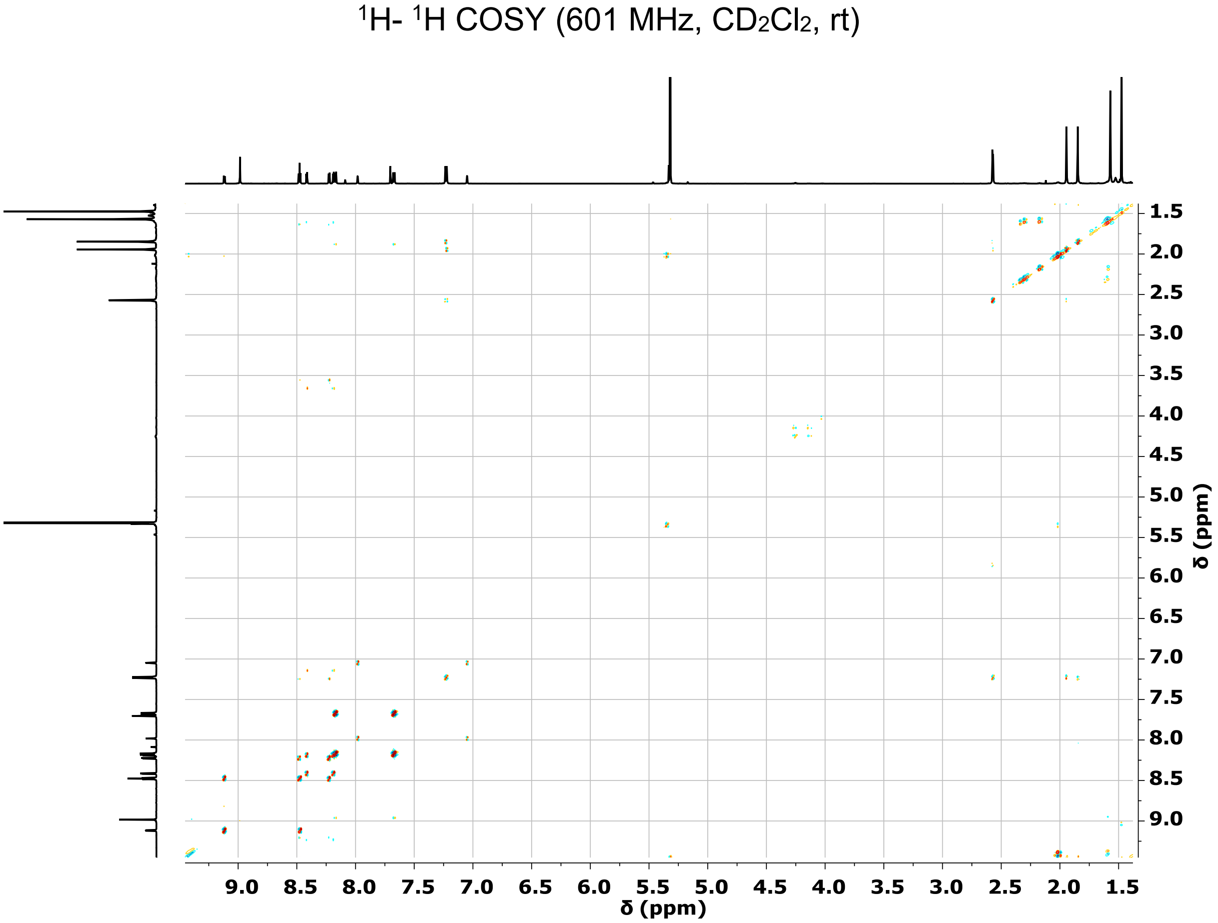

Figure S77. ^{1}H- ^{1}H COSY of **Ph-NDI-Ph.**

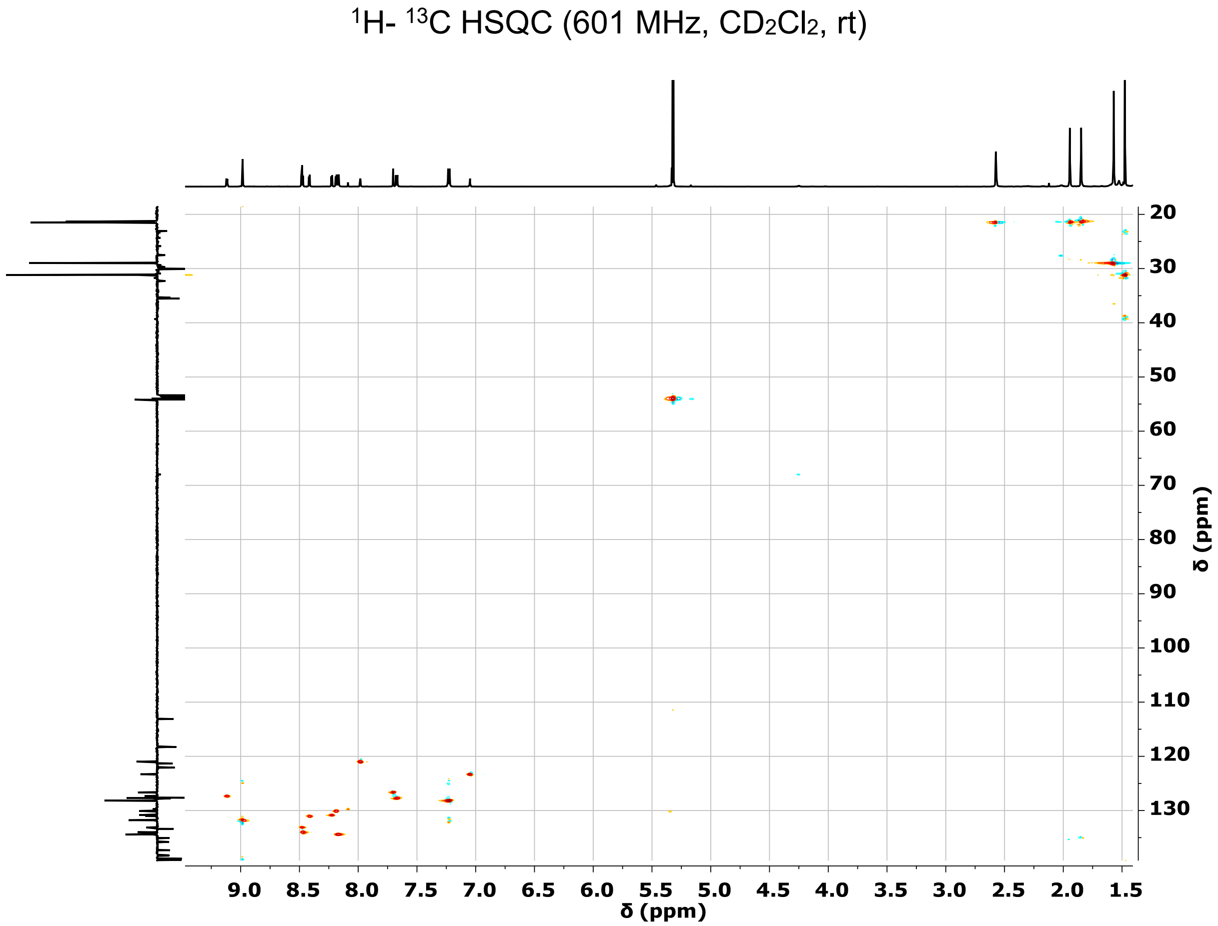

Figure S78. ¹H- ¹³C HSQC of **Ph-NDI-Ph**.

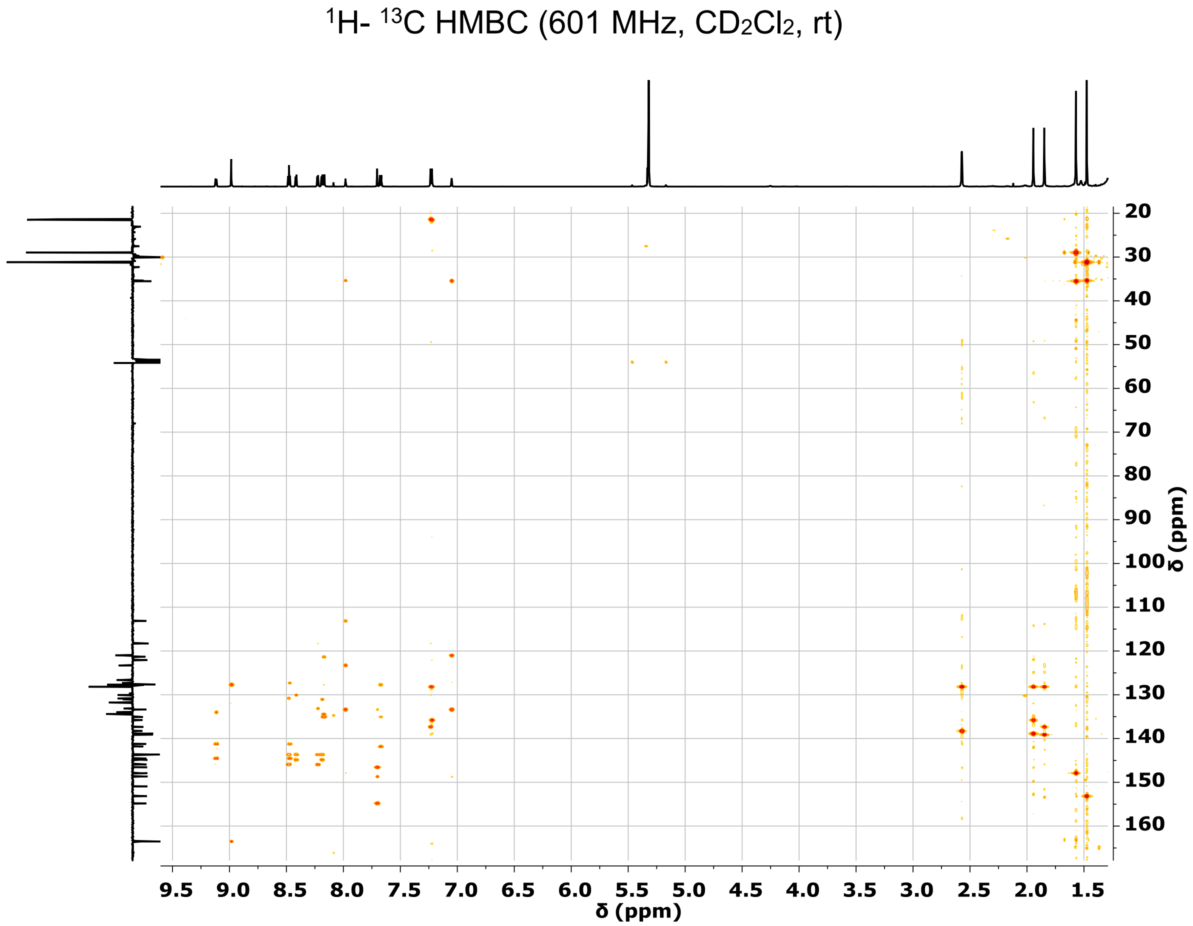

Figure S79. ¹H- ¹³C HMBC of **Ph-NDI-Ph**.

^{1}H- ^{1}H ROESY (601 MHz, CD_2Cl_2, rt)

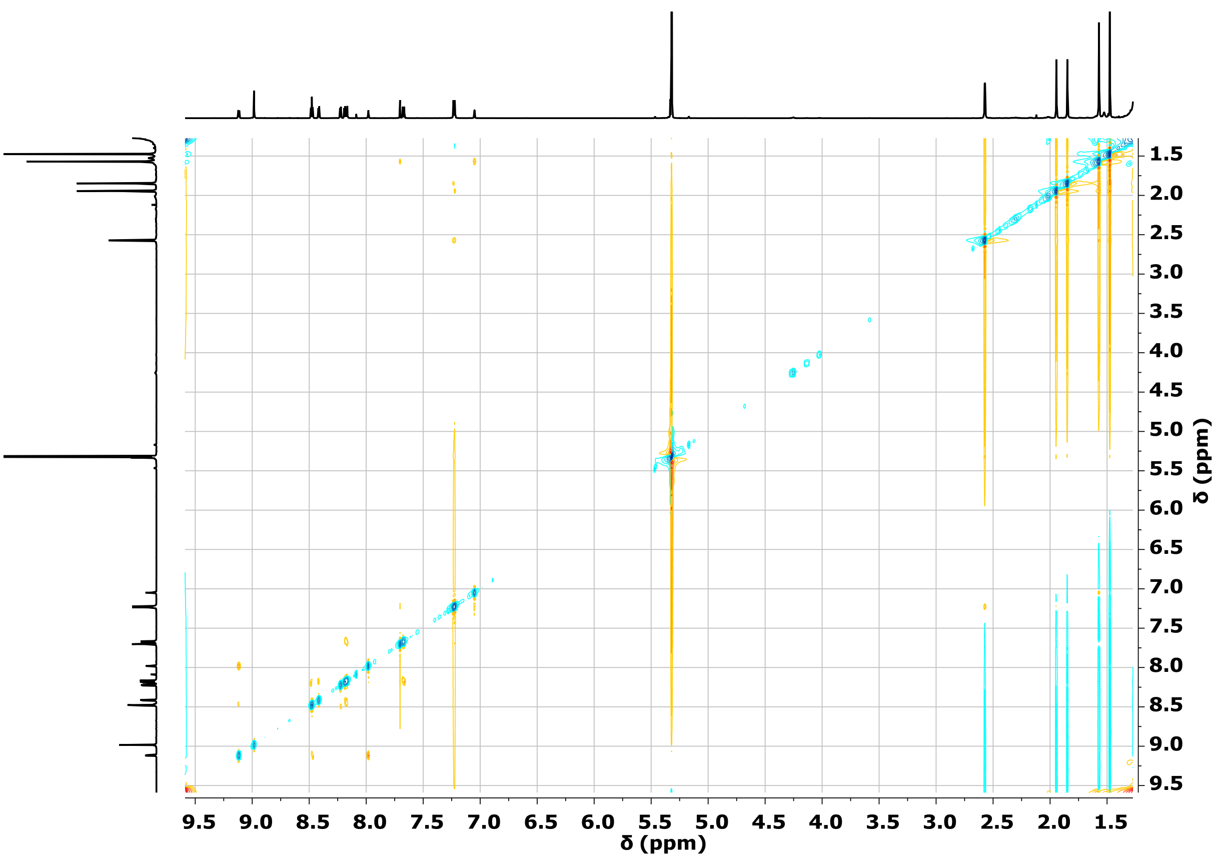

Figure S80. [1]H- [1]H ROESY of **Ph-NDI-Ph**.

MS (MALDI)

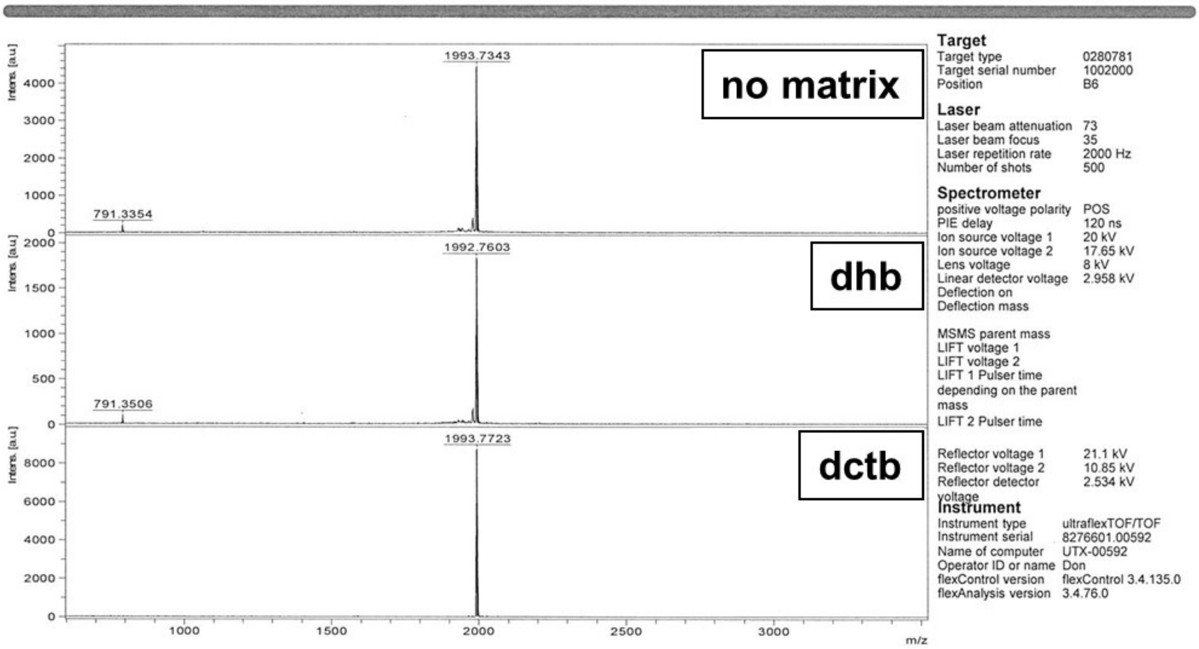

HRMS (MALDI)

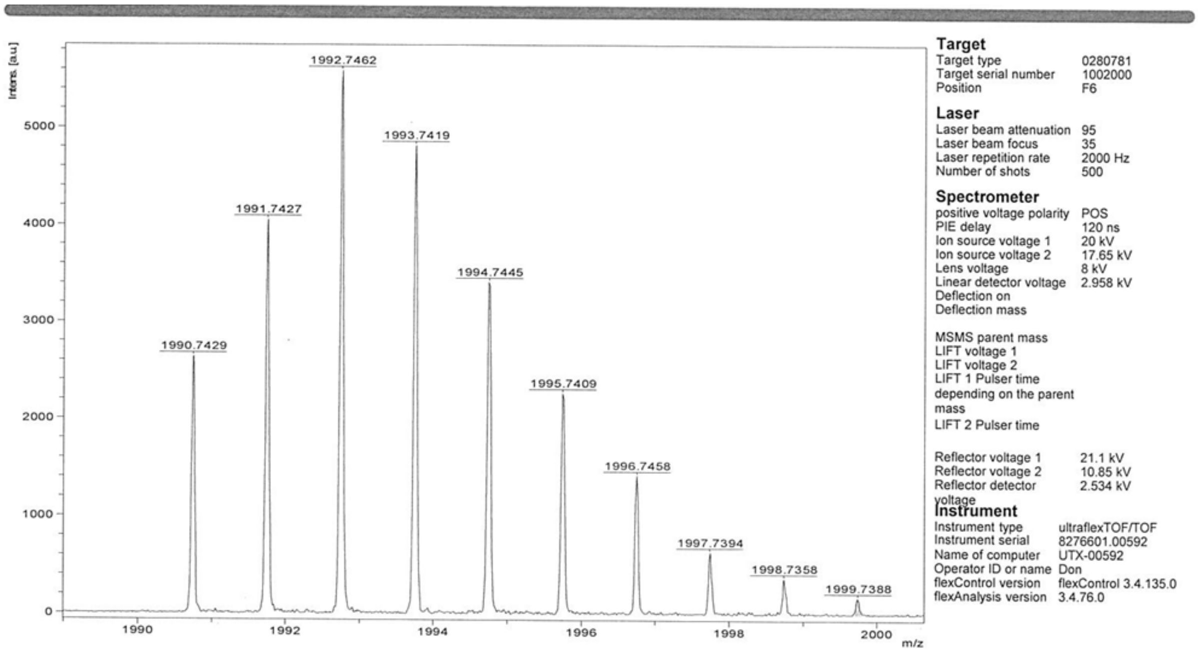

SmartFormula

Formula	Mass	Error	mSigma	DblEq	N rule	Electron Configuration
C 130 H 110 N 10 Ni 2 O 4	1,990.7413	0.7863	52.6432	81.00	ok	odd

Figure S81. MS/HRMS (MALDI) of **Ph-NDI-Ph**.

^{1}H NMR (601 MHz, CD_2Cl_2, rt)

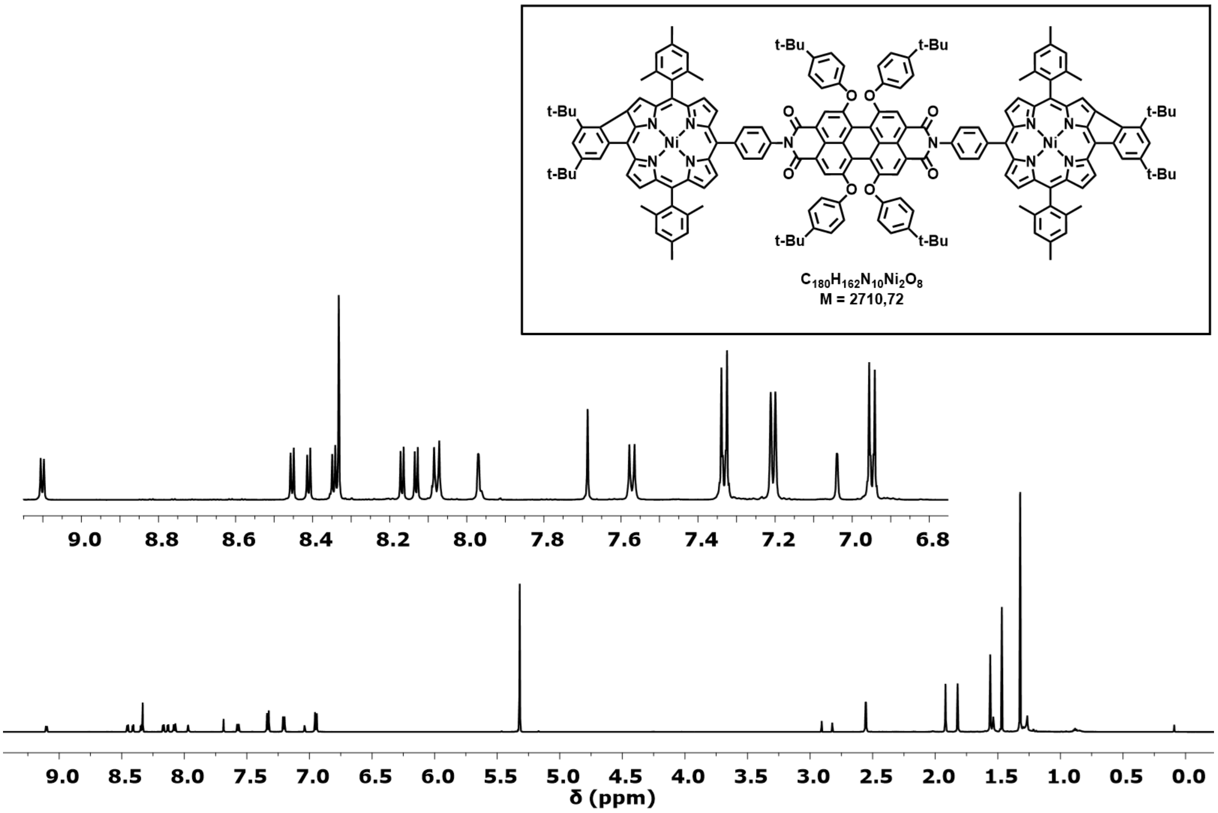

^{13}C NMR - DEPTQ135 (151 MHz, CD_2Cl_2, rt)

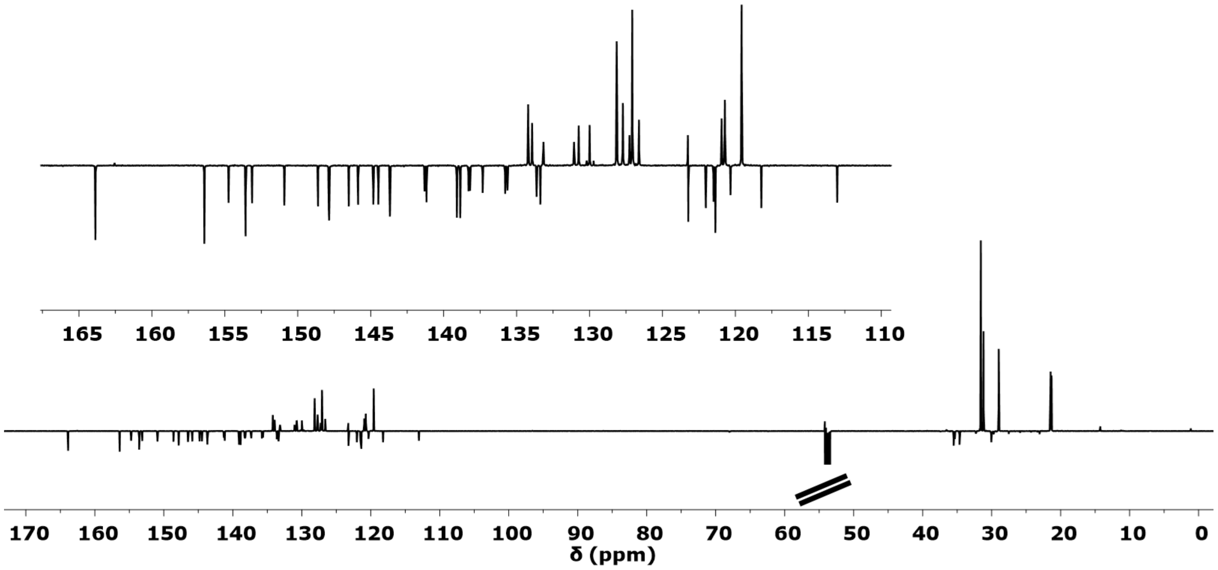

Figure S82. ^{1}H and ^{13}C NMR (DEPTQ135) of **Ph-PDI-Ph**.

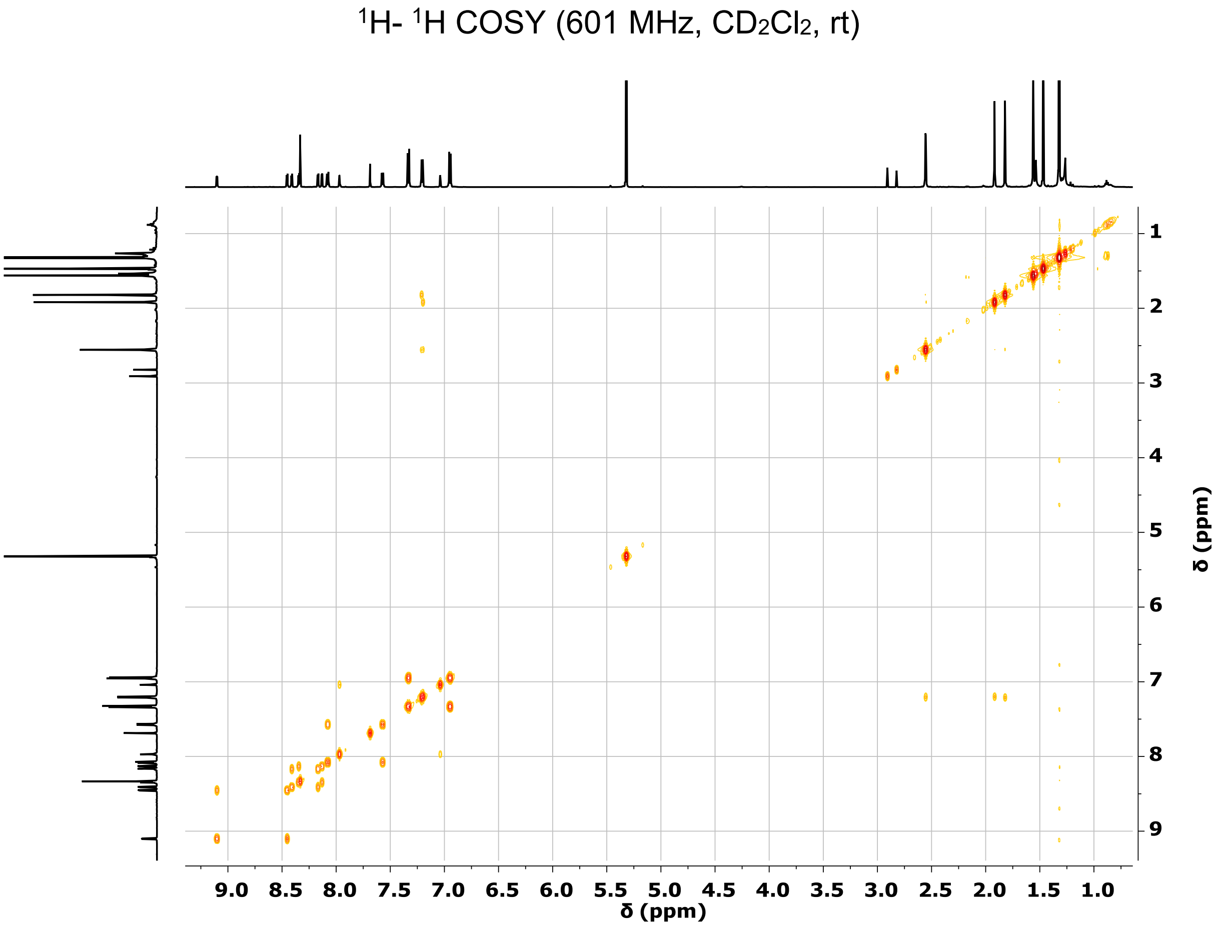

Figure S83. ^{1}H- ^{1}H COSY of **Ph-PDI-Ph**.

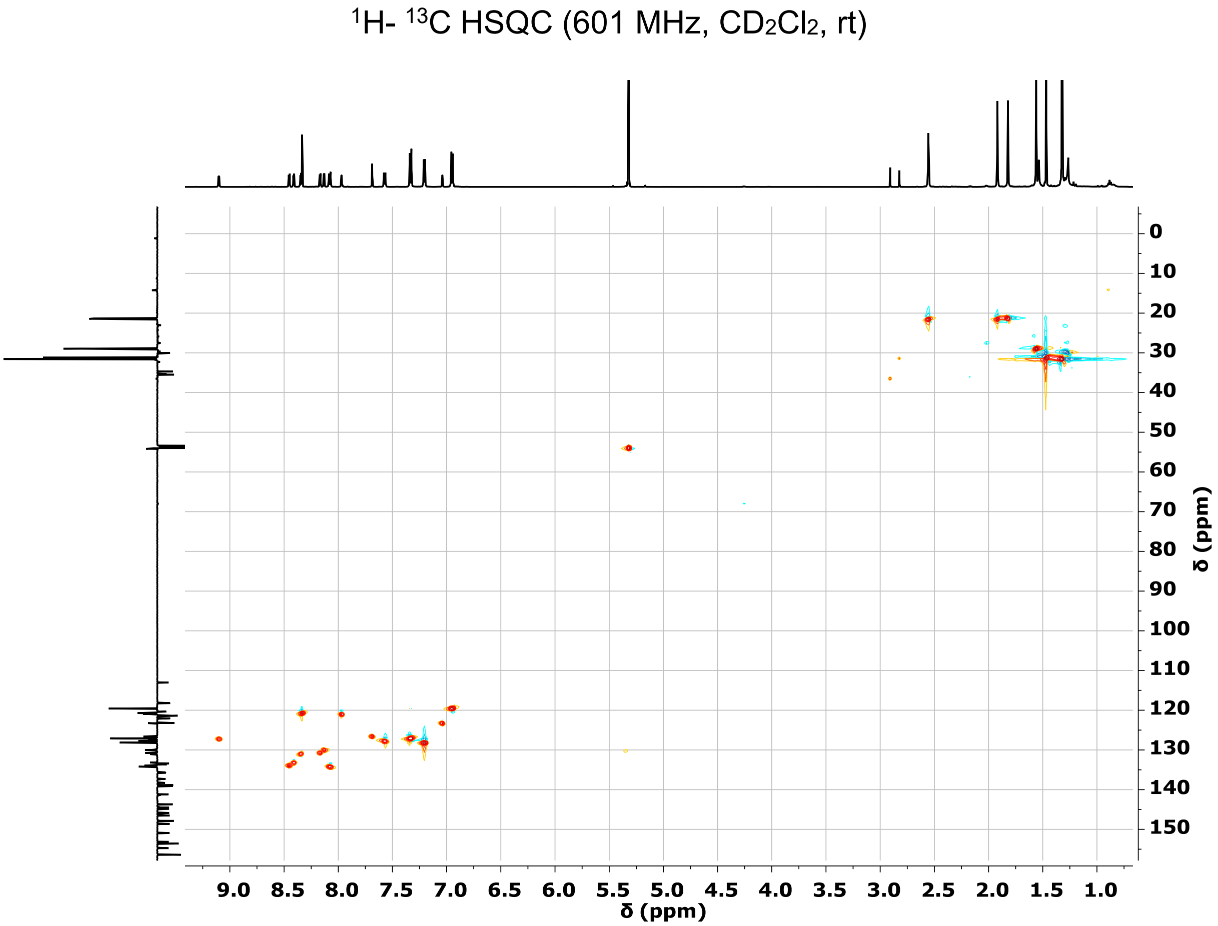

Figure S84. ^{1}H- ^{13}C HSQC of **Ph-PDI-Ph**.

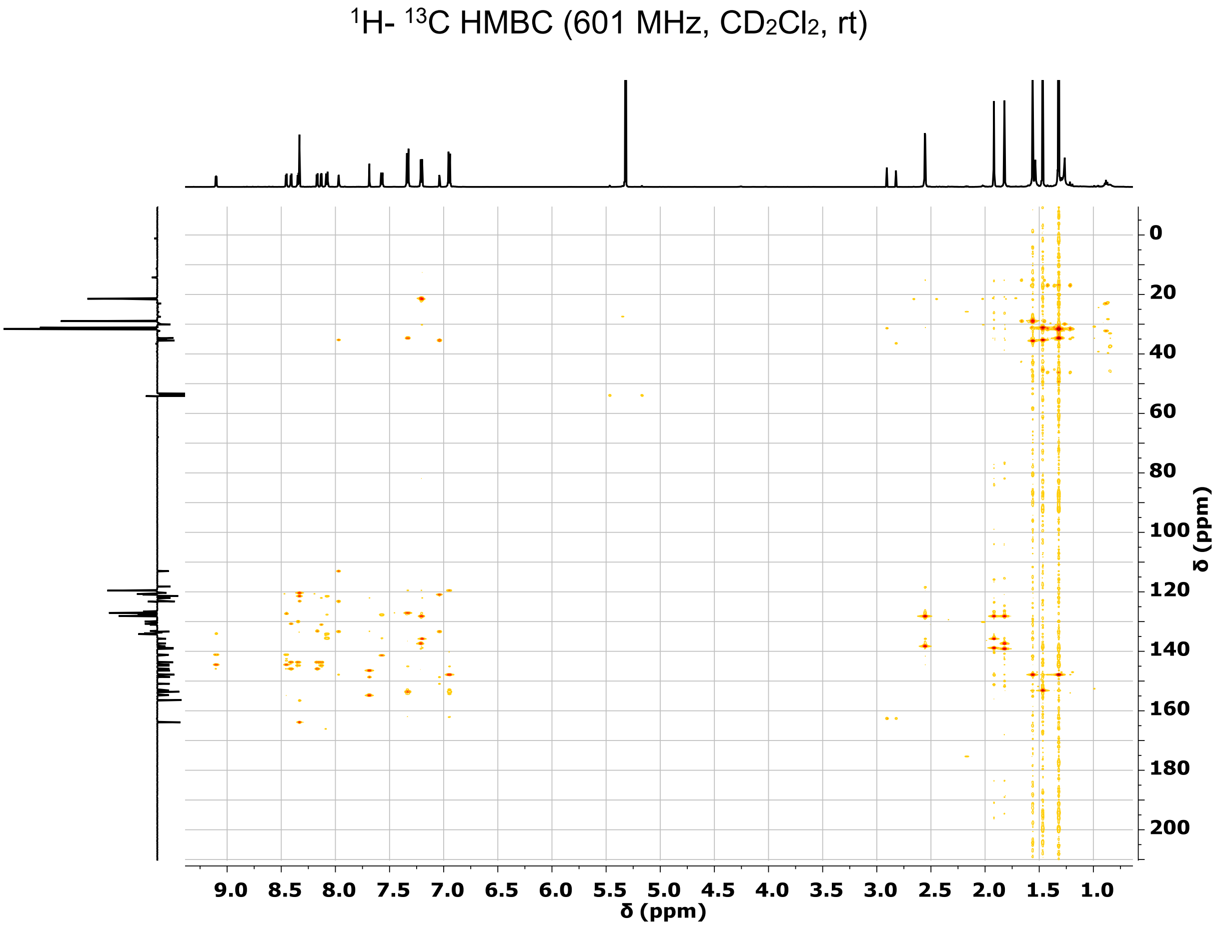

Figure S85. ^{1}H- ^{13}C HMBC of **Ph-PDI-Ph**.

MS (MALDI)

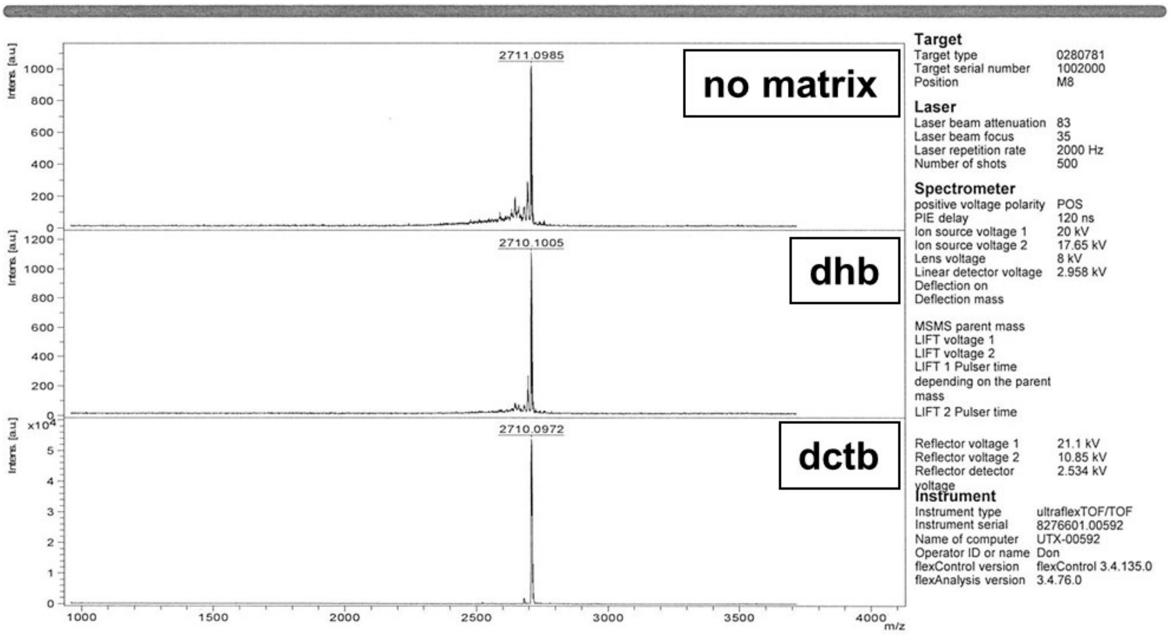

HRMS (MALDI)

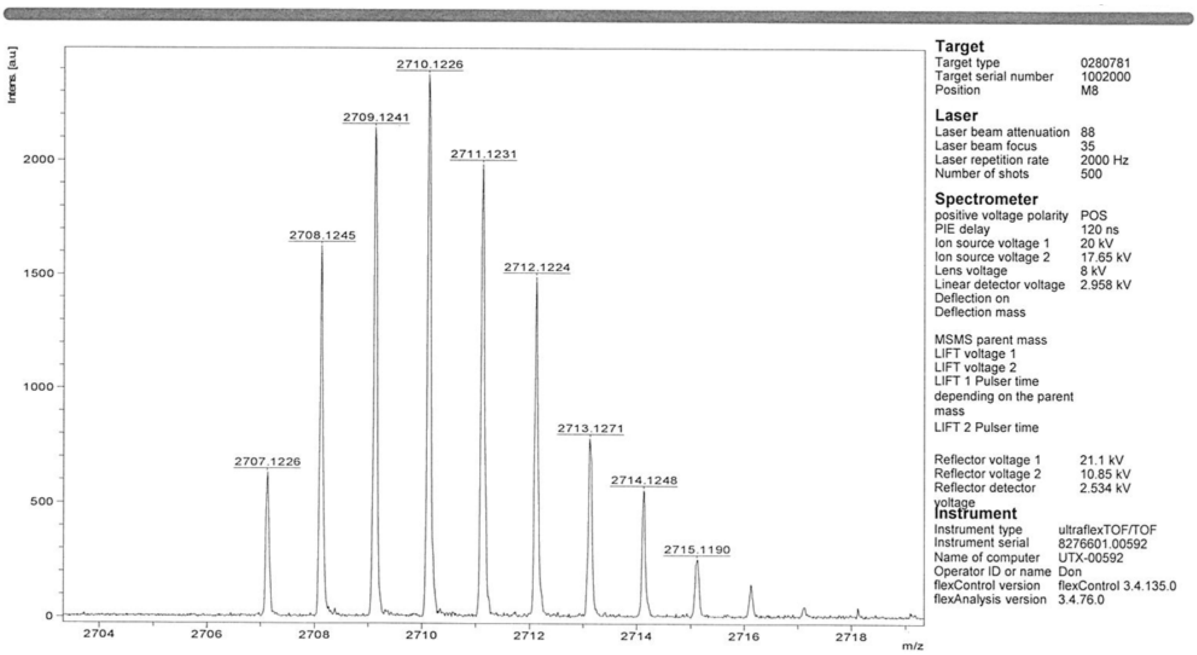

SmartFormula

Formula	Mass	Error	mSigma	DblEq	N rule	Electron Configuration
C 180 H 162 N 10 Ni 2 O 8	2,707.1278	1.9513	49.1967	105.00	ok	odd

Figure S86. MS/HRMS (MALDI) of **Ph-PDI-Ph**.

^{1}H NMR (601 MHz, CD_2Cl_2/CS_2, rt)

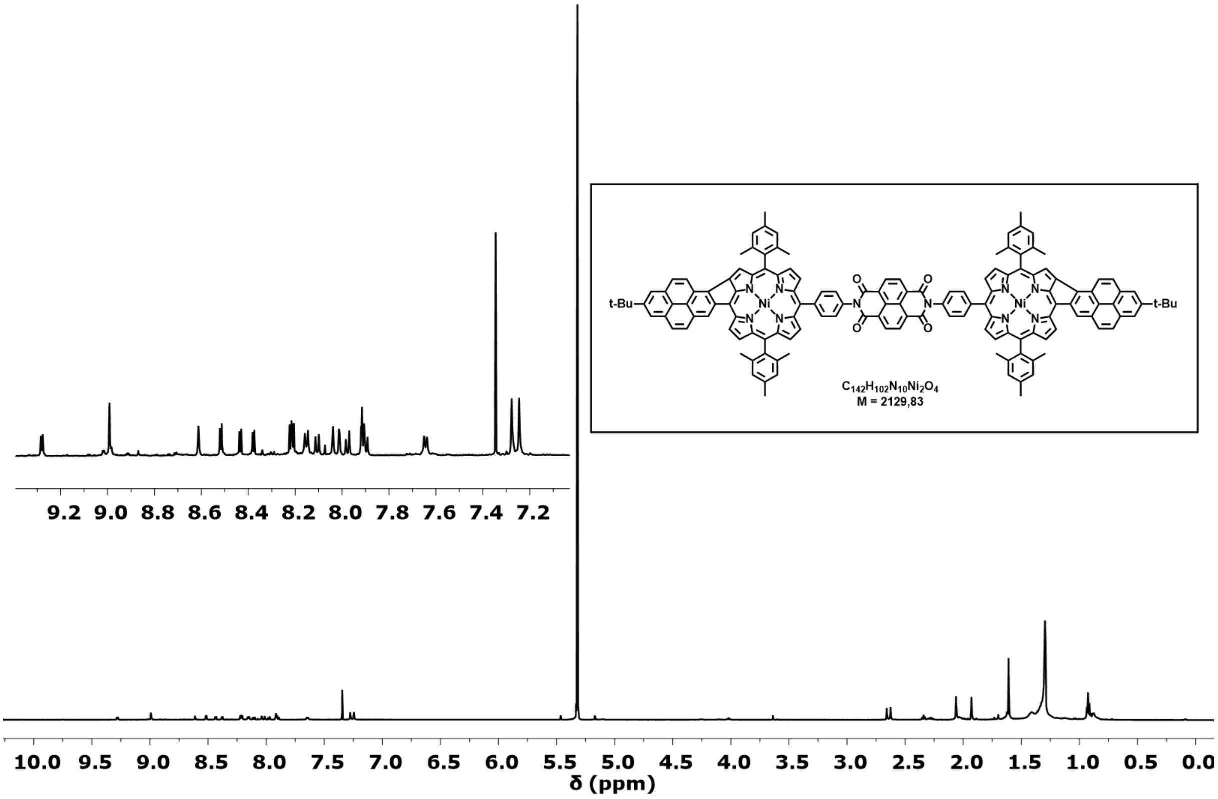

^{13}C NMR - DEPTQ135 (151 MHz, CD_2Cl_2, rt)

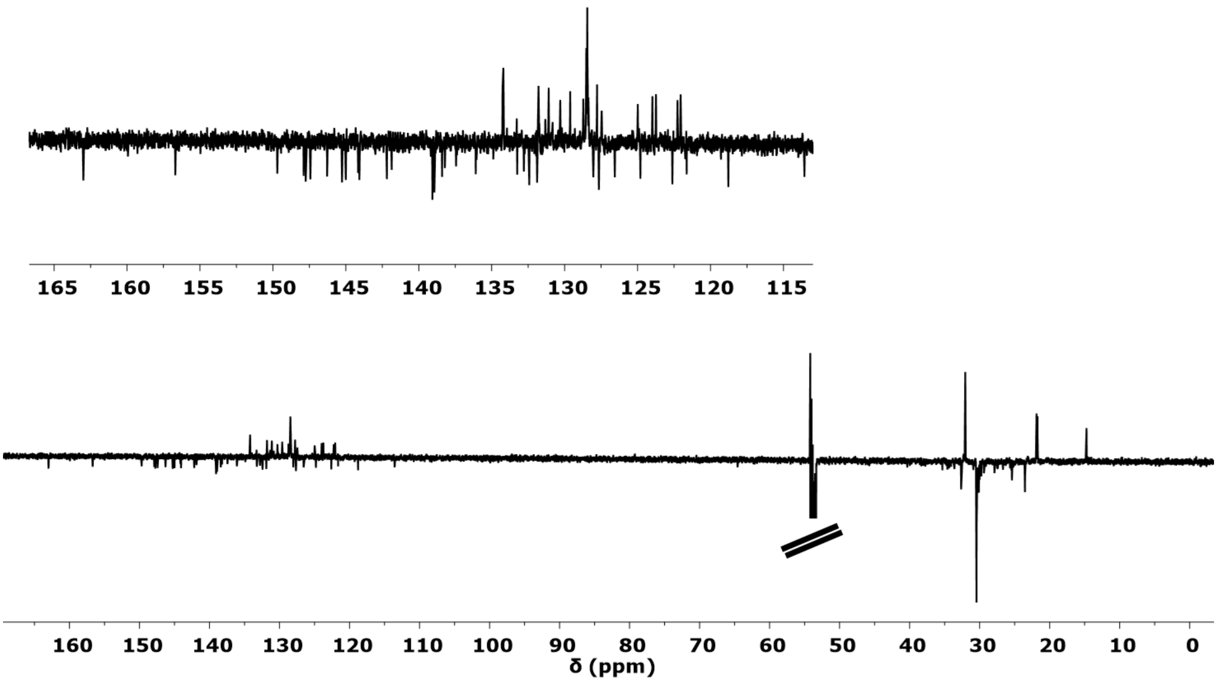

Figure S87. ^{1}H and ^{13}C NMR (DEPTQ135) of **Pyr-NDI-Pyr**.

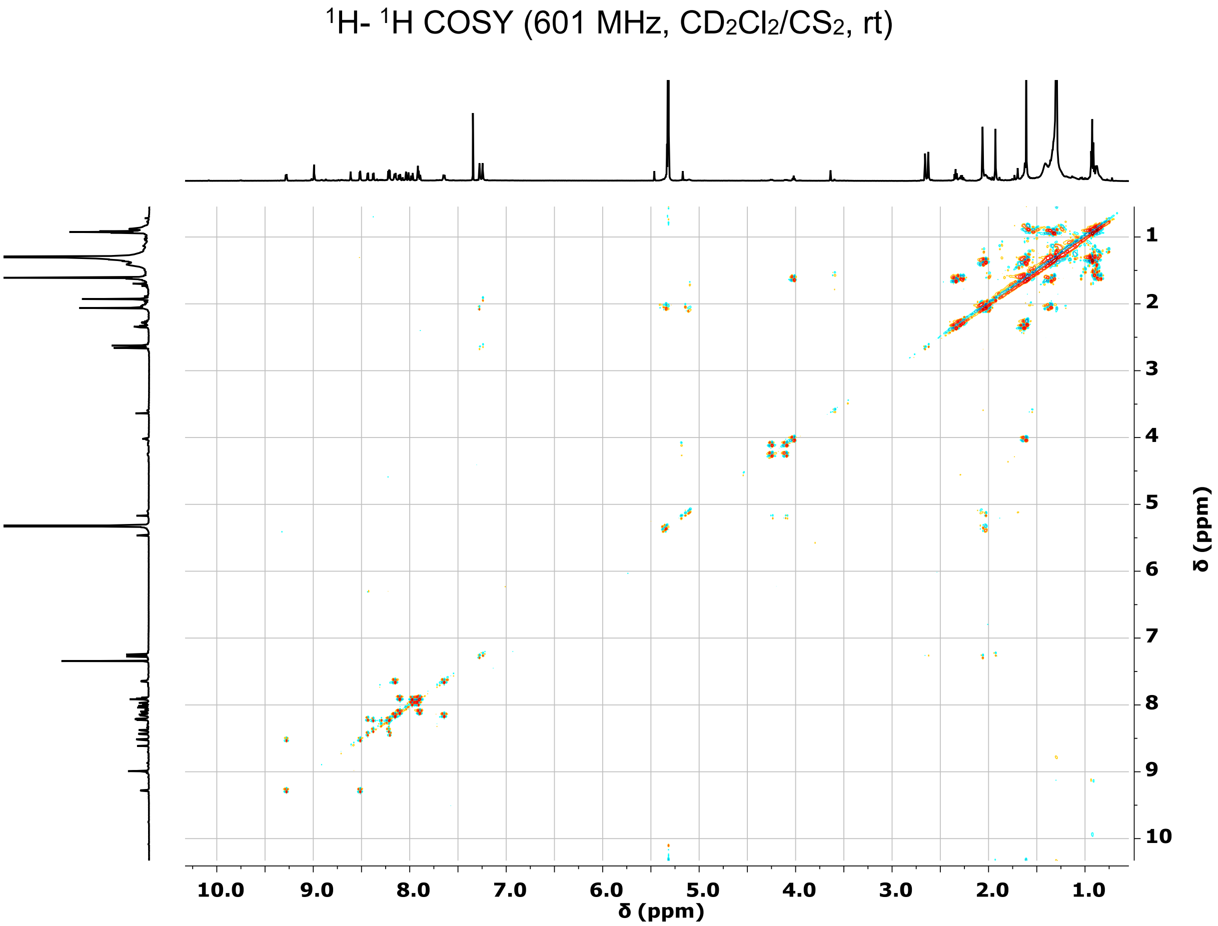

Figure S88. ^{1}H- ^{1}H COSY of **Pyr-NDI-Pyr**.

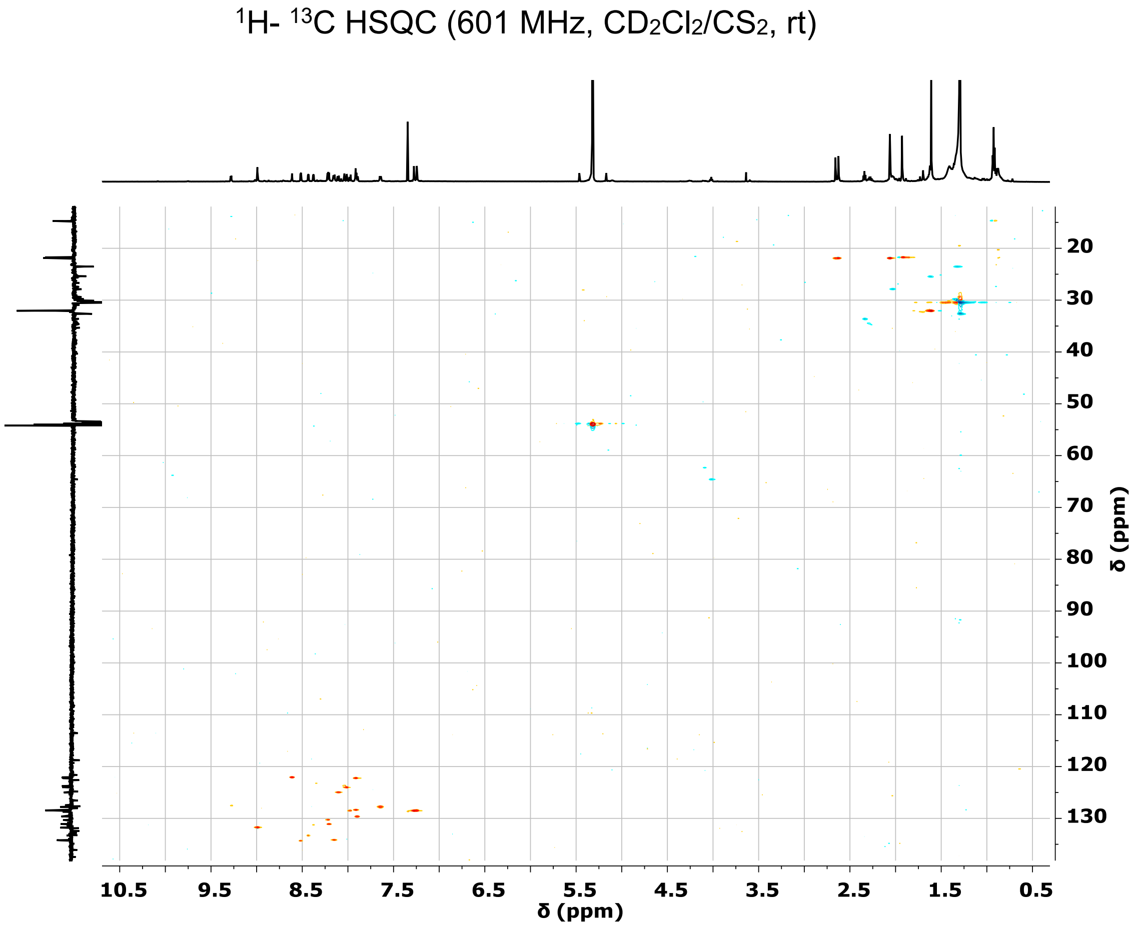

Figure S89. ^{1}H- ^{13}C HSQC of **Pyr-NDI-Pyr**.

^1H- ^{13}C HMBC (601 MHz, CD_2Cl_2/CS_2, rt)

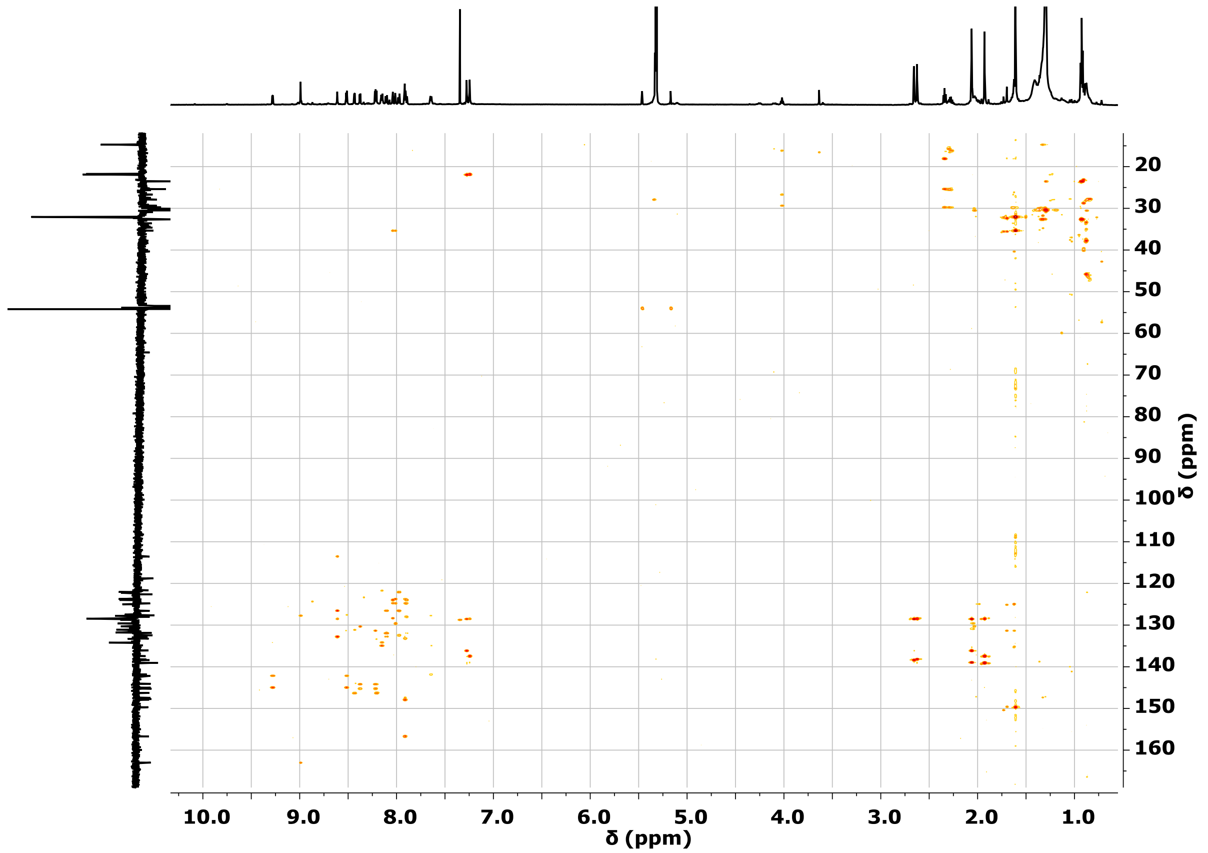

Figure S90. ^1H- ^{13}C HMBC of **Pyr-NDI-Pyr**.

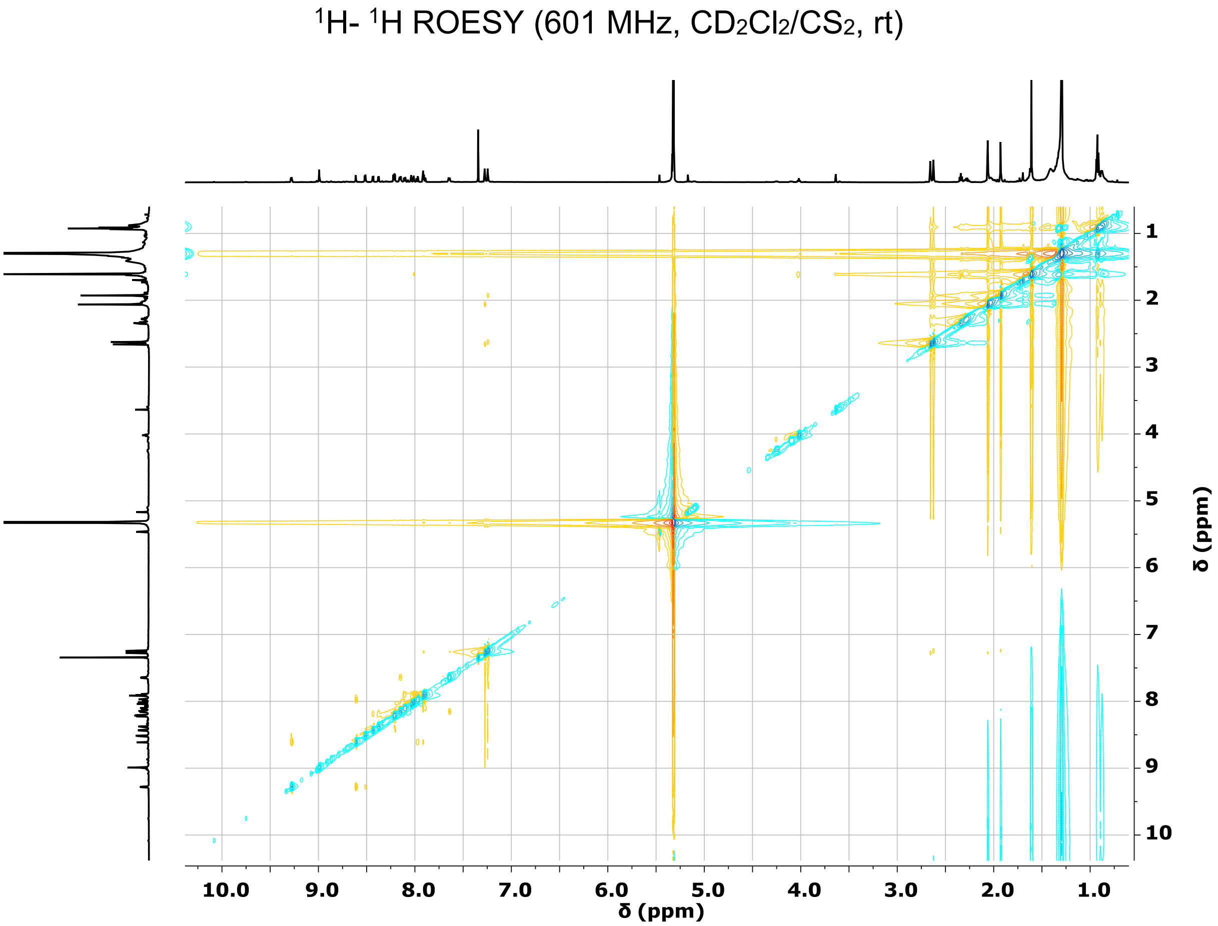

Figure S91. ^{1}H- ^{1}H ROESY of **Pyr-NDI-Pyr**.

MS (MALDI)

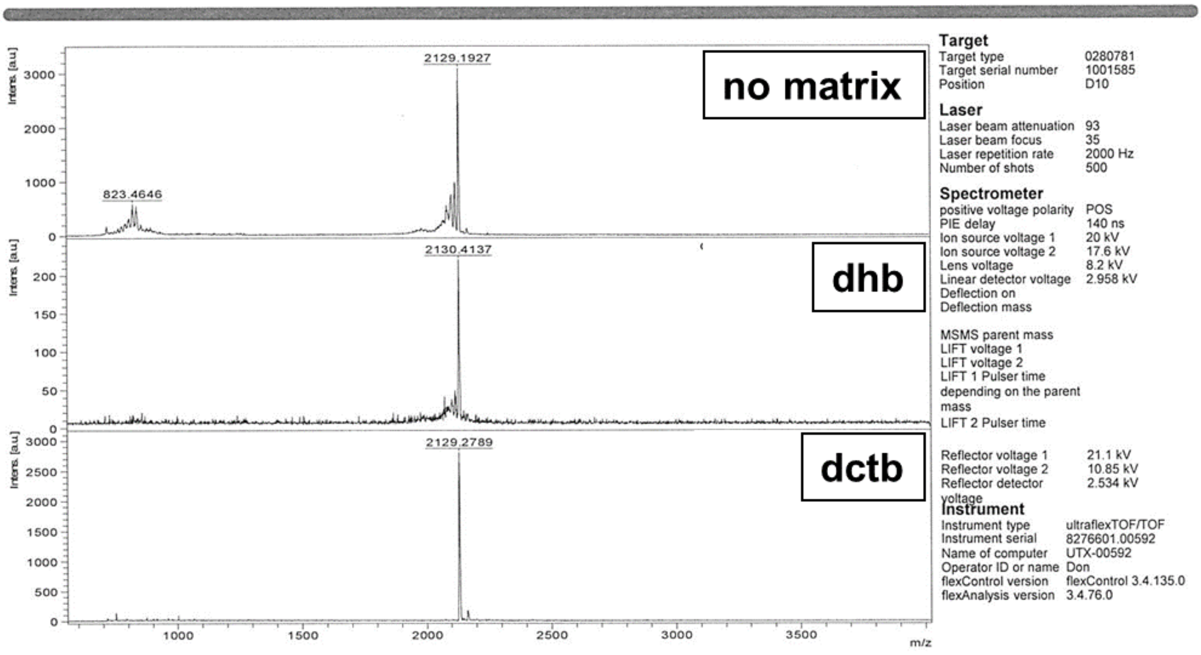

HRMS (MALDI)

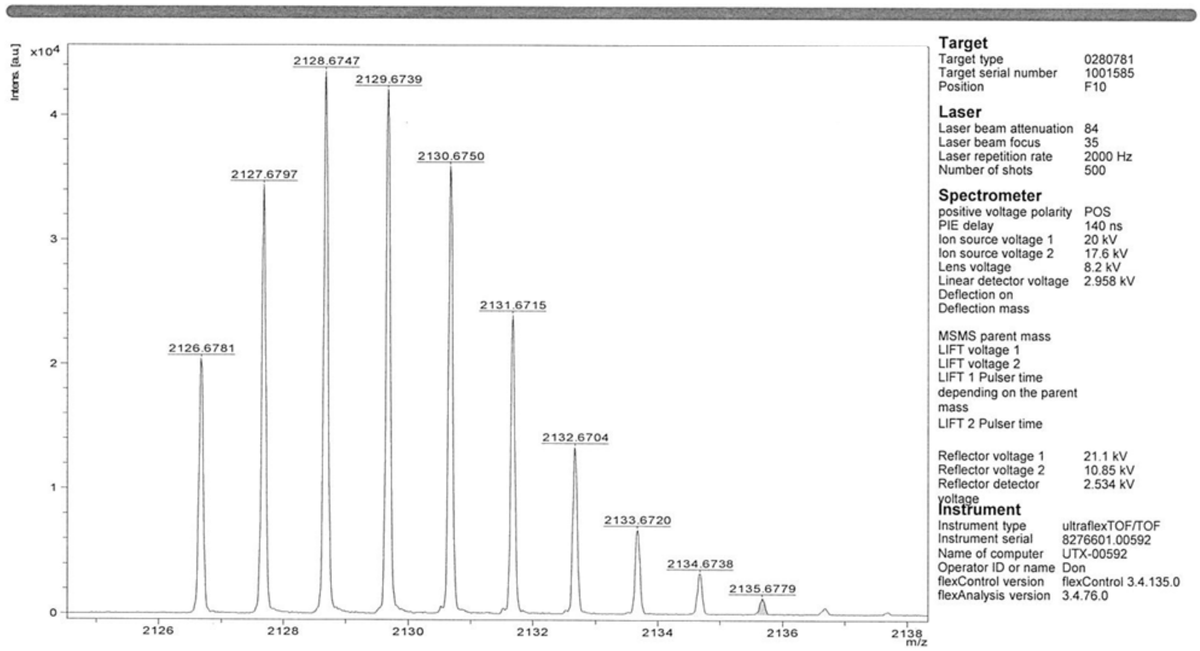

SmartFormula

Formula	Mass	Error	mSigma	DblEq	N rule	Electron Configuration
C 142 H 102 N 10 Ni 2 O 4	2,126.6787	0.2795	26.3358	97.00	ok	odd

Figure S92. MS/HRMS (MALDI) of **Pyr-NDI-Pyr**.

^{1}H NMR (601 MHz, CD_2Cl_2/CS_2, rt)

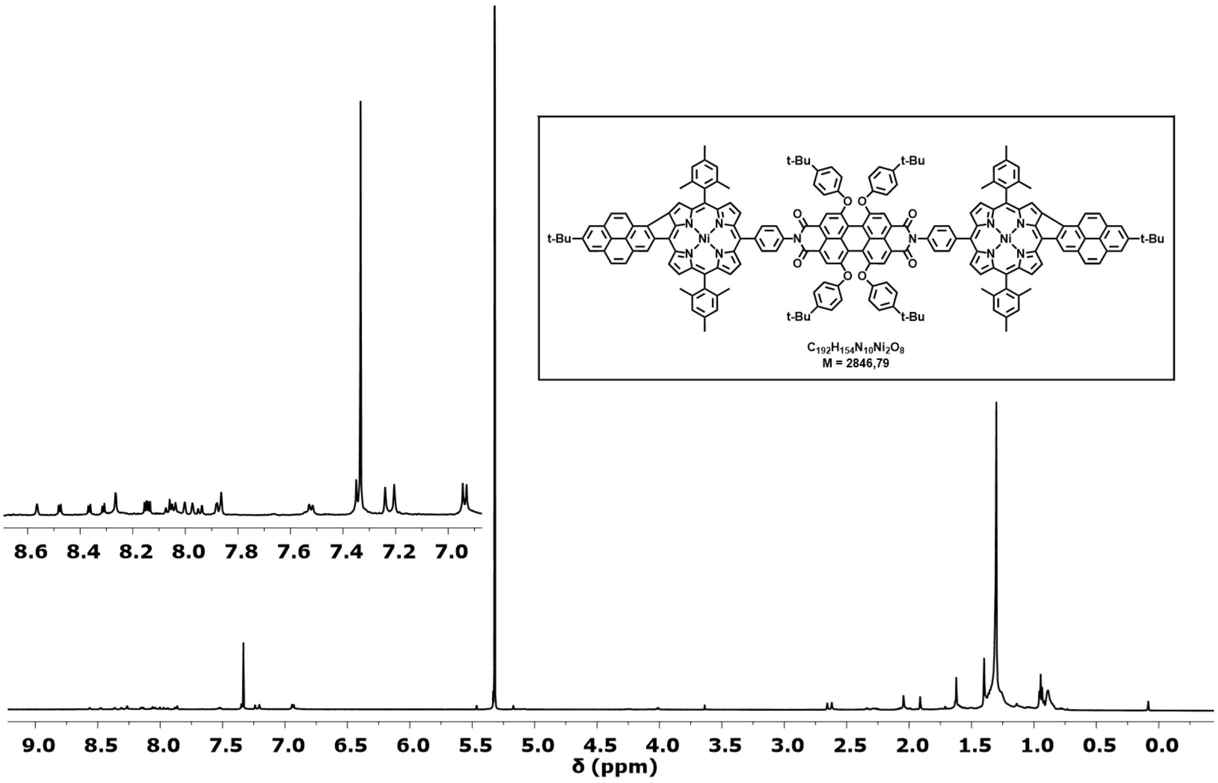

^{13}C NMR - DEPTQ135 (151 MHz, CD_2Cl_2, rt)

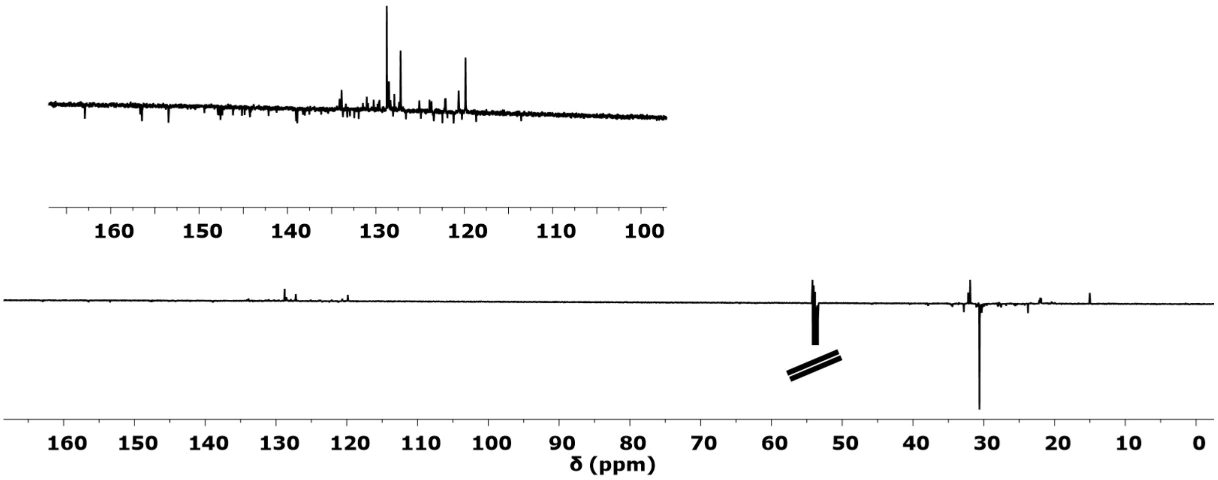

Figure S93. ^{1}H and ^{13}C NMR (DEPTQ135) of **Pyr-PDI-Pyr**.

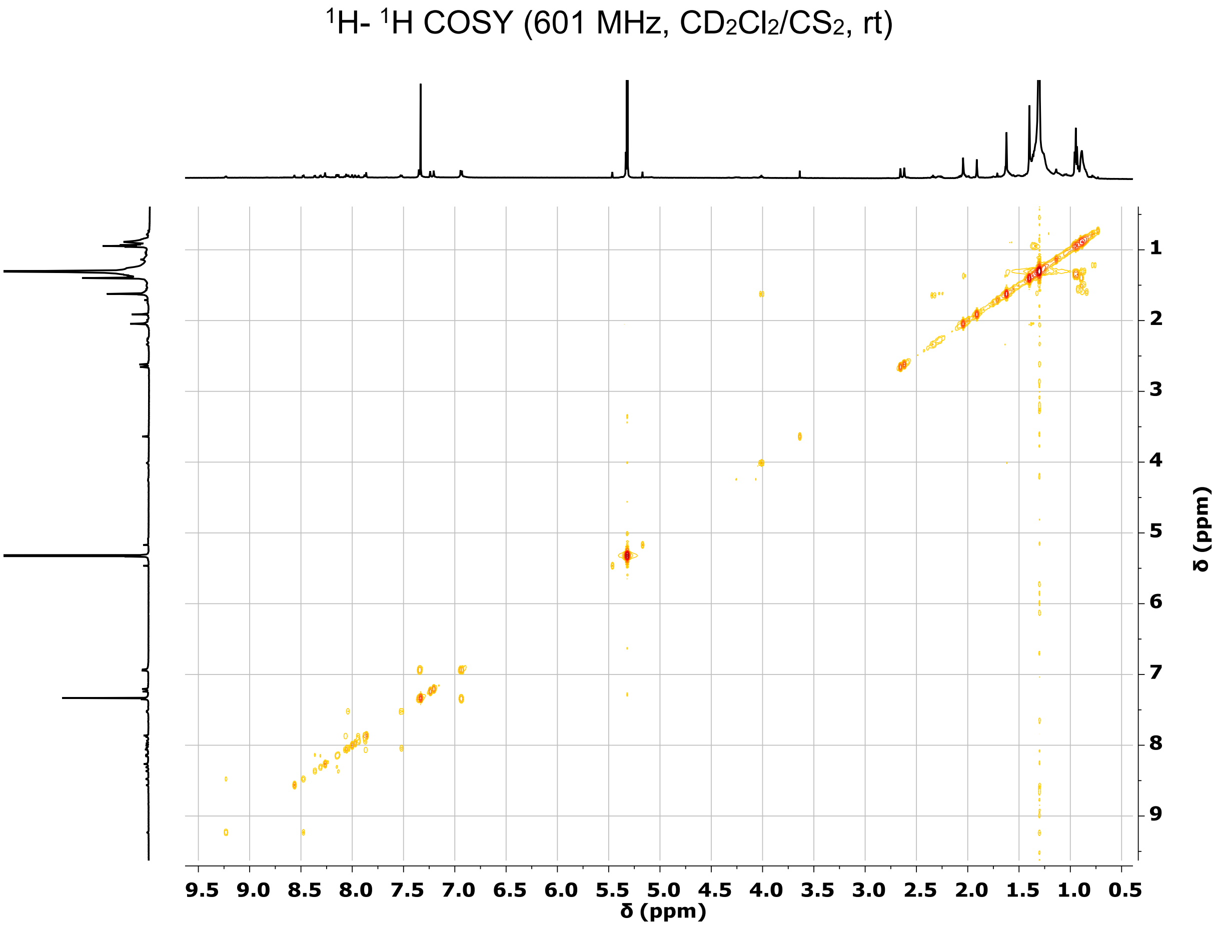

Figure S94. ^{1}H- ^{1}H COSY of **Pyr-PDI-Pyr**.

^{1}H- ^{13}C HSQC (601 MHz, CD_2Cl_2/CS_2, rt)

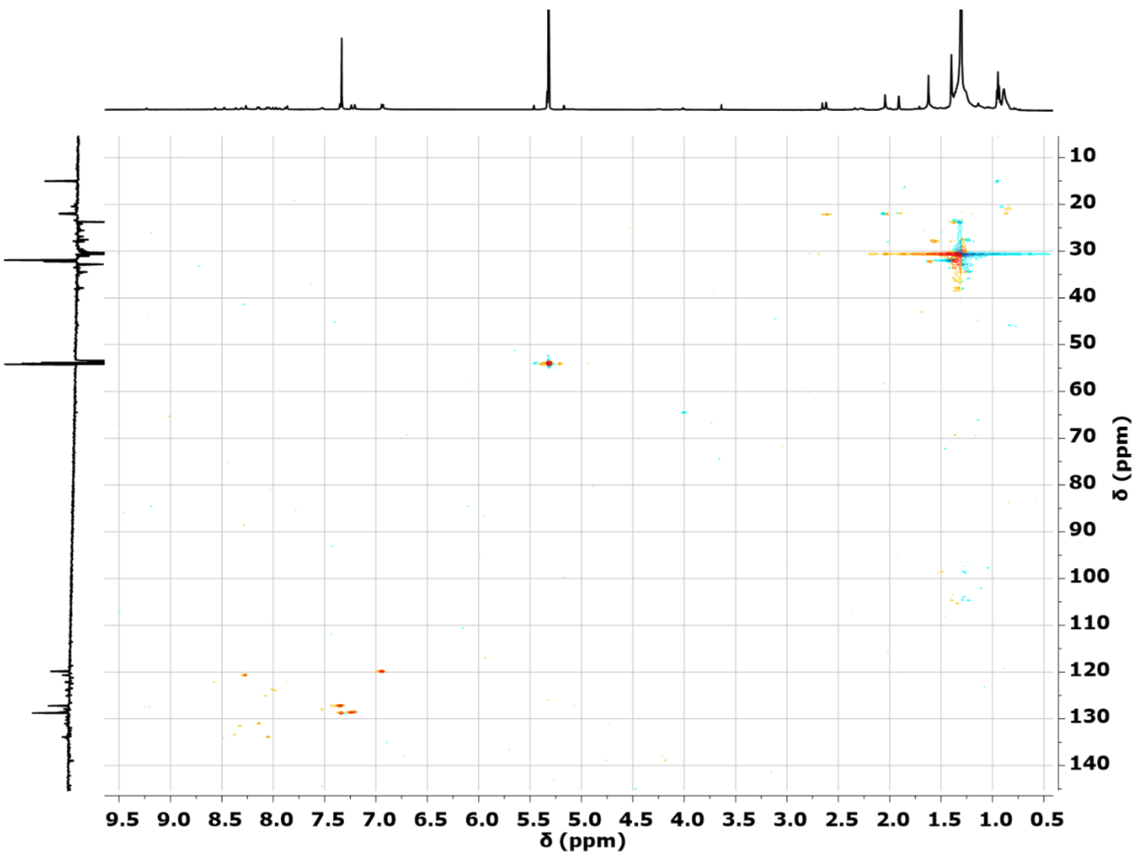

Figure S95. ^{1}H- ^{13}C HSQC of **Pyr-PDI-Pyr**.

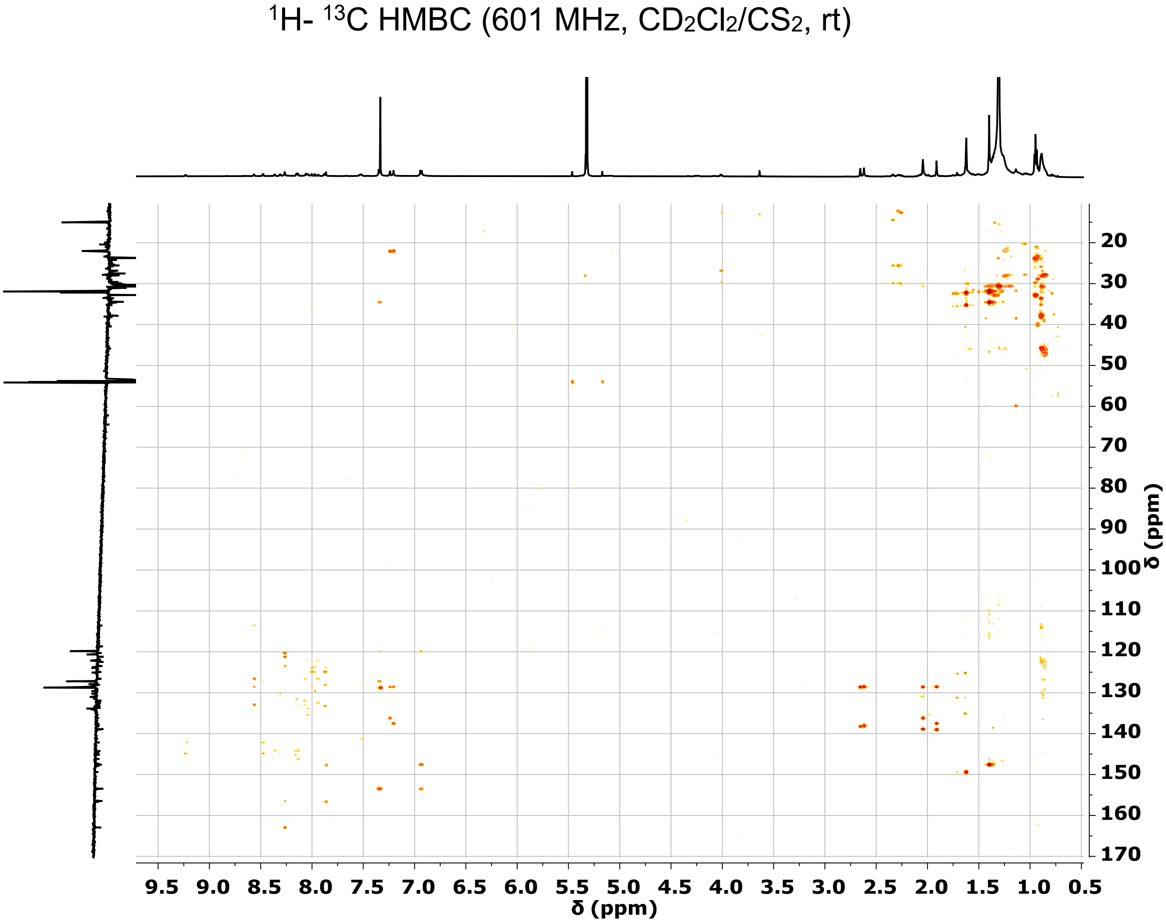

Figure S96. ^{1}H- ^{13}C HMBC of **Pyr-PDI-Pyr**.

MS (MALDI)

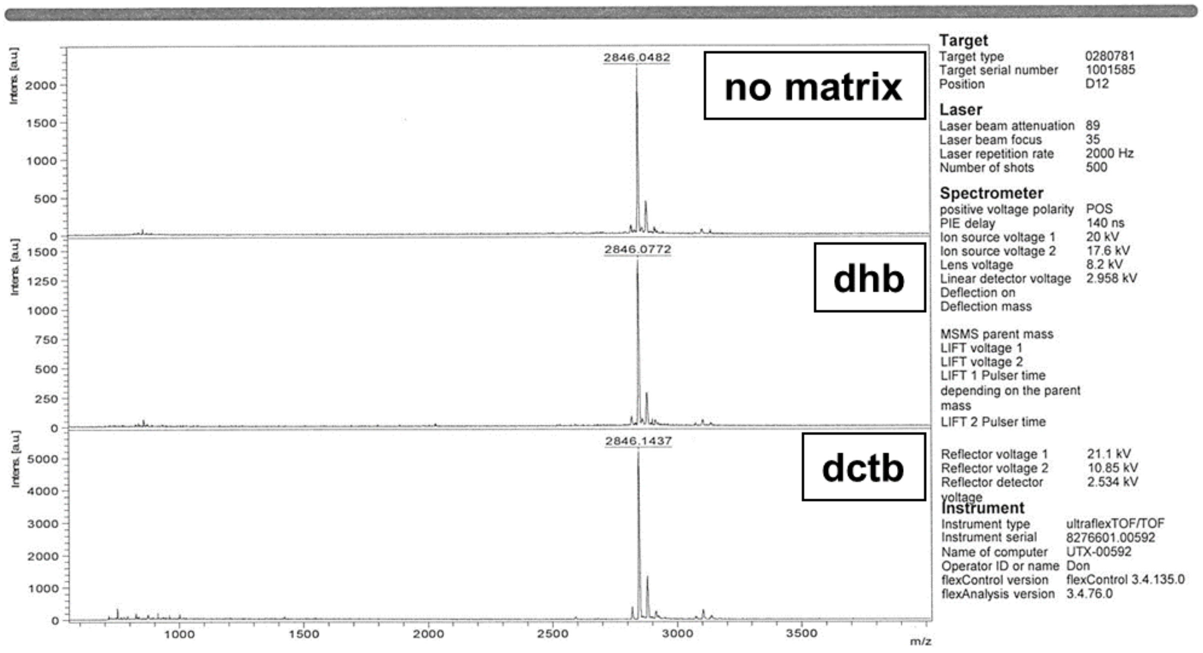

HRMS (MALDI)

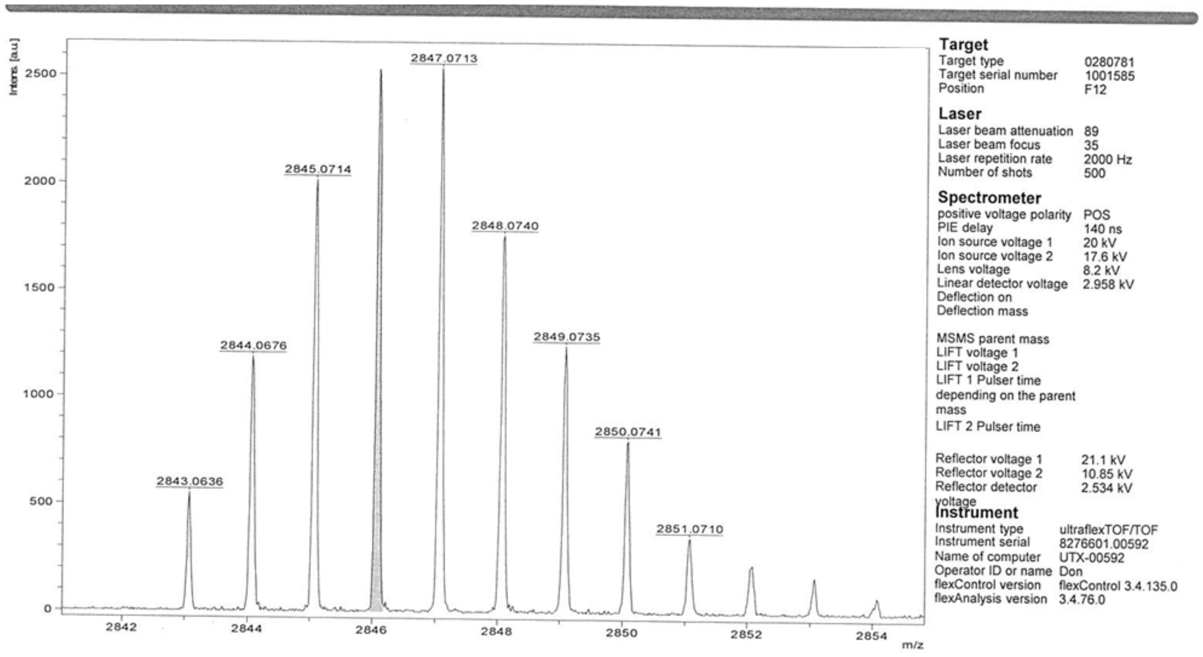

SmartFormula

Formula	Mass	Error	mSigma	DblEq	N rule	Electron Configuration
C 192 H 154 N 10 Ni 2 O 8	2,843.0652	0.5757	74.7957	121.00	ok	odd

Figure S97. MS/HRMS (MALDI) of **Pyr-PDI-Pyr**.

^1H NMR (601 MHz, CD_2Cl_2/CS_2, rt)

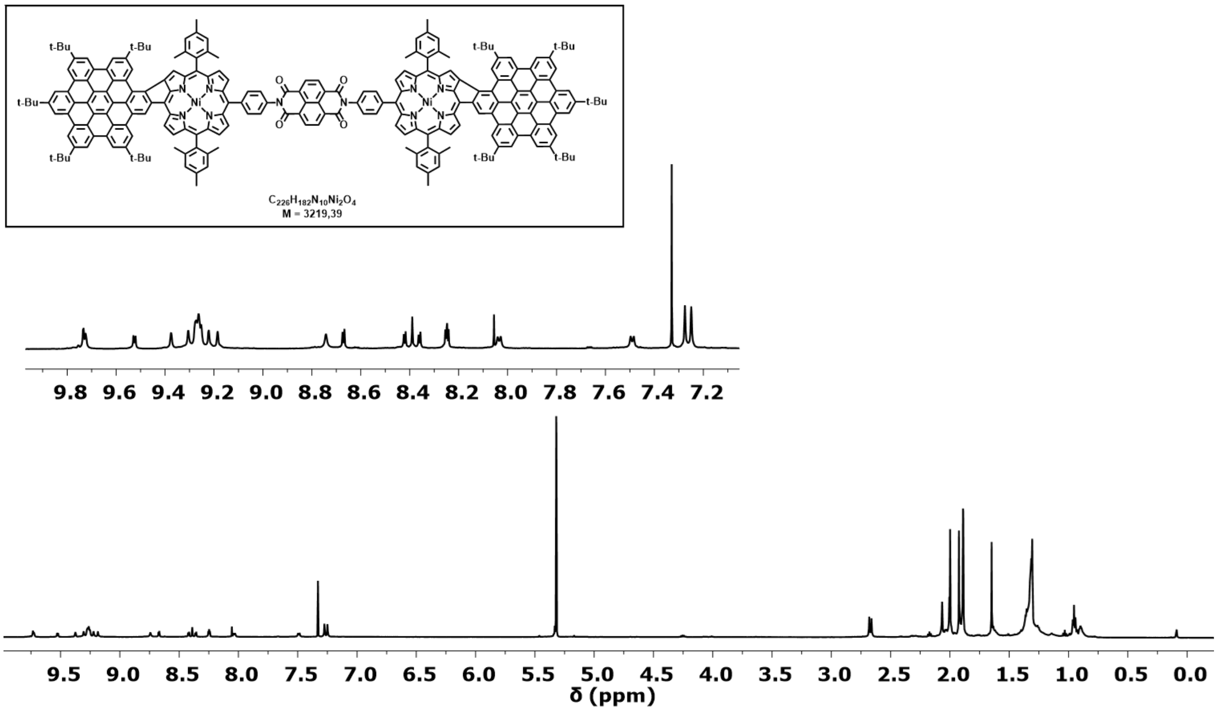

^{13}C NMR - DEPTQ135 (151 MHz, CD_2Cl_2/CS_2, rt)

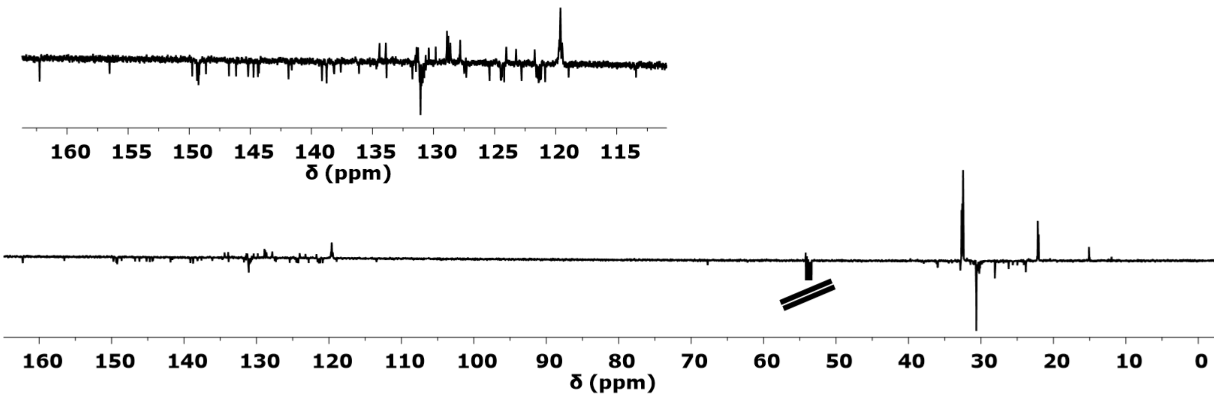

Figure S98. ^1H and ^{13}C NMR (DEPTQ135) of **HBC-NDI-HBC**.

^1H- ^1H COSY (601 MHz, CD_2Cl_2/CS_2, rt)

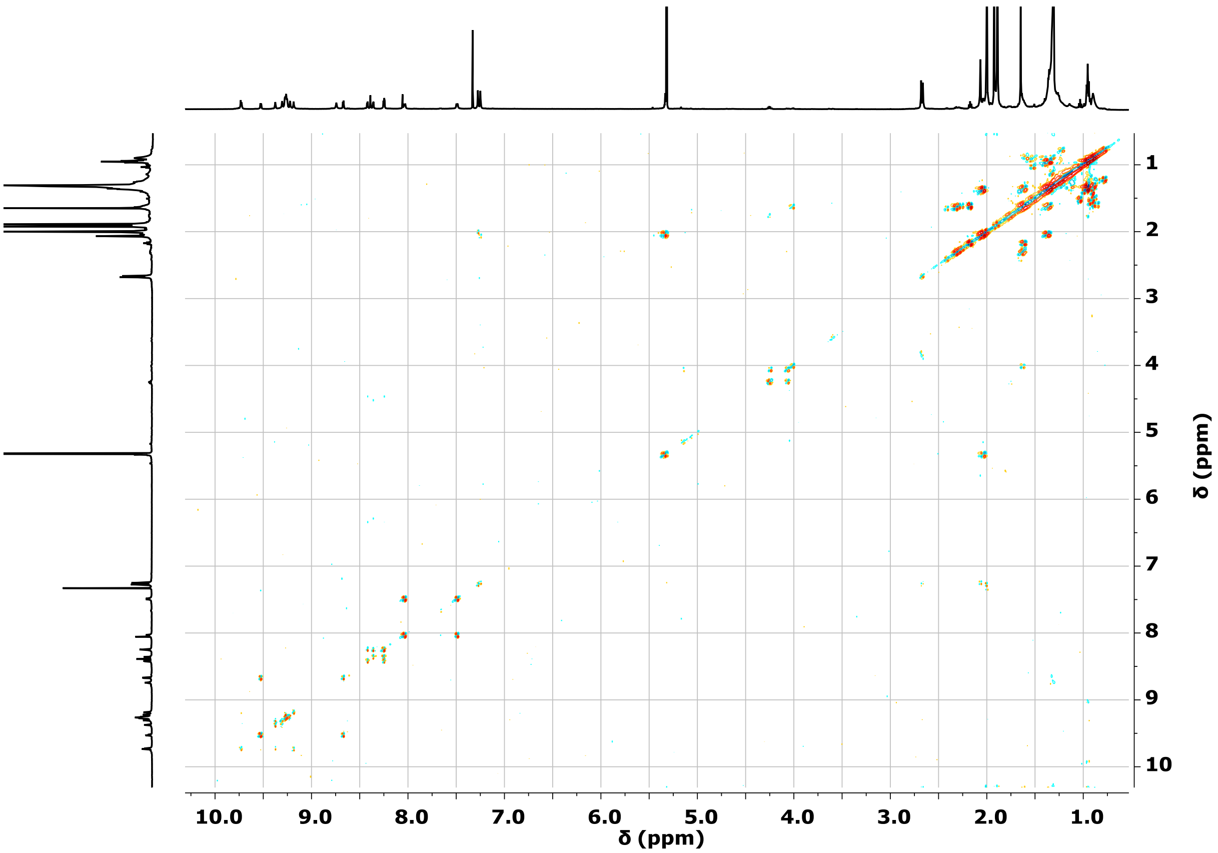

Figure S99. ^1H- ^1H COSY of **HBC-NDI-HBC**.

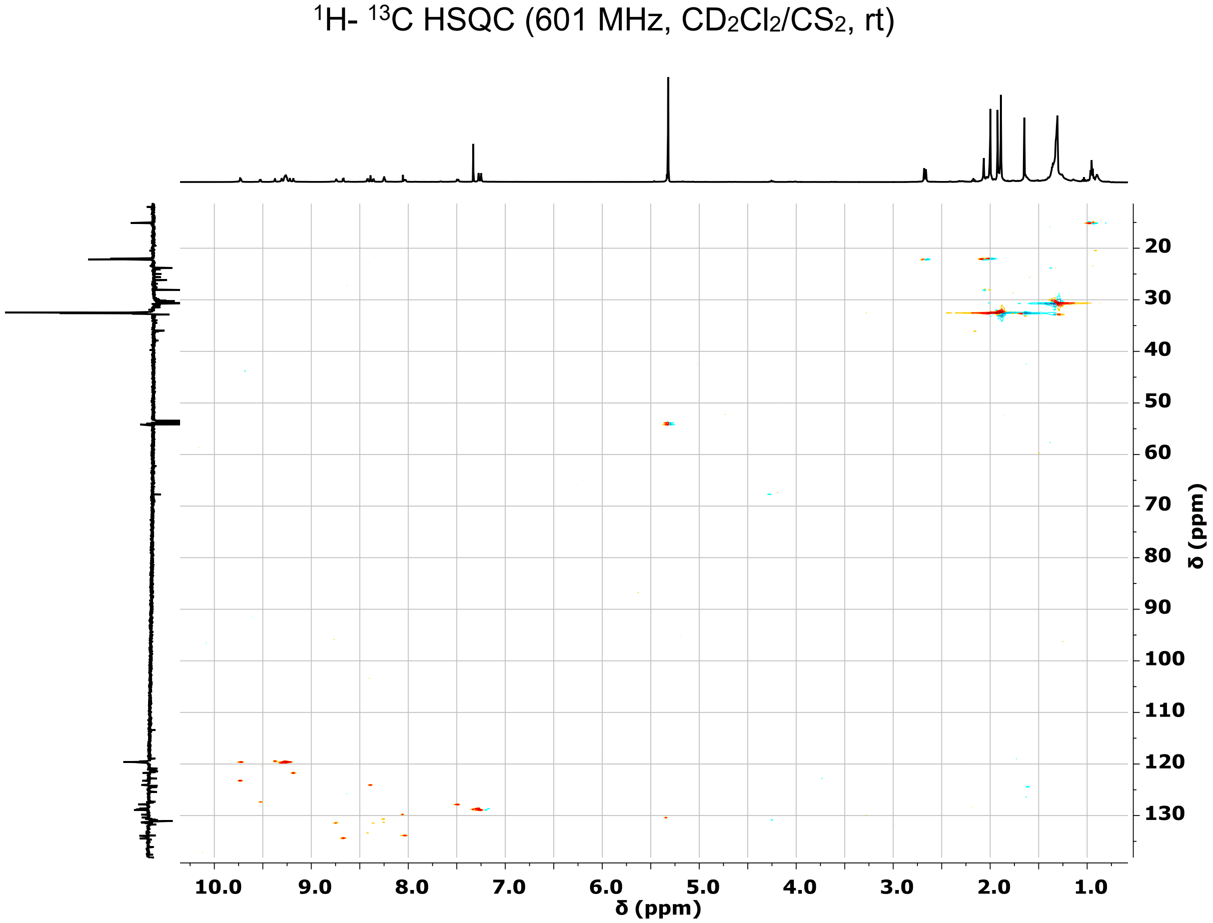

Figure S100. ^{1}H- ^{13}C HSQC of **HBC-NDI-HBC**.

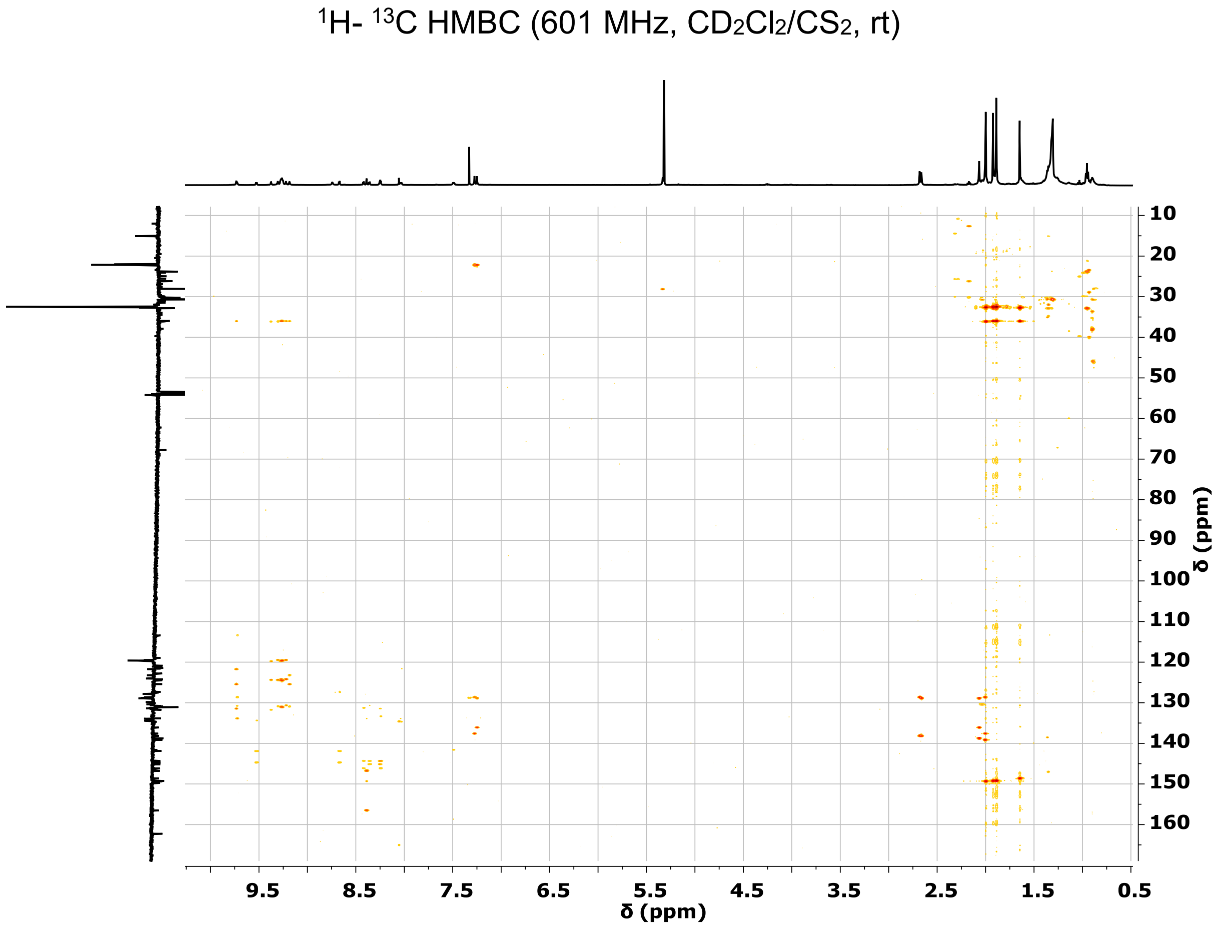

Figure S101. ^{1}H- ^{13}C HMBC of **HBC-NDI-HBC**.

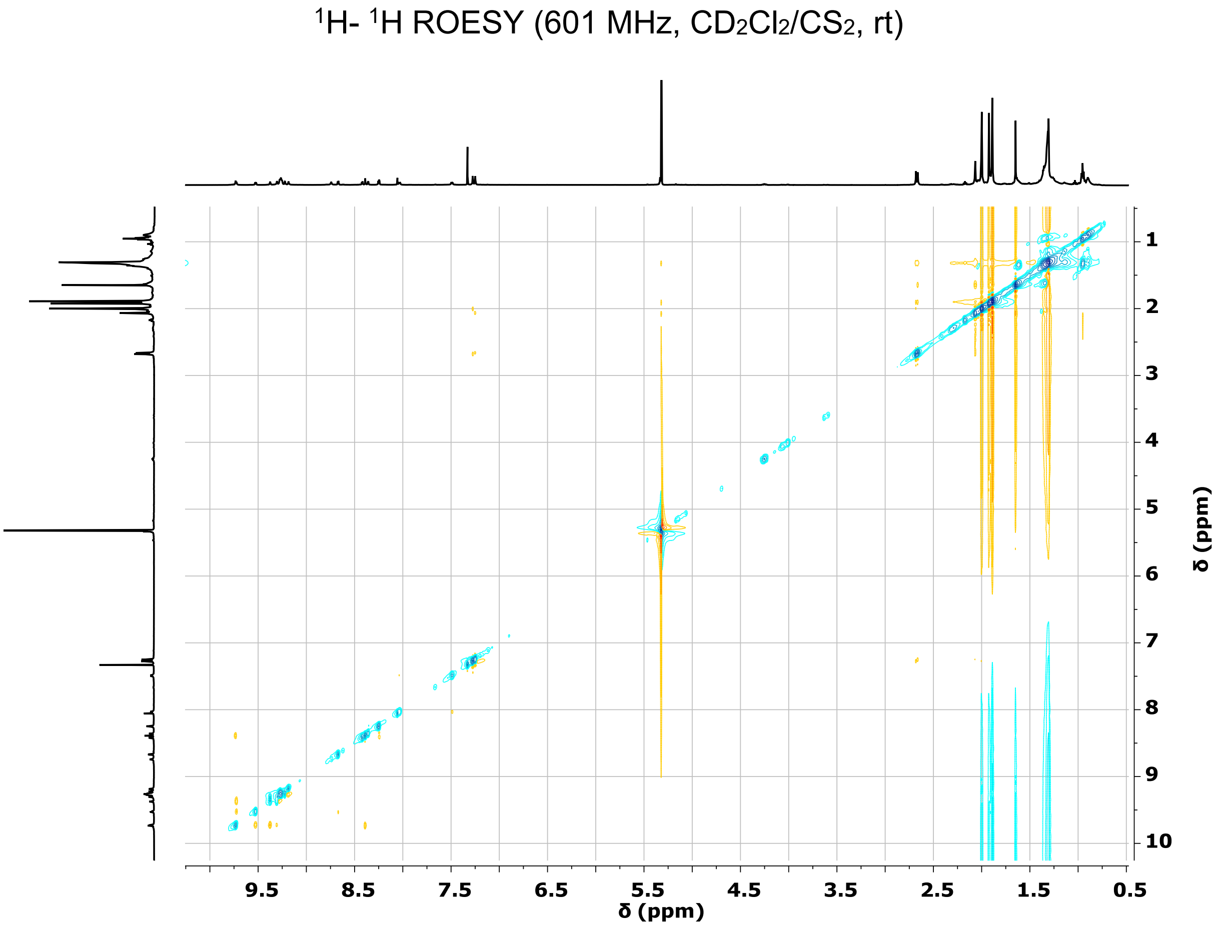

Figure S102. ^{1}H- ^{1}H ROESY of **HBC-NDI-HBC**.

MS (MALDI)

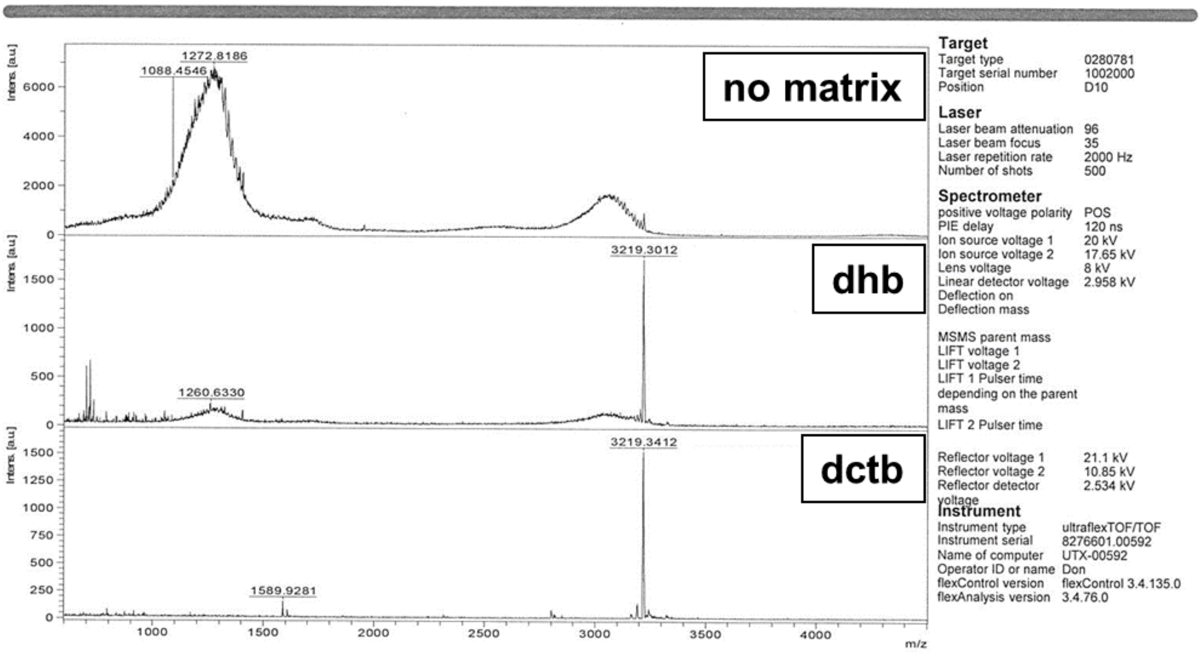

HRMS (MALDI)

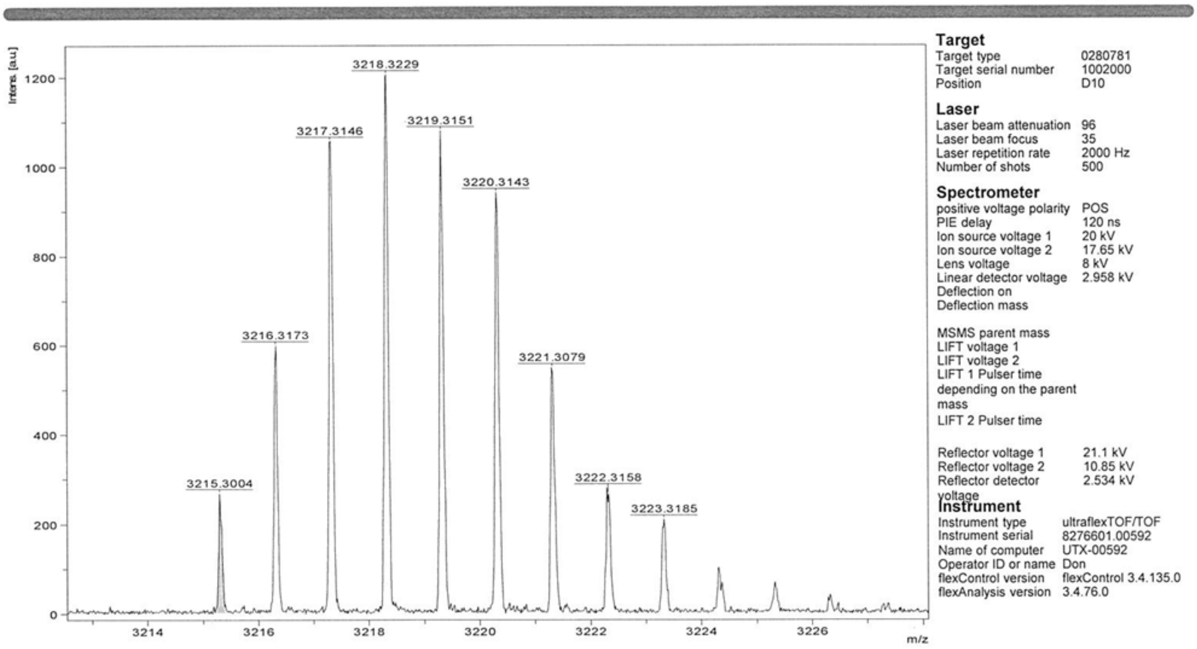

SmartFormula

Formula	Mass	Error	mSigma	DblEq	N rule	Electron Configuration
C 226 H 182 N 10 Ni 2 O 4	3,215.3047	1.3372	66.1907	141.00	ok	odd

Figure S103. MS/HRMS (MALDI) of **HBC-NDI-HBC**.

^{1}H NMR (601 MHz, CD_2Cl_2/CS_2, rt)

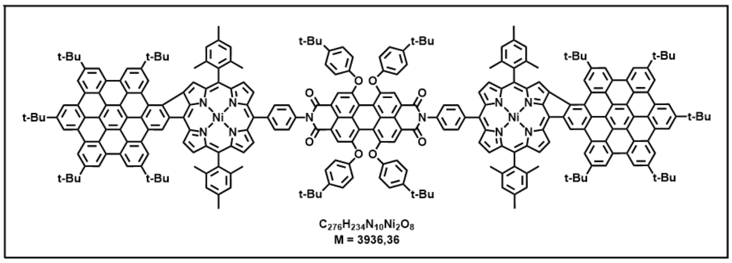

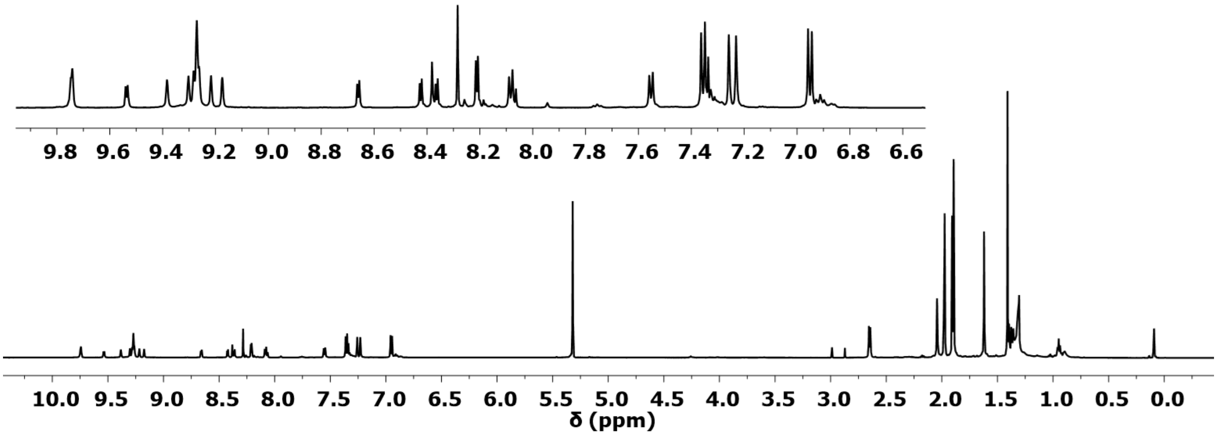

^{13}C NMR - DEPTQ135 (151 MHz, CD_2Cl_2/CS_2, rt)

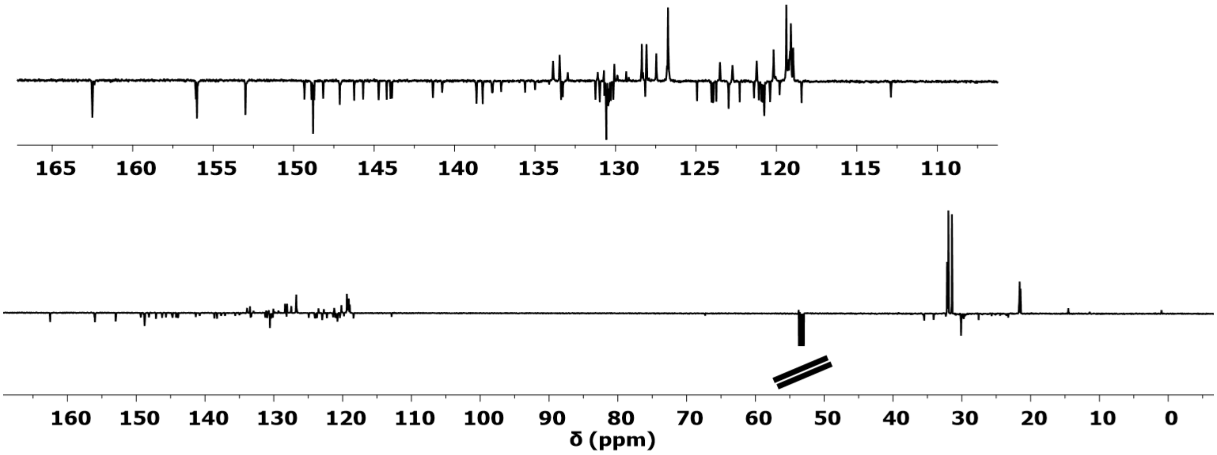

Figure S104. ^{1}H and ^{13}C NMR (DEPTQ135) of **HBC-PDI-HBC**.

^1H- ^1H COSY (601 MHz, CD_2Cl_2/CS_2, rt)

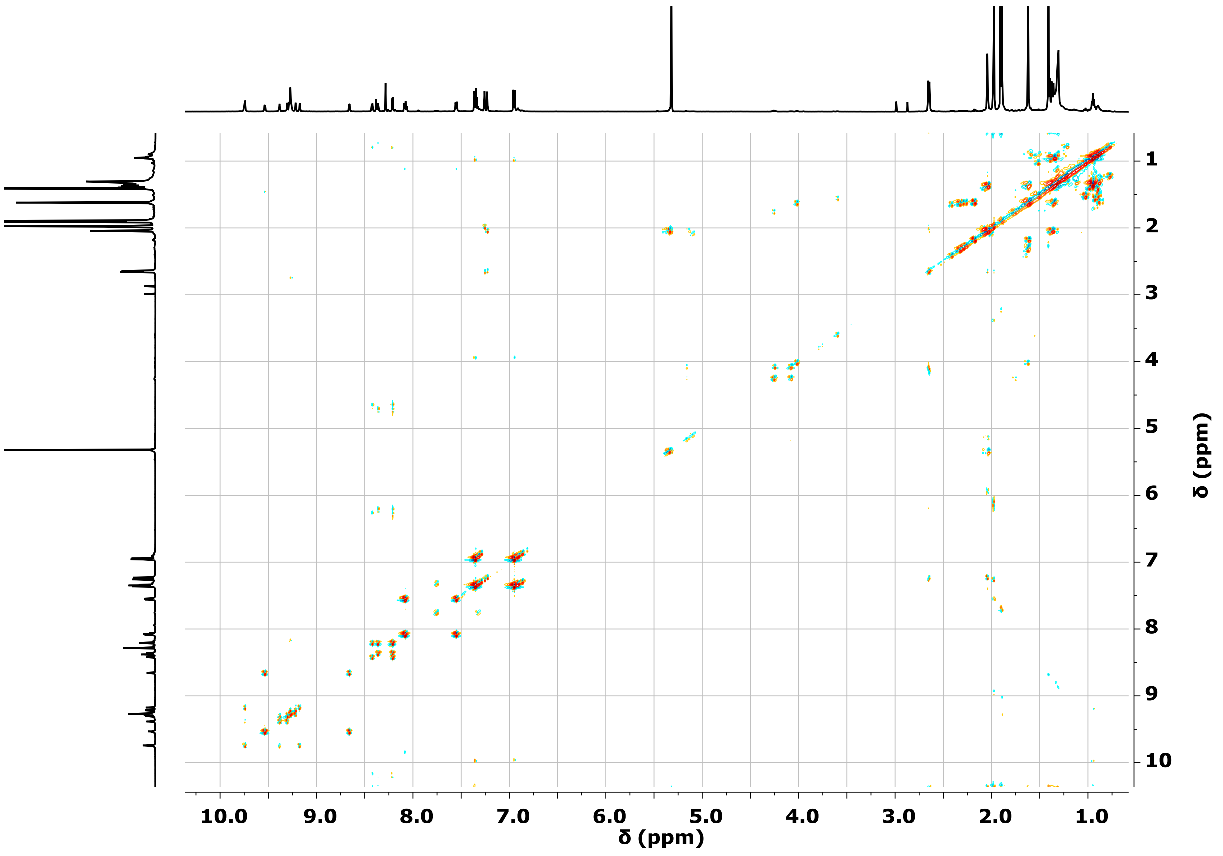

Figure S105. ^1H- ^1H COSY of **HBC-PDI-HBC**.

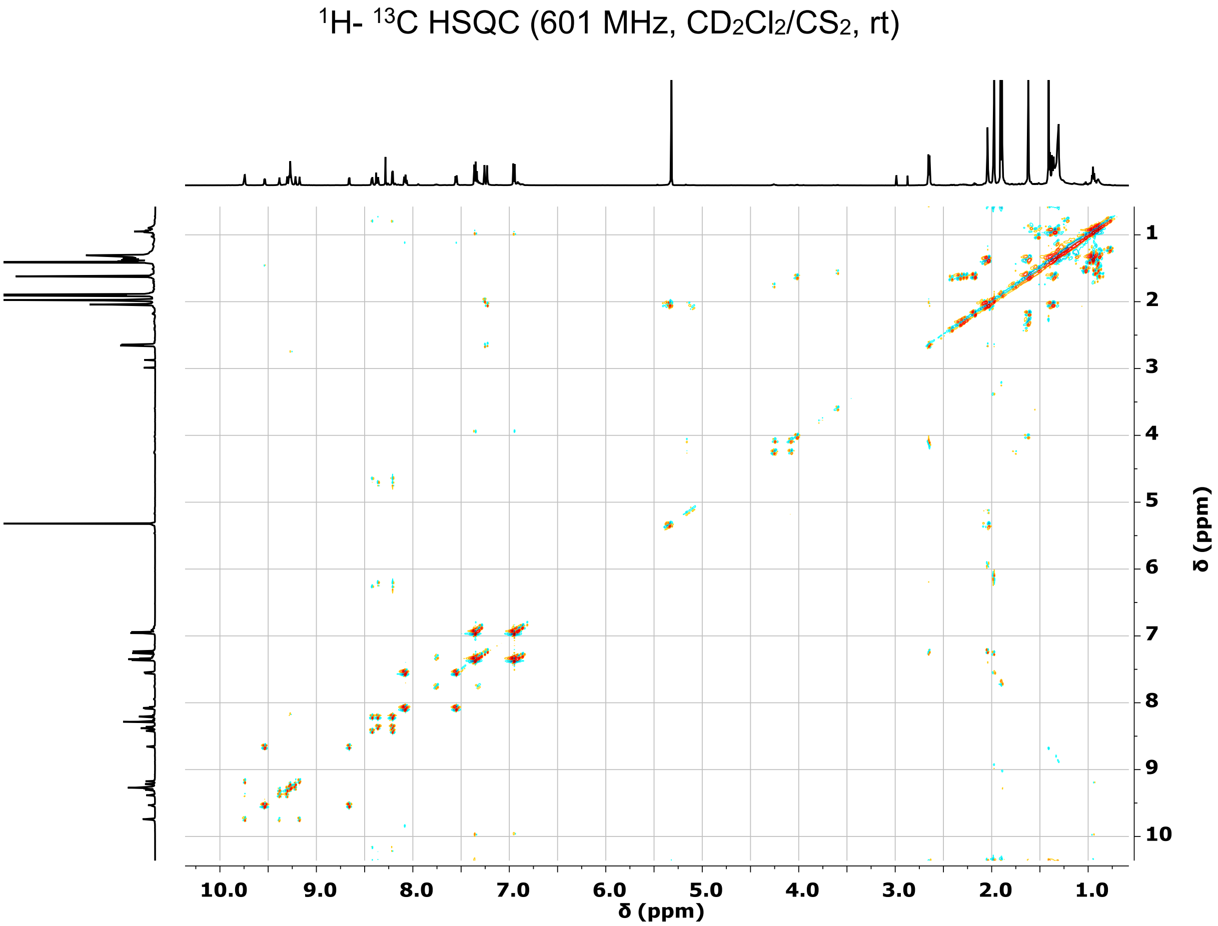

Figure S106. ^{1}H- ^{13}C HSQC of **HBC-PDI-HBC**.

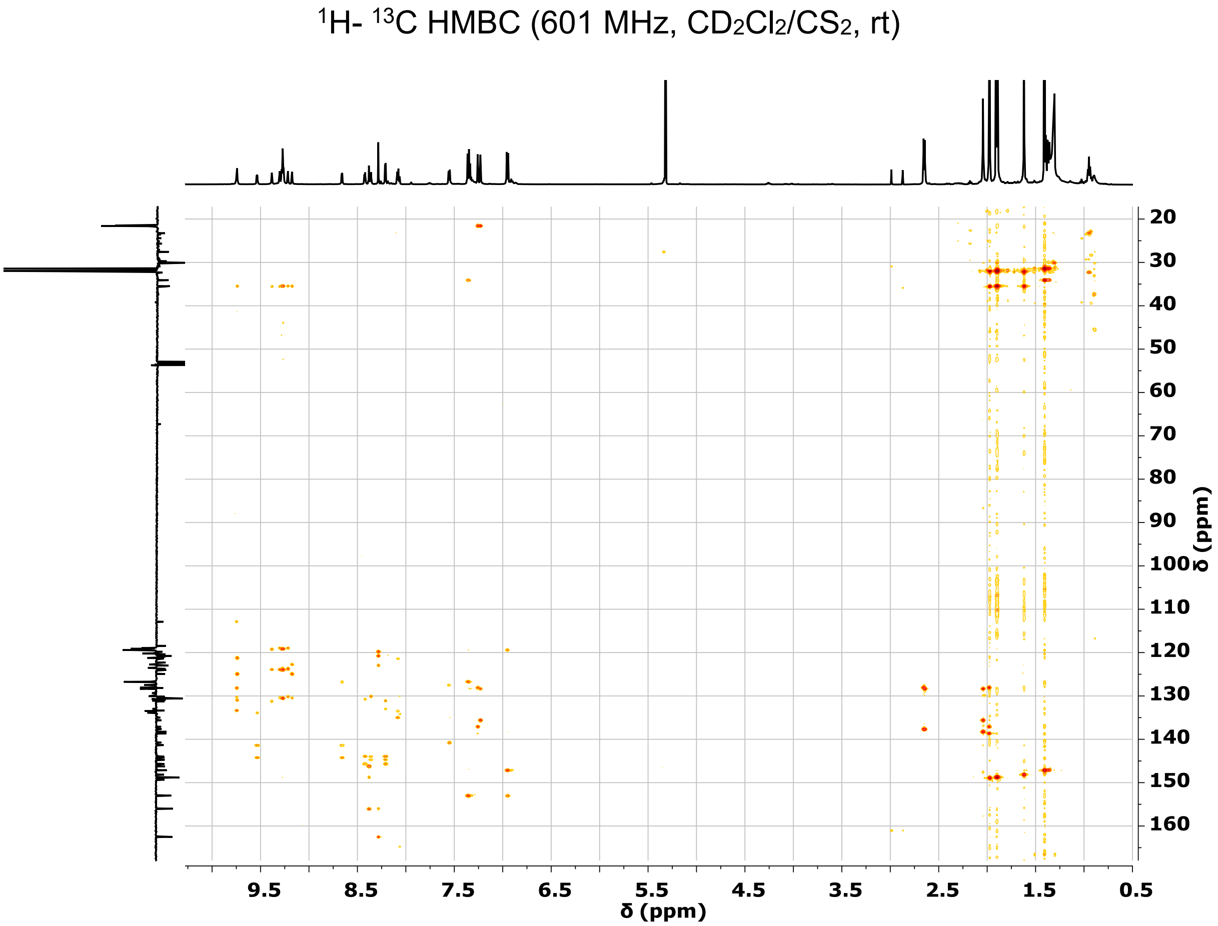

Figure S108. ^{1}H- ^{13}C HMBC of **HBC-PDI-HBC**.

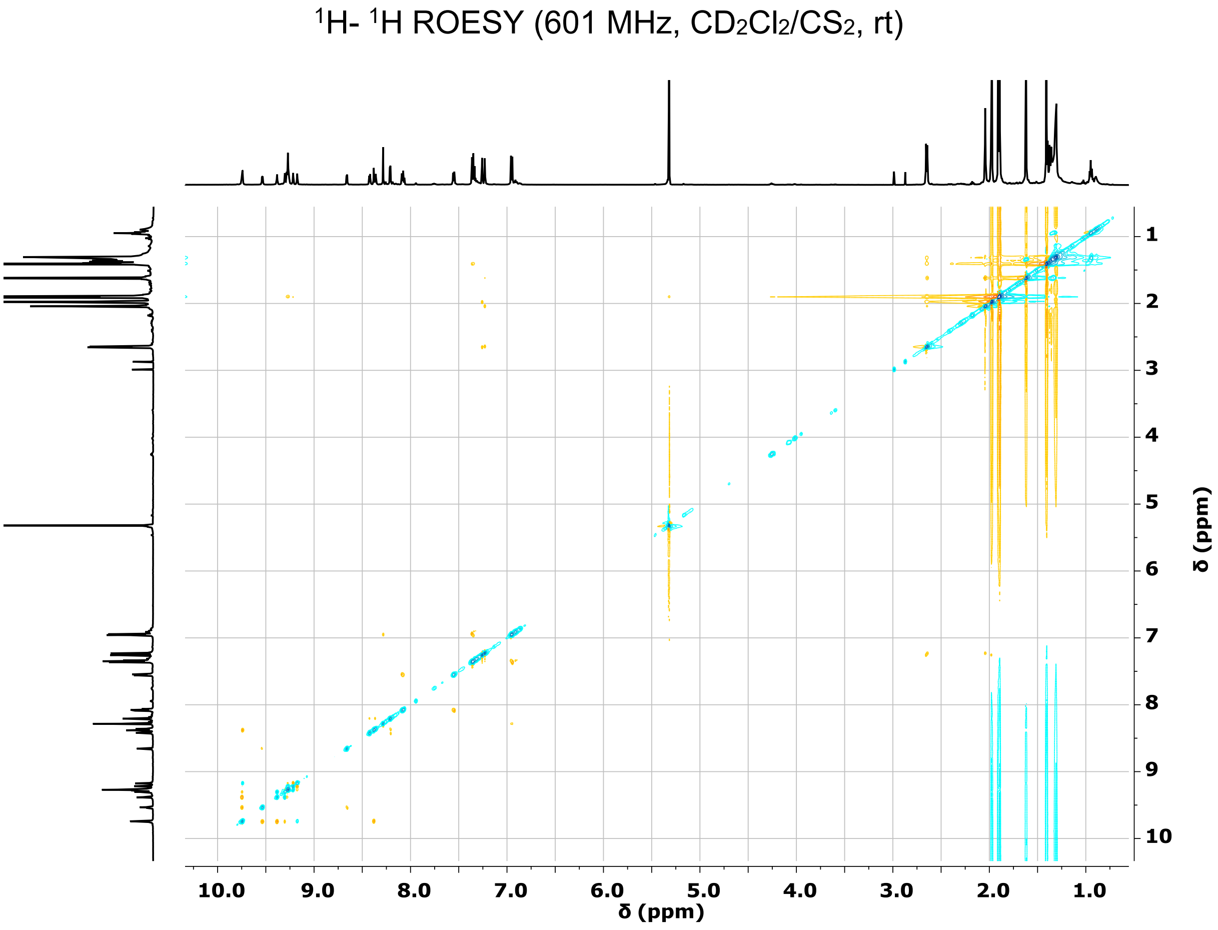

Figure S109. ^{1}H- ^{1}H ROESY of **HBC-PDI-HBC**.

MS (MALDI)

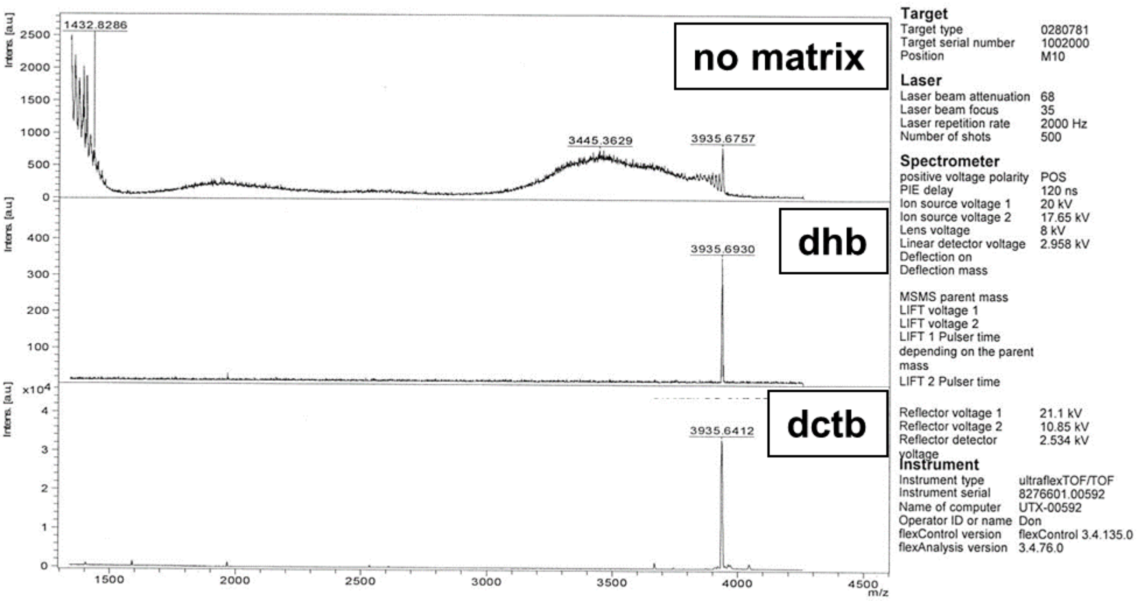

HRMS (MALDI)

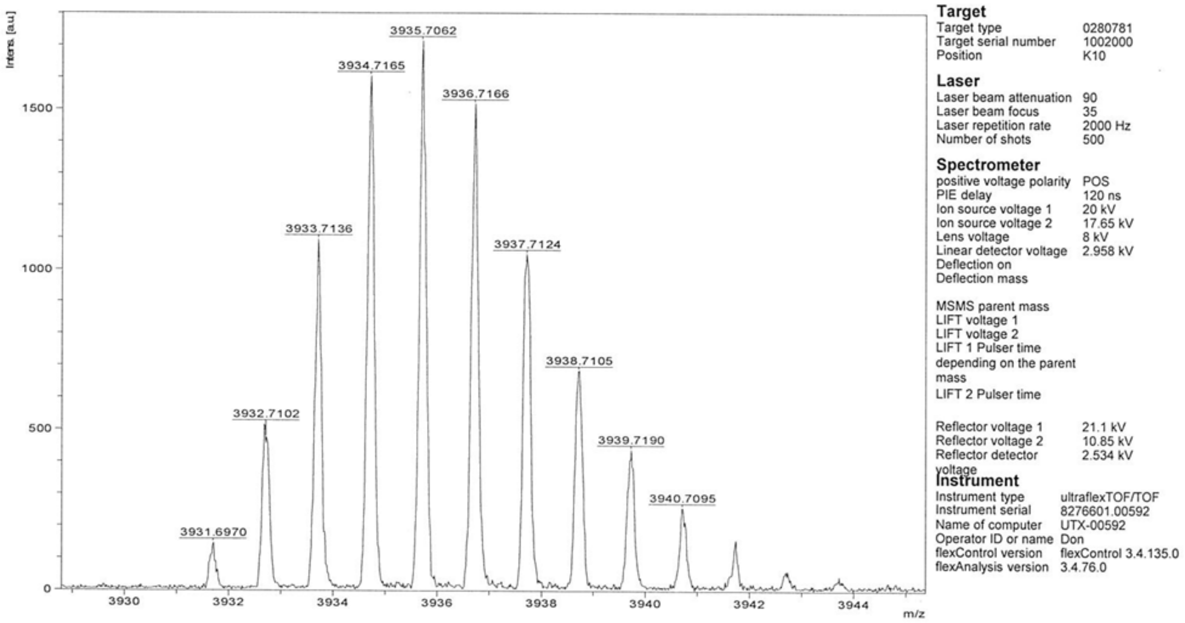

SmartFormula

Formula	Mass	Error	mSigma	DblEq	N rule	Electron Configuration
C 276 H 234 N 10 Ni 2 O 8	3,931.6913	1.4695	66.4858	165.00	ok	odd

Figure S110. MS/HRMS (MALDI) of **HBC-PDI-HBC**.

UV/Vis Absorptions of the Key Precursors

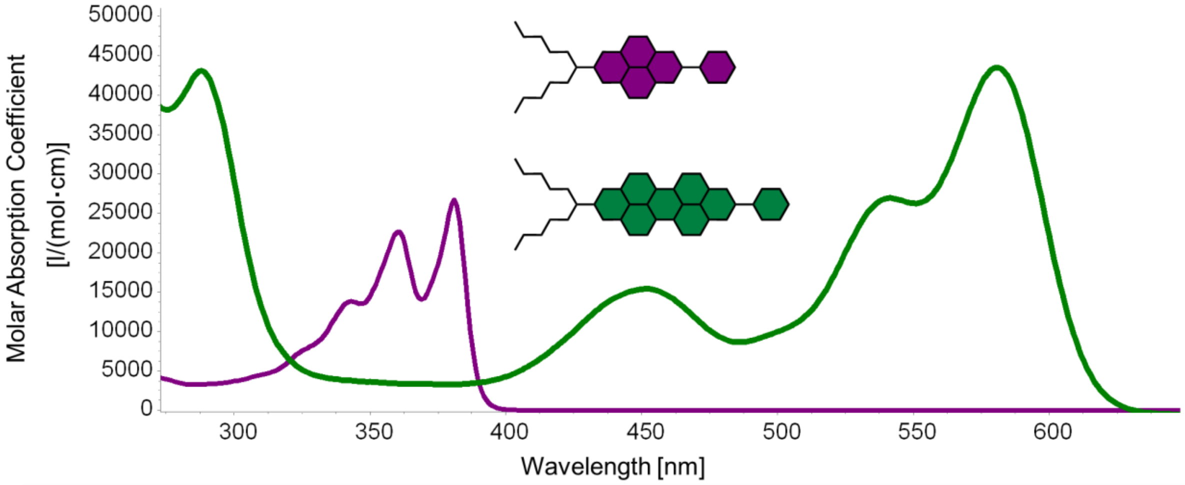

Figure S111. UV/Vis absorptions of **I-NDI** (purple) and **I-PDI** (green). Solvent: CH_2Cl_2

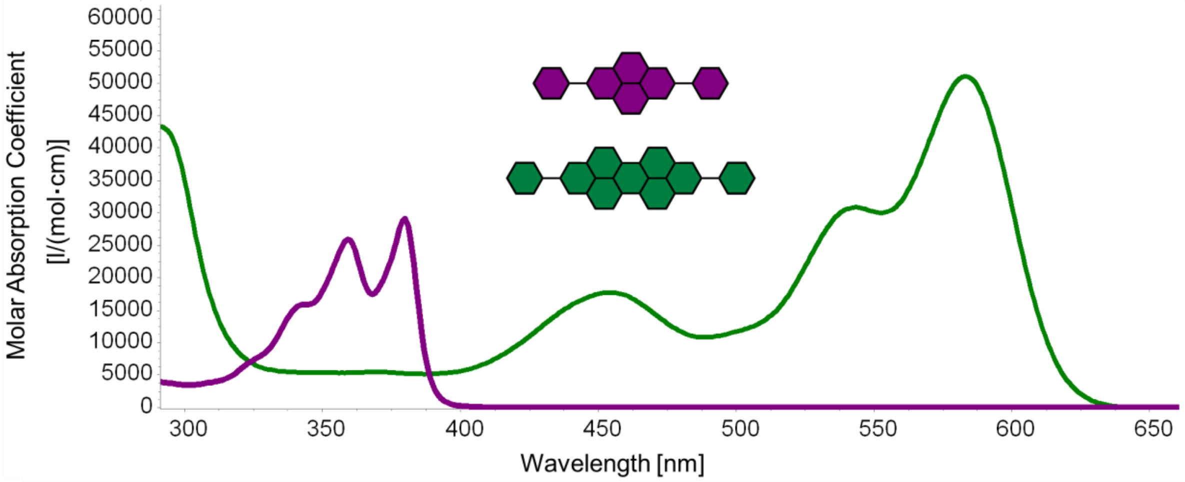

Figure S112. UV/Vis absorptions of **I-NDI-I** (purple) and **I-PDI-I** (green). Solvent: CH_2Cl_2

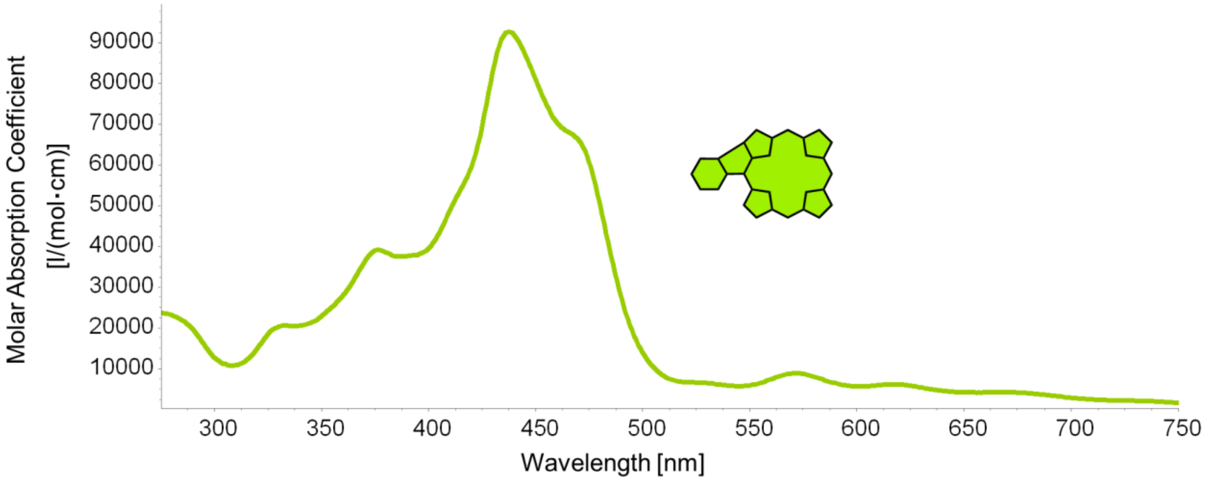

Figure S113. UV/Vis absorption of **PhBpin**. Solvent: CH_2Cl_2

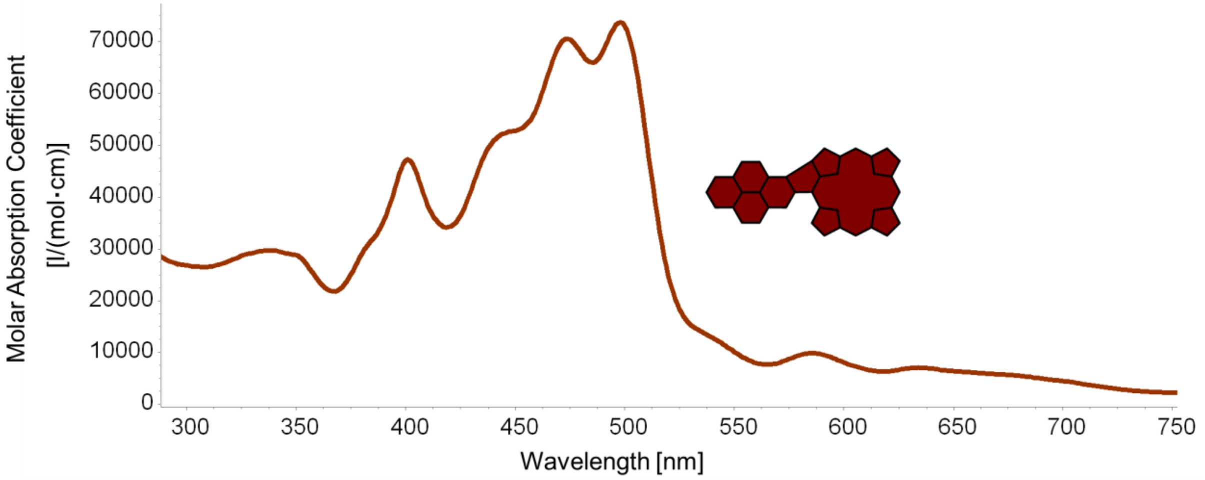

Figure S114. UV/Vis absorption of **PyrBpin**. Solvent: CH_2Cl_2

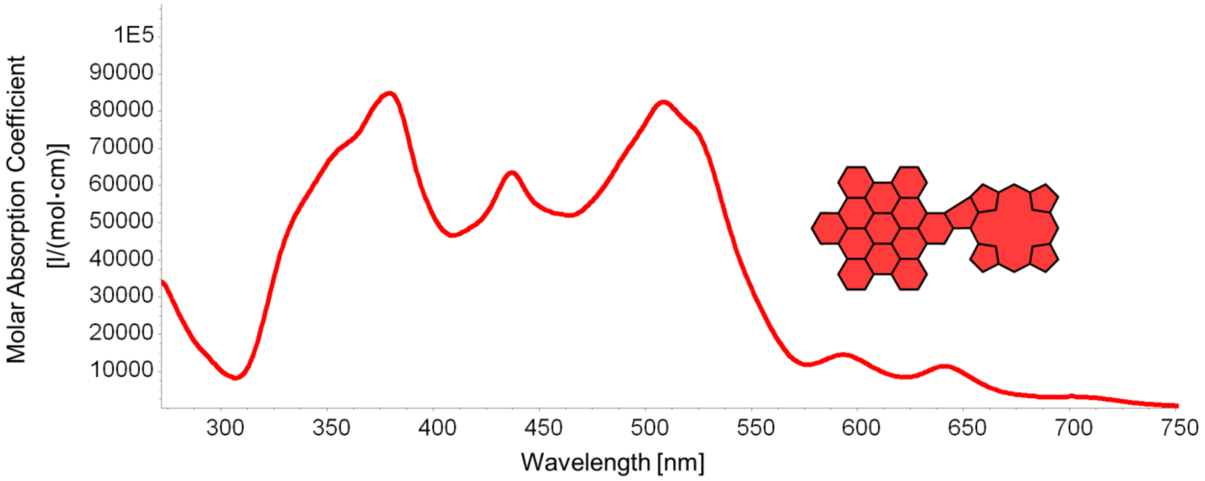

Figure S115. UV/Vis absorption of **HBCBpin**. Solvent: CH_2Cl_2

4 DFT Calculations

Geometries were relaxed using density-functional theory (DFT). The calculations were carried out with the plane-wave code PWScf of the Quantum Espresso software package,[10] utilizing the gradient-corrected Perdew-Burke-Ernzerhof (PBE) exchange-correlation functional,[11] Grimme D3 dispersion correction with Becke-Johnson damping,[12,13] Vanderbilt ultrasoft pseudopotentials,[14] and a plane-wave basis set with a kinetic energy cutoff of 30 Ry. Structures were assumed to be relaxed when a force convergence threshold of 5 meV/Å was reached.

Electronic properties were determined with the ORCA code,[15] using the B3LYP hybrid exchange-correlation functional,[16,17] the triple-zeta def2-TZVPP basis set,[18] and the RIJCOSX approximation with def2/J auxiliary basis functions.[19] Solvation effects in DCM were taken into account by employing the implicit conductor-like continuum polarization model (C-PCM).[15]

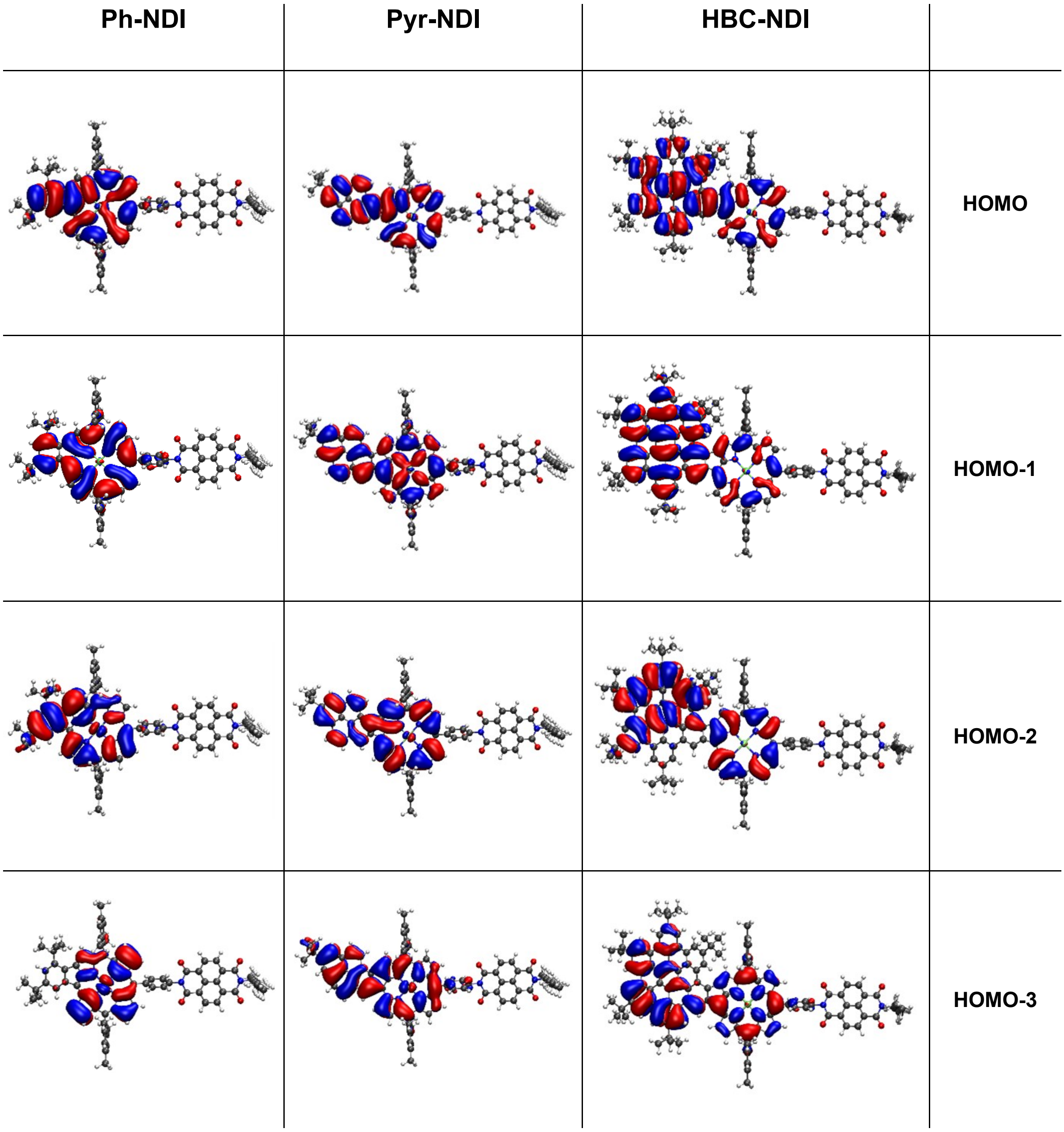

Figure S116. Geometry optimized structures and orbitals of **Ph-NDI**, **Pyr-NDI**, and **HBC-NDI**.

Ph-NDI	Pyr-NDI	HBC-NDI	
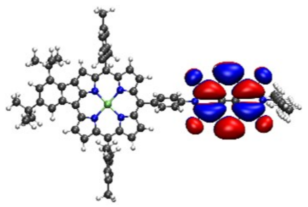	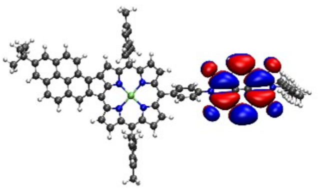	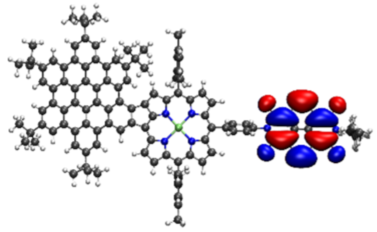	**LUMO**
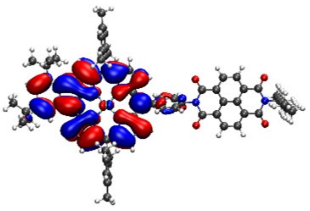	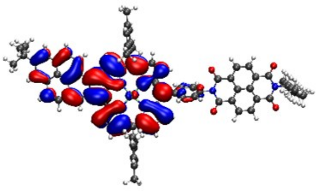	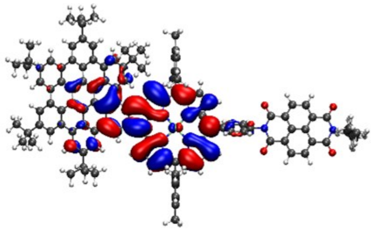	**LUMO+1**
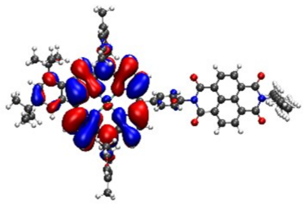	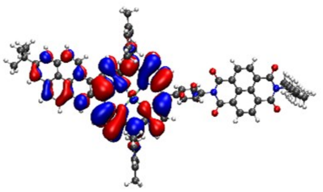	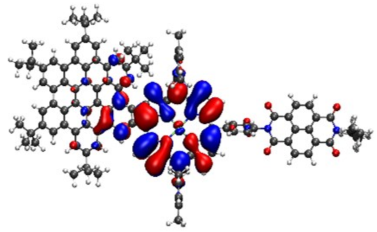	**LUMO+2**
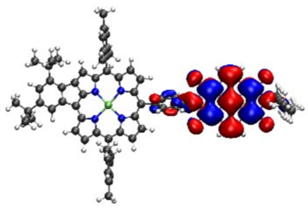	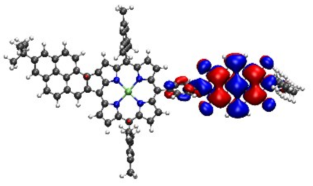	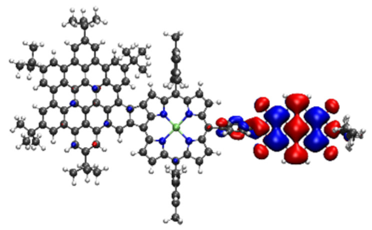	**LUMO+3**

Figure S117. Geometry optimized structures and orbitals of **Ph-NDI**, **Pyr-NDI**, and **HBC-NDI**.

Ph-PDI	Pyr-PDI	HBC-PDI	
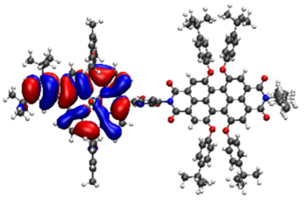	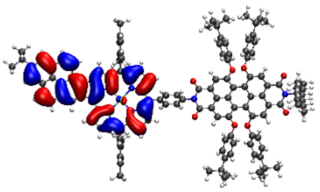	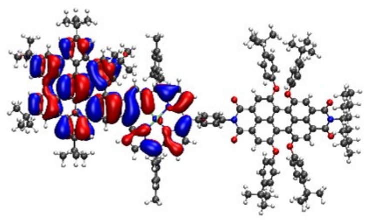	HOMO
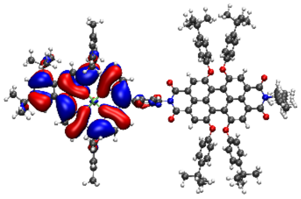	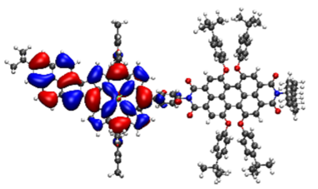	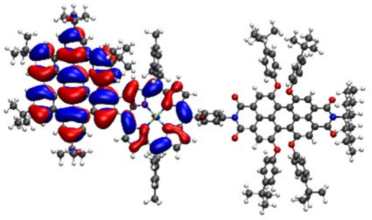	HOMO-1
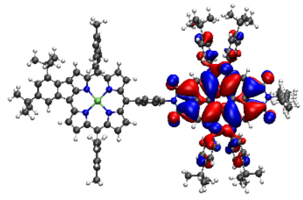	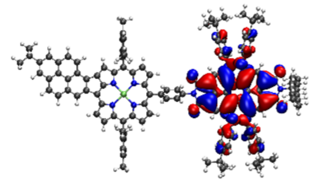	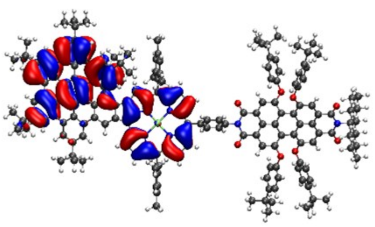	HOMO-2
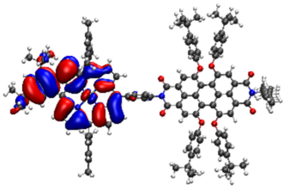	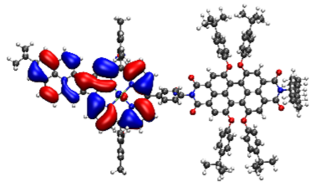	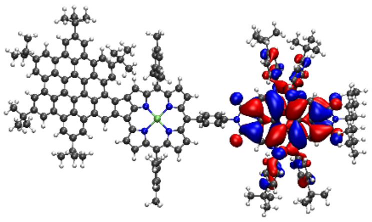	HOMO-3

Figure S118. Geometry optimized structures and orbitals of **Ph-PDI**, **Pyr-PDI**, and **HBC-PDI**.

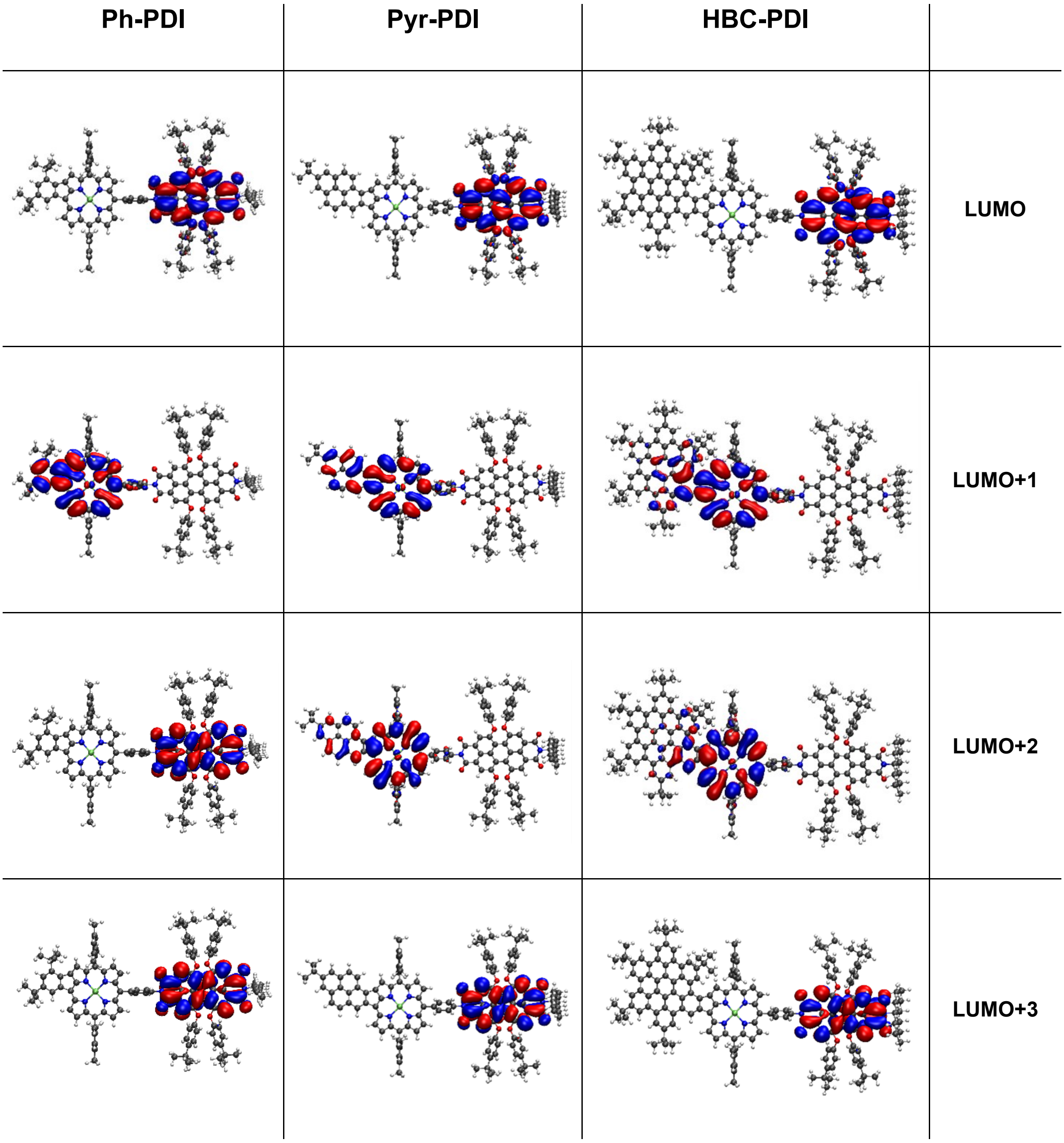

Figure S119. Geometry optimized structures and orbitals of **Ph-PDI**, **Pyr-PDI**, and **HBC-PDI**.

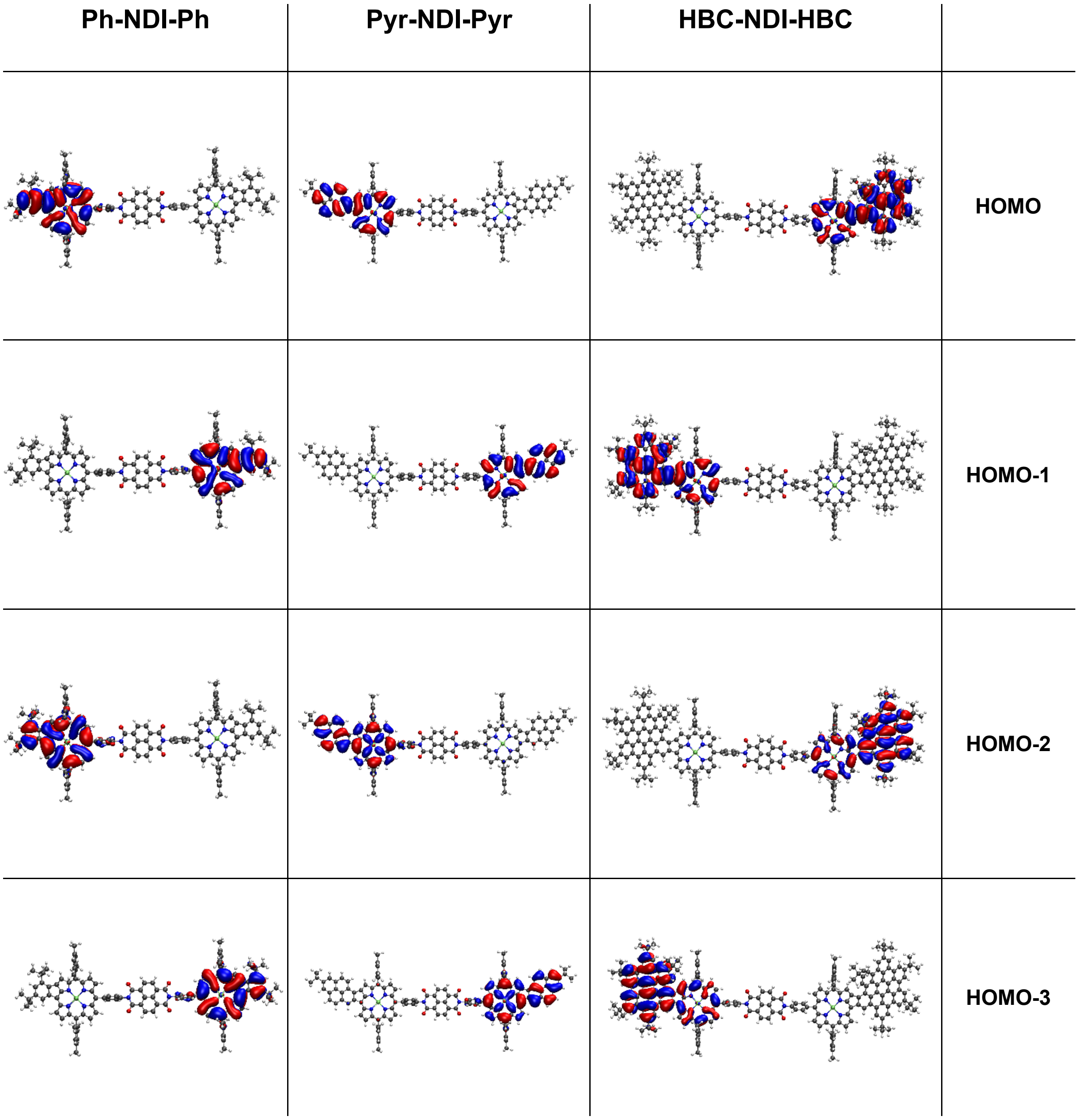

Figure S120. Geometry optimized structures and orbitals of **Ph-NDI-Ph**, **Pyr-NDI-Pyr**, and **HBC-NDI-HBC**.

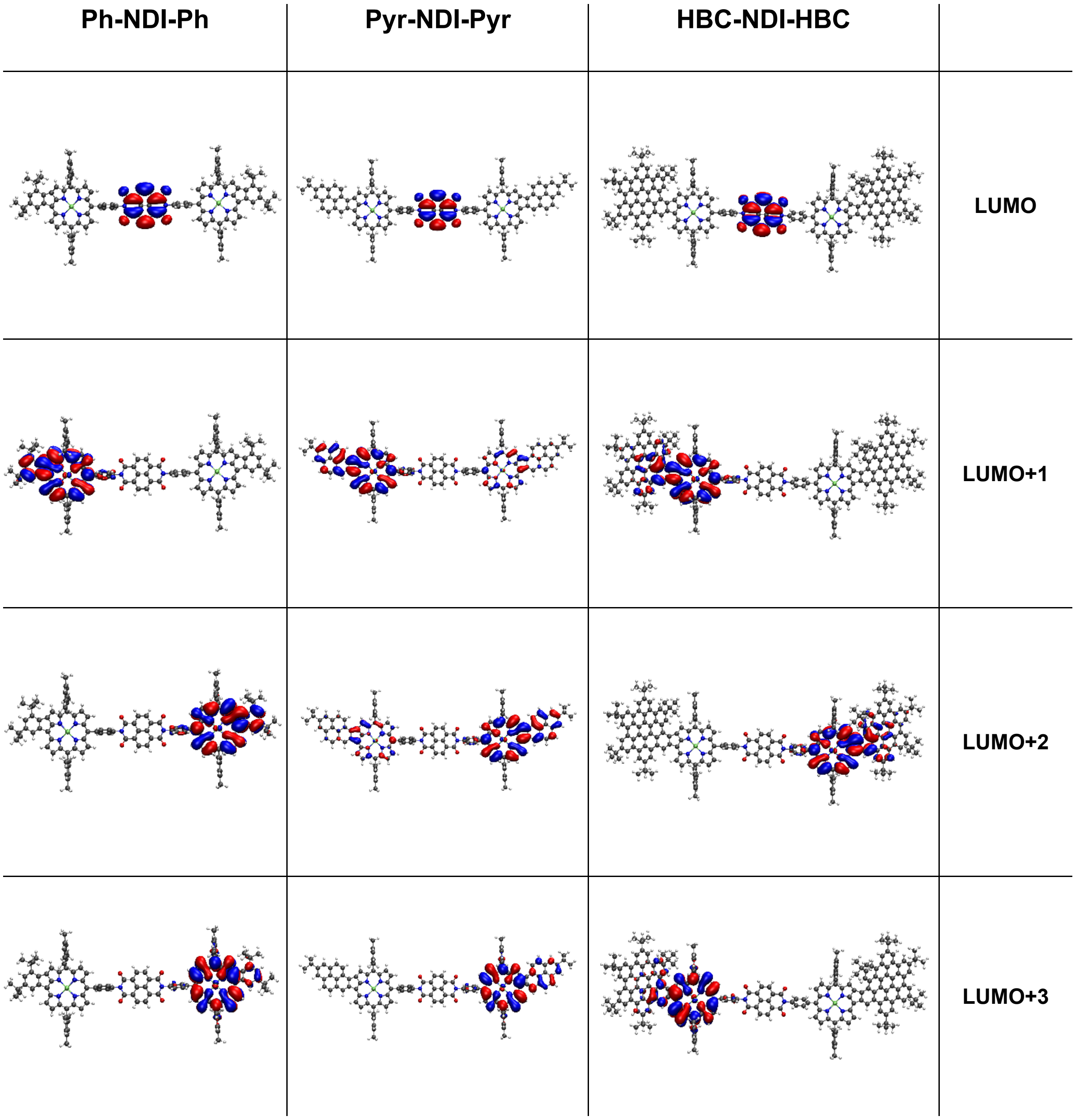

Figure S121. Geometry optimized structures and orbitals of **Ph-NDI-Ph**, **Pyr-NDI-Pyr**, and **HBC-NDI-HBC**.

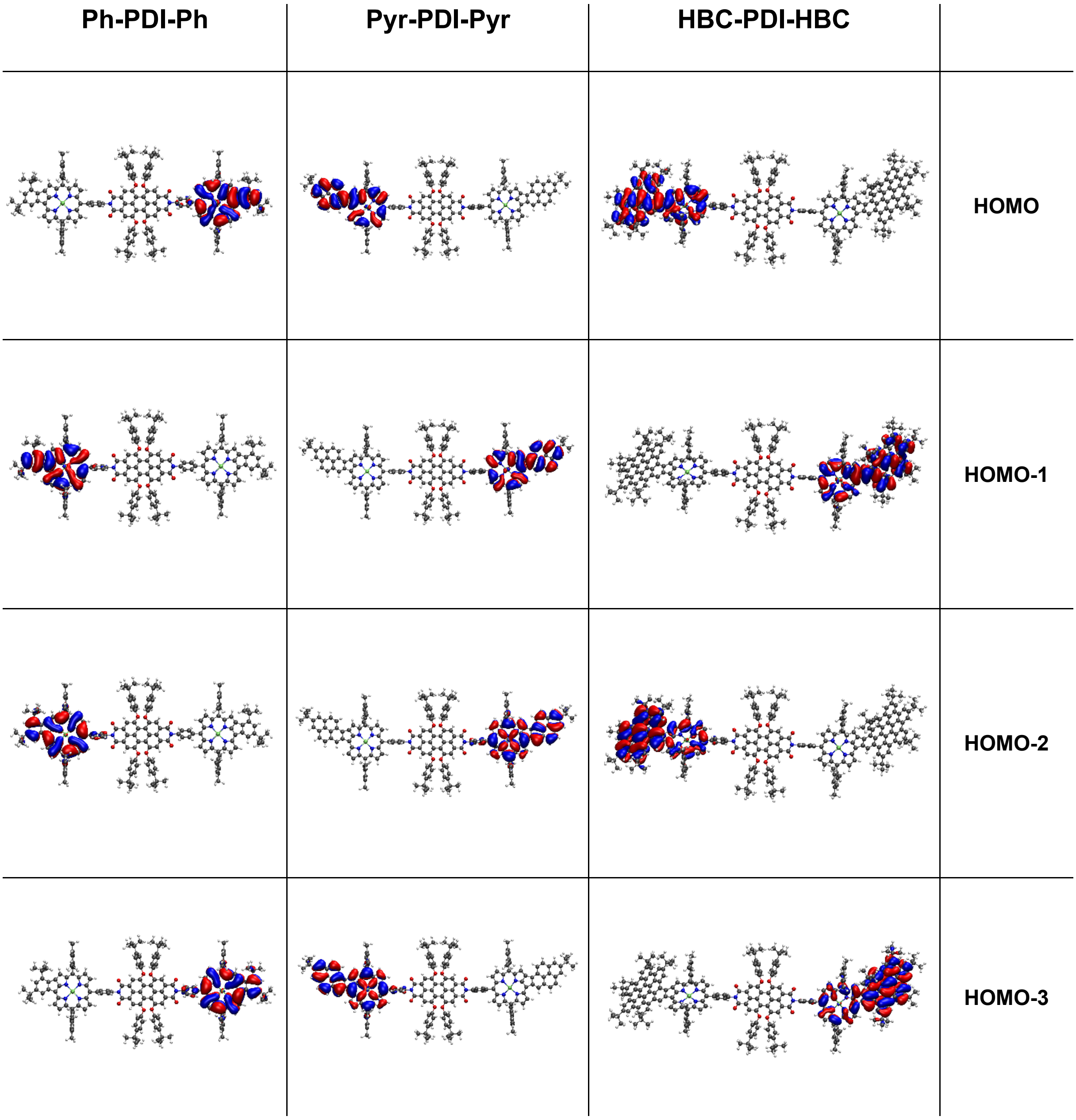

Figure S122. Geometry optimized structures and orbitals of **Ph-PDI-Ph**, **Pyr-PDI-Pyr**, and **HBC-PDI-HBC**.

Ph-PDI-Ph	Pyr-PDI-Pyr	HBC-PDI-HBC	
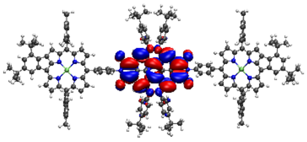	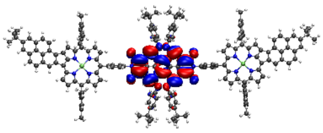	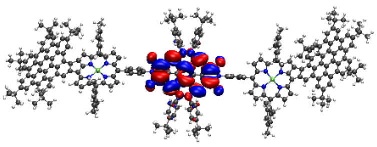	LUMO
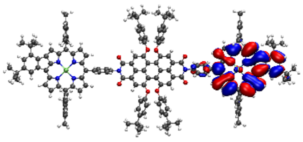	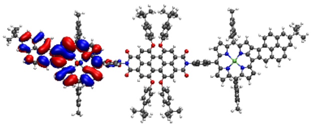	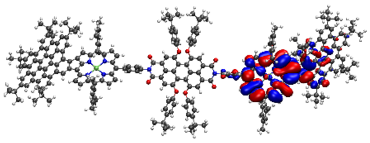	LUMO+1
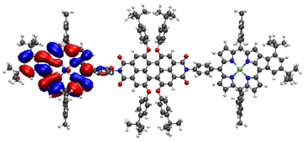	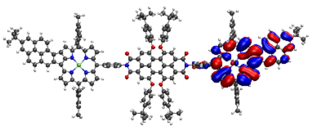	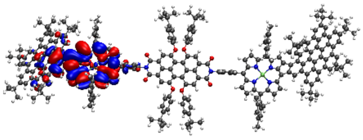	LUMO+2
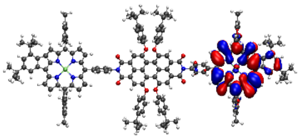	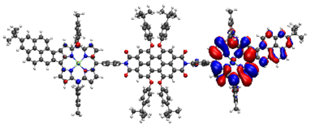	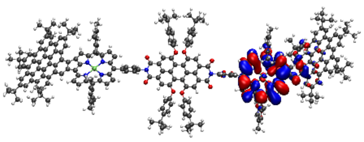	LUMO+3

Figure S123. Geometry optimized structures and orbitals of **Ph-PDI-Ph**, **Pyr-PDI-Pyr**, and **HBC-PDI-HBC**.

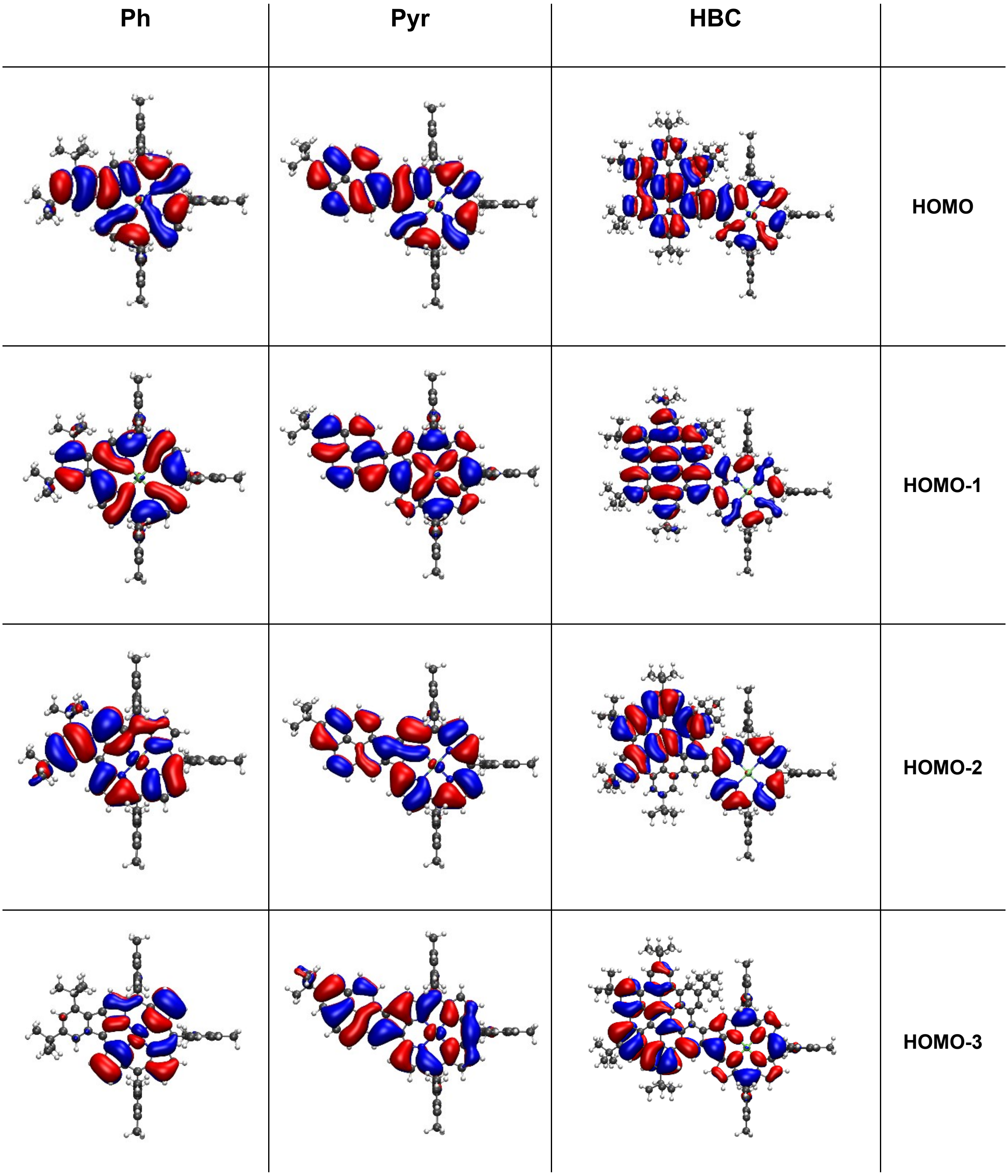

Figure S124. Geometry optimized structures and orbitals of **Ph**, **Pyr**, and **HBC**.

Ph	Pyr	HBC	
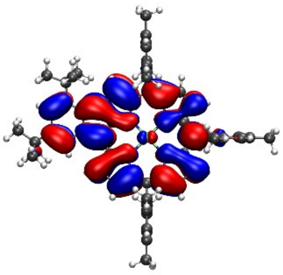	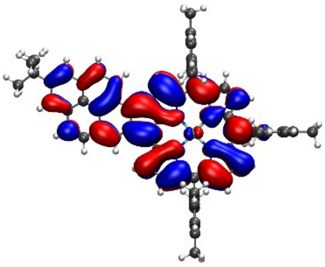	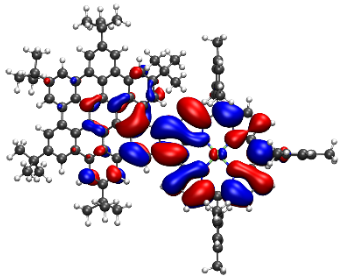	**LUMO**
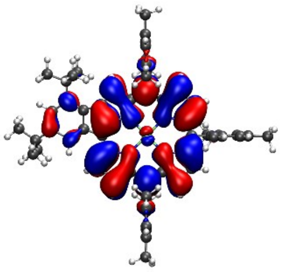	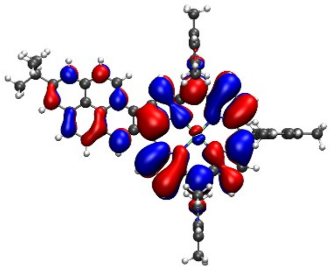	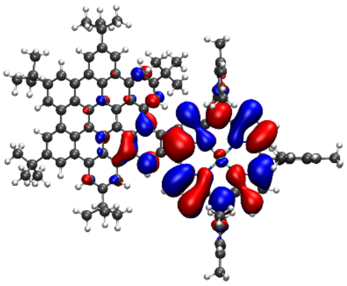	**LUMO+1**
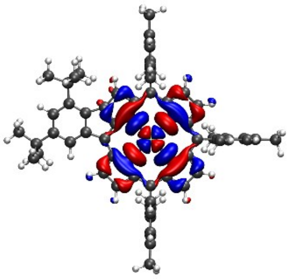	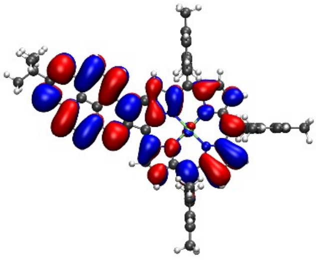	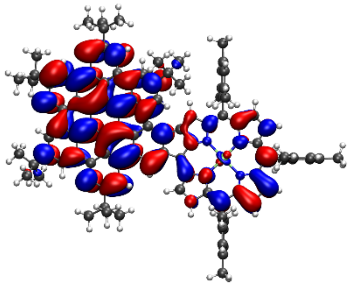	**LUMO+2**
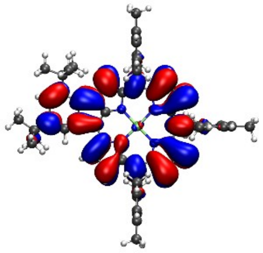	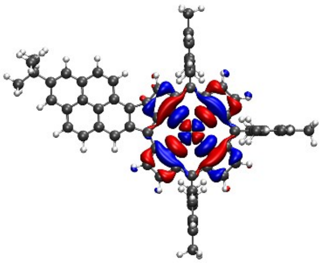	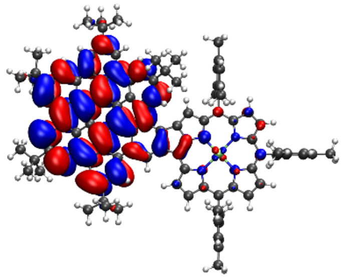	**LUMO+3**

Figure S125. Geometry optimized structures and orbitals of **Ph**, **Pyr**, and **HBC**.

NDI	PDI	
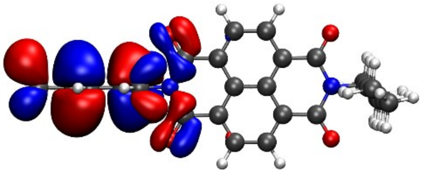	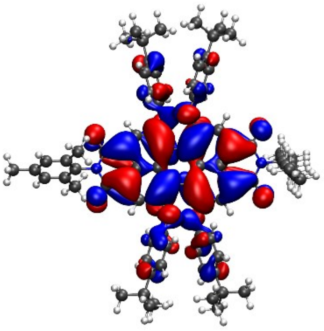	**HOMO**
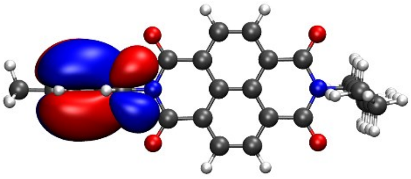	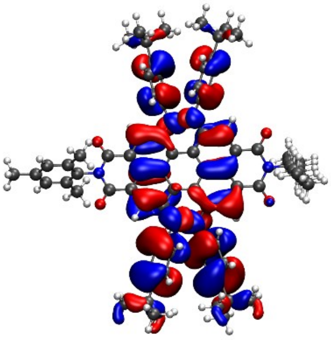	**HOMO-1**
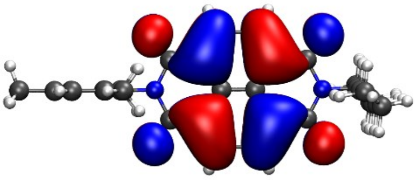	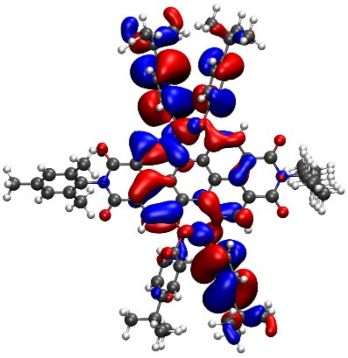	**HOMO-2**
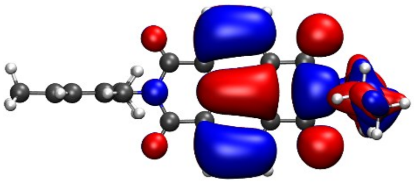	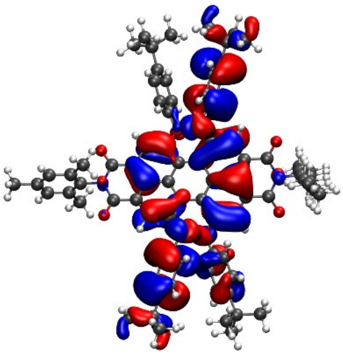	**HOMO-3**

Figure S126. Geometry optimized structures and orbitals of **NDI** and **PDI**.

NDI	PDI	
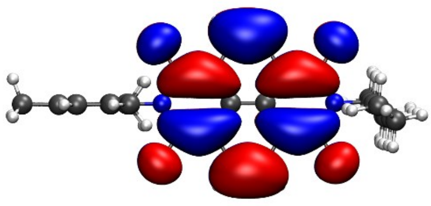	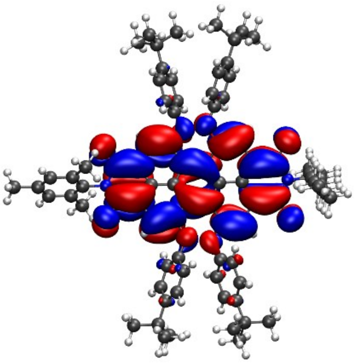	**LUMO**
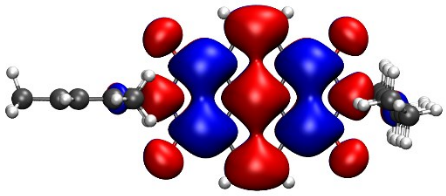	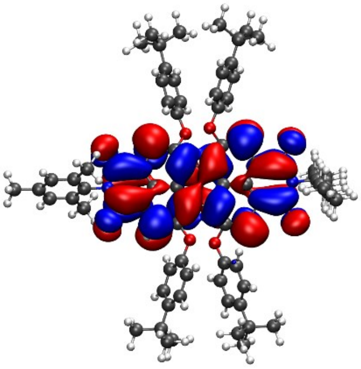	**LUMO+1**
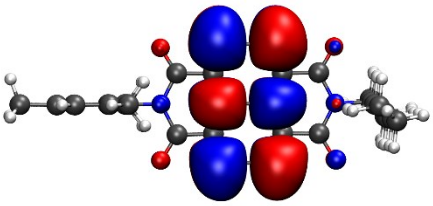	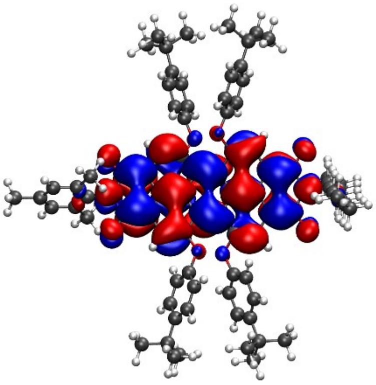	**LUMO+2**
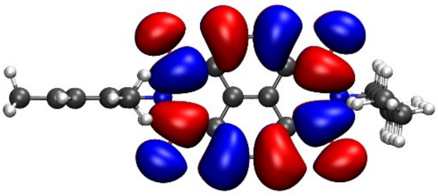	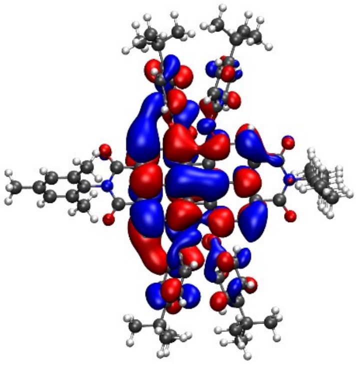	**LUMO+3**

Figure S127. Geometry optimized structures and orbitals of **NDI** and **PDI**.

Table S1. Energy eigenvalues of selected orbitals of **Ph-NDI**, **Pyr-NDI**, **HBC-NDI**, **Ph-PDI**, **Pyr-PDI**, and **HBC-PDI**.

	**Ph-NDI**	**Pyr-NDI**	**HBC-NDI**	**Ph-PDI**	**Pyr-PDI**	**HBC-PDI**
Orbital	Energy (eV)	Energy (eV)	Energy (eV)	Energy (eV)	Energy (eV)	Energy (eV)
HOMO-3	-6.354	-6.275	-5.608	-5.990	-5.773	-5.567
HOMO-2	-5.999	-5.777	-5.504	-5.565	-5.571	-5.499
HOMO-1	-5.517	-5.395	-5.255	-5.511	-5.394	-5.248
HOMO	-5.213	-5.131	-5.061	-5.203	-5.127	-5.057
LUMO	-3.548	-3.550	-3.557	-3.370	-3.372	-3.375
LUMO+1	-2.781	-2.870	-2.885	-2.768	-2.858	-2.873
LUMO+2	-2.496	-2.556	-2.560	-2.485	-2.545	-2.549
LUMO+3	-1.864	-1.868	-1.872	-2.189	-2.193	-2.194
GAP	1.665	1.581	1.504	1.833	1.754	1.682

Table S2. Energy eigenvalues of selected orbitals of **Ph-NDI-Ph**, **Pyr-NDI-Pyr**, **HBC-NDI-HBC**, **Ph-PDI-Ph**, **Pyr-PDI-Pyr,** and **HBC-PDI-HBC**.

	**Ph-NDI-Ph**	**Pyr-NDI-Pyr**	**HBC-NDI-HBC**	**Ph-PDI-Ph**	**Pyr-PDI-Pyr**	**HBC-PDI-HBC**
Orbital	Energy (eV)	Energy (eV)	Energy (eV)	Energy (eV)	Energy (eV)	Energy (eV)
HOMO-3	-5.525	-5.406	-5.255	-5.511	-5.389	-5.250
HOMO-2	-5.524	-5.406	-5.254	-5.506	-5.389	-5.248
HOMO-1	-5.213	-5.134	-5.060	-5.205	-5.127	-5.054
HOMO	-5.212	-5.133	-5.059	-5.201	-5.126	-5.053
LUMO	-3.607	-3.615	-3.616	-3.405	-3.409	-3.405
LUMO+1	-2.783	-2.869	-2.886	-2.771	-2.857	-2.877
LUMO+2	-2.782	-2.869	-2.884	-2.766	-2.856	-2.873
LUMO+3	-2.496	-2.556	-2.561	-2.486	-2.545	-2.550
GAP	1.604	1.518	1.443	1.795	1.717	1.648

Table S3. Energy eigenvalues of selected orbitals of **Ph**, **Pyr**, **HBC, NDI**, and **PDI**.

	**Ph**	**Pyr**	**HBC**	**NDI**	**PDI**
Orbital	Energy (eV)	Energy (eV)	Energy (eV)	Energy (eV)	Energy (eV)
HOMO-3	-6.333	-6.264	-5.595	-7.545	-6.288
HOMO-2	-5.981	-5.757	-5.493	-7.015	-6.219
HOMO-1	-5.497	-5.383	-5.247	-6.683	-6.000
HOMO	-5.192	-5.116	-5.052	-6.644	-5.560
LUMO	-2.750	-2.841	-2.858	-3.525	-3.359
LUMO+1	-2.466	-2.527	-2.533	-1.817	-2.182
LUMO+2	-1.614	-1.761	-1.825	-1.283	-1.798
LUMO+3	-1.312	-1.657	-1.797	-1.262	-1.031
GAP	2.442	2.275	2.194	3.118	2.201

5 References

[1] Q. Chen, L. Brambilla, L. Daukiya, K. S. Mali, S. de Feyter, M. Tommasini, K. Müllen, A. Narita, *Angew. Chemie, Int. Ed. Engl.* **2018**, *57*, 11233.

[2] R. Mishra, R. Regar, R. Singhal, P. Panini, G. D. Sharma, J. Sankar, *J. Mater. Chem. A* **2017**, *5*, 15529.

[3] D. N. Coventry, A. S. Batsanov, A. E. Goeta, J. A. K. Howard, T. B. Marder, R. N. Perutz, *Chem. Commun.* **2005**, 2172.

[4] E. K. Perttu, M. Arnold, P. M. Iovine, *Tetrahedron* **2005**, *46*, 8753.

[5] M. M. Martin, D. Lungerich, P. Haines, F. Hampel, N. Jux, *Angew. Chemie, Int. Ed. Engl.* **2019**, *58*, 8932.

[6] M. M. Martin, C. Dusold, A. Hirsch, N. Jux, *J. Porphyrins Phthalocyanines* **2020**, *24*, 268.

[7] E. L. Spitler, J. W. Colson, F. J. Uribe-Romo, A. R. Woll, M. R. Giovino, A. Saldivar, W. R. Dichtel, *Angew. Chemie, Int. Ed. Engl.* **2012**, *51*, 2623.

[8] E. J. Schulze, C. L. Ritterhoff, E. Franz, O. Tavlui, O. Brummel, B. Meyer, A. Hirsch, *Chem. Eur. J.* **2024**, *30*, e202303515.

[9] F. Schlosser, M. Moos, C. Lambert, F. Würthner, *Adv. Mater.* **2013**, *25*, 410.

[10] P. Giannozzi, S. Baroni, N. Bonini, M. Calandra, R. Car, C. Cavazzoni, D. Ceresoli, G. L. Chiarotti, M. Cococcioni, I. Dabo *et al.*, *J. Phys. Condens. Matter* **2009**, *21*, 395502.

[11] J. P. Perdew, K. Burke, M. Ernzerhof, *Phys. Rev. Lett.* **1996***, 77,* 3865–3868.

[12] S. Grimme, S. Ehrlich, L. Goerigk, *J. Comput. Chem.* **2011***, 32,* 1456–1465.

[13] S. Grimme, J. Antony, S. Ehrlich, H. Krieg, *J. Chem. Phys.* **2010**, *132*, 154104.

[14] D. Vanderbilt, *Phys. Rev. B* **1990***, 41,* 7892–7895.

[15] F. Neese, *WIREs Comput. Mol. Sci.* **2012***, 2,* 73–78.

[16] A. D. Becke, *J. Chem. Phys.* **1993***, 98,* 1372–1377.

[17] C. Lee, W. Yang, R. G. Parr, *Phys. Rev. B* **1988***, 37,* 785–789.

[18] F. Weigend, R. Ahlrichs, *Phys. Chem. Chem. Phys.* **2005**, *7*, 3297.

[19] F. Neese, F. Wennmohs, A. Hansen, U. Becker, *Chem. Phys.* **2009**, *356*, 98–109.
